# Genetic diversity of circumsporozoite protein in *Plasmodium knowlesi* isolates from Malaysian Borneo and Peninsular Malaysia

**DOI:** 10.1186/s12936-020-03451-x

**Published:** 2020-10-22

**Authors:** Eric Tzyy Jiann Chong, Joveen Wan Fen Neoh, Tiek Ying Lau, Yvonne Ai-Lian Lim, Hwa Chia Chai, Kek Heng Chua, Ping-Chin Lee

**Affiliations:** 1 Biotechnology Programme, Faculty of Science and Natural Resources, Universiti Malaysia Sabah, Jalan UMS, 88400 Kota Kinabalu, Sabah Malaysia; 2 Biotechnology Research Institute, Universiti Malaysia Sabah, Jalan UMS, 88400 Kota Kinabalu, Sabah Malaysia; 3 Department of Parasitology, Faculty of Medicine, University of Malaya, 50603 Kuala Lumpur, Malaysia; 4 Centre of Excellence for Research in AIDS (CERiA), University of Malaya, 50603 Kuala Lumpur, Malaysia; 5 Department of Biomedical Science, Faculty of Medicine, University of Malaya, 50603 Kuala Lumpur, Malaysia

**Keywords:** Plasmodium knowlesi, Circumsporozoite protein, genetic diversity, Malaysian Borneo

## Abstract

**Background:**

Understanding the genetic diversity of candidate genes for malaria vaccines such as circumsporozoite protein (*csp*) may enhance the development of vaccines for treating *Plasmodium knowlesi*. Hence, the aim of this study is to investigate the genetic diversity of non-repeat regions of *csp* in *P. knowlesi* from Malaysian Borneo and Peninsular Malaysia.

**Methods:**

A total of 46 *csp* genes were subjected to polymerase chain reaction amplification. The genes were obtained from *P. knowlesi* isolates collected from different divisions of Sabah, Malaysian Borneo, and Peninsular Malaysia. The targeted gene fragments were cloned into a commercial vector and sequenced, and a phylogenetic tree was constructed while incorporating 168 *csp* sequences retrieved from the GenBank database. The genetic diversity and natural evolution of the *csp* sequences were analysed using MEGA6 and DnaSP ver. 5.10.01. A genealogical network of the *csp* haplotypes was generated using NETWORK ver. 4.6.1.3.

**Results:**

The phylogenetic analysis revealed indistinguishable clusters of *P. knowlesi* isolates across different geographic regions, including Malaysian Borneo and Peninsular Malaysia. Nucleotide analysis showed that the *csp* non-repeat regions of zoonotic *P. knowlesi* isolates obtained in this study underwent purifying selection with population expansion, which was supported by extensive haplotype sharing observed between humans and macaques. Novel variations were observed in the C-terminal non-repeat region of *csp*.

**Conclusions:**

The *csp* non-repeat regions are relatively conserved and there is no distinct cluster of *P. knowlesi* isolates from Malaysian Borneo and Peninsular Malaysia. Distinctive variation data obtained in the C-terminal non-repeat region of *csp* could be beneficial for the design and development of vaccines to treat *P. knowlesi*.

## Background

Malaria in humans is caused by *Plasmodium* species, including *Plasmodium vivax*, *Plasmodium ovale* spp.*, Plasmodium malariae*, *Plasmodium falciparum,* and *Plasmodium knowlesi*. *Plasmodium* parasites also infect crab-eating macaque (*Macaca fascicularis*), also known as the long-tailed macaque, and southern pig-tailed macaque (*Macaca nemestrina*). Thus, malaria is considered an emerging zoonotic disease. Humans may acquire these parasites when they are close to the habitats of macaques infected with the parasites through anopheline mosquito vectors in forests. Most recently, the World Malaria Report 2019 estimates that 228 million new malaria cases and 405,000 deaths from malaria occurred around the world in 2018 [1]. These data are at odds with the vision set by the World Health Organization in early 2015, which has a goal of at least a 90% reduction of the global malaria incidence and mortality rates by 2030 [2].

Malaysia is vulnerable to malaria transmission since it is located in a hot and humid equatorial region. It is estimated that about 1.26 million people in Malaysia are living in hyperendemic areas and have high risk of contracting malaria [1]. Previously, there has been a large focus on molecularly validated *P. knowlesi* infections in humans that were misidentified as *P. malariae* by microscopy observations due to their similar morphological characteristics [3, 4]. Since then, human infections by *P. knowlesi* have been reported in Malaysia [5–7], and increased detection of the infections in the country has recently been documented [8–11]. Besides Malaysia, *P. knowlesi* infections in humans have also been reported in other Southeast Asian countries [12]. Therefore, malaria should be prevented or controlled in these areas. There are ways to control and prevent malaria. One of these proposed methods can be vaccine production. Thus, a more effective vaccine is urgently needed to control the transmission of *P. knowlesi*.

Circumsporozoite protein (*csp*) is one of the targeted candidates for vaccine development in treating malaria. This protein is multifunctional in malaria transmission, including mediation of sporozoite development and assisting sporozoite migration from mosquitoes’ midguts to mammalian livers [13–15]. The *csp* gene has been shown to be a useful biomarker for delineating the phylogenetic relationship of *Plasmodium* species [16, 17], and a recent study investigated the genetic diversity of *csp* between *P. knowlesi* isolates from Malaysian Borneo and Peninsular Malaysia [18]. However, the study was limited to only *P. knowlesi* isolates obtained from the Interior Division of Sabah in Malaysian Borneo. To obtain more accurate general interpretations, the present study adds new *P. knowlesi* isolates from the Sandakan Division of Sabah (another malaria hotspot area) to reveal more information about the genetic diversity of *csp* non-repeat regions between *P. knowlesi* isolates from Malaysian Borneo and Peninsular Malaysia.

## Methods

### Human blood samples and *Plasmodium* DNA extraction

A total of 46 human blood samples infected with *P. knowlesi* were collected from symptomatic malaria patients with informed consent in Sabah, Malaysian Borneo, from 2008 to 2011 (Table 1). The sample areas included Sandakan Division (13 samples from Telupid Health Clinic), Interior Division (6 samples from Keningau Hospital, 3 samples from Nabawan Health Clinic, 5 samples from Tambunan Hospital, and 6 samples from Tenom Hospital), and the University of Malaya Medical Centre, Peninsular Malaysia (*N* = 13). The presence of *P. knowlesi* parasites in the samples was confirmed by an experienced pathologist and verified using the PlasmoNex™ diagnostic system [19]. *Plasmodium* DNA was extracted using a previously described approach [20]. Ethical approval for this study was obtained from the Ethics Committee of University of Malaya Medical Centre (reference no. 709.2).

**Table 1:** *Plasmodium knowlesi* isolates collected in this study

Areas	Samples
Sabah, Malaysian Borneo (*N* = 33)	
Sandakan Division	SB28, SB35, SB39, SB55, SB130, SB131, SB132, SB143, SB145, SB148, SB150, SB151, SB156
Interior Division	KN010, KN013, KN030, KN037, KN045, KN048, NB028, NB032, NB046, TB013, TB016, TB049, TB066, TB073, TN003, TN023, TN024, TN026, TN027, TN029
Peninsular Malaysia (*N* = 13)	5668, 5687, 5823, 5868, 6835, 6854, 6873, 7341, 7681, 7894, 7412, 8183, 8939

### Polymerase chain reaction (PCR) amplification of *csp* gene

PCR amplification was carried out using a 20-μL reaction mixture containing 1X *GoTaq*^®^ buffer (Promega, USA), 2.0 mM of MgCl_2_ solution, 0.2 mM of dNTPs, 0.2 μM of each primer, and 1.25 units of *GoTaq*^®^ DNA polymerase (Promega, USA). The primers used for *csp* gene amplification were Pkcsp-F: 5′-TCC TCC ACA TAC TTA TAT ACA AGA-3′ and Pkcsp-R: 5′-GTA CCG TGG GGG ACG CCG-3′, which were derived from a previous study [3]. The PCR conditions were 94 °C for 4 min followed by 40 amplification cycles at 94 °C for 30 s, 55 °C for 50 s, and 72 °C for 2 min, and a final extension step at 72 °C for 10 min. PCR amplicons were subjected to electrophoresis and analysed in 1% agarose gel stained with ethidium bromide.

### Molecular cloning and sequencing

A QIAquick Gel Extraction Kit (Qiagen, Germany) was used to isolate PCR products from the agarose gel, and the purified PCR products were cloned into the pJET1.2/blunt vector using a CloneJET PCR Cloning Kit (Thermo Scientific, USA) according to manufacturer’s instructions. The ligased vectors were transformed into *Escherichia coli* strain JM109 using a conventional heat-shock method. The desired plasmid containing the *csp* gene from a single colony was extracted using a QIAprep Spin Miniprep Kit (Qiagen, Germany) according to the manufacturer’s recommendations and subjected to sequencing using pJET1.2 forward sequencing primer.

### Sequence alignment and phylogenetic analysis of *csp* gene

In addition to the 46 *csp* sequences obtained in this study, a total of 168 *csp* sequences (Additional file 1) were retrieved from the GenBank database, including 33 sequences from macaques in Sarawak, 24 sequences from macaques in Singapore, 62 sequences from humans in Peninsular Malaysia, 33 sequences from humans in Sarawak, 14 sequences from humans in Singapore, and 2 sequences as an outgroup. Alignment was performed using the CLUSTAL-W tool in Molecular Evolutionary Genetic Analysis 6 (MEGA6) software [21]. Next, the central repeat region of the *csp* gene was trimmed, whereas the N-terminal (first 195 bp of the coding *csp* gene) and the C-terminal (last 261 bp of the coding *csp* gene with exception of the stop codon) non-repeat regions were combined (total length = 453 bp) for the analysis. The maximum likelihood method was used to construct a phylogenetic tree with 1000 bootstrap replicates to test the robustness and reliability of the tree.

### *Csp* sequence diversity, natural selection, and haplotype analyses

Variation in the combined *csp* sequences was determined using DnaSP ver. 5.10.01 [22]. The data obtained included the average number of pairwise nucleotide differences (K), number of haplotypes (h), haplotype diversity (Hd), and nucleotide diversity (π). An advanced analysis of π was also performed on a sliding window of 100 bases with a step size of 25 bp to estimate the step-wise diversity of *csp*. In addition, the rates of synonymous (d_S_) and non-synonymous (d_N_) mutations were obtained and compared with a Z-test in MEGA6 using Nei and Gojobori’s approach [23] with Jukes and Cantor correction. For testing the neutral theory of evolution, Tajima’s D test [24] as well as Fu and Li’s D and F tests [25] were performed using DnaSP ver. 5.10.01. The median-joining star contraction approach in the software NETWORK ver. 4.6.1.3 [26] was used to generate the relationship of *csp* haplotypes for isolates obtained in this study.

## Results

**Fig. 1:**
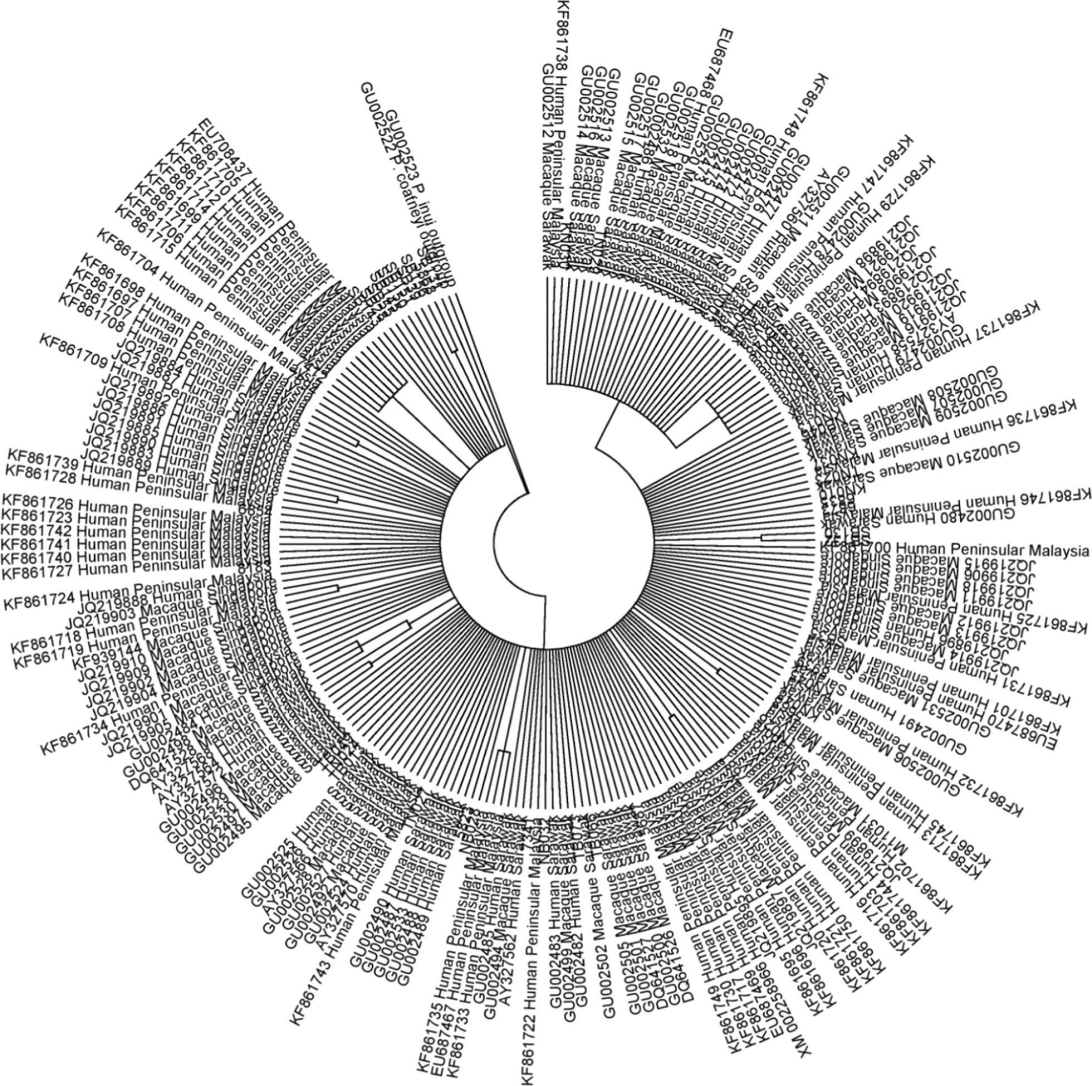
Phylogenetic tree of *csp* gene sequences constructed using the Maximum likelihood method in MEGA6. Phylogenetic tree was constructed with support of 1000 bootstrap replicates

A total of 214 *csp* combined sequences were included in the phylogenetic tree analysis. The tree revealed indistinguishable geographical clusters of the *P. knowlesi* isolates included in this study (Fig. 1). A distinct cluster was observed for outgroups, and the *P. knowlesi* isolates were genetically closer to *Plasmodium coatneyi* based on the non-repeat *csp* sequence.

**Fig. 2:**
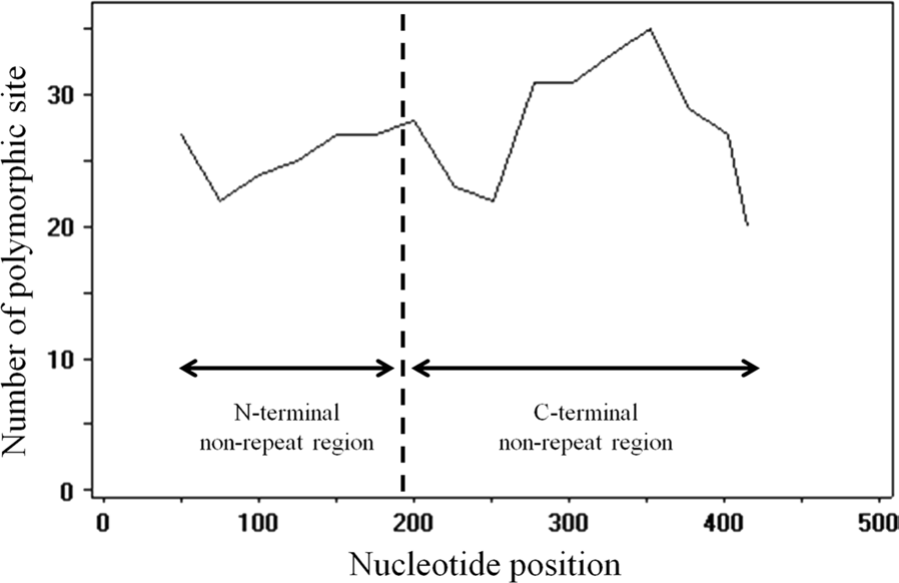
Nucleotide segregating in the *csp* non-repeat regions of *P. knowlesi*. Sliding window plot with a window length of 100 bp and step size of 25 bp for number of segregating site within the aligned *csp* sequences was generated using DnaSP ver 5.10.01

The nucleotide alignment of the *csp* in this study showed that the average number of pairwise nucleotide differences (K) was 8.958. The overall nucleotide diversity (π) and haplotype diversity (Hd) were 0.0199 ± 0.0005 and 0.9822 ± 0.0029, respectively. The detailed analysis of π with a sliding window length of 100 bp and step size of 25 bp revealed that the nucleotide position at 303–402 bp had the highest peak of nucleotide diversity (Fig. 2). The average rates of synonymous mutation (d_S_) and non-synonymous mutation (d_N_) were 0.037 and 0.015, respectively, and the d_N_/d_S_ ratio was 0.405 (d_S_ > d_N_; *p* < 0.05 in Z-test). In testing the neutral theory of evolution, Tajima’s D was − 1.830 (*p* < 0.05), whereas Fu and Li’s D and F were − 7.152 and − 5.519 (both *p*-values < 0.02), respectively.

**Fig. 3:**
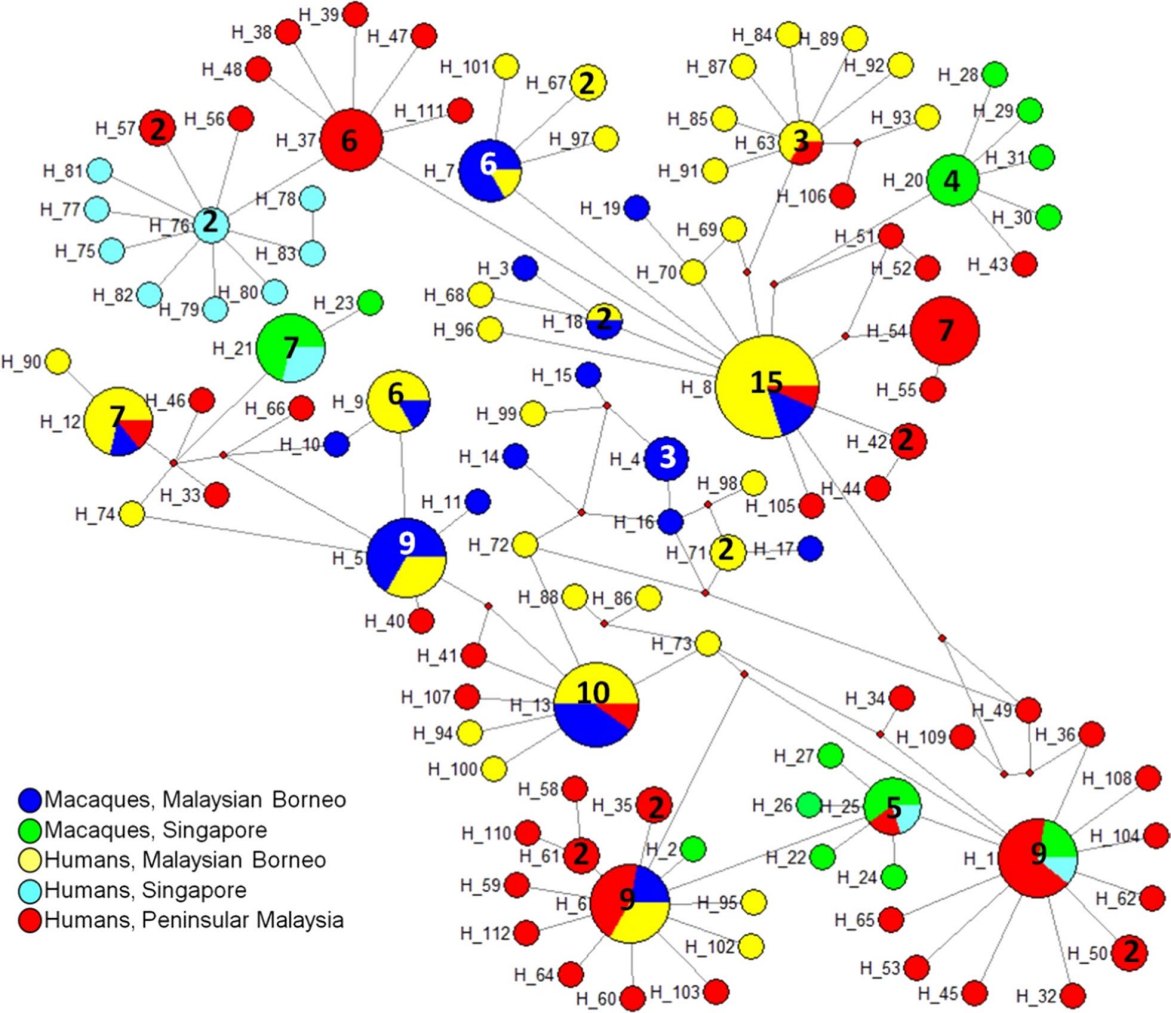
Median-joining star contraction network of *csp* haplotypes (h = 112). The total number of haplotype frequencies is represented in larger circles and unnumbered circles indicated a single haplotype

Further amino acid analysis showed totals of 28 and 35 polymorphic sites in the N-terminal and C-terminal non-repeat regions, respectively (Additional file 2). For the N-terminal non-repeat region, there were 3 dimorphic changes [L18(F,P); S29(T,P); V34(I,L)] and 25 monomorphic changes. For the C-terminal non-repeat region, there were 6 dimorphic changes [H288(Q,R); T296(I,A); A315(G,Q); N319(K,R); D324(N,E); V332(A,L)] and 29 monomorphic changes. The *csp* amino acid sequences were categorized into 112 different haplotypes. In the network analysis, high frequencies of haplotype sharing were observed for *P. knowlesi* isolates between Malaysian Borneo and Peninsular Malaysia (i.e. H_6, H_8, H_12, H_13, and H_63), as well as between humans and macaques (i.e. H_1, H_5, etc.) (Fig. 3).

## Discussion

The *csp* protein densely coats the surface of *Plasmodium* parasites and has been reported to play an important role in sporozoite development and mammalian hepatocyte invasion [13, 14]. The central repeat region of *csp* is located between N-terminal and C-terminal non-repeat regions along the gene [27], which have been found with more than 40 various repeat units in different arrangements and lengths [28]. This study solely focuses on the non-repeat regions of the *csp* gene because the highly polymorphic central repeat regions may introduce biasness and lead to misinterpretation in the phylogenetic and genetic diversity analyses.

No distinct geographical separation was observed in the phylogenetic tree. This suggests the similarity of the *P. knowlesi* isolates across different regions, including Malaysian Borneo and Peninsular Malaysia, as well as between humans and macaques based on the *csp* non-repeat regions. The findings are comparable to previous data [29–31]. *Plasmodium knowlesi* has been hypothesized to have migrated from the mainland of Southeast Asia to Malaysian Borneo during the Pleistocene era through migrating wild macaques when the two regions were still connected together [32, 33]. The present study further supports the hypothesis with the phylogenetically indistinguishable of *P. knowlesi* isolates observed in these regions. Similar to previous studies [5, 17, 29, 30], *P. knowlesi* was found to be genetically closer to *P. coatneyi*, indicating reliability of the constructed phylogenetic tree using the *csp* non-repeat regions.

The average number of pairwise nucleotide differences (K), nucleotide diversity (π) and haplotype diversity (Hd) for the combined non-repeat regions in this study were slightly higher than those reported by Fong et al. [18]. The sliding window plot with a length of 100 bp and step size of 25 bp showed that the most diverse region was within the C-terminal non-repeat region represented by high amino acid variations in this region. The RTS,S malaria vaccine, which is in Phase III trials, targets the T-cell epitopes in the C-terminal region, and recent studies reported modest results in treating *P. falciparum* [34, 35]. However, insufficient study was conducted for *P. knowlesi*. Therefore, the variation data of the C-terminal non-repeat region in this study could be beneficial for vaccine development to treat *P. knowlesi*.

The d_N_/d_S_ ratio revealed that the *P. knowlesi* isolates underwent purifying selection. Furthermore, the significant negative values of Tajima’s D as well as Fu and Li’s D and F further suggested purifying selection with population expansion of the zoonotic *P. knowlesi*, as evidenced by the abundance of *csp* haplotype sharing between humans and macaques. One possible explanation is the large areas of rapid deforestation activities in Malaysia, which have caused macaques and *Anopheles* vectors to move closer to human habitats [36]. This could be promoting the spread of *P. knowlesi* parasites.

The close interaction between these three groups is also represented by the abundance of *P. knowlesi* infections in humans, which have been reported since 2010 [6–11]. Hence, better regulations for deforestation are urgently required in Malaysia, especially for forests inhabited by *M. fascicularis* (long-tailed macaque) and *M. nemestrina* (pig-tailed macaque). Such efforts would prevent further expansion of this simian parasite in human hosts.

## Conclusions

In summary, this study has suggested that there is no distinct cluster of *P. knowlesi* isolates from different geographic regions including Malaysian Borneo and Peninsular Malaysia. The *csp* non-repeat regions of the zoonotic *P. knowlesi* isolates were found to undergo purifying selection with population expansion, which was further supported by the extensive haplotype sharing observed between humans and macaques. Nonetheless, novel mutations were observed in the C-terminal non-repeat regions of the *csp* gene, which could be beneficial for vaccine development to treat *P. knowlesi* parasites.

## Supplementary information

**Additional file 1**: Csp sequences retrieved from the GenBank database.

**Additional file 2**: Amino acid polymorphisms in the csp N-terminal and C-terminal coding sequences.

## Data Availability

The datasets analyzed in this study are available from the corresponding author on request.

## Abbreviations

PCR: Polymerase chain reaction
*csp*: Circumsporozoite protein
K: Average number of pairwise nucleotide differences
h: Number of haplotypes
Hd: Haplotype diversity
π: Nucleotide diversity
d_S_: Rate of synonymous mutation
d_N_: Rate of non-synonymous mutation
