# Supplementary material for: Genetic diversity of circumsporozoite protein in Plasmodium knowlesi isolates from Malaysian Borneo and Peninsular Malaysia
Source: Malar J. 2020 Oct 22;19:377. doi: 10.1186/s12936-020-03451-x (PMC7579551; doi:10.1186/s12936-020-03451-x)
Supplement: Supplementary file 1 — Additional file 1: Csp sequences retrieved from the GenBank database. [file 12936_2020_3451_MOESM1_ESM.docx]

**Additional file 1: *Csp* sequences retrieved from the GenBank database.** Consisting of 33 sequences from macaques in Sarawak, 24 sequences from macaques in Singapore, 62 sequences from humans in Peninsular Malaysia, 33 sequences from humans in Sarawak, 14 sequences from humans in Singapore, and 2 sequences as an outgroup.

>DQ641528 Macaque Sarawak

ATGAAGAACTTCATTCTCTTGGCCGTCTCCTCCATCCTGCTGGTGGACTTGCTCCCTACACACTTCGAACATAATGTAGATCTCTCCAGGGCCATAAATATAAATGGAGTAAGCTTCAATAATGTAGACACCAGTTCACTTGGCGCAGCACAGGTAAGACAAAGTGCTAGCCGAGGCAGAGGACTTGGTGAAAAGCCAAAAGAAGGAGCTGATAAAGAAAAAAAAAAGAAAAAAGAAGAACCAAAGAAGCCAAATGAAAATAAGCTGAAACAACCACAAGCAGAAGGTGATGGAGCAAATGCACGACAGCCACAGGCAGAAGGTGATGGAGCAAATGCACGACAACCACAAGCAGAAGGTGATGGAGCAAATGCACGACAACCACAGGCAGAAGGTGATGGAGCAAATGCACGACAACCACAGGCAGAAGGTGATGGAGCAAATGCACGACAACCACAAGCAGAAGGTGATGGAGCAAATGCACGACAACCACAGGCAGAAGGTGATGGAGCAAATGCACGACAACCACAGGCAGAAGGTGATGGAGCAAATGCACGACAACCACAAGCAGAAGGTGATGGAGCAAATGCACGACAGCCACAGGCAGAAGGTGATGGAGCAAATGCACGACAGCCACAGGCAGAAGGTGGTGGAGCAAATGCACGACAGCCACAGGCAGAAGGTGATGGAGCAAATGCACGACAACCACAAGCACAAGGAGATGGAGGAAATGCACGACAAGGAGGAAACGGGGGAGGTGCACCAGCAGGAGGAAATGAGGGGAATAAACAAGCAGGAAAAGGACAGGGACAAAACAATCAGGGTGCGAATGCCCCAAATGAAAAAGTTGTAAATGATTACCTACAGAAAATTAGATCTAGCGTTACCACCGAGTGGACTCCATGCAGTGTAACCTGTGGAAATGGTGTAAGAATTAGAAGAAGAGCTCATGCAGATAAGAAAAAGGCAGAGGACCTTACTATGGATGACCTTGAAGTGGAAGCTTGTGTAATGGATAAGTGTGCTGGCATATTTAACGTTGTGAGTAATTCATTAGGGTTAGTCATATTGTTAGTCCTAGCATTATTCAAT---------------------------------------------------------------------------------------------------------------------------------------------------------------------------------------------------------------------------------------------------------------------------------------------------------------------------------------------------------------------------------------------------------

>DQ641527 Macaque Sarawak

ATGAAGAACTTCATTCTCTTGGCCGTCTCCTCCATCCTGCTGGTGGACTTGCTCCCCACACACTTCGAACATAATGTAGATCTCTCCAGGGCCATAAATGTAAATGGAGTAAGCTTCAATAATGTAGACACCAGTTCACTTGGCGCAGCACAGGTGAGACAAAGTGCTAGCCGAGGCAGAGGACTTGGTGAGAAGCGAAAAGAAGGAGCTGATAAAGAAAAGAAAAAAGAAAAAGAAGAAGAACCAAAGAAGCCAAATGAAAATAAGCTGAAACAACCGGAACAAGCAAGACCAGGGGGAGAACAACCAGCACCAGGACCAGGGGGAGAACAACCAGCACCAGGACCAGGGGGAGAACAACCAGCACCAGGACCAGGGGGCGAACAACCAGCACCAGGACCAGGGGGCGAACAACCAGCACCAGGACCAGGGGGAGAACAACCAGCACCAGGACCAGGGGGAGAACAACCAGCACCAGGACCAGGGGGAGAACAACCAGCACCAGGACCAGGGGGCGAACAACCAGCACCAAGACCAGGGGGAGAACAACCAGCACCAGGACCAGGGGGCGAACAACCAGCACCAAGACCAGGGGGAGAACAACCAGCACCAGGACCAGGGGGCGAACAACCAGCACCAGCACCAAGGAGGGAACAACCAGCACCAGGACCAGGGGGCGAACAACCAGCACCAGGAGCAGGTGCGGGAGATGGAGCACGAGGAGGAAACGCAGGGGCAGGTAAAGGACAGGGACAAAACAATCAGGGTGCAAATGTCCCAAATGAAAAAGTTGTGAATGATTACCTACACAAAATTAGATCTAGCGTTACCACCGAGTGGACTCCATGCAGTGTAACCTGTGGAAATGGTGTAAGAATTAGAAGAAAAGCTCATGCAGATAAGAAAAAGGCAGAGAACCTTACTATGGATGACCTTGAAGTGGAAGCTTGTGTAATGGATAAGTGCGCTGGCATATTTAACGTTGTGAGTAATTCATTAGGGTTAGTCATATTGTTAGTCCTAGCATTATTCAAT------------------------------------------------------------------------------------------------------------------------------------------------------------------------------------------------------------------------------------------------------------------------------------------------------------------------------------------------------------------------------------------------------------------------------------------------------------------

>DQ641526 Macaque Sarawak

ATGAAGAACTTCATTCTCTTGGCCGTCTCCTCCATCCTGCTGGTGGACTTGCTCCCCACACACTTCGAACATAATGTAGATCTCTCCAGGGCCATAAATGTAAATGGAGTAAGCTTCAATAATGTAGACACCAGTTCACTTGGCGCAGCACAGGTGAGACAAAGTGCTAGCCGAGGCAGAGGACTTGGTGAGAAGCGAAAAGAAGGAGCTGATAAAGAAAAGAAAAAAGAAAAAGAAGAAGAACCAAAGAAGCCAAATGAAAATAAGCTGAAACAACCGGAACAAGCAAGACCAGGGGGAGAACAACCAGCACCAGGACCAGGGGGAGAACAACCAGCACCAGGACCAGGGGGAGAACAACCAGCACCAGGACCAGGGGGCGAACAACCAGCACCAGGACCAGGGGGCGAACAACCAGCACCAGGACCAGGGGGAGAACAACCAGCACCAGGACCAGGGGGAGAACAACCAGCACCAGGACCAGGGGGAGAACAACCAGCACCAGGACCAGGGGGCGAACAACCAGCACCAAGACCAGGGGGAGAACAACCAGCACCAGGACCAGGGGGCGAACAACCAGCACCAAGACCAGGGGGAGAACAACCAGCACCAGGACCAGGGGGCGAACAACCAGCACCAGCACCAAGGAGGGAACAACCAGCACCAGGACCAGGGGGCGAACAACCAGCACCAGGAGCAGGTGCGGGAGATGGAGCACGAGGAGGAAACGCAGGGGCAGGTAAAGGACAGGGACAAAACAATCAGGGTGCAAATGTCCCAAATGAAAAAGTTGTGAATGATTACCTACACAAAATTAGATCTAGCGTTACCACCGAGTGGACTCCATGCAGTGTAACCTGTGGAAATGGTGTAAGAATTAGAAGAAAAGCTCATGCAGATAAGAAAAAGGCAGAGAACCTTACTATGGATGACCTTGAAGTGGAAGCTTGTGTAATGGATAAGTGCGCTGGCATATTTAACGTTGTGAGTAATTCATTAGGGTTAGTCATATTGTTAGTCCTAGCATTATTCAAT------------------------------------------------------------------------------------------------------------------------------------------------------------------------------------------------------------------------------------------------------------------------------------------------------------------------------------------------------------------------------------------------------------------------------------------------------------------

>GU002532 Macaque Sarawak

ATGAGGAACTTCATTCTCTTGGCCGTCTCCTCCATCCTGCTGGTGGACTTGCTCCCCACACACTTCGAACATAATGTAGATCTCTCCAGGGCCATAAATGTAAATGGAGTAAGCTTCAATAATGTAGACACCAGTTCACTTGGCGCAGCACAGGTAAGACAAAGTGCTAGCCGAGGCAGAGGACTTGGTGAGAAGCGAAAAGAAGGAGCTGATAAAGAAAAGAAAAAAGAAAAAGAAGAAGAACCAAAGAAGCCAAATGAAAATAAGCTGAAACAACCGGAACAAGCAGCACCAGGAGCAGGGGGAGAACAACCAGCACCAGGAGCAGGGGGAGAACAACCAGCACCAGGAGCAGGGGGAGAACGACCAGCACCAGGAGCAGGGGGAGAACAACCAGCACCAGGAGCAGGGGGAGAACAACCAGCACCAGGAGCAGGGGGAGAACGACCAGCACCAGGAGCAGGGGGAGAACAACCAGCACCAGGAGCAGGGGGAGAACAACCAGCACCAGGAGCAGGGGGAGAACAACCAGCACCAGGAGCAGGGGGAGAACAACCAGCACCAGGAGCAGGGGGAGAACGACCAGCACCAGGAGCAGGGGGAGAACGACCAGCACCAGGAGCAGGGGGAGAACAACCAGCACCAGGAGCAGGGGGAGAACAACCAGCACCAGCACCAAGGAGGGAACAACCAGCACCAGGACCAGGTGCGGGAGATGGAGCACGAGGAGGAAACGCAGGGGCAGGTAAAGGACAGGGACAAAACAATCAGGGTGCGAATGTCCCAAATGAAAAAGTTGTGAATGATTACCTACACAAAATTAGATCTAGCGTTACCACCGAGTGGACTCCATGCAGTGTAACCTGTGGAAATGGTGTAAGAATTAGAAGAAGACAGAATGCTGGTAATAAAAAGGCAGAGGACCTTACTATGGATGACCTTGAGGTGGAAGCTTGTGTAATGGATAAGTGCGCTGGCATATTTAACGTTGTGAGTAATTCATTAGGGTTAGTCATATTGTTAGTCCTAGCATTATTCAATTAA---------------------------------------------------------------------------------------------------------------------------------------------------------------------------------------------------------------------------------------------------------------------------------------------------------------------------------------------------------------------------------------------------------------------------------------------------------

>GU002531 Macaque Sarawak

ATGAAGAACTTCATTCTCTTGGCCGTCTCCTCCATCCTGCTGGTGGACTTGCTCCCCACACACTTCGAACATAATGTAGATCTCTCCAGGGCCATAAATGTAAATGGAGTAAGCTTCAATAATGTAGACACCAGTTCACTTGGCGCAGCACAGGTAAGACAAAGTGCTAGCCGAGGCAGAGGACTTGGTGAGAAGCCAAAAGAAGGAGCTGATAAAGAAAAGAAAAAAGAAAAAGAAAAAGAAAAAGAAGAAGAACCAAAGAAGCCAAATGAAAATAAGCTGAAACAACCGGAACAACCAGCAGCAGGAGCAGGGGGCGAACAACCAGCAGCAGGAGCAGGAGGCGAACAACCAGCAGCAGGAGCAGGAGGCGAACAACCAGCAGCAGGAGCAGGAGGCGAACAACCAGCAGCAGGAGCAGGAGGCGAACAACCAGCAGCAGGAGCAGGAGGCGAACAACCAGCAGCAGGAGCAAGAGGCGAACAACCAGCAGCAGGAGCAGGAGGCGAACAACCAGCAGCAGGAGCAGGAGGCGAACAACCAGCAGCAGGAGCAAGAGGCGAACAACCAGCAGCAGGAGCAGGAGGCGAACAACCAGCAGCAGGAGCAGGAGGCGAACAACCAGCACCAGCACCAAGGAGGGAACAACCAGCAGCAGGAGCAGGGGGCGAACAACCAGCACCAGCACCAAGGAGGGAACAACCAGCACCAGGAGCAGGTGCGGGAGATGGAGCACGAGGAGGAAACGCAGGGGCAGGTAAAGGACAGGGACAAAACAATCAGGGTGCGAATGTCCCAAATGAAAAAGTTGTGAATGATTACCTACACAAAATTAGATCTAGCGTTACCACCGAGTGGACTCCATGCAGTGTAACCTGTGGAAATGGTGTAAGAATTAGAAGAAAAGGTCATGCAGGTAATAAAAAGGCAGAGGACCTTACTATGGATGACCTTGAGGTGGAAGCTTGTGTAATGGATAAGTGCGCTGGCATATTTAACGTTGTGAGTAATTCATTAGGCTTAGTCATATTGTTAGTCCTAGCATTATTCAATTAA---------------------------------------------------------------------------------------------------------------------------------------------------------------------------------------------------------------------------------------------------------------------------------------------------------------------------------------------------------------------------------------------------------------------------------------------

>GU002530 Macaque Sarawak

ATGAGGAACTTCATTCTCTTGGCCGTCTCCTCCATCCTGCTGGTGGACTTGCTCCCCACACACTTCGAACATAATGTAGATCTCTCCAGGGCCATAAATGTAAATGGAGTAAGCTTCAATAATGTAGACACCAGTTCACTTGGCGCAGCACAGGTAAGACAAAGTGCTAGCCGAGGCAGAGGACTTGGTGAGAAGCGAAAAGAAGGAGCTGATAAAGAAAAGAAAAAAGAAAAAGAAGAAGAACCAAAGAAGCCAAATGAAAATAAGCTGAAACAACCGAATGCAGAAGGTGGAGCAAATGCAGGACAACCGAATGCAGAAGGTGGAGCAAATGCAGGACAACCGAATGCAGAAGGTGGAGCAAATGCAGGACAACCTAATGCAGAAGGTGGAGCAAATGCAGGACAACCGAATGCAGAAGGTGGAGCAAATGCAGGACAACCTAATGCAGAAGGTGGAGCAAATGCAGGACAACCGAATGCAGAAGGTGGAGCAAATGCAGGACAACCGAATGCAGAAGGTGGAGCAAATGCACGACAACCGAATGCAGAAGGTGGAGCAAATGCAGGACAACCGAATGCAGAAGGTGGAGCAAATGCAGGACAACCGAATGCAGAAGGTGGAGCAAATGCAGGACAACCGAATGCAGAAGGTGGAGCAAATGCAGGACAACCGAATGCAGAAGGTGGAGCAAATGCAGGACAACCGAATGCAGAAGGTGGAGCAAATGCACGACAGCCACAGGCAGAAGGTGGTGGAGCAAATGCACGACAAGGAGGAAATGAGGGGAATAAACAAGCAGGAAAAGGACAGGGACAAAACAATCAGGGTGCGAATGCCCCAAATGAAAAAGTTGTAAATGATTACCTACAGAAAATTAGATCTAGCGTTACCACCGAGTGGACTCCATGCAGTGTAACCTGTGGAAATGGTGTAAGAATTAGAAGAAGAGCTCATGCAGATAAGAAAAAGGCAGAGGACCTTACTATGGATGACCTTGAAGTGGAAGCTTGTGTAATGGATAAGTGTGCTGGCATATTTAACGTTGTGAGTAATTCATTAGGGTTAGTCATATTGTTAGTCCTAGCATTATTCAATTAA------------------------------------------------------------------------------------------------------------------------------------------------------------------------------------------------------------------------------------------------------------------------------------------------------------------------------------------------------------------------------------------------

>GU002529 Macaque Sarawak

ATGAGGAACTTCATTCTCTTGGCCGTCTCCTCCATCCTGCTGGTGGACTTGCTCCCCACACACTTCGAACATAATGTAGATCTCTCCAGGGCCATAAATGTAAATGGAGTAAGCTTCAATAATGTAGACACCAGTTCACTTGGCGCAGCACAGGTAAGACAAAGTGCTAGCCGAGGCAGAGGACTTGGTGAGAAGCGAAAAGAAGGAGCTGATAAAGAAAAGAAAAAAGAAAAAGAAGAAGAACCAAAGAAGCCAAATGAAAATAAGCTGAAACAACCTAATGCAGAAGGTGGAGCAAATGCAGGACAACCGAATGCAGAAGGTGGAGCAAATGCAGGACAACCGAATGCAGAAGGTGGAGCAAATGCAGGACAACCTAATGCAGAAGGTGGAGCAAATGCAGGACAACCGAATGCAGAAGGTGGAGCAAATGCAGGACAACCTAATGCAGAAGGTGGAGCAAATGCAGGACAACCGAATGCAGAAGGTGGAGCAAATGCAGGACAACCGAATGCAGAAGGTGGAGCAAATGCAGGACAACCGAATGCAGAAGGTGGAGCAAATGCAGGACAACCGAATGCAGAAGGTGGAGCAAATGCAGGACAACCGAATGCAGAAGGTGGAGCAAATGCACGACAACCGAATGCAGAAGGTGGAGCAAATGCAGGACAACCGAATGCAGAAGGTGGAGCAAATGCAGGACAACCGAATGCAGAAGGTGGAGCAAATGCACGACAGCCACAGGCAGAAGGTGGTGGAGCAAATGCACGACAAGGAGGAAATGAGGGGAATAAACAAGCAGGAAAAGGACAGGGACAAAACAATCAGGGTGCGAATGCCCCAAATGAAAAAGTTGTAAATGATTACCTACAGAAAATTAGATCTAGCGTTACCACCGAGTGGACTCCATGCAGTGTAACCTGTGGAAATGGTGTAAGAATTAGAAGAAGAGCTCATGCAGATAAGAAAAAGGCAGAGGACCTTACTATGGATGACCTTGAAGTGGAAGCTTGTGTAATGGATAAGTGTGCTGGCATATTTAACGTTGTGAGTAATTCATTAGGGTTAGTCATATTGTTAGTCCTAGCATTATTCAATTAA------------------------------------------------------------------------------------------------------------------------------------------------------------------------------------------------------------------------------------------------------------------------------------------------------------------------------------------------------------------------------------------------

>GU002528 Macaque Sarawak

ATGAAGAACTTCATTCTCTTGGCCGTCTCCTCCATCCTGCTGGTGGACTTGCTCCCCACACACTTCGAACATAATGTAGATCTCTCCAGGGCCATAAATGTAAATGGAGTAAGCTTCAATAATGTAGACACCAGTTCACTTGGCGCAGCACAGGTAAGACAAAGTGCTAGCCGAGGCAGAGGACTTGGTGAGAAGCCAAAAGAAGGAGATGATAAAGAAAAGAAAAAAGAAAAAGAAAAAGAAGAAGAACCAAAGAACCTAAATGAAAATAAGCCGAAACAACCGAATGCAGAAGGTGATGGAGCTAAGCCGAAACAACCGAATGCAGAAGGTGATGGAGCTAAGCTGAAACAACCGAATGCAGAAGGTGATGGAGCTAAGCTGAAACAACCGAATGCAGAAGGTGATGGAGGAAATGCACGACAACCGAATGCAGAAGGTGATGGAGGAAATGCACGACAACCGAATGCAGAAGGTGATGGAGGAAATGCACGACAACCGAATGCAGAAGGTGATGGAGGAAATGCACGACAACCGAATGCAGAAGGTGATGGAGGAAATGCACGACAACCGAATGCAGAAGGTGATGGAGGAAATGCACGACAACCTAATGCAGAAGGTGATGGAGGAAATGCACGACAACCGAATGCAGAAGGTGATGGAGGAAATGCACGACAACCGAATGCAGAAGGTGATGGAGGAAATGCACGACAACCTAATGCAGAAGGTGATGGAGGAAATGCACGACAACCTAATGCAGAAGGTGATGGAGGAAATGCACGACAACCTAATGCAGAAGGTGATGGAGGAAATGCACGACAACCGAATGCAGAAGGTGATGGAGCAAATGCACGACAGCCACAGGCAGAAGGTGGTGGAGGAAATGCACGACAAGGAGGAAATGAGGGGAATAAACAAGCAGGAAAAGGACAGGGACAAAACAATCAGGGTGCGAATGCCCCAAATGAAAAAGTTGTAAATGATTACCTACAGAAAATTAGATCTAGCGTTACCACCGAGTGGACTCCATGCAGTGTAACCTGTGGAAATGGTGTAAGAATTAGAAGAAGAGCTCATGCAGATAAGAAAAAGGCAGAGGACCTTACTATGGATGACCTTGAAGTGGAAGCTTGTGTAATGGATAAGTGTGCTGGCATATTTAACGTTGTGAGTAATTCATTAGGGTTAGTCATATTGTTAGTCCTAGCATTATTCAATTAA------------------------------------------------------------------------------------------------------------------------------------------------------------------------------------------------------------------------------------------------------------------------

>GU002520 Macaque Sarawak

ATGAGGAACTTCATTCTCTTGGCCGTCTCCTCCATCCTGCTGGTGGACTTGCTCCCCACACACTTCGAACATAATGTAGATCTCTCCAGGGCCATAAATGTAAATGGAGTAAGCTTCAATAATGTAGACACCAGTTCACTTGGCGCAGCACAGGTAAGACAAAGTGCTAGTCGAGGCAGAGGACTTGGTGAGAAGCGAAAAGAAGGAGCTGATAAAGAAAAGAAAAAAGAAAAAGAAGAAGAACCAAAGAAGCCAAATGAAAATAAGCTGAAACAACCGGAACAAGCAGCACCAGGAGCAGGGGGAGAACAACCAGCACCAGGAGCAGGGGGAGAACGACCAGCACCAGGAGCAGGGGGAGAACAACCAGCACCAGGAGCAGGGGGAGAACAACCAGCACCAGGAGCAGGGGGAGAACAACCAGCACCAGGAGCAGGGGGAGAACAACCAGCACCAGGAGCAGGGGGAGAACGACCAGCACCAGGAGCAGGGGGAGAACAACCAGCACCAGGAGCAGGGGGAGAACAACCAGCACCAGGAGCAGGGGGAGAACAACCAGCACCAGGAGCAGGGGGAGAACGACCAGCACCAGGAGCAGGGGGAGAACAACCAGCACCAGGAGCAGGGGGAGAACGACCAGCACCAGGAGCAGGGGGAGAACAACCAGCACCAGCACCAAGGAGGGAACAACCAGCACCAGGACCAGGTGCGGGAGATGGAGCACGAGGAGGAAACGCAGGGGCAGGTAAAGGACAGGGACAAAACAATCAGGGTGCGAATGTCCCAAATGAAAAAGTTGTGAATGATTACCTACACAAAATTAGATCTAGCGTTACCACCGAGTGGACTCCATGCAGTGTAACCTGTGGAAATGGTGTAAGAATTAGAAGAAGACAGAATGCTGGTAATAAAAAGGCAGAGGACCTTACTATGGATGACCTTGAGGTGGAAGCTTGTGTAATGGATAAGTGCGCTGGCATATTTAACGTTGTGAGTAATTCATTAGGGTTAGTCATATTGTTAGTCCTAGCATTATTCAATTAA---------------------------------------------------------------------------------------------------------------------------------------------------------------------------------------------------------------------------------------------------------------------------------------------------------------------------------------------------------------------------------------------------------------------------------------------------------

>GU002518 Macaque Sarawak

ATGAAGAACTTCATTCTCTTGGCCGTCTCCTCCATCCTGCTGGTGGACTTGCTCCCCACACACTTCGAACATAATGTAGATCTCTCCAGGGCCATAAATGTAAATGGAGTAAGCTTCAATAATGTAGACACCAGTTCACTTGGCGCAGCACAGGTAAGACAAAGTGCTAGTCGAGGCAGAGGACTTGGTGAGAAGCGAAAAGAAGGAGCTGATAAAGAAAAGAAAAAAGAAAAAGAAGAAGAACCAAAGAAGCCAAATGAAAATAAGCTGAAACAACCGGAACAAGCAGCACCAGGAGCAGGGGGAGAACAACCAGCACCAGGAGCAGGGGGAGAACGACCAGCACCAGGAGCAGGGGGAGAACAACCAGCACCAGGAGCAGGGGGAGAACAACCAGCACCAGGAGCAGGGGGAGAACAACCAGCACCAGGAGCAGGGGGAGAACAACCAGCACCAGGAGCAGGGGGAGAACGACCAGCACCAGGAGCAGGGGGAGAACAACCAGCACCAGGAGCAGGGGGAGAACAACCAGCACCAGGAGCAGGGGGAGAACAACCAGCACCAGGAGCAGGGGGAGAACGACCAGCACCAGGAGCAGGGGGAGAACAACCAGCACCAGGAGCAGGGGGAGAACGACCAGCACCAGGAGCAGGGGGAGAACAACCAGCACCAGCACCAAGGAGGGAACAACCAGCACCAGGACCAGGTGCGGGAGATGGAGCACGAGGAGGAAACGCAGGGGCAGGTAAAGGACAGGGACAAAACAATCAGGGTGCGAATGTCCCAAATGAAAAAGTTGTGAATGATTACCTACACAAAATTAGATCTAGCGTTACCACCGAGTGGACTCCATGCAGTGTAACCTGTGGAAATGGTGTAAGAATTAGAAGAAGACAGAATGCTGGTAATAAAAAGGCAGAGGACCTTACTATGGATGACCTTGAGGTGGAAGCTTGTGTAATGGATAAGTGCGCTGGCATATTTAACGTTGTGAGTAATTCATTAGGGTTAGTCATATTGTTAGTCCTAGCATTATTCAATTAA---------------------------------------------------------------------------------------------------------------------------------------------------------------------------------------------------------------------------------------------------------------------------------------------------------------------------------------------------------------------------------------------------------------------------------------------------------

>GU002517 Macaque Sarawak

ATGAGGAACTTCATTCTCTTGGCCGTCTCCTCCATCCTGCTGGTGGACTTGCTCCCCACACACTTCGAACATAATGTAGATCTCTCCAGGGCCATAAATGTAAATGGAGTAAGCTTCAATAATGTAGACACCAGTTCACTTGGCGCAGCACAGGTAAGACAAAGTGCTAGCCGAGGCAGAGGACTTGGTGAGAAGCGAAAAGAAGGAGCTGATAAAGAAAAGAAAAAAGAAAAAGAAGAAGAACCAAAGAAGCCAAATGAAAATAAGCTGAAACAACCGGAACAAGCAGCACCAGGAGCAGGGGGAGAACAACCAGCACCAGGAGCAGGGGGAGAACAACCAGCACCAGGAGCAGGGGGAGAACGACCAGCACCAGGAGCAGGGGGAGAACAACCAGCACCAGGAGCAGGGGGAGAACAACCAGCACCAGGAGCAGGGGGAGAACGACCAGCACCAGGAGCAGGGGGAGAACAACCAGCACCAGGAGCAGGGGGAGAACAACCAGCACCAGGAGCAGGGGGAGAACAACCAGCACCAGGAGCAGGGGGAGAACAACCAGCACCAGGAGCAGGGGGAGAACGACCAGCACCAGGAGCAGGGGGAGAACGACCAGCACCAGGAGCAGGGGGAGAACAACCAGCACCAGGAGCAGGGGGAGAACAACCAGCACCAGCACCAAGGAGGGAACAACCAGCACCAGGACCAGGTGCGGGAGATGGAGCACGAGGAGGAAACGCAGGGGCAGGTAAAGGACAGGGACAAAACAATCAGGGTGCGAATGTCCCAAATGAAAAAGTTGTGAATGATTACCTACACAAAATTAGATCTAGCGTTACCACCGAGTGGACTCCATGCAGTGTAACCTGTGGAAATGGTGTAAGAATTAGAAGAAGACAGAATGCTGGTAATAAAAAGGCAGAGGACCTTACTATGGATGACCTTGAGGTGGAAGCTTGTGTAATGGATAAGTGCGCTGGCATATTTAACGTTGTGAGTAATTCATTAGGGTTAGTCATATTGTTAGTCCTAGCATTATTCAATTAA---------------------------------------------------------------------------------------------------------------------------------------------------------------------------------------------------------------------------------------------------------------------------------------------------------------------------------------------------------------------------------------------------------------------------------------------------------

>GU002516 Macaque Sarawak

ATGAGGAACTTCATTCTCTTGGCCGTCTCCTCCATCCTGCTGGTGGACTTGCTCCCCACACACTTCGAACATAATGTAGATCTCTCCAGGGCCATAAATGTAAATGGAGTAAGCTTCAATAATGTAGACACCAGTTCACTTGGCGCAGCACAGGTAAGACAAAGTGCTAGCCGAGGCAGAGGACTTGGTGAGAAGCGAAAAGAAGGAGCTGATAAAGAAAAGAAAAAAGAAAAAGAAGAAGAACCAAAGAAGCCAAATGAAAATAAGCTGAAACAACCGGAACAAGCAGCACCAGGAGCAGGGGGAGAACAACCAGCACCAGGAGCAGGGGGAGAACAACCAGCACCAGGAGCAGGGGGAGAACAACCAGCACCAGGAGCAGGGGGAGAACGACCAGCACCAGGAGCAGGGGGAGAACAACCAGCACCAGGAGCAGGGGGAGAACAACCAGCACCAGGAGCAGGGGGAGAACGACCAGCACCAGGAGCAGGGGGAGAACAACCAGCACCAGGAGCAGGGGGAGAACAACCAGCACCAGGAGCAGGGGGAGAACAACCAGCACCAGGAGCAGGGGGAGAACAACCAGCACCAGGAGCAGGGGGAGAACGACCAGCACCAGGAGCAGGGGGAGAACGACCAGCACCAGGAGCAGGGGGAGAACAACCAGCACCAGGAGCAGGGGGAGAACAACCAGCACCAGCACCAAGGAGGGAACAACCAGCACCAGGACCAGGTGCGGGAGATGGAGCACGAGGAGGAAACGCAGGGGCAGGTAAAGGACAGGGACAAAACAATCAGGGTGCGAATGTCCCAAATGAAAAAGTTGTGAATGATTACCTACACAAAATTAGATCTAGCGTTACCACCGAGTGGACTCCATGCAGTGTAACCTGTGGAAATGGTGTAAGAATTAGAAGAAGACAGAATGCTGGTAATAAAAAGGCAGAGGACCTTACTATGGATGACCTTGAGGTGGAAGCTTGTGTAATGGATAAGTGCGCTGGCATATTTAACGTTGTGAGTAATTCATTAGGGTTAGTCATATTGTTAGTCCTAGCATTATTCAATTAA------------------------------------------------------------------------------------------------------------------------------------------------------------------------------------------------------------------------------------------------------------------------------------------------------------------------------------------------------------------------------------------------------------------------------

>GU002515 Macaque Sarawak

ATGAGGAACTTCATTCTCTTGGCCGTCTCCTCCATCCTGCTGGTGGACTTGCTCCCCACACACTTCGAACATAATGTAGATCTCTCCAGGGCCATAAATGTAAATGGAGTAAGCTTCAATAATGTAGACACCAGTTCACTTGGCGCAGCACAGGTAAGACAAAGTGCTAGCCGAGGCAGAGGACTTGGTGAGAAGCGAAAAGAAGGAGCTGATAAAGAAAAGAAAAAAGAAAAAGAAGAAGAACCAAAGAAGCCAAATGAAAATAAGCTGAAACAACCGGAACAAGAAAGACCAGGGGGAGAACAACCAGCACCAGGACCAGGGGGCGAACAACCAGCACCAGGACCAGGGGGCGAACAACCAGCACCAAGACCAGGGGGAGAACAACCAGCACCAGGACCAGGGGGAGAACAACCAGCACCAGGACCAGGGGGAGAACAACCAGCACCAGGACCAGGGGGCGAACAACCAGCACCAAGACCAGGGGGAGAACAACCAGCACCAGGACCAGGGGGCGAACAACCAGCACCAAGACCAGGGGGAGAACAACCAGCACCAGGACCAGGGGGCGAACAACCAGCACCAAGACCAGGGGGAGAACAACCAGCACCAGGACCAGGGGGCGAACAACCAGCACCAGCACCAAGGAGGGAACAACCAGCACCAGCACCAGGAGCAGGTGCGGGAGATGGAGCACGAGGAGGAAACGCAGGGGCAGGTAAAGGACAGGGACAAAACAATCAGGGTGCGAATGTCCCAAATGAAAAAGTTGTGAATGATTACCTACACAAAATTAGATCTAGCGTTACCACCGAGTGGACTCCATGCAGTGTAACCTGTGGAAATGGTGTAAGAATTAGAAGAAGACAGAATGCTGGTAATAAAAAGGCAGAGGACCTTACTATGGATGACCTTGAGGTGGAAGCTTGTGTAATGGATAAGTGCGCTGGCATATTTAACGTTGTGAGTAATTCATTAGGGTTAGTCATATTGTTAGTCCTAGCATTATTCAATTAA------------------------------------------------------------------------------------------------------------------------------------------------------------------------------------------------------------------------------------------------------------------------------------------------------------------------------------------------------------------------------------------------------------------------------------------------------------------------------------

>GU002514 Macaque Sarawak

ATGAGGAACTTCATTCTCTTGGCCGTCTCCTCCATCCTGCTGGTGGACTTGCTCCCCACACACTTCGAACATAATGTAGATCTCTCCAGGGCCATAAATGTAAATGGAGTAAGCTTCAATAATGTAGACACCAGTTCACTTGGCGCAGCACAGGTAAGACAAAGTGCTAGCCGAGGCAGAGGACTTGGTGAGAAGCGAAAAGAAGGAGCTGATAAAGAAAAGAAAAAAGAAAAAGAAGAAGAACCAAAGAAGCCAAATGAAAATAAGCTGAAACAACCAGGGGGCGAACAACCAGCAGCAGGGGGCGAACAACCAGCAGCAGGGGGCGAACAACCAGCAGCAGGGGGTGAACAACCAGCAGCAGGGGGCGAACGACCAGCAGCAGGGGGCGAACAACCAGCAGCAGGAGGTGAACAACCAGCAGCAGGGGGCGAACAACCAGCAGCAGGGGGCGAACAACCAGCAGCAGGGGGCGAACGACCAGCAGCAGGGGGCGAACAACCAGCAGCAGGGGGTGAACAACCAGCAGCAGGGGGCGAACGACCAGCAGCAGGGGGCGAACAACCAGCAGCAGGGGGTGAACAACCAGCACCAGCACCAAGGAGGGAACAACCAGCAGCAGGGGGCGAACAACCAGCACCAGCACCAAGGAGGGAACAACCAGCACCAGGAGCAGGTGCGGGAGATGGAGCACGAGGAGGAAACCCAGGGGCAGGTAAAGGACAGGGACAAAACAATCAGGGTGCGAATGTCCCAAATGAAAAAGTTGTGAATGATTACCTACACAAAATTAGATCTAGCGTTACCACCGAGTGGACTCCATGCAGTGTAACCTGTGGAAATGGTGTAAGAATTAGAAGAAGACAGAATGCTGGTAATAAAAAGGCAGAGGACCTTACTATGGATGACCTTGAGGTGGAAGCTTGTGTAATGGATAAGTGCGCTGGCATATTTAACGTTGTGAGTAATTCATTAGGGTTAGTCATATTGTTAGTCCTAGCATTATTCAATTAA---------------------------------------------------------------------------------------------------------------------------------------------------------------------------------------------------------------------------------------------------------------------------------------------------------------------------------------------------------------------------------------------------------------------------------------------------------------------------------------

>GU002513 Macaque Sarawak

ATGAGGAACTTCATTCTCTTGGCCGTCTCCTCCATCCTGCTGGTGGACTTGCTCCCCACACACTTCGAACATAATGTAGATCTCTCCAGGGCCATAAATGTAAATGGAGTAAGCTTCAATAATGTAGACACCAGTTCACTTGGCGCAGCACAGGTAAGACAAAGTGCTAGCCGAGGCAGAGGACTTGGTGAGAAGCGAAAAGAAGGAGCTGATAAAGAAAAGAAAAAAGAAAAAGAAGAAGAACCAAAGAAGCCAAATGAAAATAAGCTGAAACAACCGGATCAAGCAGGACCAGGGGGCGAACAAGCAGGACCAGGAGGCGAACAAGCAGGACCAGGAGGCGAACAAGCAGGACCAAGACCAGGGGGCGAACAAGCAGGACCAGGAGGCGAACAAGCAGGACCAAGACCAGGGGGCGAACAAGCAGGACCAGGAGGCGAACAAGCAGGACCAAGACCAGGGGGCGAACAAGCAGGACCAGGAGGCGAACAAGCAGGACCAGGAGGCGAACAAGCAGGACCAAGACCAGGGGGCGAACAAGCAGGACCAGGGGGCGAACAAGCAGGACCAAGACCAGGGGGCGAACAAGCAGGACCAGGAGGCGAACAAGCAGGACCAAGACCAGGGGGCGAACAAGCAGGACCAGGGGGCGAACAACCAGCACCAAGACCAGGGGGAGAACAACCAGCACCAGCACCAAGGAGGGAACAACCAGCACCAGCACCAGGGGGCGAACAACCAGCACCAGGAGCAGGTGCGGGAGATGGAGCACGAGGAGGAAACCCAGGGGCAGGTAAAGGACAGGGACAAAACAATCAGGGTGCAAATGTCCCAAATGAAAAAGTTGTGAATGATTACCTACACAAAATTAGATCTAGCGTTACCACCGAGTGGACTCCATGCAGTGTAACCTGTGGAAATGGTGTAAGAATTAGAAGAAGACAGAATGCTGGTAATAAAAAGGCAGAGGACCTTACTATGGATGACCTTGAGGTGGAAGCTTGTGTAATGGATAAGTGCGCTGGCATATTTAACGTTGTGAGTAATTCATTAGGGTTAGTCATATTGTTAGTCCTAGCATTATTCAATTAA---------------------------------------------------------------------------------------------------------------------------------------------------------------------------------------------------------------------------------------------------------------------------------------------------------------------------------------------------------------------------------------------------------

>GU002512 Macaque Sarawak

ATGAGGAACTTCATTCTCTTGGCCGTCTCCTCCATCCTGCTGGTGGACTTGCTCCCCACACACTTCGAACATAATGTAGATCTCTCCAGGGCCATAAATGTAAATGGAGTAAGCTTCAATAATGTAGACACCAGTTCACTTGGCGCAGCACAGGTAAGACAAAGTGCTAGCCGAGGCAGAGGACTTGGTGAGAAGCGAAAAGAAGGAGCTGATAAAGAAAAGAAAAAAGAAAAAGAAGAAGAACCAAAGAAGCCAAATGAAAATAAGCTGAAACAACCGGAACAAGCAGCACCAGGAGCAGGGGGAGAACAACCAGCACCAGGAGCAGGGGGAGAACAACCAGCACCAGGAGCAGGGGGAGAACGACCAGCACCAGGAGCAGGGGGAGAACAACCAGCACCAGGAGCAGGGGGAGAACAACCAGCACCAGGAGCAGGGGGAGAACGACCAGCACCAGGAGCAGGGGGAGAACAACCAGCACCAGGAGCAGGGGGAGAACAACCAGCACCAGGAGCAGGGGGAGAACAACCAGCACCAGGAGCAGGGGGAGAACAACCAGCACCAGGAGCAGGGGGAGAACTACCAGCACCAGGAGCAGGGGGAGAACGACCAGCACCAGGAGCAGGGGGAGAACAACCAGCACCAGGAGCAGGGGGAGAACAACCAGCACCAGCACCAAGGAGGGAACAACCAGCACCAGGACCAGGTGCGGGAGATGGAGCACGAGGAGGAAACGCAGGGGCAGGTAAAGGACAGGGACAAAACAATCAGGGTGCGAATGTCCCAAATGAAAAAGTTGTGAATGATTACCTACACAAAATTAGATCTAGCGTTACCACCGAGTGGACTCCATGCAGTGTAACCTGTGGAAATGGTGTAAGAATTAGAAGAAGACAGAATGCTGGTAATAAAAAGGCAGAGGACCTTACTATGGATGACCTTGAGGTGGAAGCTTGTGTAATGGATAAGTGCGCTGGCATATTTAACGTCGTGAGTAATTCATTAGGGTTAGTCATATTGTTAGTCCTAGCATTATTCAATTAA---------------------------------------------------------------------------------------------------------------------------------------------------------------------------------------------------------------------------------------------------------------------------------------------------------------------------------------------------------------------------------------------------------------------------------------------------------

>GU002511 Macaque Sarawak

ATGAAGAACTTCATTCTCTTGGCCGTCTCCTCCATCCTGCTGGTGGACTTGTTCCCCACACACTTCGAACATAATGTAGATCTCTCCAGGGCCATAAATGTAAATGGAGTAAGCTTCAATAATGTAGACACCAGTTCACTTGGCGCAGCACAGGTGAGACAAAGTGCTAGCCGAGGCAGAGGACTTGGTGAGAAGCCAAAAGAAGGAGCTGATAAAGAAAAGAAAAAAGAAAAAGAAGAAAAACCAAAGAAGCCAAATGAAAATAAGCTGAAACAACCAGGGGGCGAACAACCAGCAGCAGGGGGCGAACAACCAGCAGCAGGGGGCGAACAACCAGCAGCAGGGGGTGAACAACCAGCAGCAGGGGGTGAACAACCAGCAGCAGGGGGTGAACAACCAGCAGCAGGGGGCGAACGACCAGCAGCAGGGGGCGAACAACCAGCAGCAGGGGGTGAACAACCAGCAGCAGGGGGCGAACGACCAGCAGCAGGGGGTGAACAACCAGCAGCAGGGGGCGAACGACCAGCAGCAGGGGGCGAACAACCAGCAGCAGGGGGCGAACAACCAGCAGCAGGGGGTGAACAACCAGCACCAGCACCAAGGAGGGAACAACCAGCAGCAGGGGGCGAACAACCAGCACCAGCACCAAGGAGGGAACAACCAGCACCAGGAGCAGGTGCGGGAGATGGAGCACGAGGAGGAAACGCAGGGGCAGGTAAAGGACAGGGACAAAACAATCAGGGTGCGAATGTCCCAAATGAAAAAGTTGTGAATGATTACCTACACAAAATTAGATCTAGCGTTACCACCGAGTGGACTCCATGCAGTGTAACCTGTGGAAATGGTGTAAGAATTAGAAGAAGACAGAATGCTGGTAATAAAAAGGCAGAGGACCTTACTATGGATGACCTTGAGGTGGAAGCTTGTGTAATGGATAAGTGCGCTGGCATATTTAACGTTGTGAGTAATTCATTAGGGTTAGTCATATTGTTAGTCCTAGCATTATTCAATTAA---------------------------------------------------------------------------------------------------------------------------------------------------------------------------------------------------------------------------------------------------------------------------------------------------------------------------------------------------------------------------------------------------------------------------------------------------------------------------------------

>GU002510 Macaque Sarawak

ATGAGGAACTTCATTCTCTTGGCCGTCTCCTCCATCCTGCTGGTGGACTTGCTCCCCACACACTTCGAACATAATGTAGATCTCTCCAGGGCCATAAATGTAAATGGAGTAAGCTTCAATAATGTAGACACCAGTTCACTTGGCGCAGCACAGGTAAGACAAAGTGCTAGCCGAGGCAGAGGACTTGGTGAGAAGCGAAAAGAAGGAGCTGATAAAGAAAAGAAAAAAGAAAAAGAAGAAGAACCAAAGAAGCCAAATGAAAATAAGCTGAAACAACCGAATCCAGACCAACCACAAGCACAGGGTGATGGAGCAAATGCAGGACAACCACAAGCACAAGGAGATGGAGCAAATGCAGGACAACCACAAGCACAAGGAGATGGAGCAAATGCAGGACAACCACAAGCACAGGGTGATGGAGCAAATGCAGGACAACCACAAGCACAAGGAGATGGAGCAAATGCAGGACAACCACAAGCACAGGGTGATGGAGCAAATGCAGGACAACCACAAGCACAAGGAGATGGAGCAAATGCAGGACAACCACAAGCACAGGGTGATGGAGCAAATGCAGGACAACCACAAGCACAGGGTGATGGAGCAAATGCAGGACAACCACAAGCACAGGGTGATGGAGCAAATGCAGGACAACCACAAGCACAGGGTGATAGGGGAATGCAGGACAACCACAAGCACAAGGAGATGGGGCAAATGTACCACGACAAGGAAGAAACGGGGGAGGTGCACCAGCAGGAGGAAATGAGGGGAATAAACAAGCAGGAAAAGGCCAGGGACAAAACAATCAGGGTGGAATGCCCCAAAGAAAAAGTTGTGAATGATTACCTACACAAAATTAGATCTAGCGTTACCACCGAGTGGACTCCATGCAGTGTAACCTGTGGAAATGGTGTAAGAATTAGAAGAAAAGCTCATGCAGGTAATAAAAAGGCAGAGGACCTTACTATGGATGACCTTGAGGTGGAAGCTTGTGTAATGGATAAGTGCGCTGGCATATTTAACGTTGTGAGTAATTCATTAGGGTTAGTCATATTGTTAGTCCTAGCATTATTCAATTAA------------------------------------------------------------------------------------------------------------------------------------------------------------------------------------------------------------------------------------------------------------------------------------------------------------------------------------------------------------------------------------------------------------------------

>GU002509 Macaque Sarawak

ATGAGGAACTTCATTCTCTTGGCCGTCTCCTCCATCCTGCTGGTGGACTTGCTCCCCACACACTTCGAACATAATGTAGATCTCTCCAGGGCCATAAATGTAAATGGAGTAAGCTTCAATAATGTAGACACCAGTTCACTTGGCGCAGCACAGGTAAGACAAAGTGCTAGCCGAGGCAGAGGACTTGGTGAGAAGCGAAAAGAAGGAGCTGATAAAGAAAAGAAAAAAGAAAAAGAAGAAGAACCAAAGAAGCCAAATGAAAATAAGCTGAAACAACCGAATCCAGACCAACCACAAGCACAGGGTGATGGAGCAAATGCAGGACAACCACAAGCACAAGGAGATGGAGCAAATGCAGGACAACCACAAGCACAAGGAGATGGAGCAAATGCAGGACAACCACAAGCACAGGGTGATGGAGCAAATGCAGGACAACCACAAGCACAAGGAGATGGAGCAAATGCAGGACAACCACAAGCACAGGGTGATGGAGCAAATGCAGGACAACCACAAGCACAAGGAGATGGAGCAAATGCAGGACAACCACAAGCACAGGGTGATGGAGCAAATGCAGGACAACCACAAGCACAGGGTGATGGAGCAAATGCAGGACAACCACAAGCACAGGGTGATGGAGCAAATGCAGGACAACCACAAGCACAGGGTGATAGGGCGAATGCAGGACAACCACAAGCACAAGGAGATGGGGCAAATGTACCACGACAAGGAAGAAACGGGGGAGGTGCACCAGCAGGAGGAAATGAGGGGAATAAACAAGCAGGAAAAGGACAGGGACAAAACAATCAGGGTGCGAATGCCCCAAATGAAAAAGTTGTGAATGATTACCTACACAAAATTAGATCTAGCGTTACCACCGAGTGGACTCCATGCAGTGTAACCTGTGGAAATGGTGTAAGAATTAGAAGAAAAGCTCATGCAGGTAATAAAAAGGCAGAGGACCTTACTATGGATGACCTTGAGGTGGAAGCTTGTGTAATGGATAAGTGCGCTGGCATATTTAACGTTGTGAGTAATTCATTAGGGTTAGTCATATTGTTAGTCCTAGCATTATTCAATTAA---------------------------------------------------------------------------------------------------------------------------------------------------------------------------------------------------------------------------------------------------------------------------------------------------------------------------------------------------------------------------------------------------------------------

>GU002508 Macaque Sarawak

ATGAGGAACTTCATTCTCTTGGCCGTCTCCTCCATCCTGCTGGTGGACTTGCTCCCCACACACTTCGAACATAATGTAGATCTCTCCAGGGCCATAAATGTAAATGGAGTAAGCTTCAATAATGTAGACACCAGTTCACTTGGCGCAGCACAGGTAAGACAAAGTGCTAGCCGAGGCAGAGGACTTGGTGAGAAGCGAAAAGAAGGAGCTGATAAAGAAAAGAAAAAAGAAAAAGAAGAAGAACCAAAGAAGCCAAATGAAAATAAGCTGAAACAACCGAATCCAGACCAACCACAAGCACAGGGTGATGGAGCAAATGCAGGACAACCACAAGCACAAGGAGATGGAGCAAATGCAGGACAACCACAAGCACAAGGAGATGGAGCAAATGCAGGACAACCACAAGCACAGGGTGATGGAGCAAATGCAGGACAACCACAAGCACAAGGAGATGGAGCAAATGCAGGACAACCACAAGCACAGGGTGATGGAGCAAATGCAGGACAACCACAAGCACAAGGAGATGGAGCAAATGCAGGACAACCACAAGCACAGGGTGATGGAGCAAATGCAGGACAACCACAAGCACAGGGTGATGGAGCAAATGCAGGACAACCACAAGCACAGGGTGATGGAGCAAATGCAGGACAACCACAAGCACAGGGTGATAGGGCGAATGCAGGACAACCACAAGCACAAGGAGATGGGGCAAATGTACCACGACAAGGAAGAAACGGGGGAGGTGCACCAGCAGGAGGAAATGAGGGGAATAAACAAGCAGGAAAAGGACAGGGACAAAACAATCAGGGTGCGAATGCCCCAAATGAAAAAGTTGTGAATGATTACCTACACAAAATTAGATCTAGCGTTACCACCGAGTGGACTCCATGCAGTGTAACCTGTGGAAATGGTGTAAGAATTAGAAGAAAAGCTCATGCAGGTAATAAAAAGGCAGAGGACCTTACTATGGATGACCTTGAGGTGGAAGCTTGTGTAATGGATAAGTGCGCTGGCATATTTAACGTTGTGAGTAATTCATTAGGGTTAGTCATATTGTTAGTCCTAGCATTATTCAATTAA---------------------------------------------------------------------------------------------------------------------------------------------------------------------------------------------------------------------------------------------------------------------------------------------------------------------------------------------------------------------------------------------------------------------

>GU002507 Macaque Sarawak

ATGAGGAACTTCATTCTCTTGGCCGTCTCCTCCATCCTGCTGGTGGACTTGCTCCCCACACACTTCGAACATAATGTAGATCTCTCCAGGGCCATAAATGTAAATGGAGTAAGCTTCAATAATGTAGACACCAGTTCACTTGGCGCAGCACAGGTAAGACAAAGTGCTAGCCGAGGCAGAGGACTTGGTGAGAAGCGAAAAGAAGGAGCTGATAAAGAAAAGAAAAAAGAAAAAGAAGAAGAACCAAAGAAGCCAAATGAAAATAAGCTGAAACAACCGAATCCAGACCAACCACAAGCACAGGGTGATGGAGCAAATGCAGGACAACCACAAGCACAAGGAGATGGAGCAAATGCAGGACAACCACAAGCACAAGGAGATGGAGCAAATGCAGGACAACCACAAGCACAGGGTGATGGAGCAAATGCAGGACAACCACAAGCACAAGGAGATGGAGCAAATGCAGGACAACCACAAGCACAGGGTGATGGAGCAAATGCAGGACAACCACAAGCACAAGGAGATGGAGCAAATGCAGGACAACCACAAGCACAGGGTGATGGAGCAAATGCAGGACAACCACAAGCACAGGGTGATGGAGCAAATGCAGGACAACCACAAGCACAGGGTGATGGAGCAAATGCAGGACAACCACAAGCACAGGGTGATAGGGCGAATGCAGGACAACCACAAGCACAAGGAGATGGGGCAAATGTACCACGACAAGGAAGAAACGGGGGAGGTGCACCAGCAGGAGGAAATGAGGGGAATAAACAAGCAGGAAAAGGACAGGGACAAAACAATCAGGGTGCGAATGCCCCAAATGAAAAAGTTGTGAATGATTACCTACACAAAATTAGATCTAGCGTTACCACCGAGTGGACTCCATGCAGTGTAACCTGTGGAAATGGTGTAAGAATTAGAAGAAAAGCTCATGCAGGTAATAAAAAGGCAGAGGACCTTACTATGGATGACCTTGAGGTGGAAGCTTGTGTAATGGATAAGTGCGCTGGCATATTTAACGTTGTGAGTAATTCATTAGGGTTAGTCATATTGTTAGTCCTAGCATTATTCAATTAA---------------------------------------------------------------------------------------------------------------------------------------------------------------------------------------------------------------------------------------------------------------------------------------------------------------------------------------------------------------------------------------------------------------------

>GU002506 Macaque Sarawak

ATGAAGAACTTCATTCTCTTGGCCGTCTCCTCCATCCTGCTGGTGGACTTGCTCCCCACACACTTCGAACATAATGTAGATCTCTCCAGGGCCATAAATGTAAATGGAGTAAGCTTCAATAATGTAGACACCAGTTCACTTGGCGCAGCACAGGTAAGACAAAGTGCTAGCCGAGGCAGAGGACTTGGTGAGAAGCCAAAAGAAGGAGCTGATAAAGAAAAGAAAAAAGAAAAAGAAAAAGAAAAAGAAGAAGAACCAAAGAAGCCAAATGAAAATAAGCTGAAACAACCGGAACAACCAGCAGCAGGAGCAGGGGGCGAACAACCAGCAGCAGGAGCAGGAGGCGAACAACCAGCAGCAGGAGCAGGAGGCGAACAACCAGCAGCAGGAGCAGGAGGCGAACAACCAGCAGCAGGAGCAGGAGGCGAACAACCAGCAGCAGGAGCAGGAGGCGAACAACCAGCAGCAGGAGCAGGAGGCGAACAACCAGCAGCAGGAGCAGGAGGCGAACAACCAGCAGCAGGAGCAAGAGGCGAACAACCAGCAGCAGGAGCAGGAGGCGAACAACCAGCAGCAGGAGCAGGAGGCGAACAACCAGCACCAGCACCAAGGAGGGAACAACCAGCAGCAGGAGCAGGGGGCGAACAACCAGCACCAGCACCAAGGAGGGAACAACCAGCACCAGGAGCAGGTGCGGGAGATGGAGCACGAGGAGGAAACGCAGGGGCAGGTAAAGGACAGGGACAAAACAATCAGGGTGCGAATGTCCCAAATGAAAAAGTTGTGAATGATTACCTACACAAAATTAGATCTAGCGTTACCACCGAGTGGACTCCATGCAGTGTAACCTGTGGAAATGGTGTAAGAATTAGAAGAAAAGGTCATGCAGGTAATAAAAAGGCAGAGGACCTTACTATGGATGACCTTGAGGTGGAAGCTTGTGTAATGGATAAGTGCGCTGGCATATTTAACGTTGTGAGTAATTCATTAGGCTTAGTCATATTGTTAGTCCTAGCATTATTCAATTAA------------------------------------------------------------------------------------------------------------------------------------------------------------------------------------------------------------------------------------------------------------------------------------------------------------------------------------------------------------------------------------------------------------------------------------------------------------------------

>GU002505 Macaque Sarawak

ATGAGGAACTTCATTCTCTTGGCCGTCTCCTCCATCCTGCTGGTGGACTTGCTCCCCACACACTTCGAACATAATGTAGATCTCTCCAGGGCCATAAATGTAAATGGAGTAAGCTTCAATAATGTAGACACCAGTTCACTTGGCGCAGCACAGGTAAGACAAAGTGCTAGTCGAGGCAGAGGACTTGGTGAGAAGCGAAAAGAAGGAGCTGATAAAGAAAAGAAAAAAGAAAAAGAAGAAGAACCAAAGAAGCCAAATGAAAATAAGCTGAAACAACCGGAACAAGCAGCACCAGGAGCAGGGGGAGAACAACCAGCACCAGGAGCAGGGGGAGAACGACCAGCACCAGGAGCAGGGGGAGAACAACCAGCACCAGGAGCAGGGGGAGAACAACCAGCACCAGGAGCAGGGGGAGAACAACCAGCACCAGGAGCAGGGGGAGAACAACCAGCAGCAGGGGGTGAACAACCAGCAGCAGGGGGTGAACAACCAGCACCAGCACCAAGGAGGGAACAACCAGCAGCAGGGGGTGAACAACCAGCACCAGCACCAAGGAGGGAACAACCAGCAGCAGGGGGCGAACAACCAGCACCAGGAGCAGGTGCGGGAGATGGAGCACGAGGAGGAAACGCAGGGGCAGGTAAAGGACAGGGACAAAACAATCAGGGTGCGAATGTCCCAAATGAAAAAGTTGTGAATGATTACCTACACAAAATTAGATCTAGCGTTACCACCGAGTGGACTCCATGCAGTGTAACCTGTGGAAATGGTGTAAGAATTAGAAGAAAAGCTCATGCAGATAAGAAAAAGGCAGAGAACCTTACTATGGATGACCTTGAAGTGGAAGCTTGTGTAATGGATAAGTGCGCTGGCATATTTAACGTTGTGAGTAATTCATTAGGGTTAGTCATATTGTTAGTCCTAGCATTATTCAATTAA------------------------------------------------------------------------------------------------------------------------------------------------------------------------------------------------------------------------------------------------------------------------------------------------------------------------------------------------------------------------------------------------------------------------------------------------------------------------------------------------------------------------------------------------------------------

>GU002502 Macaque Sarawak

ATGAGGAACTTCATTCTCTTGGCCGTCTCCTCCATCCTGCTGGTGGACTTGCTCCCCACACACTTCGAACATAATGTAGATCTCTCCAGGGCCATAAATGTAAATGGAGTAAGCTTCAATAATGTAGACACCAGTTCACTTGGCGCAGCACAGGTGAGACAAAGTGCTAGCCGAGGCAGAGGACTTGGTGAGAAGCGAAAAGAAGGAGCTGATAAAGAAAAGAAAAAAGAAAAAGAAGAAAAACCAAAGAAGCCAAATGAAAATAAGCTGAAACAACCAGGGGGCGAACAACCAGCAGCAGGGGGTGAACAACCAGCAGCAGGGGGCGAACAACCAGCAGCAGGGGGCGAACAACCAGCAGCAGGGGGCGAACAACCAGCAGCAGGGGGTGAACAACCAGCAGCAGGGGGTGAACAACCAGCAGCAGGGGGCGAACAACCAGCAGCAGGGGGCGAACAACCAGCAGCAGGGGGCGAACAACCAGCAGCAGGGGGCGAACAACCAGCAGCAGGGGGTGAACAACCAGCACCAGCACCAAGGAGGGAACAACCAGCAGCAGGGGGTGAACAACCAGCAGCAGGGGGTGAACAACCAGCACCAGCACCAAGGAGGGAACAACCAGCACCAGGGGGCGAACAACCAGCACCAGGAGCAGGTGCGGGAGATGGAGCACGAGGAGGAAACCCAGGGGCAGGTAAAGGACAGGGACAAAACAATCAGGGTGCGAATGTCCCAAATGAAAAAGTTGTGAATGATTACCTACACAAAATTAGATCTAGCGTTACCACCGAGTGGACTCCATGCAGTGTAACCTGTGGAAATGGTGTAAGAATTAGAAGAAAAGCTCATGCAGATAAGAAAAAGGCAGAGAACCTTACTATGGATGACCTTGAAGTGGAAGCTTGTGTAATGGATAAATGCGCTGGCATATTTAACGTTGTGAGTAATTCATTAGGGTTAGTCATATTGTTAGTCCTAGCATTATTCAATTAA------------------------------------------------------------------------------------------------------------------------------------------------------------------------------------------------------------------------------------------------------------------------------------------------------------------------------------------------------------------------------------------------------------------------------------------------------------------------------------------------------------

>GU002501 Macaque Sarawak

ATGAAGAACTTCATTCTCTTGGCCGTCTCCTCCATCCTGCTGGTGGACTTGCTCCCCACACACTTCGAACATAATGTAGATCTCTCCAGGGCCATAAATGTAAATGGAGTAAGCTTCAATAATGTAGACACCAGTTCACTTGGCGCAGCACAGGTAAGACAAAGTGCTAGCCGAGGCAGAGGACTTGGTGAGAAGCGAAAAGAAGGAGCTGATAAAGAAAAGAAAAAAGAAAAAGAAGAACCAAAGAAGCCAAATGAAAATAAGCTGAAACAACCGGAACAAGCAAGAGCAGGGGGCGAACAACCAGCAGCAGGGGGCGAACAACCAGCAGCAGGGGGCGAACAACCAGCAGCAGGGGGCGAACAACCAGCAGCAGGGGGTGAACAACCAGCAGCAGGGGGCGAACAACCAGCAGCAGGGGGCGAACAACCAGCAGCAGGGGGTGAACAACCAGCAGCAGGGGGCGAACGACCAGCAGCAGGGGGCGAACAACCAGCAGCAGGGGGCGAACAACCAGCAGCAGGGGGCGAACAACCAGCAGCAGGGGGCGAACGACCAGCAGCAGGGGGTGAACGACCAGCAGCAGGGGGTGAACAACCAGCAGCAGGGGGTGAACAACCAGCACCAGCACCAAGGAGGGAACAACCAGCAGCAGGGGGCGAACAACCAGCACCAGGAGCAGGTGCGGGAGATGGAGCACGAGGAGGAAACGCAGGGGCAGGTAAAGGACAGGGACAAAACAATCAGGGTGCGAATGTCCCAAATGAAAAAGTTGTGAATGATTACCTACACAAAATTAGATCTAGCGTTACCACCGAGTGGACTCCATGCAGTGTAACCTGTGGAAATGGTGTAAGAATTAGAAGAAAAGCTCATGCAGATAAGAAAAAGGCAGAGAACCTTACTATGGATGACCTTGAAGTGGAAGCTTGTGTAATGGATAAGTGCGCTGGCATATTTAACGTTGTGAGTAATTCATTAGGGTTAGTCATATTGTTAGTCCTAGCATTATTCAATTAA---------------------------------------------------------------------------------------------------------------------------------------------------------------------------------------------------------------------------------------------------------------------------------------------------------------------------------------------------------------------------------------------------------------------------------------------------------------------------------

>GU002500 Macaque Sarawak

ATGAAGAACTTCATTCTCTTGGCCGTCTCCTCCATCCTGCTGGTGGACTTGCTCCCCACACACTTCGAACATAATGTAGATCTCTCCAGGGCCATAAATGTAAATGGAGTAAGCTTCAATAATGTAGACACCAGTTCACTTGGCGCAGCACAGGTGAGACAAAGTGCTAGCCGAGGCAGAGGACTTGGTGAGAAGCGAAAAGAAGGAGCTGATAAAGAAAAGAAAAAAGAAAAAGAAGAAGAACCAAAGAAGCCAAATGAAAATAAGCTGAAACAACCGGAACAAGCAAGAGCAGGGGGCGAACAACCAGCAGCAGGGGGCGAACAACCAGCAGCAGGGGGCGAACAACCAGCAGCAGGGGGCGAACAACCAGCAGCAGGGGGCGAACAACCAGCAGCAGGGGGTGAACAACCAGCAGCAGGGGGCGAACAACCAGCAGCAGGGGGCGAACAACCAGCAGCAGGGGGTGAACAACCAGCAGCAGGGGGTGAACAACCAGCAGCAGGGGGTGAACAACCAGCACCAGCACCAAGGAGGGAACAACCAGCAGCAGGGGGTGAACAACCAGCACCAGCACCAAGGAGGGAACAACCAGCAGCAGGGGGCGAACAACCAGCACCAGGAGCAGGTGCGGGAGATGGAGCACGAGGAGGAAACGCAGGGGCAGGTAAAGGACAGGGACAAAACAATCAGGGTGCGAATGTCCCAAATGAAAAAGTTGTGAATGATTACCTACACAAAATTAGATCTAGCGTTACCACCGAGTGGACTCCATGCAGTGTAACCTGTGGAAATGGTGTAAGAATTAGAAGAAAAGCTCATGCAGATAAGAAAAAGGCAGAGAACCTTACTATGGATGACCTTGAAGTGGAAGCTTGTGTAATGGATAAGTGCGCTGGCATATTTAACGTTGTGAGTAATTCATTAGGGTTAGTCATATTGTTAGTCCTAGCATTATTCAATTAA---------------------------------------------------------------------------------------------------------------------------------------------------------------------------------------------------------------------------------------------------------------------------------------------------------------------------------------------------------------------------------------------------------------------------------------------------------------------------------------------------------------------------------------

>GU002499 Macaque Sarawak

ATGAAGAACTTCATTCTCTTGGCCGTCTCCTCCATCCTGCTGGTGGACTTGTTCCCCACACACTTCGAACATAATGTAGATCTCTCCAGGGCCATAAATGTAAATGGAGTAAGCTTCAATAATGTAGACACCAGTTCACTTGGCGCAGCACAGGTGAGACAAAGTGCTAGCCGAGGCAGAGGACTTGGTGAGAAGCCAAAAGAAGGAGCTGATAAAGAAAAGAAAAAAGAAAAAGAAAAAGAAAAAGAAGAAGAACCAAAGAAGCCAAATGAAAATAAGCTGAAACAACCGGAAGGAAATCAAGATGGGCGAGCACAACCGGAAGGAAATCAGGATGGTCGAGCGCAACCGGAAGGAAATCAGGATGGTCGAGCGCAACCGGAAGGAAATCAAGATGGGCGAGCACAACCGGAAGGAAATCAGGATGGTCGAGCGCAACCGGAAGGAAATCAAGATGGGCGAGCACAACCGGAAGGAAATCAGGATGGTCGAGCGCAACCGGAAGGAAATCAGGATGGGCGAGCGCAACCGGAAGGAAATCAGGATGGACGAGCACAACCGGAAGGAAATAGGGAAGCTCCAGCACAACCACAAGGAAATGGGGGGGCAGGTCAAGCACAACCGGAAGGAAATAGGGAAGCTCCAGCACAACCACAAGGAAATGGGGGGGCAGGTCAAGCACAACCGGAAGGAAATAGGGAAGCTCCAGCACAACCACAAGGAAATGGGGGGGCAGGTCAAGCACAACCACAAAAAAACGAAGGAGGAAACGCAGGAGCACGGAAAGGACAGGGACAAAACAATCAGGGTGCGAATGCCCCAAATGAAAAAGTTGTGAATGATTACCTACACAAAATTAGATCTAGCGTTACCACCGAGTGGACTCCATGCAGTGTAACCTGTGGAAATGGTGTAAGAATTAGAAGAAAAGCTCATGCAGATAAGAAAAAGGCAGAGGACCTTACTATGGATGACCTTGAAGTGGAAGCTTGTGTAATGGATAAGTGCGCTGGCATATTTAACGTTGTGAGTAATTCATTAGGGTTAGTCATATTGTTAGTCCTAGCATTATTCAATTAA---------------------------------------------------------------------------------------------------------------------------------------------------------------------------------------------------------------------------------------------------------------------------------------------------------------------------------------------------------------------------------------------------------------------

>GU002498 Macaque Sarawak

ATGAAGAACTTCATTCTCTTGGCCGTCTCCTCCATCCTGCTGGTGGACTTGCTCCCCACACACTTCGAACATAATGTAGATCTCTCCAGGGCCATAAATATAAATGGAGTAAGCTTCAATAATGTAGACACCAGTTCACTTGGCGCAGCACAGGTAAGACAAAGTGCTAGCCGAGGCAGAGGACTTGGTGAAAAGCCAAAAGAAGGAGCTGATAAAGAAAAAAAAAAAGAAAAAGAAGAACCAAAGAAGCCAAATGAAAATAAGCTGAAACAACCACAAGCAGAAGGTGATGGAGCAAATGCACGACAACCACAGGCAGAAGGTGATGGAGCAAATGCACGACAACCACAAGCAGAAGGTGATGGAGCAAATGCACGACAACCACAGGCAGAAGGTGATGGAGCAAATGCACGACAACCACAGGCAGAAGGTGATGGAGCAAATGCACGACAACCACAAGCAGAAGGTGATGGAGCAAATGCACGACAACCACAGGCAGAAGGTGATGGAGCAAATGCACGACAACCACAGGCAGAAGGTGATGGAGCAAATGCACGACAACCACAAGCAGAAGGTGATGGAGCAAATGCACGACAGCCACAGGCAGAAGGTGATGGAGCAAATGCACGACAGCCACAGGCAGAAGGTGGTGGAGCAAATGCACGACAGCCACAGGCAGAAGGTGATGGAGCAAATGCACGACAACCACAAGCACAAGGAGATGGAGGAAATGCACGACAAGGAGGAAACGGGGGAGGTGCACCAGCAGGAGGAAATGAGGGGAATAAACAAGCAGGAAAAGGACAGGGACAAAACAATCAGGGTGCGAATGCCCCAAATGAAAAAGTTGTAAATGATTACCTACAGAAAATTAGATCTAGCGTTACCACCGAGTGGACTCCATGCAGTGTAACCTGTGGAAATGGTGTAAGAATTAGAAGAAGAGCTCATGCAGATAAGAAAAAGGCAGAGGACCTTACTATGGATGACCTTGAAGTGGAAGCTTGTGTAATGGATAAGTGTGCTGGCATATTTAACGTTGTGAGTAATTCATTAGGGTTAGTCATATTGTTAGTCCTAGCATTATTCAATTAA------------------------------------------------------------------------------------------------------------------------------------------------------------------------------------------------------------------------------------------------------------------------------------------------------------------------------------------------------------------------------------------------------

>GU002497 Macaque Sarawak

ATGAGGAACTTCATTCTCTTGGCCGTCTCCTCCATCCTGCTGGTGGACTTGCTCCCCACACACTTCGAACATAATGTAGATCTCTCCAGGGCCATAAATGTAAATGGAGTAAGCTTCAATAATGTAGACACCAGTTCACTTGGCGCAGCACAGGTAAGACAAAGTGCTAGCCGAGGCAGAGGACTTGGTGAGAAGCGAAAAGAAGGAGCTGATAAAGAAAAGAAAAAAGAAAAAGAAGAAGAACCAAAGAAGCCAAATGAAAATAAGCTGAAACAACCGAATGCAGAAGGTGGAGCAAATGCAGGACAACCGAATGCAGAAGGTGGAGCAAATGCAGGACAACCGAATGCAGAAGGTGGAGCAAATGCAGGACAACCTAATGCAGAAGGTGGAGCAAATGCAGGACAACCGAATGCAGAAGGTGGAGCAAATGCAGGACAACCTAATGCAGAAGGTGGAGCAAATGCAGGACAACCGAATGCAGAAGGTGGAGCAAATGCAGGACAACCGAATGCAGAAGGTGGAGCAAATGCACGACAACCGAATGCAGAAGGTGGAGCAAATGCAGGACAACCGAATGCAGAAGGTGGAGCAAATGCAGGACAACCGAATGCAGAAGGTGGAGCAAATGCAGGACAACCGAATGCAGAAGGTGGAGCAAATGCAGGACAACCGAATGCAGAAGGTGGAGCAAATGCAGGACAACCGAATGCAGAAGGTGGAGCAAATGCACGACAGCCACAGGCAGAAGGTGGTGGAGCAAATGCACGACAAGGAGGAAATGAGGGGAATAAACAAGCAGGAAAAGGACAGGGACAAAACAATCAGGGTGCGAATGCCCCAAATGAAAAAGTTGTAAATGATTACCTACAGAAAATTAGATCTAGCGTTACCACCGAGTGGACTCCATGCAGTGTAACCTGTGGAAATGGTGTAAGAATTAGAAGAAGAGCTCATGCAGATAAGAAAAAGGCAGAGGACCTTACTATGGATGACCTTGAAGTGGAAGCTTGTGTAATGGATAAGTGTGCTGGCATATTTAACGTTGTGAGTAATTCATTAGGGTTAGTCATATTGTTAGTCCTAGCATTATTCAATTAA------------------------------------------------------------------------------------------------------------------------------------------------------------------------------------------------------------------------------------------------------------------------------------------------------------------------------------------------------------------------------------------------

>GU002496 Macaque Sarawak

ATGAGGAACTTCATTCTCTTGGCCGTCTCCTCCATCCTGCTGGTGGACTTGCTCCCCACACACTTCGAACATAATGTAGATCTCTCCAGGGCCATAAATGTAAATGGAGTAAGCTTCAATAATGTAGACACCAGTTCACTTGGCGCAGCACAGGTAAGACAAAGTGCTAGCCGAGGCAGAGGACTTGGTGAGAAGCGAAAAGAAGGAGCTGATAAAGAAAAGAAAAAAGAAAAAGAAGAAGAACCAAAGAAGCCAAATGAAAATAAGCTGAAACAACCGAATGCAGAAGGTGGAGCAAATGCAGGACAACCGAATGCAGAAGGTGGAGCAAATGCAGGACAACCGAATGCAGAAGGTGGAGCAAATGCAGGACAACCTAATGCAGAAGGTGGAGCAAATGCAGGACAACCGAATGCAGAAGGTGGAGCAAATGCAGGACAACCTAATGCAGAAGGTGGAGCAAATGCAGGACAACCGAATGCAGAAGGTGGAGCAAATGCAGGACAACCGAATGCAGAAGGTGGAGCAAATGCAGGACAACCGAATGCAGAAGGTGGAGCAAATGCAGGACAACCGAATGCAGAAGGTGGAGCAAATGCAGGACAACCGAATGCAGAAGGTGGAGCAAATGCACGACAGCCACAGGCAGAAGGTGGTGGAGCAAATGCACGACAAGGAGGAAATGAGGGGAATAAACAAGCAGGAAAAGGACAGGGACAAAACAATCAGGGTGCGAATGCCCCAAATGAAAAAGTTGTAAATGATTACCTACAGAAAATTAGATCTAGCGTTACCACCGAGTGGACTCCATGCAGTGTAACCTGTGGAAATGGTGTAAGAATTAGAAGAAGAGCTCATGCAGATAAGAAAAAGGCAGAGGACCTTACTATGGATGACCTTGAAGTGGAAGCTTGTGTAATGGATAAGTGTGCTGGCATATTTAACGTTGTGAGTAATTCATTAGGGTTAGTCATATTGTTAGTCCTAGCATTATTCAATTAA---------------------------------------------------------------------------------------------------------------------------------------------------------------------------------------------------------------------------------------------------------------------------------------------------------------------------------------------------------------------------------------------------------------------------------------------------------------------------------------------------

>GU002495 Macaque Sarawak

ATGAGGAACTTCATTCTCTTGGCCGTCTCCTCCATCCTGCTGGTGGACTTGCTCCCCACACACTTCGAACATAATGTAGATCTCTCCAGGGCCATAAATGTAAATGGAGTAAGCTTCAATAATGTAGACACCAGTTCACTTGGCGCAGCACAGGTAAGACAAAGTGCTAGCCGAGGCAGAGGACTTGGTGAGAAGCGAAAAGAAGGAGCTGATAAAGAAAAGAAAAAAGAAAAAGAAGAAGAACCAAAGAAGCCAAATGAAAATAAGCTGAAACAACCTAATGCAGAAGGTGGAGCAAATGCAGGACAACCGAATGCAGAAGGTGGAGCAAATGCAGGACAACCGAATGCAGAAGGTGGAGCAAATGCAGGACAACCTAATGCAGAAGGTGGAGCAAATGCAGGACAACCGAATGCAGAAGGTGGAGCAAATGCAGGACAACCTAATGCAGAAGGTGGAGCAAATGCAGGACAACCGAATGCAGAAGGTGGAGCAAATGCAGGACAACCGAATGCAGAAGGTGGAGCAAATGCAGGACAACCGAATGCAGAAGGTGGAGCAAATGCAGGACAACCGAATGCAGAAGGTGGAGCAAATGCAGGACAACCGAATGCAGAAGGTGGAGCAAATGCACGACAACCGAATGCAGAAGGTGGAGCAAATGCAGGACAACCGAATGCAGAAGGTGGAGCAAATGCAGGACAACCGAATGCAGAAGGTGGAGCAAATGCACGACAGCCACAGGCAGAAGGTGGTGGAGCAAATGCACGACAAGGAGGAAATGAGGGGAATAAACAAGCAGGAAAAGGACAGGGACAAAACAATCAGGGTGCGAATGCCCCAAATGAAAAAGTTGTAAATGATTACCTACAGAAAATTAGATCTAGCGTTACCACCGAGTGGACTCCATGCAGTGTAACCTGTGGAAATGGTGTAAGAATTAGAAGAAGAGCTCATGCAGATAAGAAAAAGGCAGAGGACCTTACTATGGATGACCTTGAAGTGGAAGCTTGTGTAATGGATAAGTGTGCTGGCATATTTAACGTTGTGAGTAATTCATTAGGGTTAGTCATATTGTTAGTCCTAGCATTATTCAATTAA------------------------------------------------------------------------------------------------------------------------------------------------------------------------------------------------------------------------------------------------------------------------------------------------------------------------------------------------------------------------------------------------

>GU002494 Macaque Sarawak

ATGAAGAACTTCATTCTCTTGGCCGTCTCCTCCATCCTGCTGGTGGACTTGTTCCCCACACACTTCGAACATAATGTAGATCTCTCCAGGGCCATAAATGTAAATGGAGTAAGCTTCAATAATGTAGACACCAGTTCACTTGGCGCAGCACAGGTAAGACAAAGTGCTAGCCGAGGCAGAGGACTTGGTGAGAAGCCAAAAGAAGGAGCTGATAAAGAAAAGAAAAAAGAAAAAGAAAAAGAAGAACCAAAGAAGCCAAATGAAAATAAGCTGAAACAACCGGAAGGAAATCAAGATGGGCGAGCACAACCGGAAGGAAATCAGGATGGTCGAGCGCAACCGGAAGGAAATCAGGATGGTCGAGCGCAGCCGGAAGGAAATCAAGATGGGCGAGCACAACCGGAAGGAAATCAGGATGGTCGAGCGCAACCGGAAGGAAATCAGGATGGTCGAGCGCAGCCGGAAGGAAATCAGGATGGTCGAGCGCAGCCGGAAGGAAATCAGGATGGGCGAGCGCAACCGGAAGGAAATCAGGATGGACGAGCACAACCGGAAGGAAATAGGGAAGCTCCAGCACAACCACAAGGAAATGGGGGGGCAGGTCAAGCACAACCGGAAGGAAATAGGGAAGCTCCAGCACAACCACAAGGAAATGGGGGGGCAGGTCAAGCACAACCGGAAGGAAATAGGGAAGCTCCAGCACAACCACAAGGAAATGGGGGGGCAGGTCAAGCACAACCACAAAAAAACGAAGGAGGAAACGCAGGAGCACGGAAAGGACAGGGACAAAACAATCAGGGTGCGAATGCCCCAAATGAAAAAGTTGTAAATGATTACCTACAGAAAATTAGATCTAGCGTTACCACCGAGTGGACTCCATGCAGTGTAACCTGTGGAAATGGTGTAAGAATTAGAAGAAGAGCTCATGCAGATAAGAAAAAGGCAGAGGACCTTACTATGGATGACCTTGAAGTGGAAGCTTGTGTAATGGATAAGTGTGCTGGCATATTTAACGCTGTGAGTAATTCATTAGGGTTAGTCATATTGTTAGTCCTAGCATTATTCAATTAA------------------------------------------------------------------------------------------------------------------------------------------------------------------------------------------------------------------------------------------------------------------------------------------------------------------------------------------------------------------------------------------------------------------------------

>GU002492 Macaque Sarawak

ATGAAGAACTTCATTCTCTTGGCCGTCTCCTCCATCCTGCTGGTGGACTTGCTCCCCACACACTTCGAACATAATGTAGATCTCTCCAGGGCCATAAATGTAAATGGAGTAAGCTTCAATAATGTAGACACCAGTTCACTTGGCGCAGCACAGGTAAGACAAAGTGCTAGCCGAGGCAGAGGACTTGGTGAGAAGCCAAAAGAAGGAGATGATAAAGAAAAGAAAAAAGAAAAAGAAAAAGAAGAAGAACCAAAGAACCTAAATGAAAATAAGCCGAAACAACCGAATGCAGAAGGTGATGGAGCTAAGCCGAAACAACCGAATGCAGAAGGTGATGGAGCTAAGCTGAAACAACCGAATGCAGAAGGTGATGGAGCTAAGCTGAAACAACCGAATGCAGAAGGTGATGGAGGAAATGCACGACAACCGAATGCAGAAGGTGATGGAGGAAATGCACGACAACCGAATGCAGAAGGTGATGGAGGAAATGCACGACAACCGAATGCAGAAGGTGATGGAGGAAATGCACGACAACCTAATGCAGAAGGTGATGGAGGAAATGCACGACAACCTAATGCAGAAGGTGATGGAGGAAATGCACGACAACCTAATGCAGAAGGTGATGGAGGAAATGCACGACAACCGAATGCAGAAGGTGATGGAGCAAATGCACGACAGCCACAGGCAGAAGGTGGTGGAGGAAATGCACGACAAGGAGGAAATGAGGGGAATAAACAAGCAGGAAAAGGACAGGGACAAAACAATCAGGGTGCGAATGCCCCAAATGAAAAAGTTGTAAATGATTACCTACAGAAAATTAGATCTAGCGTTACCACCGAGTGGACTCCATGCAGTGTAACCTGTGGAAATGGTGTAAGAATTAGAAGAAGAGCTCATGCAGATAAGAAAAAGGCAGAGGACCTTACTATGGATGACCTTGAAGTGGAAGCTTGTGTAATGGATAAGTGTGCTGGCATATTTAACGTTGTGAGTAATTCATTAGGGTTAGTCATATTGTTAGTCCTAGCATTATTCAATTAA------------------------------------------------------------------------------------------------------------------------------------------------------------------------------------------------------------------------------------------------------------------------------------------------------------------------------------------------------------------------------------------------------------------------------------------------------------

>M11031 Macaque Singapore

TTTATATTTAAATATATTTACACATAATTTTTCTTACTAAGTCTTTTTAACTTATCGTAAAAACAACAAAACCTTTTTTTTTGCGTGTTACATCAGTTGGAACAAGGTCTGTTCTTCTCGAATAAATCTGCATAAGGCAAATTCACGAACATCAAAAAATATACATATATATATATATTTTTTTAATCGGCTTATATTAAGCAAACGAGACAACTAGAGGCGTAGAAGTATAAGAAACCTTCCTCCACATACTTATATACAAGAACAAGATGAAGAACTTCATTCTCTTGGCCGTCTCCTCCATCCTGCTGGTGGACTTGCTCCCCACACACTTCGAACATAATGTAGATCTCTCCAGGGCAATAAATGTAAATGGAGTAAGCTTCAATAATGTAGACACCAGTTCACTTGGCGCAGCACAGGTAAGACAAAGTGCTAGCCGAGGCAGAGGACTTGGTGAGAAGCCAAAAGAAGGAGCTGATAAAGAAAAGAAAAAAGAAAAAGAAAAAGAAAAAGAAGAAGAACCAAAGAAGCCAAATGAAAATAAGCTGAAACAACCGGAACAACCAGCAGCAGGAGCAGGAGGCGAACAACCAGCAGCAGGAGCAGGAGGCGAACAACCAGCAGCAGGAGCAGGAGGCGAACAACCAGCAGCAGGAGCAAGAGGCGAACAACCAGCAGCAGGAGCAGGAGGCGAACAACCAGCAGCAGGAGCAGGAGGCGAACAACCAGCAGCAGGAGCAGGAGGCGAACAACCAGCAGCAGGAGCAGGAGGCGAACAACCAGCAGCAGGAGCAGGAGGCGAACAACCAGCAGCAGGAGCAAGAGGCGAACAACCAGCAGCAGGAGCAGGAGGCGAACAACCAGCAGCAGGAGCAGGAGGCGAACAACCAGCAGCAGGAGCAAGAGGCGAACAACCAGCAGCAGGAGCAGGAGGCGAACAACCAGCACCAGCACCAAGGAGGGAACAACCAGCACCAGGAGCAGTCGCCGGAGATGGAGCACGAGGAGGAAACGCAGGGGCAGGTAAAGGACAGGGACAAAACAATCAGGGTGCGAATGTCCCAAATGAAAAAGTTGTGAATGATTACCTACACAAAATTAGATCTAGCGTTACCACCGAGTGGACTCCATGCAGTGTAACCTGTGGAAATGGTGTAAGAATAAGAAGAAAAGGTCATGCAGGTAATAAAAAGGCAGAGGACCTTACTATGGATGACCTTGAGGTGGAAGCTTGTGTAATGGATAAGTGCGCTGGCATATTTAACGTTGTGAGTAATTCATTAGGCTTAGTCATATTGTTAGTCCTAGCATTATTCAATTAAGTAGCTGATATCCATTTTTTTCGGCGTCCCCCACGGTACATATTAAGTGTTTTGTGTTCTTTACATGCATAAAAATACTTGTCCGTAGGACATGATTTTTTCCCCTTTCTTATGAATATTTCCTGCTGTTTATATGTAACTGTATGCACATGTGTGTAAA

>KF939144 Macaque Singapore

ATGAAGAACTTCATTCTCTTGGCCGTCTCCTCCATCCTGCTGGTGGACTTGCTCCCCACATACTTCGAACATAATGTAGATCTCTCCAGGGCCATAAATGTAAATGGAGTAAGCTTCAATAGTGTAGACACCAGTTCACTTGGCGCAGCACAGGTAAGACAAAGTGCTAGCCGAGGCAGAGGACTTGGTGAGAAGCCAAAAGAAGGAGCTGATAAAGAAAAGAAAAAAGAAAAAGAAAAAGAAGAAGAACCAAAGAAGCCAAATGAAAATAAGCTGAAACAACCACAAGGAAATGGGGGGGCAGGTCAAGCACAACCAGAAGGAAATGGGGGGGCAGGTCAAGCACAACCGGAAGGAAATGGGGGGGCAGGTCAAGCACAACCAGAAGGAAATGGGGGGGCAGGTCAAGCACAACCGGAAGGAAATGGGGGGGCAGGTCAAGCACAACCGGAAGGAAATGGGGGGGCAGGTCAAGCACAACCGGAAGGAAATGGGGGGGCAGGTCAAGCACAACCGGAAGGAAATGGGGGGGCAGGTCAAGCACAACCGGAAGGAAATGGGGGGGCAGGTCAAGCACAACCGGAAGGAAATGGGGGGGCAGGTCAAGCACAACCACAAGGAAATGGGGGGGCAGGTCAAGCACAACCACAAGGAAATGGGGGGGCAGGTCAAGCACAACCACAAGGAAATGGGGGGGCAGGTCAAGCACAACCGGAAGGAAATAGGGAAGCTCCAGCACAACCACAAGGAAATGGGGGGGCAGGTCAAGCACAACCACAAAAAAACGAAGGAGGAAACGCAGGAGCACGGAAAGGACAGGGACAAAACAATCAGGGTGCGAATGCCCCAAATGAAAAAGTTGTAAATGATTACCTACAGAAAATTAGATCTAGCGTTACCACCGAGTGGACTCCATGCAGTGTAACCTGTGGAAATGGTGTAAGAATTAGAAGAAGAGCTCATGCAGATAAGAAAAAGGCAGAGGACCTTACTATGGATGACCTTGAAGTGGAAGCTTGTGTAATGGATAAGTGTGCTGGCATATTTAACGTTGTGAGTAATTCATTAGGGTTAGTCATATTGTTAGTCCTAGCATTATTCAATTAA------------------------------------------------------------------------------------------------------------------------------------------------------------------------------------------------------------------------------------------------------------------------------------------------------------------------------------------------------------------------------------------

>JQ219921 Macaque Singapore

ATGAAGAACTTCATTCTCTTGGCCGTCTCCTCCATCCTGCTGGTGGACTTGCTCCCCACACACTTCGAACATAATGTAGATCTCTCCAGGGCCATAAATGTAAATGGAGTAAGCTTCAATAATGTAGACACCAGTTCACTTGGCGCAGCACAGGTGAGACAAAGTGCTAGCCGAGGCAGAGGACTTGGTGAGAAGCCAAAAGAAGGAGCTGATAAAGAAAAGAAAAAAGAAAAAGAAAAAGAAAAAGAAGAACCAAAGAAGCCAAATGAAAATAAGCTGAAACAACCGGAACAACCAGCAGCAGGAGCAGGGGGCGAACAACCAGCAGCAGGAGCAGGAGGCGAACAACCAGCAGCAGGAGCAGGAGGCGAACAACCAGCAGCAGGAGCAGGAGGCGAACAACCAGCGGCAGGAGCAGGGGGCGAACAACCAGCAGCAGGAGCAGGAGGCGAACAACCAGCAGCAGGAGCAGGAGGCGAACAACCAGCAGCAGGAGCAGGAGGCGAACAACCAGCAGCAGGAGCAGGAGGCGAACAACCAGCAGCAGGAGCAGGGGGCGAACAACCAGCAGCAGGAGCAGGAGGCGAACAACCAGCACCAGCACCAAGGAGGGAACAACCAGCAGCAGGAGCAGGGGGCGAACAACCAGCACCAGCACCAAGGAGGGAACAACCAGCACCAGGAGCAGGTGCGGGAGATGGAGCACGAGGAGGAAACGCAGGGGCAGGTAAAGGACAGGGACAAAACAATCAGGGTGCGAATGTCCCAAATGAAAAAGTTGTGAATGATTACCTACACAAAATTAGATCTAGCGTTACCACCGAGTGGACTCCATGCAGTGTAACCTGTGGAAATGGTGTAAGAATTAGAAGAAGACAGAATGCTGGTAATAAAAAGGCAGAGGACCTTACTATGGATGACCTTGAGGTGGAAGCTTGTGTAATGGATAAGTGCGCTGGCATATTTAACGTTGTGAGTAATTCATTAGGCTTAGTCATATTGTTAGTCTTAGCATTATTCAATTAA---------------------------------------------------------------------------------------------------------------------------------------------------------------------------------------------------------------------------------------------------------------------------------------------------------------------------------------------------------------------------------------------------------------------------------------------------------------------------

>JQ219920 Macaque Singapore

ATGAAGAACTTCATTCTCTTGGCCGTCTCCTCCATCCTGCTGGTGGACTTGCTCCCCACACACTTCGAACATAATGTAGATCTCTCCAGGGCCATAAATGTAAATGGAGTAAGCTTCAATAATGTAGACACCAGTTCACTTGGCGCAGCACAGGTGAGACAAAGTGCTAGCCGAGGCAGAGGACTTGGTGAGAAGCCAAAAGAAGGAGCTGATAAAGAAAAGAAAAAAGAAAAAGAAAAAGAAAAAGAAGAACCAAAGAAGCCAAATGAAAATAAGCTGAAACAACCGGAACAACCAGCAGCAGGAGCAGGGGGCGAACAACCAGCAGCAGGAGCAGGAGGCGAACAACCAGCAGCAGGAGCAGGAGGCGAACAACCAGCAGCAGGAGCAGGAGGCGAACAACCAGCGGCAGGAGCAGGGGGCGAACAACCAGCAGCAGGAGCAGGAGGCGAACAACCAGCAGCAGGAGCAGGAGGCGAACAACCAGCAGCAGGAGCAGGAGGCGAACAACCAGCAGCAGGAGCAGGAGGCGAACAACCAGCAGCAGGAGCAGGGGGCGAACAACCAGCAGCAGGAGCAGGAGGCGAACAACCAGCACCAGCACCAAGGAGGGAACAACCAGCAGCAGGAGCAGGGGGCGAACAACCAGCACCAGCACCAAGGAGGGAACAACCAGCACCAGGAGCAGGTGCGGGAGATGGAGCACGAGGAGGAAACGCAGGGGCAGGTAAAGGACAGGGACAAAACAATCAGGGTGCGAATGTCCCAAATGAAAAAGTTGTGAATGATTACCTACACAAAATTAGATCTAGCGTTACCACCGAGTGGACTCCATGCAGTGTAACCTGTGGAAATGGTGTAAGAATTAGAAGAAGACAGAATGCTGGTAATAAAAAGGCAGAGGACCTTACTATGGATGACCTTGAGGTGGAAGCTTGTGTAATGGATAAGTGCGCTGGCATATTTAACGTTGTGAGTAATTCATTAGGCTTAGTCATATTGTTAGTCTTAGCATTATTCAATTAA---------------------------------------------------------------------------------------------------------------------------------------------------------------------------------------------------------------------------------------------------------------------------------------------------------------------------------------------------------------------------------------------------------------------------------------------------------------------------

>JQ219919 Macaque Singapore

ATGAAGAACTTCATTCTCTTGGCCGTCTCCTCCATCCTGCTGGTGGACTTGCTCCCCACACACTTCGAACATAATGTAGATCTCTCCAGGGCCATAAATGTAAATGGAGTAAGCTTCAATAATGTAGACACCAGTTCACTTGGCGCAGCACAGGTGAGACAAAGTGCTAGCCGAGGCAGAGGACTTGGTGAGAAGCCAAAAGAAGGAGCTGATAAAGAAAAGAAAAAAGAAAAAGAAAAAGAAAAAGAAGAACCAAAGAAGCCAAATGAAAATAAGCTGAAACAACCGGAACAACCAGCAGCAGGAGCAGGGGGCGAACAACCAGCAGCAGGAGCAGGAGGCGAACAACCAGCAGCAGGAGCAGGAGGCGAACAACCAGCAGCAGGAGCAGGAGGCGAACAACCAGCGGCAGGAGCAGGGGGCGAACAACCAGCAGCAGGAGCAGGAGGCGAACAACCAGCAGCAGGAGCAGGAGGCGAACAACCAGCAGCAGGAGCAGGAGGCGAACAACCAGCAGCAGGAGCAGGAGGCGAACAACCAGCAGCAGGAGCAGGGGGCGAACAACCAGCAGCAGGAGCAGGAGGCGAACAACCAGCACCAGCACCAAGGAGGGAACAACCAGCAGCAGGAGCAGGGGGCGAACAACCAGCACCAGCACCAAGGAGGGAACAACCAGCACCAGGAGCAGGTGCGGGAGATGGAGCACGAGGAGGAAACGCAGGGGCAGGTAAAGGACAGGGACAAAACAATCAGGGTGCGAATGTCCCAAATGAAAAAGTTGTGAATGATTACCTACACAAAATTAGATCTAGCGTTACCACCGAGTGGACTCCATGCAGTGTAACCTGTGGAAATGGTGTAAGAATTAGAAGAAGACAGAATGCTGGTAATAAAAAGGCAGAGGACCTTACTATGGATGACCTTGAGGTGGAAGCTTGTGTAATGGATAAGTGCGCTGGCATATTTAACGTTGTGAGTAATTCATTAGGCTTAGTCATATTGTTAGTCTTAGCATTATTCAATTAA---------------------------------------------------------------------------------------------------------------------------------------------------------------------------------------------------------------------------------------------------------------------------------------------------------------------------------------------------------------------------------------------------------------------------------------------------------------------------

>JQ219918 Macaque Singapore

ATGAAGAACTTCATTCTCTTGGCCGTCTCCTCCATCCTGCTGGTGGACTTGCTCCCCACACACTTCGAACACAATGTAGATCTCTCCAGGGCCATAAATGTAAATGGAGTAAGCTTCAATAATGTAGACACCAGTTCACTTGGCGCAGCACAGGTGAGACAAAGTGCTAGCCGAGGCAGAGGACTTGGTGAGAAGCCAAAAGAAGGAGCTGATAAAGAAAAGAAAAAAGAAAAAGAAGAAGAACCAAAGAAGCCAAATGAAAATAAGCTGAAACAACCGGATGCAGTACCAGGGGGCGAAGAACCAGCACCAGGAAGGGAACAGCCAGCACCAGGAAGGGAGGAACCAGCACCAGGAAGGGAACAGCCAGCACCAGGAAGGGAAGAACCAGCACCAGGAAGGGAACAGCCAGCACCAGGAAGGGAAGAACCAGCGCCAGGAAGGGAACAGCCAGCACCAGGAAGGGAACAGCCAGCACCAGGAAGGGAGGAACCAGCACCAGGAAGGGAACAACCAGCACCAGGAAGGGAGGAACCAGCACCAGGAAGGGAACAACCAGCACCAGGAAGGGAACAGCCAGCACCAGGAAGGGAACAGCCAGCACCGGGGGGTGAACAACCAGCACCAGGAAGGGAACAGCCAGCACCGGGTGGTGAACAACCAGCACCAGCACCAGGAGCAGGTGCGGGAGATGGAGCACGAGGAGGAAACGCAGGGGCAGGGAAAGGACAGGGACAAAACAATCAGGGTGCAAATGTCCCAAATGAAAAAGTTGTGAATGATTACCTACACAAAATTAGATCTAGCGTTACCACCGAGTGGACTCCATGCAGTGTAACCTGTGGAAATGGTGTAAGAATTAGAAGAAAAGGTCATGCAGGTAATAAAAAGGCAGAGGACCTTACTATGGATGACCTTGAGGTGGAAGCTTGTGTAATGGATAAGTGCGCTGGCATATTTAACGTTGTGAGTAATTCATTAGGCTTAGTCATATTGTTAGTCCTAGCATTATTCAATTAA---------------------------------------------------------------------------------------------------------------------------------------------------------------------------------------------------------------------------------------------------------------------------------------------------------------------------------------------------------------------------------------------------------------------------------------------------------------------------------

>JQ219916 Macaque Singapore

ATGAAGAACTTCATTCTCTTGGCCGTCTCCTCCATCCTGCTGGTGGACTTGCTCCCCACACACTTCGAACATAATGTAGATCTCTCCAGGGCCATAAATGTAAATGGAGTAAGCTTCAATAATGTAGACACCAGTTCACTTGGCGCAGCACGGGTGAGACAAAGTGCTAGCCGAGGCAGAGGACTTGGTGAGAAGCCAAAAGAAGGAGCTGATAAAGAAAAGAAAAAAGAAAAAGAAAAAGAAAAAGAAGAACCAAAGAAGCCAAATGAAAATAAGCTGAAACAACCGGAACAACCAGCAGCAGGAGCAGGGGGCGAACAACCAGCAGCAGGAGCAGGAGGCGAACAACCAGCAGCAGGAGCAGGAGGCGAACAACCAGCAGCAGGAGCAGGAGGCGAACAACCAGCGGCAGGAGCAGGGGGCGAACAACCAGCAGCAGGAGCAGGAGGCGAACAACCAGCAGCAGGAGCAGGAGGCGAACAACCAGCAGCAGGAGCAGGAGGCGAACAACCAGCAGCAGGAGCAGGAGGCGAACAACCAGCAGCAGGAGCAGGGGGCGAACAACCAGCAGCAGGAGCAGGAGGCGAACAACCAGCACCAGCACCAAGGAGGGAACAACCAGCAGCAGGAGCAGGGGGCGAACAACCAGCACCAGCACCAAGGAGGGAACAACCAGCACCAGGAGCAGGTGCGGGAGATGGAGCACGAGGAGGAAACGCAGGGGCAGGTAAAGGACAGGGACAAAACAATCAGGGTGCGAATGTCCCAAATGAAAAAGTTGTGAATGATTACCTACACAAAATTAGATCTAGCGTTACCACCGAGTGGACTCCATGCAGTGTAACCTGTGGAAATGGTGTAAGAATTAGAAGAAGACAGAATGCTGGTAATAAAAAGGCAGAGGACCTTACTATGGACGACCTTGAGGTGGAAGCTTGTGTAATGGATAAGTGCGCTGGCATATTTAACGTTGTGAGTAATTCATTAGGCTTAGTCATATTGTTAGTCTTAGCATTATTCAATTAA---------------------------------------------------------------------------------------------------------------------------------------------------------------------------------------------------------------------------------------------------------------------------------------------------------------------------------------------------------------------------------------------------------------------------------------------------------------------------

>JQ219915 Macaque Singapore

ATGAAGAACTTCATTCTCTTGGCCGTCTCCTCCATCCTGCTGGTGGACTTGCTCCCCACACACTTCGAACATAATGTAGATCTCTCCAGGGCCATAAATGTAAATGGAGTAAGCTTCAATAATGTAGACACCAGTTCACTTGGCGCAGCACAGGTGAGACAAAGTGCTAGCCGAGGCAGAGGACTTGGTGAGAAGCCAAAAGAAGGAGCTGATAAAGAAAAGAAAAAAGAAAAAGAAGAAGAACCAAAGAAGCCAAATGAAAATAAGCTGAAACAACCGGATGCAGTACCAGGGGGCGAAGAACCAGCACCAGGAAGGGAACAGCCAGCACCAGGAAGGGAGGAACCAGCACCAGGAAGGGAACAGCCAGCACCAGGAAGGGAAGAACCAGCACCAGGAAGGGAACAGCCAGCACCAGGAAGGGAAGAACCAGCGCCAGGAAGGGAACAGCCAGCACCAGGAAGGGAACAGCCAGCACCAGGAAGGGAGGAACCAGCACCAGGAAGGGAACAACCAGCACCAGGAAGGGAGGAACCAGCACCAGGAAGGGAACAACCAGCACCAGGAAGGGAACAGCCAGCACCAGGAAGGGAACAGCCAGCACCGGGGGGTGAACAACCAGCACCAGGAAGGGAACAGCCAGCACCGGGTGGTGAACAACCAGCACCAGCACCAGGAGCAGGTGCGGGAGATGGAGCACGAGGAGGAAACGCAGGGGCAGGGAAAGGACAGGGACAAAACAATCAGGGTGCAAATGTCCCGAATGAAAAAGTTGTGAATGATTACCTACACAAAATTAGATCTAGCGTTACCACCGAGTGGACTCCATGCAGTGTAACCTGTGGAAATGGTGTAAGAATTAGAAGAAAAGGTCATGCAGGTAATAAAAAGGCAGAGGACCTTACTATGGATGACCTTGAGGTGGAAGCTTGTGTAATGGATAAGTGCGCTGGCATATTTAACGTTGTGAGTAATTCATTAGGCTTAGTCATATTGTTAGTCCTAGCATTATTCAATTAA---------------------------------------------------------------------------------------------------------------------------------------------------------------------------------------------------------------------------------------------------------------------------------------------------------------------------------------------------------------------------------------------------------------------------------------------------------------------------------

>JQ219914 Macaque Singapore

ATGAAGAACTTCATTCTCTTGGCCGTCTCCTCCATCCTGCTGGTGGACTTGCTCCCCACACACTTCGAACATAATGTAGATCTCTCCAGGGCCATAAATGTAAATGGAGTAAGCTTCAATAATGTAGACACCAGTTCACTTGGCGCAGCACAGGTGAGACAAAGTGCTAGCCGAGGCAGAGGACTTGGTGAGAAGCCAAAAGAAGGAGCTGATAAAGAAAAGAAAAAAGAAAAAGAAGAAGAACCAAAGAAGCCAAATGAAAATAAGCTGAAACAACCGGATGCAGTACCAGGGGGCGAAGAACCAGCACCAGGAAGGGAACAGCCAGCACCAGGAAGGGAGGAACCAGCACCAGGAAGGGAACAGCCAGCACCAGGAAGGGAAGAACCAGCACCAGGAAGGGAACAGCCAGCACCAGGAAGGGAAGAACCAGCGCCAGGAAGGGAACAGCCAGCACCAGGAAGGGAACAGCCAGCACCAGGAAGGGAGGAGCCAGCACCAGGAAGGGAACAACCAGCACCAGGAAGGGAGGAACCAGCACCAGGAAGGGAACAACCAGCACCAGGAAGGGAACAGCCAGCACCAGGAAGGGAACAGCCAGCACCGGGGGGTGAACAACCAGCACCAGGAAGGGAACAGCCAGCACCGGGTGGTGAACAACCAGCACCAGCACCAGGAGCAGGTGCGGGAGATGGAGCACGAGGAGGAAACGCAGGGGCAGGGAAAGGACAGGGACAAAACAATCAGGGTGCAAATGTCCCAAATGAAAAAGTTGTGAATGATTACCTACACAAAATTAGATCTAGCGTTACCACCGAGTGGACTCCATGCAGTGTAACCTGTGGAAATGGTGTAAGAATTAGAAGAAAAGGTCATGCAGGTAATAAAAAGGCAGAGGACCTTACTATGGATGACCTTGAGGTGGAAGCTTGTGTAATGGATAAGTGCGCTGGCATATTTAACGTTGTGAGTAATTCATTAGGCTTAGTCATATTGTTAGTCCTAGCATTATTCAATTAA---------------------------------------------------------------------------------------------------------------------------------------------------------------------------------------------------------------------------------------------------------------------------------------------------------------------------------------------------------------------------------------------------------------------------------------------------------------------------------

>JQ219913 Macaque Singapore

ATGAAGAACTTCATTCTCTTGGCCGTCTCCTCCATCCTGCTGGTGGACTTGCTCCCCACACACTTCGAACATAATGTAGATCTCTCCAGGGCCATAAATGTAAATGGAGTAAGCTTCAATAATGTAGACACCAGTTCACTTGGCGCAGCACAGGTGAGACAAAGTGCTAGCCGAGGCAGAGGACTTGGTGAGAAGCCAAAAGAAGGAGCTGATAAAGAAAAGAAAAAAGAAAAAGAAGAAGAACCAAAGAAGCCAAATGAAAATAAGCTGAAACAACCGGATGCAGTACCAGGGGGCGAAGAACCAGCACCAGGAAGGGAACAGCCAGCACCAGGAAGGGAGGAACCAGCACCAGGAAGGGAACAGCCAGCACCAGGAAGGGAAGAACCAGCACCAGGAAGGGAACAGCCAGCACCAGGAAGGGAAGAACCAGCGCCAGGAAGGGAACAGCCAGCACCAGGAAGGGAACAGCCAGCACCAGGAAGGGAGGAACCAGCACCAGGAAGGGAACAACCAGCACCAGGAAGGGAGGAACCAGCACCAGGAAGGGAACAACCAGCACCAGGAAGGGAACAGCCAGCACCAGGAAGGGAACAGCCAGCACCGGGGGGTGAACAACCAGCACCAGGAAGGGAACAGCCAGCACCGGGTGGTGAACAACCAGCACCAGCACCAGGAGCAGGTGCGGGAGATGGAGCACGAGGAGGAAACGCAGGGGCAGGGAAAGGACAGGGACAAAACAATCAGGGTGCAAATGTCCCAAATGAAAAAGTTGTGAATGATTACCTACACAAAATTAGATCTAGCGTTACCACCGAGTGGACTCCATGCAGTGTAACCTGTGGAAATGGTGTAAGAATTAGAAGAAAAGGTCATGCAGGTAATAAAAAGGCAGAGGACCTTACTATGGATGACCTTGAGGTGGAAGCTTGTGTAATGGATAAGTGCGCTGGCATATTTAACGTTGTGAGTAATTCATTAGGCTTAGTCATATTGTTAGTCCTAGCATTATTCAATTAA---------------------------------------------------------------------------------------------------------------------------------------------------------------------------------------------------------------------------------------------------------------------------------------------------------------------------------------------------------------------------------------------------------------------------------------------------------------------------------

>JQ219912 Macaque Singapore

ATGAAGAACTTCATTCTCTTGGCCGTCTCCTCCATCCTGCTGGTGGACTTGCTCCCCACACACTTCGAACATAATGTAGATCTCTCCAGGGCCATAAATGTAAATGGAGTAAGCTTCAATAATGTAGACACCAGTTCACTTGGCGCAGCACAGGTGAGACAAAGTGCTAGCCGAGGCAGAGGACTTGGTGAGAAGCCAAAAGAAGGAGCTGATAAAGAAAAGAAAAAAGAAAAAGAAGAAGAACCAAAGAAGCCAAATGAAAATAAGCTGAAACAACCGGATGCAGTACCAGGGGGCGAAGAACCAGCACCAGGAAGGGAACAGCCAGCACCAGGAAGGGAGGAACCAGCACCAGGAAGGGAACAGCCAGCACCAGGAAGGGAAGAACCAGCACCAGGAAGGGAACAGCCAGCACCAGGAAGGGAAGAACCAGCGCCAGGAAGGGAACAGCCAGCACCAGGAAGGGAACAGCCAGCACCAGGAAGGGAGGAACCAGCACCAGGAAGGGAACAACCAGCACCAGGAAGGGAGGAACCAGCACCAGGAAGGGAACAACCAGCACCAGGAAGGGAACAGCCAGCACCAGGAAGGGAACAGCCAGCACCGGGGGGTGAACAACCAGCACCAGGAAGGGAACAGCCAGCACCGGGTGGTGAACAACCAGCACCAGCACCAGGAGCAGGTGCGGGAGATGGAGCACGAGGAGGAAACGCAGGGGCAGGGAAAGGACAGGGACAAAACAATCAGGGTGCAAATGTCCCAAATGAAAAAGTTGTGAATGATTACCTACACAAAATTAGATCTAGCGTTACCACCGAGTGGACTCCATGCAGTGTAACCTGTGGAAATGGTGTAAGAATTAGAAGAAAAGGTCATGCAGGTAATAAAAAGGCAGAGGACCTTACTATGGATGACCTTGAGGTGGAAGCTTGTGTAATGGATAAGTGCGCTGGCATATTTAACGTTGTGAGTAATTCATTAGGCTTAGTCATATTGTTAGTCCTAGCATTATTCAATTAA---------------------------------------------------------------------------------------------------------------------------------------------------------------------------------------------------------------------------------------------------------------------------------------------------------------------------------------------------------------------------------------------------------------------------------------------------------------------------------

>JQ219911 Macaque Singapore

ATGAAGAACTTCATTCTCTTGGCCGTCTCCTCCATCCTGCTGGTGGACTTGCTCCCCACACACTTCGAACATAATGTAGATCTCTCCAGGGCCATAAATGTAAATGGAGTAAGCTTCAATAATGCAGACACCAGTTCACTTGGCGCAGCACAGGTGAGACAAAGTGCTAGCCGAGGCAGAGGACTTGGTGAGAAGCCAAAAGAAGGAGCTGATAAAGAAAAGAAAAAAGAAAAAGAAGAAGAACCAAAGAAGCCAAATGAAAATAAGCTGAAACAACCGGATGCAGTACCAGGGGGCGAAGAACCAGCACCAGGAAGGGAACAGCCAGCACCAGGAAGGGAGGAACCAGCACCAGGAAGGGAACAGCCAGCACCAGGAAGGGAAGAACCAGCACCAGGAAGGGAACAGCCAGCACCAGGAAGGGAAGAACCAGCGCCAGGAAGGGAACAGCCAGCACCAGGAAGGGAACAGCCAGCACCAGGAAGGGAGGAACCAGCACCAGGAAGGGAACAACCAGCACCAGGAAGGGAGGAACCAGCACCAGGAAGGGAACAACCAGCACCAGGAAGGGAGCAGCCAGCACCAGGAAGGGAACAGCCAGCACCGGGGGGTGAACAACCAGCACCAGGAAGGGAACAGCCAGCACCGGGTGGTGAACAACCAGCACCAGCACCAGGAGCAGGTGCGGGAGATGGAGCACGAGGAGGAAACGCAGGGGCAGGGAAAGGACAGGGACAAAACAATCAGGGTGCAAATGTCCCAAATGAAAAAGTTGTGAATGATTACCTACACAAAATTAGATCTAGCGTTACCACCGAGTGGACTCCATGCAGTGTAACCTGTGGAAATGGTGTAAGAATTAGAAGAAAAGGTCATGCAGGTAATAAAAAGGCAGAGGACCTTACTATGGATGACCTTGAGGTGGAAGCTTGTGTAATGGATAAGTGCGCTGGCATATTTAACGTTGTGAGTAATTCATTAGGCTTAGTCATATTGTTAGTCCTAGCATTATTCAATTAA---------------------------------------------------------------------------------------------------------------------------------------------------------------------------------------------------------------------------------------------------------------------------------------------------------------------------------------------------------------------------------------------------------------------------------------------------------------------------------

>JQ219910 Macaque Singapore

ATGAAGAACTTCATTCTCTTGGCCGTCTCCTCCATCCTGCTGGTGGACTTGCTCCCCACATACTTCGAACATAATGTAGATCTCTCCAGGGCCATAAATGTAAATGGAGTAAGCTTCAATAGTGTAGACACCAGTTCACTTGGCGCAGCACAGGTAAGACAAAGTGCTAGCCGAGGCAGAGGACTTGGTGAGAAGCCAAAAGAAGGAGCTGATAAAGAAAAGAAAAAAGAAAAAGAAAAAGAAGAAGAACCAAAGAAGCCAAATGAAAATAAGCTGAAACAACCACAAGGAAATGGGGGGGCAGGTCAAGCACAACCAGAAGGAAATGGGGGGGCAGGTCAAGCACAACCGGAAGGAAATGGGGGGGCAGGTCAAGCACAACCAGAAGGAAATGGGGGGGCAGGTCAAGCACAACCGGAAGGAAATGGGGGGGCAGGTCAAGCACAACCGGAAGGAAATGGGGGGGCAGGTCAAGCACAACCGGAAGGAAATGGGGGGGCAGGTCAAGCACAACCGGAAGGAAATGGGGGGGCAGGTCAAGCACAACCGGAAGGAAATGGGGGGGCAGGTCAAGCACAACCGGAAGGAAATGGGGGGGCAGGTCAAGCACAACCACAAGGAAATGGGGGGGCAGGTCAAGCACAACCACAAGGAAATGGGGGGGCAGGTCAAGCACAACCACAAGGAAATGGGGGGGCAGGTCAAGCACAACCGGAAGGAAATAGGGAAGCTCCAGCACAACCACAAGGAAATGGGGGGGCAGGTCAAGCACAACCACAAAAAAACGAAGGAGGAAACGCAGGAGCACGGAAAGGACAGGGACAAAACAATCAGGGTGCGAATGCCCCAAATGAAAAAGTTGTAAATGATTACCTACAGAAAATTAGATCTAGCGTTACCACCGAGTGGACTCCATGCAGTGTAACCTGTGGAAATGGTGTAAGAATTAGAAGAAGAGCTCATGCAGATAAGAAAAAGGCAGAGGACCTTACTATGGATGACCTTGAAGTGGAAGCTTGTGTAATGGATAAGTGTGCTGGCATATTTAACGTTGTGAGTAATTCATTAGGGTTAGTCATATTGTTAGTCCTAGCATTATTCAATTAA------------------------------------------------------------------------------------------------------------------------------------------------------------------------------------------------------------------------------------------------------------------------------------------------------------------------------------------------------------------------------------------

>JQ219909 Macaque Singapore

ATGAAGAACTTCATTCTCTTGGCCGTCTCCTCCATCCTGCTGGTGGACTTGCTCCCCACATACTTCGAACATAATGTAGATCTCTCCAGGGCCATAAATGTAAATGGAGTAAGCTTCAATAGTGTAGACACCAGTTCACTTGGCGCAGCACAGGTAAGACAAAGTGCTAGCCGAGGCAGAGGACTTGGTGAGAAGCCAAAAGAAGGAGCTGATAAAGAAAAGAAAAAAGAAAAAGAAAAAGAAGAAGAACCAAAGAAGCCAAATGAAAATAAGCTGAAACAACCACAAGGAAATGGGGGGGCAGGTCAAGCACAACCAGAAGGAAATGGGGGGGCAGGTCAAGCACAACCGGAAGGAAATGGGGGGGCAGGTCAAGCACAACCAGAAGGAAATGGGGGGGCAGGTCAAGCACAACCGGAAGGAAATGGGGGGGCAGGTCAAGCACAACCGGAAGGAAATGGGGGGGCAGGTCAAGCACAACCGGAAGGAAATGGGGGGGCAGGTCAAGCACAACCGGAAGGAAATGGGGGGGCAGGTCAAGCACAACCGGAAGGAAATGGGGGGGCAGGTCAAGCACAACCGGAAGGAAATGGGGGGGCAGGTCAAGCACAACCACAAGGAAATGGGGGGGCAGGTCAAGCACAACCACAAGGAAATGGGGGGGCAGGTCAAGCACAACCACAAGGAAATGGGGGGGCAGGTCAAGCACAACCGGAAGGAAATAGGGAAGCTCCAGCACAACCACAAGGAAATGGGGGGGCAGGTCAAGCACAACCACAAAAAAACGAAGGAGGAAACGCAGGAGCACGGAAAGGACAGGGACAAAACAATCAGGGTGCGAATGCCCCAAATGAAAAAGTTGTAAATGATTACCTACAGAAAATTAGATCTAGCGTTACCACCGAGTGGACTCCATGCAGTGTAACCTGTGGAAATGGTGTAAGAATTAGAAGAAGAGCTCATGCAGATAAGAAAAAGGCAGAGGACCTTACTATGGATGACCTTGAAGTGGAAGCTTGTGTAATGGATAAGTGTGCTGGCATATTTAACGTTGTGAGTAATTCATTAGGGTTAGTCATATTGTTAGTCCTAGCATTATTCAATTAA------------------------------------------------------------------------------------------------------------------------------------------------------------------------------------------------------------------------------------------------------------------------------------------------------------------------------------------------------------------------------------------

>JQ219908 Macaque Singapore

ATGAAGAACTTCATTCTCTTGGCCGTCTCCTCCATCCTGCTGGTGGACTTGCTCCCCACACACTTCGAACATAATGTAGATCTCTCCAGGGCCATAAATGTAAATGGAGTAAGCTTCAATAATGTAGACACCAGTTCACTTGGCGCAGCACAGGTGAGACAAAGTGCTAGCCGAGGCAGAGGACTTGGTGAGAAGCCAAAAGAAGGAGCTGATAAAGAAAAGAAAAAAGAAAAAGAAAAAGAAAAAGAAGAACCAAAGAAGCCAAATGAAAATAAGCTGAAACAACCGGAACAACCAGCAGCAGGAGCAGGGGGCGAACAACCAGCAGCAGGAGCAGGAGGCGAACAACCAGCAGCAGGAGCAGGAGGCGAACAACCAGCAGCAGGAGCAGGAGGCGAACAACCAGCGGCAGGAGCAGGGGGCGAACAACCAGCAGCAGGAGCAGGAGGCGAACAACCAGCAGCAGGAGCAGGAGGCGAACAACCAGCAGCAGGAGCAGGAGGCGAACAACCAGCAGCAGGAGCAGGAGGCGAACAACCAGCAGCAGGAGCAGGGGGCGAACAACCAGCAGCAGGAGCAGGAGGCGAACAACCAGCACCAGCACCAAGGAGGGAACAACCAGCAGCAGGAGCAGGGGGCGAACAACCAGCACCAGCACCAAGGAGGGAACAACCAGCACCAGGAGCAGGTGCGGGAGATGGAGCACGAGGAGGAAACGCAGGGGCAGGTAAAGGACAGGGACAAAACAATCAGGGTGCGAATGTCCCAAATGAAAAAGTTGTGAATGATTACCTACACAAAATTAGATCTAGCGTTACCACCGAGTGGACTCCATGCAGTGTAACCTGTGGAAATGGTGTAAGAATTAGAAGAAGACAGAATGCTGGTAATAAAAAGGCAGAGGACCTTACTATGGATGACCTTGAGGTGGAAGCTTGTGTAATGGATAAGTGCGCTGGCATATTTAACGTTGTGAGTAATTCATTAGGCTTAGTCATATTGTTAGTCTTAGCATTATTCAATTAA---------------------------------------------------------------------------------------------------------------------------------------------------------------------------------------------------------------------------------------------------------------------------------------------------------------------------------------------------------------------------------------------------------------------------------------------------------------------------

>JQ219906 Macaque Singapore

ATGAAGAACTTCATTCTCTTGGCCGTCTCCTCCATCCTGCTGGTGGACTTGCTCCCCACACACTTCGAACATAATGTAGATCTCTCCAGGGCCATAAATGTAAATGGAGTAAGCTTCAATAATGTAGACACCAGTTCACTTGGCGCAGCACAGGTGAGACAAAGTGCTAGCCGAGGCAGAGGACTTGGTGAGAAGCCAAAAGAAGGAGCTGATAAAGAAAAGAAAAAAGAAAAAGAAGAAGAACCAAAGAAGCCAAATGAAAATAAGCTGAAACAACCGGATGCAGTACCAGGGGGCGAAGAACCAGCACCAGGAAGGGAACAGCCAGCACCAGGAAGGGAGGAACCAGCACCAGGAAGGGAACAGCCAGCACCAGGAAGGGAAGAACCAGCACCAGGAAGGGAACAGCCAGCACCAGGAAGGGAAGAACCAGCGCCAGGAAGGGAACAGCCAGCACCAGGAAGGGAACAGCCAGCACCAGGAAGGGAGGAACCAGCACCAGGAAGGGAACAACCAGCACCAGGAAGGGAGGAACCAGCACCAGGAAGGGAACAACCAGCACCAGGAAGGGAACAGCCAGCACCAGGAAGGGAACAGCCAGCACCGGGGGGTGAACAACCAGCACCAGGAAGGGAACAGCCAGCACCGGGTGGTGAACAACCAGCACCAGCACCAGGAGCAGGTGCGGGAGATGGAGCACGAGGAGGAAACGCAGGGGCAGGGAAAGGACAGGGACAAAACAATCAGGGTGCAAATGTCCCAAATGAAAAAGTTGTGAATGATTACCTACACAAAATTAGATCTAGCGTTACCACCGAGTGGACTCCATGCAGTGTAACCTGTGGAAATGGTGTAAGAATTAGAAGAAAGGGTCATGCAGGTAATAAAAAGGCAGAGGACCTTACTATGGATGACCTTGAGGTGGAAGCTTGTGTAATGGATAAGTGCGCTGGCATATTTAACGTTGTGAGTAATTCATTAGGCTTAGTCATATTGTTAGTCCTAGCATTATTCAATTAA---------------------------------------------------------------------------------------------------------------------------------------------------------------------------------------------------------------------------------------------------------------------------------------------------------------------------------------------------------------------------------------------------------------------------------------------------------------------------------

>JQ219905 Macaque Singapore

ATGAAGAACTTCATTCTCTTGGCCGTCTCCTCCATCCTGCTGGTGGACTTGCTCCCCACATACTTCGAACATAATGTAGATCTCTCCAGGGCCGTAAATGTAAATGGAGTAAGCTTCAATAGTGTAGACACCAGTTCACTTGGCGCAGCACAGGTAAGACAAAGTGCTAGCCGAGGCAGAGGACTTGGTGAGAAGCCAAAAGAAGGAGCTGATAAAGAAAAGAAAAAAGAAAAAGAAAAAGAAGAAGAACCAAAGAAGCCAAATGAAAATAAGCTGAAACAACCACAAGGAAATGGGGGGGCAGGTCAAGCACAACCAGAAGGAAATGGGGGGGCAGGTCAAGCACAACCGGAAGGAAATGGGGGGGCAGGTCAAGCACAACCAGAAGGAAATGGGGGGGCAGGTCAAGCACAACCGGAAGGAAATGGGGGGGCAGGTCAAGCACAACCGGAAGGAAATGGGGGGGCAGGTCAAGCACAACCGGAAGGAAATGGGGGGGCAGGTCAAGCACAACCGGAAGGAAATGGGGGGGCAGGTCAAGCACAACCACAAGGAAATGGGGGGGCAGGTCAAGCACAACCACAAGGAAATGGGGGGGCAGGTCAAGCACAACCACAAGGAAATGGGGGGGCAGGTCAAGCACAACCACAAGGAAATGGGGGGGCAGGTCAAGCACAACCGGAAGGAAATGGGGGGGCAGGTCAAGCACAACCACAAGGAAATGGGGGGGCAGGTCAAGCACAACCGGAAGGAAATAGGGAAGCTCCAGCACAACCACAAGGAAATGGGGGGGCAGGTCAAGCACAACCACAAAAAAACGAAGGAGGAAACGCAGGAGCACGGAAAGGACAGGGACAAAACAGTCAGGGTGCGAATGCCCCAAATGAAAAAGTTGTAAATGATTACCTACAGAAAATTAGATCTAGCGTTACCACCGAGTGGACTCCATGCAGTGTAACCTGTGGAAATGGTGTAAGAATTAGAAGGAGAGCTCATGCAGATAAGAAAAAGGCAGGGGACCTTACTATGGATGACCTTGAAGTGGAAGCTTGTGTAATGGATAAGTGTGCTGGCATATTTAACGCTGTGAGTAATTCATTAGGGTTAGTCATATTGTTAGTCCTAGCATTATTCAATTAA---------------------------------------------------------------------------------------------------------------------------------------------------------------------------------------------------------------------------------------------------------------------------------------------------------------------------------------------------------

>JQ219904 Macaque Singapore

ATGAAGAACTTCATTCTCTTGGCCGTCTCCTCCATCCTGCTGGTGGACTTGCTCCCCACATACTTCGAACATAATGTAGATCTCTCCAGGGCCATAAATGTAAATGGAGTAAGCTTCAATAGTGTAGACACCAGTTCACTTGGCGCAGCACAGGTAAGACAAAGTGCTAGCCGAGGCAGAGGACTTGGTGAGAAGCCAAAAGAAGGAGCTGATAAAGAAAAGAAAAAAGAAAAAGAAAAAGAAGAAGAACCAAAGAAGCCAAATGAAAATAAGCTGAAACAACCACAAGGAAATGGGGGGGCAGGTCAAGCACAACCAGAAGGAAATGGGGGGGCAGGTCAAGCACAACCGGAAGGAAATGGGGGGGCAGGTCAAGCACAACCAGAAGGAAATGGGGGGGCAGGTCAAGCACAACCGGAAGGAAATGGGGGGGCAGGTCAAGCACAACCGGAAGGAAATGGGGGGGCAGGTCAAGCACAACCGGAAGGAAATGGGGGGGCAGGTCAAGCACAACCGGAAGGAAATGGGGGGGCAGGTCAAGCACAACCGGAAGGAAATGGGGGGGCAGGTCAAGCACAACCGGAAGGAAATGGGGGGGCAGGTCAAGCACAACCACAAGGAAATGGGGGGGCAGGTCAAGCACAACCACAAGGAAATGGGGGGGCAGGTCAAGCACAACCACAAGGAAATGGGGGGGCAGGTCAAGCACAACCGGAAGGAAATAGGGAAGCTCCAGCACAACCACAAGGAAATGGGGGGGCAGGTCAAGCACAACCACAAAAAAACGAAGGAGGAAACGCAGGAGCACGGAAAGGACAGGGACAAAACAATCAGGGTGCGAATGCCCCAAATGAAAAAGTTGTAAATGATTACCTACAGAAAATTAGATCTAGCGTTACCACCGAGTGGACTCCATGCAGTGTAACCTGTGGAAATGGTGTAAGAATTAGAAGAAGAGCTCATGCGGATAAGAAAAAGGCAGAGGACCTTACTATGGATGACCTTGAAGTGGAAGCTTGTGTAATGGATAAGTGTGCTGGCATATTTAACGTTGTGAGTAATTCATTAGGGTTAGTCATATTGTTAGTCCTAGCATTATTCAATTAA------------------------------------------------------------------------------------------------------------------------------------------------------------------------------------------------------------------------------------------------------------------------------------------------------------------------------------------------------------------------------------------

>JQ219903 Macaque Singapore

ATGAAGAACTTCATTCTCTTGGCCGTCTCCTCCATCCTGCTGGTGGACTTGCTCCCCACATACTTCGAACATAATGTAGATCTCTCCAGGGCCATAAATGTAAATGGAGTAAGCTTCAATAGTGTAGACACCAGTTCACTTGGCGCAGCACAGGTAAGACAGAGTGCTAGCCGAGGCAGAGGACTTGGTGAGAAGCCAAAAGAAGGAGCTGATAAAGAAAAGAAAAAAGAAAAAGAAAAAGAAGAAGAACCAAAGAAGCCAAATGAAAATAAGCTGAAACAACCACAAGGAAATGGGGGGGCAGGTCAAGCACAACCAGAAGGAAATGGGGGGGCAGGTCAAGCACAACCGGAAGGAAATGGGGGGGCAGGTCAAGCACAACCAGAAGGAAATGGGGGGGCAGGTCAAGCACAACCGGAAGGAAATGGGGGGGCAGGTCAAGCACAACCGGAAGGAAATGGGGGGGCAGGTCAAGCACAACCGGAAGGAAATGGGGGGGCAGGTCAAGCACAACCGGAAGGAAATGGGGGGGCAGGTCAAGCACAACCGGAAGGAAATGGGGGGGCAGGTCAAGCACAACCGGAAGGAAATGGGGGGGCAGGTCAAGCACAACCACAAGGAAATGGGGGGGCAGGTCAAGCACAACCACAAGGAAATGGGGGGGCAGGTCAAGCACAACCACAAGGAAATGGGGGGGCAGGTCAAGCACAACCGGAAGGAAATAGGGAAGCTCCAGCACAACCACAAGGAAATGGGGGGGCAGGTCAAGCACAGCCACAAAAAAACGAAGGAGGAAACGCAGGAGCACGGAAAGGACAGGGACAAAACAATCAGGGTGCGAATGCCCCAAATGAAAAAGTTGTAAATGATTACCTACAGAAAATTAGATCTAGCGTTACCACCGAGTGGACTCCATGCAGTGTAACCTGTGGAAATGGTGTAAGAATTAGAAGAAGAGCTCATGCAGATAAGAAAAAGGCAGAGGACCTTACTGTGGATGACCTTGAAGTGGAAGCTTGTGTAATGGATAAGTGTGCTGGCATATTTAACGTTGTGAGTAATTCATTAGGGTTAGTCATATTGTTAGTCCTAGCATTATTCAATTAA------------------------------------------------------------------------------------------------------------------------------------------------------------------------------------------------------------------------------------------------------------------------------------------------------------------------------------------------------------------------------------------

>JQ219902 Macaque Singapore

ATGAAGAACTTCATTCTCTTGGCCGTCTCCTCCATCCTGCTGGTGGACTTGCTCCCCACATACTTCGAACATAATGTAGATCTCTCCAGGGCCATAAATGTAAATGGAGTAAGCTTCAATAGTGTAGACACCAGTTCACTTGGCGCAGCACAGGTAAGACAAAGTGCTAGCCGAGGCAGAGGACTTGGTGAGAAGCCAAAAGAAGGAGCTGATAAAGAAAAGAAAAAAGAAAAAGAAAAAGAAGAAGAACCAAAGAAGCCAAATGAAAATAAGCTGAAACAACCACAAGGAAATGGGGGGGCAGGTCAAGCACAACCAGAAGGAAATGGGGGGGCAGGTCAAGCACAACCGGAAGGAAATGGGGGGGCAGGTCAAGCACAACCAGAAGGAAATGGGGGGGCAGGTCAAGCACAACCGGAAGGAAATGGGGGGGCAGGTCAAGCACAACCGGAAGGAAATGGGGGGGCAGGTCAAGCACAACCGGAAGGAAATGGGGGGGCAGGTCAAGCACAACCGGAAGGAAATGGGGGGGCAGGTCAAGCACAACCGGAAGGAAATGGGGGGGCAGGTCAAGCACAACCGGAAGGAAATGGGGGGGCAGGTCAAGCACAACCACAAGGAAATGGGGGGGCAGGTCAAGCACAACCACAAGGAAATGGGGGGGCAGGTCAAGCACAACCACAAGGAAATGGGGGGGCAGGTCAAGCACAACCGGAAGGAAATAGGGAAGCTCCAGCACAACCACAAGGAAATGGGGGGGCAGGTCAAGCACAACCACAAAAAAACGAAGGAGGAAACGCAGGAGCACGGAAAGGACAGGGACAAAACAATCAGGGTGCGAATGCCCCAAATGAAAAAGTTGTAAATGATTACCTACAGAAAATTAGATCTAGCGTTACCACCGAGTGGACTCCATGCAGTGTAACCTGTGGAAATGGTGTAAGAATTAGAAGAAGAGCTCATGCAGATAAGAAAAAGGCAGAGGACCTTACTATGGATGACCTTGAAGTGGAAGCTTGTGTAATGGATAAGTGTGCTGGCATATTTAACGTTGTGAGTAATTCATTAGGGTTAGTCATATTGTTAGTCCTAGCATTATTCAATTAA------------------------------------------------------------------------------------------------------------------------------------------------------------------------------------------------------------------------------------------------------------------------------------------------------------------------------------------------------------------------------------------

>JQ219901 Macaque Singapore

ATGAAGAACTTCATTCTCTTGGCCGTCTCCTCCATCCTGCTGGTGGACCTGCTCCCCACATACTTCGAACATAATGTAGATCTCTCCAGGGCCATAAATGTAAATGGAGTAAGCTTCAATAGTGTAGACACCAGTTCACTTGGCGCAGCACAGGTAAGACAAAGTGCTAGCCGAGGCAGAGGACTTGGTGAGAAGCCAAAAGAAGGAGCTGATAAAGAAAAGAAAAAAGAAAAAGAAAAAGAAGAAGAACCAAAGAAGCCAAATGAAAATAAGCTGAAACAACCACAAGGAAATGGGGGGGCAGGTCAAGCACAACCAGAAGGAAATGGGGGGGCAGGTCAAGCACAACCGGAAGGAAATGGGGGGGCAGGTCAAGCACAACCAGAAGGAAATGGGGGGGCAGGTCAAGCACAACCGGAAGGAAATGGGGGGGCAGGTCAAGCACAACCGGAAGGAAATGGGGGGGCAGGTCAAGCACAACCGGAAGGAAATGGGGGGGCAGGTCAAGCACAACCGGAAGGAAATGGGGGGGCAGGTCAAGCACAACCGGAAGGAAATGGGGGGGCAGGTCAAGCACAACCGGAAGGAAATGGGGGGGCAGGTCAAGCACAACCGGAAGGAAATGGGGGGGCAGGTCAAGCACAACCGGAAGGAAATGGGGGGGCAGGTCAAGCACAACCGGAAGGAAATGGGGGGGCAGGTCAAGCACAACCACAAGGAAATGGGGGGGCAGGTCAAGCACAACCACAAAAAAACGAAGGAGGAAACGCAGGAGCACGGAAAGGACAGGGACAAAACAATCAGGGTGCGAATGCCCCAAATGAAAAAGTTGTAAATGATTACCTACAGAAAATTAGATCTAGCGTTACCACCGAGTGGACTCCATGCAGTGTAACCTGTGGAAATGGTGTAAGAATTAGAAGAAGAGCTCATGCAGATAAGAAAAAGGCAGAGGACCTTACTATGGATGACCTTGAAGTGGAAGCTTGTGTAATGGATAAGTGTGCTGGCATATTTAACGTTGTGAGTAATTCATTAGGGTTAGTCATATTGTTAGTCCTAGCATTATTCAATTAA------------------------------------------------------------------------------------------------------------------------------------------------------------------------------------------------------------------------------------------------------------------------------------------------------------------------------------------------------------------------------------------------------------------------

>JQ219899 Macaque Singapore

ATGAAGAACTTCATTCTCTTGGCCGTCTCCTCCATCCTGCTGGTGGACTTGCTCCCCACACACTTCGAACATAATGTAGATCTCTCCAGGGCCATAAATGTAAATGGAGTAAGCTTCAATAATGTAGACACCAGTTCACTTGGCGCAGCACAGGTGAGACAAAGTGCTAGCCGAGGCAGAGGACTTGGTGAGAAGCCAAAAGAAGGAGCTGATAAAGAAAAGAAAAAAGAAAAAGGAAAAGAAAAAGAAGAAGAACCAAAGAAGCCAAATGAAAATAAGCTGAAACAACCGAATGAAGGACAACCACAAGCACAGGGTGATGGAGCAAATGCAGGACAACCACAAGCACAAGGAGATGGAGCAAATGCAGGACAACCACAAGCACAGGGTGATGGAGCAAATGCAGGACAACCACAAGCACAGGGTGATGGAGCAAATGCAGGACAACCACAAGCACAAGGAGATGGAGCAAATGCAGGACAACCACAAGCACAGGGTGATGGAGCAAATGCAGGGCAACCACAAGCACAGGGTGATGGAGCAAATGCAGGACAACCACAAGCACAAGGAGATGGAGCAAATGCAGGACAACCACAAGCACAAGGAGATGGAGCAAATGCAGGACAACCACAAGCACAGGGTGATGGAGCAAATGCAGGACAACCACAAGCACAGGGTGATAGGGCGAATGCAGGACAACCACAAGCACAAGGAGATGGGGCAAATGTACCACGACAAGGAAGAAACGGGGGAGGTGCACCAGCAGGAGGAAATGAGGGGAATAAACAAGCAGGAAAAGGACAGGGACAAAACAATCAGGGTGCGAATGCCCCAAATGAAAAAGTTGTGAATGATTACCTACACAAAATTAGATCTAGCGTTACCACCGAGTGGACTCCATGCAGTGTAACCTGTGGAAATGGTGTAAGAATTAGAAGAAAAGCTCATGCAGGTAATAAAAAGGCAGAGGACCTTACTATGGATGACCTTGAGGTGGAAGCTTGTGTAATGGATAAGTGCGCTGGCATATTTAACGTTGTGAGTAATTCATTAGGCTTAGTCATATTGTTAGTCCTAGCATTATTCAATTAA---------------------------------------------------------------------------------------------------------------------------------------------------------------------------------------------------------------------------------------------------------------------------------------------------------------------------------------------------------------------------------------------------------

>JQ219898 Macaque Singapore

ATGAAGAACTTCATTCTCTTGGCCGTCTCCTCCATCCTGCTGGTGGACTTGCTCCCCACACACTTCGAACATAATGTAGATCTCTCCAGGGCCATAAATGTAAATGGAGTAAGCTTCAATAATGTAGACACCAGTTCACTTGGCGCAGCACAGGTGAGACAAAGTGCTAGCCGAGGCAGAGGACTTGGTGAGAAGCCAAAAGAAGGAGCTGATAAAGAAAAGAAAAAAGAAAAAGAAAAAGAAAAAGAAGAACCAAAGAAGCCAAATGAAAATAAGCTGAAACAACCGGAACAACCAGCAGCAGGAGCAGGGGGCGAACAACCAGCAGCAGGAGCAGGAGGCGAACAACCAGCAGCAGGAGCAGGAGGCGAACAACCAGCAGCAGGAGCAGGAGGCGAACAACCAGCGGCAGGAGCAGGGGGCGAACAACCAGCAGCAGGAGCAGGAGGCGAACAACCAGCAGCAGGAGCAGGAGGCGAACAACCAGCAGCAGGAGCAGGAGGCGAACAACCAGCAGCAGGAGCAGGAGGCGAACAACCAGCAGCAGGAGCAGGGGGCGAACAACCAGCAGCAGGAGCAGGAGGCGAACAACCAGCACCAGCACCAAGGAGGGAACAACCAGCAGCAGGAGCAGGGGGCGAACAACCAGCACCAGCACCAAGGAGGGAACAACCAGCACCAGGAGCAGGTGCGGGAGATGGAGCACGAGGAGGAAACGCAGGGGCAGGTAAAGGACAGGGACAAAACAATCAGGGTGCGAATGTCCCAAATGAAAAAGTTGTGAATGATTACCTACACAAAATTAGATCTAGCGTTACCACCGAGTGGACTCCATGCAGTGTAACCTGTGGAAATGGTGTAAGAATTAGAAGAAGACAGAATGCTGGTAATAAAAAGGCAGAGGACCTTACTATGGATGACCTTGAGGTGGAAGCTTGTGTAATGGATAAGTGCGCTGGCATATTTAACGTTGTGAGTAATTCATTAGGCTTAGTCATATTGTTAGTCTTAGCATTATTCAATTAA---------------------------------------------------------------------------------------------------------------------------------------------------------------------------------------------------------------------------------------------------------------------------------------------------------------------------------------------------------------------------------------------------------------------------------------------------------------------------

>JQ219897 Macaque Singapore

ATGAAGAACTTCATTCTCTTGGCCGTCTCCTCCATCCTGCTGGTGGACTTGCTCCCCACACACTTCGAACATAATGTAGATCTCTCCAGGGCCATAAATGTAAATGGAGTAAGCTTCAATAATGTAGACACCAGTTCACTTGGCGCAGCACAGGTGAGACAAAGTGCTAGCCGAGGCAGAGGACTTGGTGAGAAGCCAAAAGAAGGAGCTGATAAAGAAAAGAAAAAAGAAAAAGGAAAAGAAAAAGAAGAAGAACCAAAGAAGCCAAATGAAAATAAGCTGAAACAACCGAATGAAGGACAACCACAAGCACAGGGTGATGGAGCAAATGCAGGACAACCACAAGCACAAGGAGATGGAGCAAATGCAGGACAACCACAAGCACAGGGTGATGGAGCAAATGCAGGACAACCACAAGCACAGGGTGATGGAGCAAATGCAGGACAACCACAAGCACAAGGAGATGGAGCAAATGCAGGACAACCACAAGCACAGGGTGATGGAGCAAATGCAGGGCAACCACAAGCACAGGGTGATGGAGCAAATGCAGGACAACCACAAGCACAAGGAGATGGAGCAAATGCAGGACAACCACAAGCACAAGGAGATGGAGCAAATGCAGGACAACCACAAGCACAGGGTGATGGAGCAAATGCAGGACAACCACAAGCACAGGGTGATAGGGCGAATGCAGGACAACCACAAGCACAAGGAGATGGGGCAAATGTACCACGACAAGGAAGAAACGGGGGAGGTGCACCAGCAGGAGGAAATGAGGGGAATAAACAAGCAGGAAAAGGACAGGGACAAAACAATCAGGGTGCGAATGCCCCAAATGAAAAAGTTGTGAATGATTACCTACACAAAATTAGATCTAGCGTTACCACCGAGTGGACTCCATGCAGTGTAACCTGTGGAAATGGTGTAAGAATTAGAAGAAAAGCTCATGCAGGTAATAAAAAGGCAGAGGACCTTACTATGGATGACCTTGAGGTGGAAGCTTGTGTAATGGATAAGTGCGCTGGCATATTTAACGTTGTGAGTAATTCATTAGGCTTAGTCATATTGTTAGTCCTAGCATTATTCAATTAA---------------------------------------------------------------------------------------------------------------------------------------------------------------------------------------------------------------------------------------------------------------------------------------------------------------------------------------------------------------------------------------------------------

>XM_002258966 Human Peninsular Malaysia

ATGAAGAACTTCATTCTCTTGGCCGTCTCCTCCATCCTGCTGGTGGACTTGCTCCCCACACACTTCGAACATAATGTAGATCTCTCCAGGGCCATAAATGTAAATGGAGTAAGCTTCAATAATGTAGACACCAGTTCACTTGGCGCAGCACAGGTGAGACAAAGTGCTAGCCGAGGCAGAGGACTTGGTGAGAAGCCAAAAGAAGGAGCTGATAAAGAAAAGAAAAAAGAAAAAGGAAAAGAAAAAGAAGAAGAACCAAAGAAGCCAAATGAAAATAAGCTGAAACAACCGAATGAAGGACAACCACAAGCACAGGGTGATGGAGCAAATGCAGGACAACCACAAGCACAAGGAGATGGAGCAAATGCAGGACAACCACAAGCACAGGGTGATGGAGCAAATGCAGGACAACCACAAGCACAGGGTGATGGAGCAAATGCAGGACAACCACAAGCACAAGGAGATGGAGCAAATGCAGGACAACCACAAGCACAGGGTGATGGAGCAAATGCAGGGCAACCACAAGCACAGGGTGATGGAGCAAATGCAGGACAACCACAAGCACAAGGAGATGGAGCAAATGCAGGACAACCACAAGCACAAGGAGATGGAGCAAATGCAGGACAACCACAAGCACAGGGTGATGGAGCAAATGCAGGACAACCACAAGCACAGGGTGATAGGGCGAATGCAGGACAACCACAAGCACAAGGAGATGGGGCAAATGTACCACGACAAGGAAGAAACGGGGGAGGTGCACCAGCAGGAGGAAATGAGGGGAATAAACAAGCAGGAAAAGGACAGGGACAAAACAATCAGGGTGCGAATGCCCCAAATGAAAAAGTTGTGAATGATTACCTACACAAAATTAGATCTAGCGTTACCACCGAGTGGACTCCATGCAGTGTAACCTGTGGAAATGGTGTAAGAATTAGAAGAAAAGCTCATGCAGGTAATAAAAAGGCAGAGGACCTTACTATGGATGACCTTGAGGTGGAAGCTTGTGTAATGGATAAGTGCGCTGGCATATTTAACGTTGTGAGTAATTCATTAGGCTTAGTCATATTGTTAGTCCTAGCATTATTCAATTAA---------------------------------------------------------------------------------------------------------------------------------------------------------------------------------------------------------------------------------------------------------------------------------------------------------------------------------------------------------------------------------------------------------

>KF861750 Human Peninsular Malaysia

ATGAAGAACTTCATTCTCTTGGCCGTCTCCTCCATCCTGCTGGTGGACTTGCTCCCCACACACTTCGAACATAATGTAGATCTCTCCAGGGCCATAAATGTAAATGGAGTAAGCTTCAATAATGTAGACACCAGTTCACTTGGCGCAGCACAGGTGAGACAAAGTGCTAGCCGAGGCAGAGGACTTGGTGAGAAGCCAAAAGAAGGAGCTGATAAAGAAAAGAAAAAAGAAAAAGGAAAAGAAAAAGAAGGAGAACCAAAGAAGCCAAATGAAAATAAGCTGAAACAACCGAATGAAGGACAACCACAAGCACAGGGTGATGGAGCAAATGCAGGACAGCCACAAGCACAAGGAGATGGAGCAAATGCAGGACAACCACAAGCACAGGGTGATGGAGCAAATGCAGGACAACCACAAGCACAGGGTGATGGAGCAAATGCAGGACAACCACAAGCACAAGGAGATGGAGCAAATGCAGGACAACCACAAGCACAGGGTGATGGAGCAAATGCAGGGCAACCACAAGCACAGGGTGATGGAGCAAATGCAGGACAACCACAAGCACAAGGAGATGGAGCAAATGCAGGACAACCACAAGCACAAGGAGATGGAGCAAATGCAGGACAACCACAAGCACAGGGTGATGGAGCAAATGCAGGACAACCACAAGCACAGGGTGATAGGGCGAATGCAGGACAACCACAAGCACAAGGAGATGGGGCAAATGTACCACGACAAGGAAGAAACGGGGGAGGTGCACCAGCAGGAGGAAATGAGGGGAATAAACAAGCAGGAAAAGGGCAGGGACAAAACAATCAGGGTGCGAATGCCCCAAATGAAAAAGTTGTGAATGATTACCTACACAAAATTAGATCTAGCGTTACCACCGAGTGGACTCCATGCAGTGTAACCTGTGGAAATGGTGTAAGAATTAGAAGAAAAGCTCATGCAGGTAATAAAAAGGCAGAGGACCTTACTATGGATGACCTTGAGGTGGAAGCTTGTGTAATGGATAAGTGCGCTGGCATATTTAACGTTGTGAGTAATTCATTAGGCTTAGTCATATTGTTAGTCCTAGCATTATTCAATTAA---------------------------------------------------------------------------------------------------------------------------------------------------------------------------------------------------------------------------------------------------------------------------------------------------------------------------------------------------------------------------------------------------------

>KF861749 Human Peninsular Malaysia

ATGAAGAACTTCATTCTCTTGGCCGTCTCCTCCATCCTGCTGGTGGACTTGCTCCCCACACACTTCGAACATAATGTAGATCTCTCCAGGGCCATAAATGTAAATGGAGTAAGCTTCAATAATGTAGACACCAGTTCACTTGGCGCAGCACAGGTGAGACAAAGTGCTAGCCGAGGCAGAGGACTTGGTGAGAAGCCAAAAGAAGGAGCTGATAAAGAAAAGAAAAAAGAAAAAGGAAAAGAAAAAGAAGAAGAACCAAAGAAGCCAAATGAAAATAAGCTGAAACAACCGAATGAAGGACAACCACAAGCACAGGGTGATGGAGCAAATGCAGGACAACCACAAGCACAAGGAGATGGAGCAAATGCAGGACAACCACAAGCACAGGGTGATGGAGCAAATGCAGGACAACCACAAGCACAGGGTGATGGAGCAAATGCAGGACAACCACAAGCACGAGGAGATGGAGCAAATGCAGGACAACCACAAGCACAGGGTGATGGAGCAAATGCAGGACAACCACAAGCACAGGGTGATGGAGCAAATGCAGGGCAACCACAAGCACAGGGTGATGGAGCAAATGCAGGACAACCACAAGCACAAGGAGATGGAGCAAATGCAGGACAACCACAAGCACAAGGAGATGGAGCAAATGCAGGACAACCACAAGCACAGGGTGATGGAGCAAATGCAGGACAACCACAAGCACAGGGTGATAGGGCGAATGCAGGGCAACCACAAGCACAAGGAGATGGGGCAAATGTACCACGACAAGGAAGAAACGGGGGAGGTGCACCAGCAGGAGGAAATGAGGGGAATAAACAAGCAGGAAAAGGACAGGGACAAAACAATCAGGGTGCGAATGCCCCAAATGAAAAAGTTGTGAATGATTACCTACACAAAATTAGATCTAGCGTTACCACCGAGTGGACTCCATGCAGTGTAACCTGTGGAAATGGTGTAAGAATTAGAAGAAAAGCTCATGCAGGTAATAAAAAGGCAGAGGACCTTACTATGGATGACCTTGAGGTGGAAGCTTGTGTAATGGATAAGTGCGCTGGCATATTTAACGTTGTAAGTAATTCATTAGGCTTAGTCATATTGTTAGTCCTAGCATTATTCAATTAA---------------------------------------------------------------------------------------------------------------------------------------------------------------------------------------------------------------------------------------------------------------------------------------------------------------------------------------------------------------------

>KF861748 Human Peninsular Malaysia

ATGAGGAACTTCATTCTCTTGGCCGTCTCCTCCATCCTGCTGGTGGACTTGTTCCCCACACACTTCGAACATAATGTAGATCTCTCCAGGGCCATAAATGTAAATGGAGTAAGCTTCAATAATGTAGACACCAGTTCACTTGGCGCAGCACAGGTGAGACAAAGTGCTAGCCGAGGCAGAGGACTTGGTGAGAAGCGAAAAGAAGGAGCTGATAAGGAAAAGAAAAAAGAAAAAGAAGAAGAACCAAAGAAGCCAAATGAAAATAAGCTGAAACAACCGGATCAAGCAGGACCAGGGGGCGAACAAGCAGGACCAGGAGGCGAACAAGCAGGACCAGGAGGCGAACAAGCAGGACCAGGAGGCGAACAAGCAGGACCAAGACCAGGGGGCGAACAAGCAGGACCAGGAGGCGAACAAGCAGGACCAAGACCAGGGGGCGAACAAGCAGGACCAAGACCAGGGGGCGAACAAGCAGGACCAAGACCAGGGGGCGAACAAGCAGGACCAGGAGGCGAACAAGCAGGACCAGGGGGCGAACAAGCAGGACCAGGGGGCGAACAAGCAGGACCAAGACCAGGGGGCGAACAAGCAGGACCAGGAGGCGAACAAGCAGGACCAAGACCAGGGGGCGAACAAGCAGGACCAGGGGGCGAACAACCAGCACCAAGACCAGGGGGAGAACAACCAGCACCAGCACCAAGGAGGGAACAACCAGCACCAGGACCAGGGGGCGAACAACCAGCACCAGGAGCAGGTGCGGGAGATGGAGCACGAGGAGGAAACCCAGGGGCAGGTAAAGGACAGGGACAAAACAATCAGGGTGCAAATGTCCCAAATGAAAAAGTTGTGAATGATTACCTACACAAAATTAGATCTAGCGTTACCACCGAGTGGACTCCATGCAGTGTAACCTGTGGAAATGGTGTAAGAATTAGAAGAAGACAGAATGCTGGTAATAAAAAGGCAGAGGACCTTACTATGGATGACCTTGAGGTGGAAGCTTGTGTAATGGATAAGTGCGCTGGCATATTTAACGTTGTGAGTAATTCATTAGGGTTAGTCACATTGTTAGTCCTAGCATTATTCAATTAA---------------------------------------------------------------------------------------------------------------------------------------------------------------------------------------------------------------------------------------------------------------------------------------------------------------------------------------------------------------------------------------------------------

>KF861747 Human Peninsular Malaysia

ATGAAGAACTTCATTCTCTTGGCCGTCTCCTCCATCCTGCTGGTGGACTTGTTCCCCACACACTTCGAACATAATGTAGATCTCTCCAGGGCCATAAATGTAAATGGAGTAAGCTTCAATAATGTAGACACCAGTTCACTTGGCGCAGCACAGGTGAGACAAAGTGCTAGCCGAGGCAGAGGACTTGGTGAGAAGCCAAAAGAAGGAGCTGATAAAGAAAAGAAAAAAGAAAAAGAAGAAAAACCAAAGAAGCCAAATGAAAATAAGCTGAAACAACCAGGGGGCGAACAATCAGCAGCAGGGGGCGAACAACCAGCAGCAGGGGGCGAACAACCAGCAGCAGGGGGTGAACAACCAGCAGCAGGGGGTGAACAACCAGCAGCAGGGGGTGAACAACCAGCAGCAGGGGGCGAACGACCAGCAGCAGGGGGCGAACAACCAGCAGCAGGGGGTGAACAACCAGCAGCAGGGGGCGAACGACCAGCAGCAGGGGGTGAACAACCAGCAGCAGGGGGCGAACGACCAGCAGCAGGGGGCGAACAACCAGCAGCAGGGGGCGAACAACCAGCAGCAGGGGGTGAACAACCAGCGCCAGCACCAAGGAGGGAACAACCAGCAGCAGGGGGCGAACAACCAGCACCAGCACCAAGGAGGGAACAACCAGCACCAGGAGCAGGTGCGGGAGATGGAGCACGAGGAGGAAACGCAGGGGCAGGTAAAGGACAGGGACAAAACAATCAGGGTGCGAATGTCCCAAATGAAAAAGTTGTGAATGATTACCTACACAAAATTAGATCTAGCGTTACCACCGAGTGGACTCCATGCAGTGTAACCTGTGGAAATGGTGTAAGAATTAGAAGAAGACAGAATGCTGGTAATAAAAAGGCAGAGGACCTTACTATGGATGACCTTGAGGTGGAAGCTTGTGTAATGGATAAGTGCGCTGGCATATTTAACGTTGTGAGTAATTCATTAGGGTTAGTCATATTGTTAGTCCTAGCATTATTCAATTAA---------------------------------------------------------------------------------------------------------------------------------------------------------------------------------------------------------------------------------------------------------------------------------------------------------------------------------------------------------------------------------------------------------------------------------------------------------------------------------------

>KF861746 Human Peninsular Malaysia

ATGAGGAACTTCATTCTCTTGGCCGTCTCCTCCATCCTGCTGGTGGACTTGTTCCCCACACACTTCGAACATAATGTAGATCTCTCCAGGGCCATAAATGTAAATGGAGTAAGCTTCAATAATGTAGACACCAGTTCACTTGGCGCAGCACAGGTGAGACAAAGTGCTAGCCGAGGCAGAGGACTTGGTGAGAAGCCAAAAGAAGGAGCTGATAAAGAAAAGAAAAAAGAAAAAGGAAAAGAAAAAGAAGAAGAACCAAAGAAGCCAAATGAAAATAAGCTGAAACAACCGAATGAAGGACAACCACAAGCACAGGGTGATGGAGCAAATGCAGGACAACCACAAGCACAAGGAGATGGAGCAAATGCAGGACAACCACAAGCACAAGGAGATGGAGCAAATGCAGGACAACCACAAGCACAGGGTGATGGAGCAAATGCAGGACAACCACAAGCACAGGGTGATGGAGCAAATGCAGGACAACCACAAGCACAAGGAGATGGAGCAAATGCAGGACAACCACAAGCACAGGGTGATGGAGCAAATGCAGGGCAACCACAAGCACAGGGTGATGGAGCAAATGCAGGACAACCACAAGCACAAGGAGATGGAGCAAATGCAGGACAACCACAAGCACAAGGAGATGGAGCAAATACAGGACAACCACAAGCACAGGGTGATGGAGCAAATGCAGGACAACCACAAGCACAGGGTGATAGGGCGAATGCAGGACAACCACAAGCACAAGGAGATGGGGCAAATGTACCACGACAAGGAAGAAACGGGGGAGGTGCACCAGCAGGAGGAAATGAGGGGAATAAACAAGCAGGAAAAGGACAGGGACAAAACAATCAGGGTGCGAATGCCCCAAATGAAAAAGTTGTGAATGATTACCTACACAAAATTAGATCTAGCGTTACCACCGAGTGGACTCCATGCAGTGTAACCTGTGGAAATGGTGTAAGAATTAGAAGAAAAGCTCATGCAGGTAATAAAAAGGCAGAGGACCTTACTATGGATGACCTTGAGGTGGAAGCTTGTGTAATGGATAAGTGCGCTGGCATATTTAACGTTGTGAGTAATTCATTAGGCTTAGTCATATTGTTAGTCCTAGCATTATTCAATTAA---------------------------------------------------------------------------------------------------------------------------------------------------------------------------------------------------------------------------------------------------------------------------------------------------------------------------------------------------------------------

>KF861745 Human Peninsular Malaysia

ATGAAGAACTTCATTCTCTTGGCCGTCTCCTCCATCCTGCTGGTGGACTTGCTCCCCACACACTTCGAACATAATGTAGATCTCTCCAGGGCCATAAATGTAAATGGAGTAAGCTTCAATAATGTAGACACCAGTTCACCTGGCGCAGCACAGGTAAGACAAAGTGCTAGCCGAGGCAGAGGACTTGGTGAGAAGCCAAAAGAAGGAGCTGATAAAGAAAAGAAAAAAGAAAAAGAAAAAGAAAAAGAAGAAGAACCAAAGAAGCCAAATGAAAATAAGCTGAAACAACCGGAACAACCAGCAGCAGGAGCAGGGGGCGAACAACCAGCAGCAGGAGCAGGAGGCGAACAACCAGCAGCAGGAGCAGGAGGCGAACAACCAGCAGCAGGAGCAGGAGGCGAACAACCAGCAGCAGGAGCAAGAGGCGAACAACCAGCAGCAGGAGCAGGAGGCGAACAACCAGCAGCAGGAGCAAGAGGCGAACAACCAGCAGCAGGAGCAGGAGGCGAACAACCAGCAGCAGGAGCAGGAGGCGAACAACCAGCAGCAGGAGCAGGAGGCGAACAACCAGCAGCAGGAGCAGGGGGCGAACAACCAGCAGCAGGAGCAGGAGGCGAACAACCAGCACCAGCACCAAGGAGGGAACAACCAGCAGCAGGAGCAGGGGGCGAACAACCAGCACCAGCACCAAGGAGGGAACAACCAGCACCAGGAGCAGGTGCGGGAGATGGAGCACGAGGAGGAAACGCAGGGGCAGGTAAAGGACAGGGACAAAACAATCAGGGTGCGAATGTCCCAAATGAAAAAGTTGTGAATGATTACCTACGCAAAATTAGATCTAGCGTTACCACCGAGTGGACTCCATGCAGTGTAACCTGTGGAAATGGTGTAAGAATTAGAAGAAAAGGTCATGCAGGTAATAAAAAGGCAGAGGACCTTACTATGGATGACCTTGAGGTGGAAGCTTGTGTAATGGATAAGTGCGCTGGCATATTTAACGTTGTGAGTAATTCATTAGGCTTAGTCATATTGTTAGTCCTAGCATTATTCAATTAA---------------------------------------------------------------------------------------------------------------------------------------------------------------------------------------------------------------------------------------------------------------------------------------------------------------------------------------------------------------------------------------------------------------------------------------------

>KF861744 Human Peninsular Malaysia

ATGAAGAACTTCATTCTCTTGGCCGTCTCCTCCATCCTGCTGGTGGACTTGCTCCCCACACACTTCGAACATAATGTAGATCTCTCCAGGGCCATAAATGTAAATGGAGTAAGCTTCAATAATGTAGACACCAGTTCACTTGGCGCAGCACAGGTGAGACAAAGTGCTAGCCGAGGCAGAGGACTTGGTGAGAAGCCAAAAGAAGGAGCTGATAAAGAAAAGAAAAAAGAAAAAGGAAAAGAAAAAGAAGAAGAACCAAAGAAGCCAAATGAAAATAAGCTGAAACAACCGAATGAAGGACAACCACAAGCACAGGGTGATGGAGCAAATGCAGGACAACCACAAGCACAAGGAGATGGAGCAAATGCAGGACAACCACAAGCACAAGGAGATGGAGCAAATGCAGGACAACCACAAGCACAGGGTGATGGAGCAAATGCAGGACAACCACAAGCACAGGGTGATGGAGCAAATGCAGGACAACCACAAGCACAAGGAGATGGAGCAAATGCAGGACAACCACAAGCACAGGGTGATGGAGCAAATGCAGGGCAACCACAAGCACAGGGTGATGGAGCAAATGCAGGACAACCACAAGCACAAGGAGATGGAGCAAATGCAGGACAACCACAAGCACAAGGAGATGGAGCAAATGCAGGACAACCACAAGCACAGGGTGATGGAGCAAATGCAGGACAACCACAAGCACAGGGTGATAGGGCGAATGCAGGACAACCACAAGCACAAGGAGATGGGGCAAATGTACCACGACAAGGAAGAAACGGGGGAGGTGCACCAGCAGGAGGAAATGAGGGGAATAAACAAGCAGGAAAAGGACAGGGACAAAACAATCAGGGTGCGAATGCCCCAAATGAAAAAGTTGTGAATGATTACCTACACAAAATTAGATCTAGCGTTACCACCGAGTGGACTCCATGCAGTGTAACCTGTGGAAATGGTGTAAGAATTAGAAGAAAAGCTCATGCAGGTAATAAAAAGGCAGAGGACCTTACTATGGATGACCTTGAAGTGGAAGCTTGTGTAATGGATAAGTGTGCTGGCATATTTAACGTTGTGAGTAATTCATTAGGGTTAGTCATATTGTTAGTCCTAGCATTATTCAATTAA---------------------------------------------------------------------------------------------------------------------------------------------------------------------------------------------------------------------------------------------------------------------------------------------------------------------------------------------------------------------

>KF861743 Human Peninsular Malaysia

ATGAAGAACTTCATTCTCTTGGCCGTCTCCTCCATCCTGCTGGTGGACTTGCTCCCCACACACTTCGAACATAATGTAGATCTCTCCAGGGCCATAAATGTAAATGGAGTAAGCTTCAATAATGTAGACACCAGTTCACTTGGCGCAGCACAGGTAAGACAAAGTGCTAGCCGAGGCAGAGGACTTGGTGAGAAGCCAAAAGAAGGAGCTGATAAAGAAAAGAAAAAAGAAAAAGAAAAAGAAAAAGAAGAAGAACCAAAGAAGCCAAATGAAAATAAGCTGAAACAACCGGAAGGAAATCAAGATGGGCGAGCACAACCGGAAGGAAATCAGGATGGTCGAGCGCAACCGGAAGGAAATCAGGATGGTCGAGCTCAACCGGAAGGAAATCAAGATGGGCGAGCACAACCGGAAGGAAATCAGGATGGTCGAGCGCAACCGGAAGGAAATCAAGATGGGCGAGCACAACCGGAAGGAAATCAGGATGGTCGAGCACAACCGGAAGGAAATCAGGATGGTCGAGCGCAACCGGAAGGAAATCAGGATGGACGAGCACAACCGGAAGGAAATAGGGAAGCTCCAGCACAACCACAAGGAAATGGGGGGGCAGGTCAAGCACAACCGGAAGGAAATAGGGAAGCTCCAGCACAACCACAAGGAAATGGGGGGGCAGGTCAAGCACAACCGGAAGGAAATAGGGAAGCTCCAGCACAACCACAAGGAAATGGGGGGGCAGGTCAAGCACAACCACAAAAAAACGAAGGAGGAAACGCAGGAGCACGGAAAGGACAGGGACAAAACAATCAGGGTGCGAATGCCCCAAATGAAAAAGTTGTAAATGATTACCTACAGAAAATTAGATCTAGCGTTACCACCGAGTGGACTCCATGCAGTGTAACCTGTGGAAATGGTGTAAGAATTAGAAGAAGAGCTCATGCAGATAAGAAAAAGGCAGAGGACCTTACTATGGATGACCTTGAAGTGGAAGCTTGTGTAATGGATAAGTGTGCTGGCATATTTAACGTTGTGAGTAATTCATTAGGGTTAGTCATATTGTTAGTCCTAGCATTATTCAATTAA---------------------------------------------------------------------------------------------------------------------------------------------------------------------------------------------------------------------------------------------------------------------------------------------------------------------------------------------------------------------------------------------------------------------

>KF861742 Human Peninsular Malaysia

ATGAAGAACTTCATTCTCTTGGCCGTCTCCTCCATCCTGCTGGTGGACTTGCTCCCCACACACTTCGAACATAATGTAGATCTCTCCAGGGCCATAAATGTAAATGGAGTAAGCTTCAATAATGTAGACACCAGTTCACTTGGCGCAGCACAGGTAAGACAAAGTGCTAGCCGAGGCAGAGGACTTGGTGAGAAGCCAAAAGAAGGAGATGATAAAGAAAAGAAAAAAGAAAAAGAAAAAGAAGAAGAACCAAAGAAGCTAAATGAAAATAAGCCGAAACAACCGAATGTAGAAGGTGATGGAGCTAAGCTGAAACAACCGAATGAAGAAGGTGATGGAGCTAAGCTGAAACAACCGAATGAAGAAGGTGATGGAGCTAAGCTGAAACAACCGAATGCAGAAGGTGGAGCTAAGCTGAAACAACCGAATGCAGAAGGTGGAGCAAATGCAGGACAACCGAATGCAGAAGGTGGAGCAAATGCAGGACAACCGAATGCAGAAGGTGGAGCAAATGCAGGACAACCGAATGCAGAAGGTGGAGCAAATGCAGGACAACCGAATGCAGAAGGTGGAGCAAATGCAGGACAACCGAATGCAGAAGGTGGAGCGAATGCAGGACAACCGAATGCAGAAGGTGGAGCAAATGCACGACAACCGAATGCAGAAGGTGGAGCAAATGCACGACAACCGAATGCAGAAGGTGGAGCAAATGCACGACAACCTAATGCAGAAGGTGGAGCAAATGCAGGACAACCGAATGCAGAAGGTGGAGCAAATGCAGGACAACCGAATGCAGAAGGTGGAGCAAATGCACGACAGCCACAGGCAGAAGGTGGTGGAGCAAATGCACGACAGCCACAGGCAGAAGGTGGTGGAGCAAATGCACGACAAGGAGGAAATGAGGGGAATAAACAAGCAGGAAAAGGACAGGGACAAAACAATCAGGGTGCGAATGCCCCAAATGAAAAAGTTGTAAATGATTACCTACAGAAAATTAGATCTAGCGTTACCACCGAGTGGACTCCATGCAGTGTAACCTGTGGAAATGGTGTAAGAATTAGAAGAAGAGCTCATGCAGATAAGAAAAAGGCAGAGGACCTTACTATGGATGACCTTGAAGTGGAAGCTTGTGTAATGGATAAGTGTGCTGGCATATTTAACGTTGTGAGTAATTCATTAGGGTTAGTAATATTGTTAGTCCTAGCATTATTCAATTAA---------------------------------------------------------------------------------------------------------------------------------------------------------------------------------------------------------------------------------------------------------------------------

>KF861741 Human Peninsular Malaysia

ATGAAGAACTTCATTCTCTTGGCCGTCTCCTCCATCCTGCTGGTGGACTTGCTCCCCACACACTTCGAACATAATGTAGATCTCTCCAGGGCCATAAATGTAAATGGAGTAAGCTTCAATAATGTAGACACCAGTTCACTTGGCGCAGCACAGGTAAGACAAAGTGCTAGCCGAGGCAGAGGACTTGGTGAGAAGCCAAAAGAAGGAGATGATAAAGAAAAGAAAAAAGAAAAAGAAAAAGAAGAAGAACCAAAGAAGCTAGATGAAAATAAGCCGAAACAACCGAATGTAGAAGGTGATGGAGCTAAGCTGAAACAACCGAATGAAGAAGGTGATGGAGCTAAGCTGAAACAACCGAATGAAGAAGGTGATGGAGCTAAGCTGAAACAACCGAATGCAGAAGGTGGAGCTAAGCTGAAACAACCGAATGCAGAAGGTGGAGCAAATGCAGGACAACCGAATGCAGAAGGTGGAGCAAATGCAGGACAACCGAATGCAGAAGGTGGAGCAAATGCAGGACAACCGAATGCAGAAGGTGGAGCAAATGCAGGACAACCGAATGCAGAAGGTGGAGCAAATGCAGGACAACCGAATGCAGAAGGTGGAGCAAATGCAGGACAACCGAATGCAGAAGGTGGAGCAAATGCACGACAACCGAATGCAGAAGGTGGAGCAAATGCACGACAACCGAATGCAGAAGGTGGAGCAAATGCACGACAACCTAATGCAGAAGGTGGAGCAAATGCAGGACAACCGAATGCAGAAGGTGGAGCAAATGCAGGACAACCGAATGCAGAAGGTGGAGCAAATGCACGACAGCCACAGGCAGAAGGTGGTGGAGCAAATGCACGACAGCCACAGGCAGAAGGTGGTGGAGCAAATGCACGACAAGGAGGAAATGAGGGGAATAAACAAGCAGGAAAAGGACAGGGACAAAACAATCAGGGTGCGAATGCCCCAAATGAAAAAGTTGTAAATGATTACCTACAGAAAATTAGATCTAGCGTTACCACCGAGTGGACTCCATGCAGTGTAACCTGTGGAAATGGTGTAAGAATTAGAAGAAGAGCTCATGCAGATAAGAAAAAGGCAGAGGACCTTACTATGGATGACCTTGAAGTGGAAGCTTGTGTAATGGATAAGTGTGCTGGCATATTTAGCGTTGTGAGTAATTCATTAGGGTTAGTAATATTGTTAGTCCTAGCATTATTCAATTAA---------------------------------------------------------------------------------------------------------------------------------------------------------------------------------------------------------------------------------------------------------------------------

>KF861740 Human Peninsular Malaysia

ATGAAGAACTTCATTCTCTTGGCCGTCTCCTCCATCCTGCTGGTGGACTTGCTCCCCACACACTTCGAACATAATGTAGATCTCTCCAGGGCCATAAATGTAAATGGAGTAAGCTTCAATAATGTAGACACCAGTTCACTTGGCGCAGCACAGGTAAGACAAAGTGCTAGCCGAGGCAGAGGACTTGGTGAGAAGCCAAAAGAAGGAGATGATAAAGAAAAGAAAAAAGAAAAAGAAAAAGAAGAAGAACCAAAGAAGCTAAATGAAAATAAGCCGAAACAACCGAATGTAGAAGGTGATGGAGCTAAGCTGAAACAACCGAATGAAGAAGGTGATGGAGCTAAGCTGAAACAACCGAATGAAGAAGGTGATGGAGCTAAGCTGAAACAACCGAATGCAGAAGGTGGAGCTAAGCTGAAACAACCGAATGCAGAAGGTGGAGCAAATGCAGGACAACCGAATGCAGAAGGTGGAGCAAATGCAGGACAACCGAATGCAGAAGGTGGAGCAAATGCAGGACAACCGAATGCAGAAGGTGGAGCAAATGCAGGACAACCGAATGCAGAAGGTGGAGCAAATGCAGGACAACCGAATGCAGAAGGTGGAGCAAATGCAGGACAACCGAATGCAGAAGGTGGAGCAAATGCACGACAACCGAATGCAGAAGGTGGAGCAAATGCACGACAACCGAATGCAGAAGGTGGAGCAAATGCACGACAACCTAATGCAGAAGGTGGAGCAAATGCAGGACAACCGAATGCAGAAGGTGGAGCAAATGCAGGACAACCGAATGCAGAAGGTGGAGCAAATGCACGACAGCCACAGGCAGAAGGTGGTGGAGCAAATGCACGACAGCCACAGGCAGAAGGTGGTGGAGCAAATGCACGACAAGGAGGAAATGAGGGGAATAAACAAGCAGGAAAAGGACAGGGACAAAACAATCAGGGTGCGAATGCCCCAAATGAAAAAGTTGTAAATGATTACCTACAGAAAATTAGATCTAGCGTTACCACCGAGTGGACTCCATGCAGTGTAACCTGTGGAAATGGTGTAAGAATTAGAAGAAGAGCTCATGCAGATAAGAAAAAGGCAGAGGACCTTACTATGGATGACCTTGAAGTGGAGGCTTGTGTAATGGATAAGTGTGCTGGCATATTTAACGTTGTGAGTAATTCATTAGGGTTAGTAATATTGTTAGTCCTAGCATTATTCAATTAA---------------------------------------------------------------------------------------------------------------------------------------------------------------------------------------------------------------------------------------------------------------------------

>KF861739 Human Peninsular Malaysia

ATGAAGAACTTCATTCTCTTGGCCGTCTCCTCCATCCTGCTGGTGGACTTGCTCCCCACACACTTCGAACATAATGTAGATCTCTCCAGGGCCATAAATGTAAATGGAGTAAGCTTCAATAATGTAGACACCAGTTCACTTGGCGCAGCACAGGTAAGACAAAGTGCTAGCCGAGGCAGAGGACTTGGTGAGAAGCCAAAAGAAGGAGATGATAAAGAAAAGAAAAAAGAAAAAGAAAAAGAAGAAGAACCAAAGAAGCTAAATGAAAATAAGCCGAAACAACCGAATGTAGAAGGTGATGGAGCTAAGCTGAAACAACCGAATGAAGAAGGTGATGGAGCTAAGCTGAAACAACCGAATGAAGAAGGTGATGGAGCTAAGCTGAAACAACCGAATGCAGAAGGTGGAGCTAAGCTGAAACAACCGAATGCAGAAGGTGGAGCAAATGCAGGACAACCGAATGCAGAAGGTGGAGCAAATGCAGGACAACCGAATGCAGAAGGTGGAGCAAATGCAGGACAACCGAATGCAGAAGGTGGAGCAAATGCAGGACAACCGAATGCAGAAGGTGGAGCAAATGCAGGACAACCGAATGCAGAAGGTGGAGCAAATGCAGGACAACCGAATGCAGAAGGTGGAGCAAATGCACGACAACCGAATGCAGAAGGTGGAGCAAATGCACGACAACCGAATGCAGAAGGTGGAGCAAATGCACGACAACCTAATGCAGAAGGTGGAGCAAATGCAGGACAACCGAATGCAGAAGGTGGAGCAAATGCAGGACAACCGAATGCAGAAGGTGGAGCAAATGCACGACAGCCACAGGCAGAAGGTGGTGGAGCAAATGCACGACAGCCACAGGCAGAAGGTGGTGGAGCAAATGCACGACAAGGAGGAAATGAGGGGAATAAACAAGCAGGAAAAGGACAGGGACAAAACAATCAGGGTGCGAATGCCCCAAATGAAAAAGTTGTAAATGATTACCTACAGAAAATTAGATCTAGCGTTACCACCGAGTGGACTCCATGCAGTGTAACCTGTGGAAATGGTGTAAGAATTAGAAGAAGAGCTCATGCAGATAAGAAAAAGGCAGAGGACCTTACTATGGATGACCTTGAAGTGGAAGCTTGTGTAATGGATAAGTGTGCTGGCATATTTAACGTTGTGAGTAATTCATTAGGGTTAGTAATATTGTTAGTCCTAGCATTATTCAATTAA---------------------------------------------------------------------------------------------------------------------------------------------------------------------------------------------------------------------------------------------------------------------------

>KF861738 Human Peninsular Malaysia

ATGAGGAACTTCATTCTCTTGGCCGTCTCCTCCATCCTGCTGGTGGACTTGCTCCCCACACACTTCGAACATAATGTAGATCTCTCCAGGGCCATAAATGTAAATGGAGTAAGCTTCAATAATGTAGACACCAGTTCACTTGGCGCAGCACAGGTAAGACAAAGTGCTAGCCGAGGCAGAGGACTTGGTGAGAAGCGAAAAGAAGGAGCTGATAAAGAAAAGAAAAAAGAAAAAGAAGAAGAACCAAAGAAGCCAAATGAAAATAAGCTGAAACAACCGAATCCAGACCAACCACAAGCACAGGGTGATGGAGCAAATGCAGGACAACCACAAGCACAAGGAGATGGAGCAAATGCAGGACAACCACAAGCACAAGGAGATGGAGCAAATGCAGGACAACCACAAGCACAAGGAGATGGAGCAAATGCAGGACAACCACAAGCACAAGGAGATGGAGCAAATGCAGGACAACCACAAGCACAAGGAGATGGAGCAAATGCAGGACAACCACAAGCACAGGGTGATGGAGCAAATGCAGGACAACCACAAGCACAAGGAGATGGAGCAAATGCAGGACAACCACAAGCACAGGGTGATGGAGCAAATGCAGGACAACCACAAGCACAGGGTGATGGAGCAAATGCAGGACAGCCACAAGCACAGGGTGATGGAGCAAATGCAGGACAACCACAAGCACAGGGTGATAGGGCGAATGCAGGACAACCACAAGCACAAGGAGATGGGGCAAATGTACCACGACAAGGAAGAAACGGGGGAGGTGCACCAGCAGGAGGAAATGAGGGGAATAAACAAGCAGGAAAAGGACAGGGACAAAACAATCAGGGTGCGAATGCCCCAAATGAAAAAGTTGTGAATGATTACCTACACAAAATTAGATCTAGCGTTACCACCGAGTGGACTCCATGCAGTGTAACCTGTGGAAATGGTGTAAGAATTAGAAGAAGACAGAATGCTGGTAATAAAAAGGCAGAGGACCTTACTATGGATGACCTTGAGGCGGAAGCTTGTGTAATGGATAAGTGCGCTGGCATATTTAACGTTGTGAGTAATTCATTAGGGTTAGTCATATTGTTAGTCCTAGCATTATTCAATTAA---------------------------------------------------------------------------------------------------------------------------------------------------------------------------------------------------------------------------------------------------------------------------------------------------------------------------------------------------------------------------------

>KF861737 Human Peninsular Malaysia

ATGAGGAACTTCATTCTCTTGGCCGTCTCCTCCATCCTGCTGGTGGACTTGCTCCCCACACACTTCGAACATAATGTAGATCTCTCCAGGGCCATAAATGTAAATGGAGTAAGCTTCAATAATGTAGACACCAGTTCACTTGGCGCAGCACAGGTAAGACAAAGTGCTAGCCGAGGCAGAGGACTTGGTGAGAAGCGAAAAGAAGGAGCTGATAAAGAAAAGAAAAAAGAAAAAGAAGAAGAACCAAAGAAGCCAAATGAAAATAAGCTGAAACAACCGAATCCAGACCAACCACAAGCACAGGGTAATGGAGCAAATGCAGGACAACCACAAGCACAAGGAGATGGAGCAAATGCAGGACAACCACAAGCACAAGGAGATGGAGCAAATGCAGGACAACCACAAGCACAAGGAGATGGAGCAAATGCAGGACAACCACAAGCACAAGGAGATGGAGCAAATGCAGGACAACCACAAGCACAAGGAGATGGAGCAAATGCAGGACAACCACAAGCACAAGGAGATGGAGCAAATGCAGGACAACCACAAGCACAAGGAGATGGAGCAAATGCAGGACAACCACAAGCACAGGGTGATGGAGCAAATGCAGGACAACCACAAGCACAAGGAGATGGAGCAAATGCAGGACAACCACAAGCACAGGGTGATGGAGCAAATGCAGGACAACCACAAGCACAGGGTGATGGAGCAAATGCAGGACAACCACAAGCACAGGGTGATGGAGCAAATGCAGGACAACCACAAGCACAGGGTGATAGGGCGAATGCAGGACAACCACAAGCACAAGGAGATGGGGCAAATGTACCACGACAAGGAAGAAACGGGGGAGGTGCACCAGCAGGAGGAAATGAGGGGAATAAACAAGCAGGAAAAGGACAGGGACAAAACAATCAGGGTGCGAATGCCCCAAATGAAAAAGTTGTGAATGATTACCTACACAAAATTAGATCTAGCGTTACCACCGAGTGGACTCCATGCAGTGTAACCTGTGGAAATGGTGTAAGAATTAGAAGAAAAGCTCATGCAGGTAATAAAAAGGCAGAGGACCTTACTATGGATGACCTTGAGGTGGAAGCTTGTGTAATGGATAAGTGCGCTGGCATATTTAACGTTGTGAGTAATTCATTAGGGTTAGTCATATTGTTAGTCCTAGCATTATTCAATTAA---------------------------------------------------------------------------------------------------------------------------------------------------------------------------------------------------------------------------------------------------------------------------------------------------------

>KF861736 Human Peninsular Malaysia

ATGAGGAACTTCATTCTCTTGGCCGTCTCCTCCATCCTGCTGGTGGACTTGCTCCCCACACACTTCGAACATAATGTAGATCTCTCCAGGGCCATAAATGTAAATGGAGTAAGCTTCAATAATGTAGACACCAGTTCACTTGGCGCAGCACAGGTAAGACAAAGTGCTAGCCGAGGCAGAGGACTTGGTGAGAAGCGAAAAGAAGGAGCTGATAAAGAAAAGAAAAAAGAAAAAGAAGAAGAACCAAAGAAGCCAAATGAAAATAAGCTGAAACAACCGAATCCAGACCAACCACAAGCACAGGGTGATGGAGCAAATGCAGGACAACCACAAGCACAAGGAGATGGAGCAAATGCAGGACAACCACAAGCACAAGGAGATGGAGCAAATGCAGGACAACCACAAGCACAAGGAGATGGAGCAAATGCAGGACAACCACAAGCACAAGGAGATGGAGCAAATGCAGGACAACCACAGGCACAAGGAGATGGAGCAAATGCAGGACAACCACAAGCACAGGGTGATGGAGCAAATGCAGGACAACCACAAGCACAAGGAGATGGAGCAAATGCAGGACAACCACAAGCACAGGGTGATGGAGCAAATGCAGGACAACCACAAGCACAGGGTGATGGAGCAAATGCAGGACAACCACAAGCACAGGGTGATGGAGCAAATGCAGGACAACCACAAGCACAGGGTGATAGGGCGAATGCAGGACAACCACAAGCACAAGGAGATGGGGCAAATGTACCACGACAAGGAAGAAACGGGGGAGGTGCACCAGCAGGAGGAAATGAGGGGAATAAACAAGCAGGAAAAGGACAGGGACAAAACAATCAGGGTGCGAATGCCCCAAATGAAAAAGTTGTGAATGATTACCTACACAAAATTAGATCTAGCGTTACCACCGAGTGGACTCCATGCAGTGTAACCTGTGGAAATGGTGTAAGAATTAGAAGAAAAGCTCATGCGGGTAATAAAAAGGCAGAGGACCTTACTATGGATGACCTTGAGGTGGAAGCTTGTGTAATGGATAAGTGCGCTGGCATATTTAACGTTGTGAGTAATTCATTAGGGTTAGTCATATTGTTAGTCCTAGCATTATTCAATTAA---------------------------------------------------------------------------------------------------------------------------------------------------------------------------------------------------------------------------------------------------------------------------------------------------------------------------------------------------------------------------------

>KF861735 Human Peninsular Malaysia

ATGAAGAACTTCATTCTCTTGGCCGTCTCCTCCATCCTGCTGGTGGACTTGCTCCCCACACACTTCGAACATAATGTAGATCTCTCCAGGGCCATAAATGTAAATGGAGTAAGCTTCAATAATGTAGACACCAGTTCACTTGGCGCAGCACAGGTGAGACAAAGTGCTAGCCGAGGCAGAGGACTTGGTGAGAAGCCAAAAGAAGGAGCTGATAAAGAAAAGAAAAAAGAAAAAGAAAAAGAAGAAGAACCAAAGAAGCCAAATGAAAATAAGCTGAAACAACCTAATGCAGAAGGTGATGGGGCAAATGCACGACAACCTAATGCAGAAGGTGATGGAGCAAATGCACGACAACCGAATGCAGAAGGTGATGGAGCAAATGCACGACAACCTAATGCAGAAGGTGATGGAGCAAATGCACGACAACCTAATGCAGAAGGTGATGGAGCAAATGCACGACAACCTAATGCAGAAGGTGATGGAGCAAATGCACGACAACCTAATGCAGAAGGTAATGGAGCAAATGCACGACAACCGAATGCAGAAGGTGGTGGAGCAAATGCACGACAACCGAATGCAGAAGGTGATGGAGCAAATGCACGACAACCTAATGCAGAAGGTGATGGAGCAAATGCACGACAACCGAATGCAGAAGGTGGTGGAGCAAATGCACGACAGCCACAGGCAGAAGGTGATGGAGCAAATGCACGACAACCACAAGCACAAGGAGATGGAGGAAATGCACGACAAGGAGGAAACGGGGGAGGTGCACCAGCAGGAGGAAATGAGGGGAATAAACAAGCAGGAAAAGGACAGGGACAAAACAATCAGGGTGCGAATGCCCCAAATGAAAAAGTTGTAAATGATTACCTACAGAAAATTAGATCTAGCGTTACCACCGAGTGGACTCCATGCAGTGTAACCTGTGGAAATGGTGTAAGAATTAGAAGAAGAGCTCATGCAGATAAGAAAAAGGCAGAGGACCTTACTATGGATGACCTTGAAGTGGAAGCTTGTGTAATGGATAAGTGTGCTGGCATATTTAACGTTGTGAGTAATTCATTAGGGTTCGTCATATTGTTAGTCCTAGCATTATTCAATTAA---------------------------------------------------------------------------------------------------------------------------------------------------------------------------------------------------------------------------------------------------------------------------------------------------------------------------------------------------------------------------------------------

>KF861734 Human Peninsular Malaysia

ATGAAGAACTTCATTCTCTTGGCCGTCTCCTCCATCCTGCTGGTGGACTTGCTCCCCACATACTTCGAACATAATGTAGATCTCTCCAGGGCCATAAATGTAAATGGAGTAAGCTTCAATAGTGTAGACACCAGTTCACTTGGCGCAGCACAGGTAAGACAAAGTGCTAGCCGGGGCAGAGGACTTGGTGAGAAGCCAAAAGAAGGAGCTGATAAAGAAAAGAAAAAAGAAAAAGAAAAAGAAGAAGAACCAAAGAAGCCAAATGAAAATAAGCTGAAACAACCACAAGGAAATGGGGGGGCAGGTCAAGCACAACCAGAAGGAAATGGGGGGGCAGGTCAAGCACAACCGGAAGGAAATGGGGGGGCAGGTCAAGCACAACCAGAAGGAAATGGGGGGGCAGGTCAAGCACAACCGGAAGGAAATGGGGGGGCAGGTCAAGCACAACCGGAAGGAAATGGGGGGGCAGGTCAAGCACAACCGGAAGGAAATGGGGGGGCAGGTCAAGCACAACCGGAAGGAAATGGGGGGGCAGGTCAAGCACAACCGGAAGGAAATGGGGGGGCAGGTCAAGCACAACCGGAAGGAAATGGGGGGGCAGGTCAAGCACAACCACAAGGAAATGGGGGGGCAGGTCAAGCACAACCACAAGGAAATGGGGGGGCAGGTCAAGCACAACCACAAGGAAATGGGGGGGCAGGTCAAGCACAACCGGAAGGAAATAGGGAAGCTCCAGCACAACCACAAGGAAATGGGGGGGCAGGTCAAGCACAACCACAAAAAAACGAAGGAGGAAACGCAGGAGCACGGAAAGGACAGGGACAAAACAATCAGGGTGCGAATGCCCCAAATGAAAAAGTTGTAAATGATTACCTACAGAAAATTAGATCTAGCGTTACCACCGAGTGGACTCCATGCAGTGTAACCTGTGGAAATGGTGTAAGAATTAGAAGAAGAGCTCATGCAGATAAGAAAAAGGCAGAGGACCTTACTATGGATGACCTTGAAGTGGAAGCTTGTGTAATGGATAAGTGTGCTGGCATATTTAACGTTGTGAGTAATTCATTAGGGTTAGTCATATTGTTAGTCCTAGCATTATTCAATTAA------------------------------------------------------------------------------------------------------------------------------------------------------------------------------------------------------------------------------------------------------------------------------------------------------------------------------------------------------------------------------------------

>KF861733 Human Peninsular Malaysia

ATGAAGAACTTCATTCTCTTGGCCGTCTCCTCCATCCTGCTGGTGGACTTGCTCCCCACACACTTCGAACATAATGTAGATCTCTCCAGGGCCATAAATGTAAATGGAGTAAGCTTCAATAATGTAGACACCAGTTCACTTGGCGCAGCACAGGTGAGACAAAGTGCTAGCCGAGGCAGAGGACTTGGTGAGAAGCCAAAAGAAGGAGCTGATAAAGAAAAGAAAAAAGAAAAAGAAAAAGAAGAAGAACCAAAGAAGCCAAATGAAAATAAGCTGAAACAACCTAATGCAGAAGGTGATGGAGCAAATGCACGACAACCTAATGCAGAAGGTGATGGAGCAAATGCACGACAACCGAATGCAGAAGGTGATGGAGCAAATGCACGACAACCTAATGCAGAAGGTGATGGAGCAAATGCACGACAACCGAATGCAGAAGGTGATGGAGCAAATGCACGACAACCTAATGCAGAAGGTGATGGAGCAAATGCACGACAACCGAATGCAGAAGGTGGTGGAGCAAATGCACGACAACCACAGGCAGAAGGTGATGGAGCAAATGCACGACAACCACAAGCACAAGGAGATGGAGGAAATGCACGACAAGGAGGAAACGGGGGAGGTGCACCAGCAGGAGGAAATGAGGGGAATAAACAAGCAGGAAAAGGACAGGGACAAAACAATCAGGGTGCGAATGCCCCAAATGAAAAAGTTGTAAATGATTACCTACAGAAAATTAGATCTAGCGTTACCACCGAGTGGACTCCATGCAGTGTAATCTGTGGAAATGGTGTAAGAATTAGAAGAAGAGCTCATGCAGATAAGAAAAAGGCAGAGGACCTTACTATGGATGACCTTGAAGTGGAAGCTTGTGTAATGGATAAGTGTGCTGGCATATTTAACGTTGTGAGTAATTCATTAGGGTTCGTCATATTGTTAGTCCTAGCATTATTCAATTAA---------------------------------------------------------------------------------------------------------------------------------------------------------------------------------------------------------------------------------------------------------------------------------------------------------------------------------------------------------------------------------------------------------------------------------------------------------------------------------------------------------------------------------------------

>KF861732 Human Peninsular Malaysia

ATGAAGAACTTCATTCTCTTGGCCGTCTCCTCCATCCTGCTGGTGGACTTGCTCCCCACACACTTCGAACATAATGTAGATCTCTCCAGGGCCATAAATGTAAATGGAGTAAGCTTCAATAATGTAGACACCAGTTCACTTGGCGCAGCACAGGTAAGACAAAGTGCTAGCCGAGGCAGAGGACTTGGTGAGAAGCCAAAAGAAGGAGCTGATAAAGAAAAGAAAAAAGAAAAAGAAAAAGAAAAAGAAGAAGAACCAAAGAAGCCAAATGAAAATAAGCTGAAACAACCGGAACAACCAGCAGCAGGAGCAGGGGGCGAACAACCAGCAGCAGGAGCAGGAGGCGAACAACCAGCAGCAGGAGCAGGAGGCGAACAACCAGCAGCAGGAGCAGGAGGCGAACAACCAGCAGCAGGAGCAAGAGGCGAACAACCAGCAGCAGGAGCAGGAGGCGAACAACCAGCAGCAGGAGCAAGAGGCGAACAACCAGCAGCAGGAGCAGGAGGCGAACAACCAGCAGCAGGAGCAGGAGGCGAACAACCAGCAGCAGGAGCAGGAGGCGAACAACCAGCAGCAGGAGCAAGAGGCGAACAACCAGCAGCAGGAGCAGGAGGCGAACAACCAGCACCAGCACCAAGGAGGGAACAACCAGCAGCAGGAGCAGGGGGCGAACAACCAGCACCAGCACCAAGGAGGGAACAACCAGCACCAGGAGCAGGTGCGGGAGATGGAGCACGAGAAGGAAACGCAGGGGCAGGTAAAGGACAGGGACAAAACAATCAGGGTGCGAATGTCCCAAATGAAAAAGTTGTGAATGATTACCTACACAAAATTAGATCTAGCGTTACCACCGAGTGGACTCCATGCAGTGTAACCTGTGGAAATGGTGTAAGAATTAGAAGAAAAGGTCATGCAGGTAATAAAAAGGCAGAGGACCTTACTATGGATGACCTTGAGGTGGAAGCTTGTGTAATGGATAAGTGCGCTGGCATATTTAACGTTGTGAGTAATTCATTAGGCTTAGTCATATTGTTAGTCCTAGCATTATTCAATTAA---------------------------------------------------------------------------------------------------------------------------------------------------------------------------------------------------------------------------------------------------------------------------------------------------------------------------------------------------------------------------------------------------------------------------------------------

>KF861731 Human Peninsular Malaysia

ATGAAGAACTTCATTCTCTTGGCCGTCTCCTCCATCCTGCTGGTGGACTTGCTCCCCACACACTTCGAACATAATGTAGATCTCTCCAGGGCCATAAATGTAAATGGAGTAAGCTTCAATAATGTAGACACCAGTTCACTTGGCGCAGCACAGGTAAGACAAAGTGCTAGCCGAGGCAGAGGACTTGGTGAGAAGCCAAAAGAAGGAGCTGATAAAGAAAAGAAAAAAGAAAAAGAAAAAGAAAAAGAAGAAGAACCAAAGAAGCCAAATGAAAATAAGCTGAAACAACCGGAACAACCAGCAGCAGGAGCAGGGGGCGAACAACCAGCAGCAGGAGCAGGAGGCGAACAACCAGCAGCAGGAGCAGGAGGCGAACAACCAGCAGCAGGAGCAGGAGGCGAACAACCAGCAGCAGGAGCAAGAGGCGAACAACCAGCAGCAGGAGCAGGAGGCGAACAACCAGCAGCAGGAGCAAGAGGCGAACAACCAGCAGCAGGAGCAGGAGGCGAACAACCAGCAGCAGGAGCAGGAGGCGAACAACCAGCAGCAGGAGCAGGAGGCGAACAACCAGCAGCAGGAGGCGAACAACCAGCAGCAGGAGCAGGAGGCGAACAACCAGCAGCAGGAGCAAGAGGCGAACAACCAGCAGCAGGAGCAGGAGGCGAACAACCAGCACCAGCACCAAGGAGGGAACAACCAGCAGCAGGAGCAGGGGGCGAACAACCAGCACCAGCACCAAGGAGGGAACAACCAGCACCAGGAGCAGGTGCGGGAGATGGAGCACGAGGAGGAAACGCAGGGGCAGGTAAAGGACAGGGACAAAACAATCAGGGTGCGAATGTCCCAAATGAAAAAGTTGTGAATGATTACCTACACAAAATTAGATCTAGCGTTACCACCGAGTGGACTCCATGCAGTGTAACCTGTGGAAATGGTGTAAGAATTAGAAGAAAAGGTCATGCAGGTAATAAAAAGGCAGAGGACCTTACTATGGATGACCTTGAGGTGGAAGCTTGTGTAATGGATAAGTGCGCTGGCATATTTAACGTTGTGAGTAATTCATTAGGCTTAGTCATATTGTTAGTCCTAGCATTATTCAATTAA---------------------------------------------------------------------------------------------------------------------------------------------------------------------------------------------------------------------------------------------------------------------------------------------------------------------------------------------------------------------------------------------

>KF861730 Human Peninsular Malaysia

ATGAAGAACTTCATTCTCTTGGCCGTCTCCTCCATCCTGCTGGTGGACTTGCTCCCCACACACTTCGAACATAATGTAGATCTCTCCAGGGCCATAAATGTAAATGGAGTAGGCTTCAATAATGTAGACACCAGTTCACTTGGCGCATCACAGGTGAGACAAAGTGCTAGCCGAGGCAGAGGACTTGGTGAGAAGCCAAAAGAAGGAGCTGATAAAGAAAAGAAAAAAGAAAAAGGAAAAGAAAAAGAAGAAGAACCAAAGAAGCCAAATGAAAATAAGCTGAAACAACCGAATGAAGGACAACCACAAGCACAGGGTGATGGAGCAAATGCAGGACAACCACAAGCACAAGGAGATGGAGCAAATGCAGGACAACCACAAGCACAAGGAGATGGAGCAAATGCAGGACAACCACAAGCACAGGGTGATGGAGCAAATGCAGGACAACCACAAGCACAAGGAGATGGAGCAAATGCAGGACAACCACAAGCACAGGGTGATGGAGCAAATGCAGGGCAACCACAAGCACAGGGTGATGGAGCAAATGCAGGACAACCACAAGCACAAGGAGATGGAGCAAATGCAGGACAACCACAAGCACAAGGAGATGGAGCAAATGCAGGACAACCACAAGCACAGGGTGATGGAGCAAATGCAGGACAACCACAAGCACAGGGTGATAGGGCGAATGCAGGACAACCACAAGCACAAGGAGATGGGGCAAATGTACCACGACAAGGAAGAAACGGGGGAGGTGCACCAGCAGGAGGAAATGAGGGGAATAAACAAGCAGGAAAAGGACAGGGACAAAACAATCAGGGTGCGAATGCCCCAAATGAAAAAGTTGTGAATGATTACCTACACAAAATTAGATCTAGCGTTACCACCGAGTGGACTCCATGCAGTGTAACCTGTGGAAATGGTGTAAGAATTAGAAGAAAAGCTCATGCAGGTAATAAAAAGGCAGAGGACCTTACTATGGATGACCTTGAGGTGGAAGCTTGTGTAATGGATAAGTGCGCTGGCATATTTAACGTTGTGAGTAATTCATTAGGCTTAGTCATATTGTTAGTCCTAGCATTATTCAATTAA---------------------------------------------------------------------------------------------------------------------------------------------------------------------------------------------------------------------------------------------------------------------------------------------------------------------------------------------------------------------------------------------------------

>KF861729 Human Peninsular Malaysia

ATGAAGAACTTCATTCTCTTGGCCGTCTCCTCCATCCTGCTGGTGGACTTGCTCCCCACACACTTCGAACATAATGTAGATCTCTCCAGGGCCATAAATGTAAATGGAGTAAGCTTCAATAATGTAGACACCAGTTCACTTGGCGCATCACAGGTGAGACAAAGTGCTAGCCGAGGCAGAGGACTTGGTGAGAAGCCAAAAGAAGGAGCTGATAAAGAAAAGAAAAAAGAAAAAGGAAAAGAAAAAGAAGAAGAACCAAAGAAGCCAAATGAAAATAAGCTGAAACAACCGAATGAAGGACAACCACAAGCACAGGGTGATGGAGCAAATGCAGGACAACCACAAGCACAAGGAGATGGAGCAAATGCAGGACAACCACAAGCACAAGGAGATGGAGCAAATGCAGGACAACCACAAGCACAGGGTGATGGAGCAAATGCAGGACAACCACAAGCACAAGGAGATGGAGCAAATGCAGGACAACCACAAGCACAGGGTGATGGAGCAAATGCAGGGCAACCACAAGCACAGGGTGATGGAGCAAATGCAGGACAACCACAAGCACAAGGAGATGGAGCAAATGCAGGACAACCACAAGCACAAGGAGATGGAGCAAATGCAGGACAACCACAAGCACAGGGTGATGGAGCAAATGCAGGACAACCACAAGCACAGGGTGATAGGGCGAATGCAGGACAACCACAAGCACAAGGAGATGGGGCAAATGTACCACGACAAGGAAGAAACGGGGGAGGTGCACCAGCAGGAGGAAATGAGGGGAATAAACAAGCAGGAAAAGGACAGGGACAAAACAATCAGGGTGCGAATGCCCCAAATGAAAAAGTTGTGAATGATTACCTACACAAAATTAGATCTAGCGTTACCACCGAGTGGACTCCATGCAGTGTAACCTGTGGAAATGGTGTAAGAATTAGAAGAAGACAGAATGCTGGTAATAAAAAGGCAGAGGACCTTACTATGGATGACCTTGAGGTGGAAGCTTGTGTAATGGATAAGTGCGCTGGCATATTTAACGTTGTGAGTAATTCATTAGGGTTAGTCATATTGTTAGTCCTAGCATTATTCAATTAA---------------------------------------------------------------------------------------------------------------------------------------------------------------------------------------------------------------------------------------------------------------------------------------------------------------------------------------------------------------------------------------------------------

>KF861728 Human Peninsular Malaysia

ATGAAGAACTTCATTCTCTTGGCCGTCTCCTCCATCCTGCTGGTGGACTTGCTCCCCACACACTTCGAACATAATGTAGATCTCTCCAGGGCCATAAATGTAAATGGAGTAAGCTTCAATAATGTAGACACCAGTTCACTTGGCGCAGCACAGGTAAGACAAAGTGCTAGCCGAGGCAGAGGACTTGGTGAGAAGCCAAAAGAAGGAGATGATAAAGAAAAGAAAAAAGAAAAAGAAAAAGAAGAAGAACCAAAGAAGCTAAATGAAAATAAGCCGAAACAACCGAATGTAGAAGGTGATGGAGCTAAGCTGAAACAACCGAATGAAGAAGGTGATGGAGCTAAGCTGAAACAACCGAATGAAGAAGGTGATGGAGCTAAGCTGAAACAACCGAATGCAGAAGGTGGAGCTAAGCTGAAACAACCGAATGCAGAAGGTGGAGCAAATGCAGGACAACCGAATGCAGAAGGTGGAGCAAATGCAGGACAACCGAATGCAGAAGGTGGAGCAAATGCAGGACAACCGAATGCAGAAGGTGGAGCAAATGCAGGACAACCGAATGCAGAAGGTGGAGCAAATGCAGGACAACCGAATGCAGAAGGTGGAGCAAATGCAGGACAACCGAATGCAGAAGGTGGAGCAAATGCAGGACAACCGAATGCAGAAGGTGGAGCAAATGCACGACAACCGAATGCAGAAGGTGGAGCAAATGCACGACAACCGAATGCAGAAGGTGGAGCAAATGCACGACAACCTAATGCAGAAGGTGGAGCAAATGCAGGACAACCGAATGCAGAAGGTGGAGCAAATGCAGGACAACCGAATGCAGAAGGTGGAGCAAATGCACGACAGCCACAGGCAGAAGGTGGTGGAGCAAATGCACGACAGCCACAGGCAGAAGGTGGTGGAGCAAATGCACGACAAGGAGGAAATGAGGGGAATAAACAAGCAGGAAAAGGACAGGGACAAAACAATCAGGGTGCGAATGCCCCAAATGAAAAAGTTGTAAATGATTACCTACAGAAAATTAGATCTAGCGTTACCACCGAGTGGACTCCATGCAGTGTAACCTGTGGAAATGGTGTAAGAATTAGAAGAAGAGCTCATGCAGATAAGAAAAAGGCAGAGGACCTTACTATGGATGACCTTGAAGTGGAAGCTTGTGTAATGGATAAGTGTGCTGGCATATTTAACGTTGTGAGTAATTCATTAGGGTTAGTAATATTGTTAGTCCTAGCATTATTCAATTAA------------------------------------------------------------------------------------------------------------------------------------------------------------------------------------------------------------------------------------------

>KF861727 Human Peninsular Malaysia

ATGAAGAACTTCATTCTCTTGGCCGTCTCCTCCATCCTGCTGGTGGACTTGCTCCCCACACACTTCGAACATAATGTAGATCTCTCCAGGGCCATAAATGTAAATGGAGTAAGCTTCAATAATGTAGACACCAGTTCACTTGGCGCAGCACAGGTAAGACAAAGTGCTAGCCGAGGCAGAGGACTTGGTGAGAAGCCAAAAGAAGGAGATGATAAAGAAAAGAAAAAAGAAAAAGAAAAAGAAGAAGAACCAAAGAAGCTAAATGAAAATAAGCCGAAACAACCGAATGTAGAAGGTGATGGAGCTAAGCTGAAACAACCGAATGAAGAAGGTGATGGAGCTAAGCTGAAACAACCGAATGAAGAAGGTGATGGAGCCAAGCTGAAACAACCGAATGCAGAAGGTGGAGCTAAGCTGAAACAACCGAATGCAGAAGGTGGAGCAAATGCAGGACAACCGAATGCAGAAGGTGGAGCAAATGCAGGACAACCGAATGCAGAAGGTGGAGCAAATGCAGGACAACCGAATGCAGAAGGTGGAGCAAATGCAGGACAACCGAATGCAGAAGGTGGAGCAAATGCAGGACAACCGAATGCAGAAGGTGGAGCAAATGCAGGACAACCGAATGCAGAAGGTGGAGCAAATGCAGGACAACCGAATGCAGAAGGTGGAGCAAATGCACGACAACCGAATGCAGAAGGTGGAGCAAATGCACGACAACCGAATGCAGAAGGTGGAGCAAATGCACGACAACCTAATGCAGAAGGTGGAGCAAATGCAGGACAACCGAATGCAGAAGGTGGAGCAAATGCAGGACAACCGAATGCAGAAGGTGGAGCAAATGCACGACAGCCACAGGCAGAAGGTGGTGGAGCAAATGCACGACAGCCACAGGCAGAAGGTGGTGGAGCAAATGCACGACAAGGAGGAAATGAGGGGAATAAACAAGCAGGAAAAGGACAGGGACAAAACAATCAGGGTGCGAATGCCCCAAATGAAAAAGTTGTAAATGATTACCTACAGAAAATTAGATCTAGCGTTACCACCGAGTGGACTCCATGCAGTGTAACCTGTGGGAATGGTGTAAGAATTAGAAGAAGAGCTCATGCAGATAAGAAAAAGGCAGAGGACCTTACTATGGATGACCTTGAAGTGGAAGCTTGTGTAATGGATAAGTGTGCTGGCATATTTAACGTTGTGAGTAATTCATTAGGGTTAGTAATATTGTTAGTCCTAGCATTATTCAATTAA------------------------------------------------------------------------------------------------------------------------------------------------------------------------------------------------------------------------------------------

>KF861726 Human Peninsular Malaysia

ATGAAGAACTTCATTCTCTTGGCCGTCTCCTCCATCCTGCTGGTGGACTTGCTCCCCACACACTTCGAACATAATGTAGATCTCTCCAGGGCCATAAATGTAAATGGAGTAAGCTTCAATAATGTAGACACCAGTTCACTTGGCGCAGCACAGGTAAGACAAAGTGCTAGCCGAGGCAGAGGACTTGGTGAGAAGCCAAAAGAAGGAGATGATAAAGAAAAGAAAAAAGAAAAAGAAAAAGAAGAAGAACCAAAGAAGCTAAATGAAAATAAGCCGAAACAACCGAATGTAGAAGGTGATGGAGCTAAGCTGAAACAACCGAATGAAGAAGGTGATGGAGCTAAGCTGAAACAACCGAATGAAGAAGGTGATGGAGCTAAGCTGAAACAACCGAATGCAGAAGGTGGAGCTAAGCTGGAACAACCGAATGCAGAAGGTGGAGCAAATGCAGGACAACCGAATGCAGAAGGTGGAGCAAATGCAGGACAACCGAATGCAGAAGGTGGAGCAAATGCAGGACAACCGAATGCAGAAGGTGGAGCAAATGCAGGACAACCGAATGCAGAAGGTGGAGCAAATGCAGGACAACCGAATGCAGAAGGTGGAGCAAATGCAGGACAACCGAATGCAGAAGGTGGAGCAAATGCAGGACAACCGAATGCAGAAGGTGGAGCAAATGCACGACAACCGAATGCAGAAGGTGGAGCAAATGCACGACAACCGAATGCAGAAGGTGGAGCAAATGCACGACAACCTAATGCAGAAGGTGGAGCAAATGCAGGACAACCGAATGCAGAAGGTGGAGCAAATGCAGGACAACCGAATGCAGAAGGTGGAGCAAATGCACGACAGCCACAGGCAGAAGGTGGTGGAGCAAATGCACGACAGCCACAGGCAGAAGGTGGTGGAGCAAATGCACGACAAGGAGGAAATGAGGGGAATAAACAAGCAGGAAAAGGACAGGGACAAAACAATCAGGGTGCGAATGCCCCAAATGAAAAAGTTGTAAATGATTACCTACAGAAAATTAGATCTAGCGTTACCACCGAGTGGACTCCATGCAGTGTAACCTGTGGAAATGGTGTAAGAATTAGAAGAAGAGCTCATGCAGATAAGAAAAAGGCAGAGGACCTTACTATGGATGACCTTGAAGTGGAAGCTTGTGTAATGGATAAGTGTGCTGGCATATTTAACGTTGTGAGTAATTCATTAGGGTTAGTAATATTGTTAGTCCTAGCATTATTCAATTAA------------------------------------------------------------------------------------------------------------------------------------------------------------------------------------------------------------------------------------------

>KF861725 Human Peninsular Malaysia

ATGAAGAACTTCATTCTCTTGGCCGTCTCCTCCATCCTGCTGGTGGACTTGCTCCCCACACACTTCGAACATAATGTAGATCTCTCCAGGGCCATAAATGTAAATGGAGTAAGCTTCAATAATGTAGACACCAGTTCACTTGGCGCAGCACAGGTGAGACAAAGTGCTAGCCGAGGCAGAGGACTTGGTGAGAAGCCAAAAGAAGGAGCTGATAAAGAAAAGAAAAAAGAAAAAGAAAAAGAAGAAGAACCAAAGAAGCCAAATGAAAATAAGCTGAAACAACCGGAACAAGCAAGACCAGGAGCAGGAGGCGAGCAACCAGCACCAGGAGCAGGAGGCGAACAACCAGCACCAGGAGCAGGAGGCGAACAACCAGCACCAGGAGCAGGAGGCGAGCAACCAGCACCAGGAGCAGGAGGCGAGCAACCAGCACCAGGAGCAGGAGGCGAACAACCAGCACCAGGAGCAGGGGGCGAACAACCAGCACCAGGAGCAGGAGGCGAACAACCAGCACCAGGAGCAGGGGGCGGACAACCAGCACCAGGAGCAGGAGGCGAACAACCAGCACCAGGAGCAGGAGGCGAACAACCAGCACCAGCACCAAGGAGGGAACAACCAGCACCAGGACCAGGGGGCGAACAACCAGCACCAGCACCAAGGAGGGAACAACCAGCACCAGGAGCAGGTGCGGGAGATGGAGCACGAGGAGGAAACGCAGGGGCAGGTAAAGGACAGGGACAAAACAATCAGGGTGCGAATGTCCCAAATGAAAAAGTTGTGAATGATTACCTACACAAAATTAGATCTAGCGTTACCACCGAGTGGACTCCATGCAGTGTAACCTGTGGAAATGGTGTAAGAATTAGAAGAAAAGGTCATGCAGGTAATAAAAAGGCAGAGGACCTTACTATGGATGACCTTGAGGTGGAAGCTTGTGTAATGGATAAGTGCGCTGGCATATTTAACGTTGTGAGTAATTCATTAGGCTTAGTCATATTGTTAGTCCTAGCATTATTCAATTAA------------------------------------------------------------------------------------------------------------------------------------------------------------------------------------------------------------------------------------------------------------------------------------------------------------------------------------------------------------------------------------------------------------------------------------------------------------------------------

>KF861724 Human Peninsular Malaysia

ATGAAGAACTTCATTCTCTTGGCCGTCTCCTCCATCCTGCTGGTGGACTTGCTCCCCACACACTTCGAACATAATGTAGATCTCTCCAGGGCCATAAATGTAAATGGAGTAAGCTTCAATAATGTAGACACCAGTTCACTTGGCGCAGCACAGGTAAGACAAAGTGCTAGCCGAGGCAGAGGACTTGGTGAGAAGCCAAAAGAAGGAGATGATAAAGAAAAGAAAAAAGAAAAAGAAAAGGAAGAAGAACCAAAGAAGCTAAATGAAAATAAGCCGAAACAACCGAATGTAGAAGGTGATGGAGCTAAGCTGAAACAACCGAATGAAGAAGGTGATGGAGCTAAGCTGAAACGACCGAATGAAGAAGGTGATGGAGCTAAGCTGAAACAACCGAATGCAGAAGGTGGAGCTAAGCCGAAACAACCGAATGCAGAAGGTGGAGCAAATGCAGGACAACCGAATGCAGAAGGTGGAGCAAATGCAGGACAACCGAATGCAGAAGGTGGAGCAAATGCAGGACAACCGAATGCAGAAGGTGGAGCAAATGCAGGACAACCGAATGCAGAAGGTGGAGCAAATGCAGGACAACCGAATGCAGAAGGTGGAGCAAATGCAGGACAACCGAATGCAGAAGGTGGAGCAAATGCACGACAACCGAATGCAGAAGGTGGAGCAAATGCACGACAACCGAATGCAGAAGGTGGAGCAAATGCACGACAACCTAATGCAGAAGGTGGAGCAAATGCAGGACAACCGAATGCAGAAGGTGGAGCAAATGCAGGACAACCGAATGCAGAAGGTGGAGCAAATGCACGACAGCCACAGGCAGAAGGTGGTGGAGCAAATGCACGACGGCCACAGGCAGAAGGTGGTGGAGCAAATGCACGACAAGGAGGAAATGAGGGGAATAAACAAGCAGGAAAAGGACAGGGACAAAACAATCAGGGTGCGAATGCCCCAAATGAAAAAGTTGTAAATGATTACCTACAGAAAATTAGATCTAGCGTTACCACCGAGTGGACTCCATGCAGTGTAACCTGTGGAAATGGTGTAAGAATTAGAAGAAGAGCTCATGCAGATAAGAAAAAGGCAGAGGACCTTACTATGGATGACCTTGAAGTGGAAGCTTGTGTAATGGATAAGTGTGCTGGCATATTTAACGTTGTGAGTAACTCATTAGGGTTAGTAATATTGTTAGCCCTAGCATTATTCAATTAA---------------------------------------------------------------------------------------------------------------------------------------------------------------------------------------------------------------------------------------------------------------------------

>KF861723 Human Peninsular Malaysia

ATGAAGAACTTCATTCTCTTGGCCGTCTCCTCCATCCTGCTGGTGGACTTGCTCCCCACACACTTCGAACATAATGTAGATCTCTCCAGGGCCATAAATGTAAATGGAGTAAGCTTCAATAATGTAGACACCAGTTCACTTGGCGCAGCACAGGTAAGACAAAGTGCTAGCCGAGGCAGAGGACTTGGTGAGAAGCCAAAAGAAGGAGATGATAAAGAAAAGAAAAAAGAAAAAGAAAAAGAAGAAGAACCAAAGAAGCTAAATGAAAATAAGCCGAAACAACCGAATGTAGAAGGTGATGGAGCTAAGCTGAAACAACCGAATGAAGAAGGTGATGGAGCTAAGCTGAAACAACCGAATGAAGAAGGTGATGGAGCTAAGCTGAAACAACCGAATGCAGAAGGTGGAGCTAAGCTGAAACAACCGAATGCAGAAGGTGGAGCAAATGCAGGACAACCGAATGCAGAAGGTGGAGCAAATGCAGGACAACCGAATGCAGAAGGTGGAGCAAATGCAGGACAACCGAATGCAGAAGGTGGAGCAAATGCAGGACAACCGAATGCAGAAGGTGGAGCAAATGCAGGACAACCGAATGCAGAAGGTGGAGCAAATGCAGGACAACCGAATGCAGAAGGTGGAGCAAATGCACGACAACCGAATGCAGAAGGTGGAGCAAATGCACGACAACCGAATGCAGAAGGTGGAGCAAATGCACGACAACCTAATGCAGAAGGTGGAGCAAATGCAGGACAACCGAATGCAGAAGGTGGAGCAAATGCAGGACAACCGAATGCAGAAGGTGGAGCAAATGCACGACAGCCACAGGCAGAAGGTGGTGGAGCAAATGCACGACAGCCACAGGCAGAAGGTGGTGGAGCAAATGCACGACAAGGAGGAAATGAGGGGAATAAACAAGCAGGAAAAGGACAGGGACAAAACAATCAGGGTGCGAATGCCCCAAATGAAAAAGTTGTAAATGATTACCTACAGAAAATTAGATCTAGCGTTACCACCGAGTGGACTCCATGCAGTGTAACCTGTGGAAATGGTGTAAGAATTAGAAGAAGAGCTCATGCAGATAAGAAAAAGGCAGAGGACCTTACTATGGATGACCTTGAAGTGGAAGCTTGTGTAATGGATAAGTGTGCTGGCATATTTAACGTTGTGAGTAATTCATTAGGGTTAGTAATATTGTTAGTCCTAGCATTATTCAATTAA---------------------------------------------------------------------------------------------------------------------------------------------------------------------------------------------------------------------------------------------------------------------------

>KF861722 Human Peninsular Malaysia

ATGAAGAACTTCATTCTCTTGGCCGTCTCCTCCATCCTGCTGGTGGACTTGCTCCCCACACACTTCGAACATAATGTAGATCTCTCCAGGGCCATAAATGTAAATGGAGTAAGCTTCAATAATGTAGACACCAGTTCACTTGGCGCAGCACAGGTAAGACAAAGTGCTAGCCGAGGCAGAGGACTTGGTGAGAAGCCAAAAGAAGGAGCTGATAAAGAAAAGAAAAAAGAAAAAGAAGAAGAACCAAAGAAGCCAAATGGAAATAAGCTGAAACAACCAGCACCAGGGGGCGAACAACCGGCACCAGGGGGCGAACAACCAGCACCAGGGGGCGAACAACCAGCACCAGGGGGCGAACAACCAGCACCAGGGGGCGAACAACCAGCACCAGGGGGCGAACAACCGGCACCAGGGGGCGAACAACCGGCACCAGGGGGCGAACAACCAGCACCAGGGGGCGAACAACCAGCACCAGGGGGCGAACAACCAGCACCAGGGGGCGAACAACCAGCACCAGGGGGCGAACAACCAGCACCGGGGGGTGAACAACCAGCACCAGCACCAAGGAGGGAACAACCAGCACCAGGGGGCGAACAACCAGCACCAGGGGGCGAACAACCAGCACCAAGGAGGGAACAACCAGCACCAGGAGCAGGTGCGGGAGATGGAGCACGAGGAGGAAACGCAGGGGCAGGGAAAGGACAGGGACAAAACAATCAGGGTGCGAGTGTCCCAAATGAAAAAGTTGTGAATGATTACCTACACAAAATTAGATCTAGCGTTACCACCGAGTGGACTCCATGCAGTGTAACCTGTGGAAATGGTGTAAGAATTAGAAGAAAAGCTCATGCAGATAAGAAAAAGGCAGAGGACCTTACTATGGATGACCTTGAAGTGGAAGCTTGTGTAATGGATAAGTGTGCTGGCATATTTAACGTTGTGAGTAATTCATTAGGGTTAGTCATATTGTTAGTCCTAGCATTATTCAATTAA------------------------------------------------------------------------------------------------------------------------------------------------------------------------------------------------------------------------------------------------------------------------------------------------------------------------------------------------------------------------------------------------------------------------------------------------------------------------------------------------------------

>KF861721 Human Peninsular Malaysia

ATGAAGAACTTCATTCTCTTGGCCGTCTCCTCCATCCTGCTGGTGGACTTGCTCCCCACACACTTCGAACATAATGTAGATCTCTCCAGGGCCATAAATGTAAATGGAGTAAGCTTCAATAATGTAGACACCAGTTCACTTGGCGCAGCACAGGTGAGACAAAGTGCTAGCCGAGGCAGAGGACTTGGTGAGAAGCCAAAAGAAGGAGCTGATAAAGAAAAGAAAAAAGAAAAAGGAAAAGAAAAAGAAGAAGAACCAAAGAAGCCAAATGAAAATAAGCTGAAACAACCGAATGAAGGACAACCACAAGCACAGGGTGATGGAGCAAATGCAGGACAACCACAAGCACAAGGAGATGGAGCAAATGCAGGACAACCACAAGCACAAGGAGATGGAGCAAATGCAGGACAACCACAAGCACAGGGTGATGGAGCAAATGCAGGACAACCACAAGCACAGGGTGATGGAGCAAATGCAGGACAACCACAAGCACAAGGAGATGGAGCAAATGCAGGACAACCACAAGCACAGGGTGATGGAGCAAATGCAGGACAACCACAAGCACAGGGTGATAGGGCGAATGCAGGACAACCACAAGCACAAGGAGATGGGGCAAATGTACCACGACAAGGAAGAAACGGGGGAGGTGCACCAGCAGGAGGAAATGAGGGGAATAAACAAGCAGGAAAAGGACAGGGACAAAACAATCAGGGTGCGAATGCCCCAAATGAAAAAGTTGTGAATGATTACCTACACAAAATTAGATCTAGCGTTACCACCGAGTGGACTCCATGCAGTGTAACCTGTGGAAATGGTGTAAGAATTAGAAGAAAAGCTCATGCAGGTAATAAAAAGGCAGAGGACCTTACTGTGGATGACCTTGAGGTGGAAGCTTGTGTAATGGATAAGTGCGCTGGCATATTTAACGTTGTGAGTAATTCATTAGGCTTAGTCATATTGTTAGTCCTAGCATTATTCAATTAA---------------------------------------------------------------------------------------------------------------------------------------------------------------------------------------------------------------------------------------------------------------------------------------------------------------------------------------------------------------------------------------------------------------------------------------------------------------------------------------------------------------------

>KF861720 Human Peninsular Malaysia

ATGAAGAACTTCATTCTCTTGGCCGTCTCCTCCATCCTGCTGGTGGACTTGCTCCCCACACACTTCGAACATAATGTAGATCTCTCCAGGGCCATAAATGTAAATGGAGTAAGCTTCAATAATGTAGACACCAGTTCACTTGGCGCAGCACAGGTGAGACAAAGTGCTAGCCGAGGCAGAGGACTTGGTGAGAAGCCAAAAGAAGGAGCTGATAAAGAAAAGAAAAAAGAAAAAGGAAAAGAAAAAGAAGAAGAACCAAAGAAGCCAAATGAAAATAAGCTGAAACAACCGAATGAAGGACAACCACAAGCACAGGGTGATGGAGCAAATGCAGGACAACCACAAGCACAAGGAGATGGAGCAAATGCAGGACAACCACAAGCACAAGGAGATGGAGCAAATGCAGGACAACCACAAGCACAGGGTGATGGAGCAAATGCAGGACAACCACAAGCACAGGGTGATGGAGCAAATGCAGGACAACCACAAGCACAAGGAGATGGAGCAAATGCAGGACAACCACAAGCACAGGGTGATGGAGCAAATGCAGGACAACCACAAGCACAGGGTGATAGGGCGAATGCAGGACAACCACAAGCACAAGGAGATGGGGCAAATGTACCACGACAAGGAAGAAACGGGGGAGGTGCACCAGCAGGAGGAAATGAGGGGAATAAACAAGCAGGAAAAGGACAGGGACAAAACAATCAGGGTGCGAATGCCCCAAATGAAAAAGTTGTGAATGATTACCTACACAAAATTAGATCTAGCGTTACCACCGAGTGGACTCCATGCAGTGTAACCTGTGGAAATGGTGTAAGAATTAGAAGAAAAGCTCATGCAGGTAATAAAAAGGCAGAGGACCTTACTGTGGATGACCTTGAGGTGGAAGCTTGTGTAATGGATAAGTGCGCTGGCATATTTAACGTTGTGAGTAATTCATTAGGCTTAGTCATATTGTTAGTCCTAGCATTATTCAATTAA---------------------------------------------------------------------------------------------------------------------------------------------------------------------------------------------------------------------------------------------------------------------------------------------------------------------------------------------------------------------------------------------------------------------------------------------------------------------------------------------------------------------

>KF861719 Human Peninsular Malaysia

ATGAAGAACTTCATTCTCTTGGCCGTCTCCTCCATCCTGCTGGTGGACTTGCTCCCCACATACTTCGAACATAATGTAGATCTCTCCAGGGCCATAAATGTAAATGGAGTAAGCTTCAATAATGTAGACACCAGTTCACTTGGCGCAGCACAGGTAAGACAAAGTGCTAGCCGAGGCAGAGGACTTGGTGAGAAGCCAAAAGAAGGAGCTGATAAAGAAAAGAAAAAAGAAAAAGAAAAAGAAAAAGAAGAAGAACCAAAGAAGCCAAATGAAAATAAGCTGAAACAACCGGAAGGAAATGGGGGGGCAGGTCCAGCACAACCGGAAGGAAATGGGGGCGCAGGTCCAGCACAACCGGAAGGAAATGGGGGGGCAGGTCCAGCACAACCGGAAGGAAATGGGGGGGCAGGTCCAGCACAACCGGAAGGAAATGGGGGGGCAGGTCCAGCACAACCGGAAGGAAATGGGGGGGCAGGTCCAGCACAACCAGAAGGAAATGGGGGGGCAGGTCCAGCACAACCGGAAGGAAATGGGGGGGCAGGTCCAGCACAACCGGAAGGAAATGGGGGGGCAGGTCCAGCACAACCGGAAGGAAATGGGGGGGCAGGTCCAGCACAACCACAAGGAAATGGGGGGGCAGGTCCAGCACAACCACAAGGAAATGGGGGGGCAGGTCCAGCACAACCACAAGGAAATGGGGGGGCAGGTCCAGCACAACCACAAGGAAATGGGGGGGCAGGTCAAGCACAACCACAAAAAAACGAAGGAGGAAACGCAGGAGCACGGAAAGGACAGGGACAAAACAATCAGGGTGCGAATGCCCCAAATGAAAAAGTTGTAAATGATTACCTACAGAAAATTAGATCTAGCGTTACCACCGAGTGGACTCCATGCAGTGTAACCTGTGGAAATGGTGTAAGAATTAAAAGAAGAGCTCATGCAGATAAGAAAAAGGCAGAGGACCTTACTATGGATGACCTTGAAGTGGAAGCTTGTGTAATGGATAAGTGTGCTGGCATATTTAACGTTGTGAGTAATTCATTAGGGTTAGTCATATTGTTAGTCCTAGCATTATTCAATTAA------------------------------------------------------------------------------------------------------------------------------------------------------------------------------------------------------------------------------------------------------------------------------------------------------------------------------------------------------------------------------------------------------------------

>KF861718 Human Peninsular Malaysia

ATGAAGAACTTCATTCTCTTGGCCGTCTCCTCCATCCTGCTGGTGGACTTGCTCCCCACATACTTCGAACATAATGTAGATCTCTCCAGGGCCATAAATGTAAATGGAGTAAGCTTCAATAATGTAGACACCAGTTCACTTGGCGCAGCACAGGTAAGACAAAGTGCTAGCCGAGGCAGAGGACTTGGTGAGAAGCCAAAAGAAGGAGCTGATAAAGAAAAGAAAAAAGAAAAAGAAAAAGAAAAAGAAGAAGAACCAAAGAAGCCAAATGAAAATAAGCTGAAACAACCGGAAGGAAATGGGGGGGCAGGTCCAGCACAACCGGAAGGAAATGGGGGCGCAGGTCCAGCACAACCGGAAGGAAATGGGGGGGCAGGTCCAGCACAACCGGAAGGAAATGGGGGGGCAGGTCCAGCACAACCGGAAGGAAATGGGGGGGCAGGTCCAGCACAACCGGAAGGAAATGGGGGGGCAGGTCCAGCACAACCAGAAGGAAATGGGGGGGCAGGTCCAGCACAACCGGAAGGAAATGGGGGGGCAGGTCCAGCACAACCGGAAGGAAATGGGGGGGCAGGTCCAGCACAACCGGGGGGGCAGGTCCAGCACAACCGGAAGGAAATGGGGGGGCAGGTCCAGCACAACCGGAAGGAAATGGGGGGGCAGGTCCAGCACAACCACAAGGAAATGGGGGGGCAGGTCCAGCACAACCACAAGGAAATGGGGGGGGCAGGTCCAGCACAACCACAAGGAAATGGGGGGGCAGGTCCAGCACAACCACAAGGAAATGGGGGGGCAGGTCAAGCACAACCACAAAAAAACGAAGGAGGAAACGCAGGAGCACGGAAAGGACAGGGACAAAACAATCAGGGTGCGAATGCCCCAAATGAAAAAGTTGTAAATGATTACCTACAGAAAATTAGATCTAGCGTTACCACCGAGTGGACTCCATGCAGTGTAACCTGTGGAGATGGTGTAAGAATTAAAAGAAGAGCTCATGCAGATAAGAAAAAGGCAGAGGACCTTACTATGGATGACCTTGAAGTGGAAGCTTGTGTAATGGATAAGTGTGCTGGCATATTTAACGTTGTGAGTAATTCATTAGGGTTAGTCATATTGTTAGTCCTAGCATTATTCAATTAA---------------------------------------------------------------------------------------------------------------------------------------------------------------------------------------------------------------------------------------------------------------------------------------------------------------------------------------------------------

>KF861717 Human Peninsular Malaysia

ATGAAGAACTTCATTCTCTTGGCCGTCTCCTCCATCCTGCTGGTGGACTTGCTCCCCACACACTTCGAACATAATGTAGATCTCTCCAGGGCCATAAATGTAAATGGAGTAAGCTTCAATAATGTAGACACCAGTTCACTTGGCGCAGCACAGGTGAGACAAAGCGCTAGCCGAGGCAGAGGACTTGGTGAGAAGCCAAAAGAAGGAGCTGATAAAGAAAAGAAAAAAGAAAAAGGAAAAGAAAAAGAAGAAGAACCAAAGAAGCCAAATGAAAATAAGCTGAAACAACCGAATGAAGGACAACCACAAGCACAGGGTGATGGAGCAAATGCAGGACAACCACAAGCACAAGGAGATGGAGCAAATGCAGGACAACCACAAGCACAAGGAGATGGAGCAAATGCAGGACAACCACAAGCACAGGGTGATGGAGCAAATGCAGGACAACCACAAGCACAGGGTGATGGAGCAAATGCAGGACAACCACAAGCACAAGGAGATGGAGCAAATGCAGGACAACCACAAGCACAGGGTGATGGAGCAAATGCAGGGCAACCACAAGCACAGGGTGATGGAGCAAATGGTGATGGAGCAAATGCAGGGCAACCACAAGCACAGGGTGATGGAGCAAATGCAGGACAACCACAAGCACAAGGAGATGGAGCAAATGCAGGACAACCACAAGCACAAGGAGATGGAGCAAATGCAGGACAACCACAAGCACAGGGTGATGGAGCAAATGCAGGACAACCACAAGCACAGGGTGATAGGGCGAATGCAGGACAACCACAAGCACAAGGAGATGGGGCAAATGTACCACGACAAGGAAGAAACGGGGGAGGTGCACCAGCAGGAGGAAATGAGGGGAATAAACAAGCAGGAAAAGGACAGGGACAAAACAATCAGGGTGCGAATGCCCCAAATGAAAAAGTTGTGAATGATTACCTACACAAAATTAGATCTAGCGTTACCACCGAGTGGACTCCATGCAGTGTAACCTGTGGAAATGGTGTAAGAATTAGAAGAAAAGCTCATGCAGGTAATAAAAAGGCAGAGGACCTTACTATGGATGACCTTGAGGTGGAAGCTTGTGTAATGGATAAGTGCGCTGGCATATTTAACGTTGTGAGTAATTCATTAGGCTTAGTCATATTGTTAGTCCTAGCATTATTCAATTAA------------------------------------------------------------------------------------------------------------------------------------------------------------------------------------------------------------------------------------------------------------------------------------------------------------------

>KF861716 Human Peninsular Malaysia

ATGAAGAACTTCATTCTCTTGGCCGTCTCCTCCATCCTGCTGGTGGACTTGCTCCCCACACACTTCGAACATAATGTAGATCTCTCCAGGGCCATAAATGTAAATGGAGTAAGCTTCAATAATGTAGACACCAGTTCACTTGGCGCAGCACAGGTGAGACAAAGTGCTAGCCGAGGCAGAGGACTTGGTGAGAAGCCAAAAGAAGGAGCTGATAAAGAAAAGAAAAAAGAAAAAGGAAAAGAAAAAGAAGAAGAACCAAAGAAGCCAAATGAAAATAAGCTGAAACAACCGAATGAAGGACAACCACAAGCACAGGGTGATGGAGCAAATGCAGGACAACCACAAGCACAAGGAGATGGAGCAAATGCAGGACAACCACAAGCACAAGGAGATGGAGCAAATGCAGGACAACCACAAGCACAGGGTGATGGAGCAAATGCAGGACAACCACAAGCACAGGGTGATGGAGCAAATGCAGGACAACCACAAGCACAAGGAGATGGAGCAAATGCAGGACAACCACAAGCACAGGGTGATGGAGCAAATGCAGGGCAACCACAAGCACAGGGTGATGGAGCAAATGCAGGACAACCACAAGCACAAGGAGATGGAGCAAATGCAGGACAACCACAAGCACAAGGAGATGGAGCAAATGCAGGACAACCACAAGCACAGGGTGATGGAGCAAATGCAGGACAACCACAAGCACAGGGTGATAGGGCGAATGCAGGACAACCACAAGCACAAGGAGATGGGGCAAATGTACCACGACAAGGAAGAAACGGGGGAGGTGCACCAGCAGGAGGAAATGAGGGGAATAAACAAGCAGGAAAAGGACAGGGACAAAACAATCAGGGTGCGAATGCCCCAAATGAAAAAGTTGTGAATGATTACCTACACAAAATTAGATCTAGCGTTACCACCGAGTGGACTCCATGCAGTGTAACCTGTGGAAATGGTGTAAGAATTAGAAGAAAAGCTCATGCAGGTAATAAAAAGGCAGAGGACCTTACTATGGATGACCTTGAGGTGGAAGCTTGTGTAATGGATAAGTGCGCTGGCATATTTAACGTTGTGAGTAATTCATTAGGCTTAGTCATATTGTTAGTCCTAGCATTATTCAATTAA---------------------------------------------------------------------------------------------------------------------------------------------------------------------------------------------------------------------------------------------------------------------------------------------------------------------------------------------------------------------

>KF861715 Human Peninsular Malaysia

ATGAAGAACTTCATTCTCTTGGCCGTCTCCTCCATCCTGCTGGTGGACTTGCTCCCAACACACTTCGAACATAATGTAGATCTCTCCAGGGCCATAAATGTAAATGGAGTAAGCTTCAATAATGTAGACACCAGTTCACTTGGCGCAGCACAGGTAAGACAAAGTGCTAGCCGAGGCAGAGGACTTGGTGAGAAACCAAAAGAAGGAGCTGATAAAGAAAAGAAAAAAGAAAAAGAAAAAGAAGAAGAACCAAAGAAGCCAAATGAAAATAAGCTGAAACAACCGGAAGGAAATAGGGAAGCTCCAGGACAACCGGAAGGAAATAGGGAAGCTCCAGGACAACCGGAAGGAAATAGGGAAGCTCCAGGACAACCGGAAGGAAATAGGGAAGCTCCAGGACAACCGGAAGGAAATAGGGAAGCTCCAGGACAACCGGAAGGAAATAGGGAAGCTCCAGGACAACCGGAAGGAAATAGGGAAGCTCCAGGACAACCGGAAGGAAATAGGGAAGCTCCAGGACAACCGGAAGGAAATAGGGAAGCTCCAGGACAACCGGAAGGAAATAGGGAAGCTCCAGGACAACCGGAAGGAAATAGGGAAGCTCCAGGACAACCGGAAGGAAATAGGGAAGCTCCAGGACAACCGGAAGGAAATAGGGAAGCTCCAGCACAACCGGAAGGAAATAGGGAAGCTCCAGCACAACCACAAGGAAATGGGGGGGCAGGTCAAGCACAACCACAAAAAAACGAAGGAGGAAACGCAGGAGCACGGAAAGGACAGGGACAAAACAATCAGGGTGCGAATGCCCCAAATGAAAAAGTTGTAAATGATTACCTACAGAAAATTAGATCTAGCGTTACCACCGAGTGGACTCCATGCAGTGTAACCTGTGGAAATGGTGTAAGAATTAAAAGAAAAGCTCATGCAGATAAGAAAAAGGCAGAGGACCTTACTATGGATGACCTTGAAGTGGAAGCTTGTGTAATGGATAAGTGTGCTGGCATATTTAACGTTGTGAGTAATTCATTAGGGTTAGTCATATTGTTAGTCCTAGCATTATTCAATTAA---------------------------------------------------------------------------------------------------------------------------------------------------------------------------------------------------------------------------------------------------------------------------------------------------------------------------------------------------------------------------------------------------------------------------------

>KF861714 Human Peninsular Malaysia

ATGAAGAACTTCATTCTCTTGGCCGTCTCCTCCATCCTGCTGGTGGACTTGCTCCCAACACACTTCGAACATAATGTAGATCTCTCCAGGGCCATAAATGTAAATGGAGTAAGCTTCAATAATGTAGACACCAGTTCACTTGGCGCAGCACAGGTAAGACAAAGTGCTAGCCGAGGCAGAGGACTTGGTGAGAAACCAAAAGAAGGAGCTGATAAAGAAAAGAAAAAAGAAAAAGAAAAAGAAGAAGAACCAAAGAAGCCAAATGAAAATAAGCTGAAACAACCGGAAGGAAATAGGGAAGCTCCAGGACAACCGGAAGGAAATAGGGAAGCTCCAGGACAACCGGAAGGAAATAGGGAAGCTCCAGGACAACCGGAAGGAAATAGGGAAGCTCCAGGACAACCGGAAGGAAATAGGGAAGCTCCAGGACAACCGGAAGGAAATAGGGAAGCTCCAGGACAACCGGAAGGAAATAGGGAAGCTCCAGGACAACCGGAAGGAAATAGGGAAGCTCCAGGACAACCGGAAGGAAATAGGGAAGCTCCAGGACAACCGGAAGGAAATAGGGAAGCTCCAGGACAACCGGAAGGAAATAGGGAAGCTCCAGCACAACCGGAAGGAAATAGGGAAGCTCCAGGACAACCGGAAGGAAATAGGGAAGCTCCAGCACAACCGGAAGGAAATAGGGAAGCTCCAGCACAACCACAAGGAAATGGGGGGGCAGGTCAAGCACAACCACAAAAAAACGAAGGAGGAAACGCAGGAGCACGGAAAGGACAGGGACAAAACAATCAGGGTGCGAATGCCCCAAATGAAAAAGTTGTAAATGATTACCTACAGAAAATTAGATCTAGCGTTACCACCGAGTGGACTCCATGCAGTGTAACCTGTGGAAATGGTGTAAGAATTAAAAGAAAAGCTCATGCAGATAAGAAAAAGGCAGAGGACCTTACTATGGATGACCTTGAAGTGGAAGCTTGTGTAATGGATAAGTGTGCTGGCATATTTAACGTTGTGAGTAATTCATTAGGGTTAGTCATATTGTTAGTCCTAGCATTATTCAATTAA---------------------------------------------------------------------------------------------------------------------------------------------------------------------------------------------------------------------------------------------------------------------------------------------------------------------------------------------------------------------------------------------------------------------------------

>KF861713 Human Peninsular Malaysia

ATGAAGAACTTCATTCTCTTGGCCGTCTCCTCCATCCTGCTGGTGGACTTGCTCCCCACACACTTCGAACATAATGTAGATCTCTCCAGGGCCATAAATGTAAATGGAGTAAGCTTCAATAATGTAGACACCAGTTCACCTGGCGCAGCACAGGTAAGACAAAGTGCTAGCCGAGGCAGAGGACTTGGTGAGAAGCCAAAAGAAGGAGCTGATAAAGAAAAGAAAAAAGAAAAAGAAAAAGAAAAAGAAGAAGAACCAAAGAAGCCAAATGAAAATAAGCTGAAACAACCGGAACAACCAGCAGCAGGAGCAGGGGGCGAACAACCAGCAGCAGGAGCAGGAGGCGAACAACCAGCAGCAGGAGCAGGAGGCGAACAACCAGCAGCAGGAGCAGGAGGCGAACAACCAGCAGCAGGAGCAAGAGGCGAACAACCAGCAGCAGGAGCAGGAGGCGAACAACCAGCAGCAGGAGCAAGAGGCGAACAACCAGCAGCAGGAGCAGGAGGCGAACAACCAGCAGCAGGAGCAGGAGGCGAACAACCAGCAGCAGGAGCAGGAGGCGAACAACCAGCAGCAGGAGCAGGGGGCGAACAACCAGCAGCAGGAGCAGGAGGCGAACAACCAGCACCAGCACCAAGGAGGGAACAACCAGCAGCAGGAGCAGGGGGCGAACAACCAGCACCAGCACCAAGGAGGGAACAACCAGCACCAGGAGCAGGTGCGGGAGATGGAGCACGAGGAGGAAACGCAGGGGCAGGTAAAGGACAGGGACAAAACAATCAGGGTGCGAATGTCCCAAATGAAAAAGTTGTGAATGATTACCTACGCAAAATTAGATCTAGCGTTACCACCGAGTGGACTCCATGCAGTGTAACCTGTGGAAATGGTGTAAGAATTAGAAGAAAAGGTCATGCAGGTAATAAAAAGGCAGAGGACCTTACTATGGATGACCTTGAGGTGGAAGCTTGTGTAATGGATAAGTGCGCTGGCATATTTAACGTTGTGAGTAATTCATTAGGCTTAGTCATATTGTTAGTCCTAGCATTATTCAATTAA---------------------------------------------------------------------------------------------------------------------------------------------------------------------------------------------------------------------------------------------------------------------------------------------------------------------------------------------------------------------------------------------------------------------------------------------

>KF861712 Human Peninsular Malaysia

ATGAAGAACTTCATTCTCTTGGCCGTCTCCTCCATCCTGCTGGTGGACTTGCTCCCAACACACTTCGAACATAATGTAGATCTCTCCAGGGCCATAAATGTAAATGGAGTAAGCTTCAATAATGTAGACACCAGTTCACTTGGCGCAGCACAGGTAAGACAAAGTGCTAGCCGAGGCAGAGGACTTGGTGAGAAACCAAAAGAAGGAGCTGATAAAGAAAAGAAAAAAGAAAAAGAAAAAGAAGAAGAACCAAAGAAGCCAAATGAAAATAAGCTGAAACAACCGGAAGGAAATAGGGAAGCTCCAGGACAACCGGAAGGAAATAGGGAAGCTCCAGGACAACCGGAAGGAAATAGGGAAGCTCCAGGACAACCGGAAGGAAATAGGGAAGCTCCAGGACAACCGGAAGGAAATAGGGAAGCTCCAGGACAACCGGAAGGAAATAGGGAAGCTCCAGGACAACCGGAAGGAAATAGGGAAGCTCCAGGACAACCGGAAGGAAATAGGGAAGCTCCAGCACAACCGGAAGGAAATAGGGAAGCTCCAGGACAACCGGAAGGAAATAGGGAAGCTCCAGCACAACCGGAAGGAAATAGGGAAACTCCAGCACAACCACAAGGAAATGGGGGGGCAGGTCAAGCACAACCACAAAAAAACGAAGGAGGAAACGCAGGAGCACGGAAAGGACAGGGACAAAACAATCAGGGTGCGAATGCCCCAAATGAAAAAGTTGTAAATGATTACCTACAGAAAATTAGATCTAGCGTTACCACCGAGTGGACTCCATGCAGTGTAACCTGTGGAAATGGTGTAAGAATTAAAAGAAAAGCTCATGCAGATAAGAAAAAGGCAGAGGACCTTACTATGGATGACCTTGAAGTGGAAGCTTGTGTAATGGATAAGTGTGCTGGCATATTTAACGTTGTGAGTAATTCATTAGGGTTAGTCATATTGTTAGTCCTAGCATTATTCAATTAA---------------------------------------------------------------------------------------------------------------------------------------------------------------------------------------------------------------------------------------------------------------------------------------------------------------------------------------------------------------------------------------------------------------------------------------------------------------------------------------------------------------------------

>KF861711 Human Peninsular Malaysia

ATGAAGAACTTCATTCTCTTGGCCGTCTCCTCCATCCTGCTGGTGGACTTGCTCCCAACACACTTCGAACATAATGTAGATCTCTCCAGGGCCATAAATGTAAATGGAGTAAGCTTCAATAATGTAGACACCAGTTCACTTGGCGCAGCACAGGTAAGACAAAGTGCTAGCCGAGGCAGAGGACTTGGTGAGAAACCAAAAGAAGGAGCTGATAAAGAAAAGAAAAAAGAAAAAGAAAAAGAAGAAGAACCAAAGAAGCCAAATGAAAATAAGCTGAAACAACCGGAAGGAAATAGGGAAGCTCCAGGACAACCGGAAGGAAATAGGGAAGCTCCAGGACAACCGGAAGGAAATAGGGAAGCTCCAGGACAACCGGAAGGAAATAGGGAAGCTCCAGGACAACCGGAAGGAAATAGGGAAGCTCCAGGACAACCGGAAGGAAATAGGGAAGCTCCAGGACAACCGGAAGGAAATAGGGAAGCTCCAGGACAACCGGAAGGAAATAGGGAAGCTCCAGGACAACCGGAAGGAAATAGGGAAGCTCCAGGACAACCGGAAGGAAATAGGGAAGCTCCAGGACAACCGGAAGGAAATAGGGAAGCTCCAGCACAACCGGAAGGAAATAGGGAAGCTCCAGGACAACCGGAAGGAAATAGGGAAGCTCCAGCACAACCGGAAGGAAATAGGGAAGCTCCAGCACAACCACAAGGAAATGGGGGGGCAGGTCAAGCACAACCACAAAAAAACGAAGGAGGAAACGCAGGAGCACGGAAAGGACAGGGACAAAACAATCAGGGTGCGAATGCCCCAAATGAAAAAGTTGTAAATGATTACCTACAGAAAATTAGATCTAGCGTTACCACCGAGTGGACTCCATGCAGTGTAACCTGTGGAAATGGTGTAAGAATTAAAAGAAAAGCTCATGCAGATAAGAAAAAGGCAGAGGACCTTACTATGGATGACCTTGAAGTGGAAGCTTGTGTAATGGATAAGTGTGCTGGCATATTTAACGTTGTGAGTAATTCATTAGGGTTAGTCATATTGTTAGTCCTAGCATTATTCAATTAA---------------------------------------------------------------------------------------------------------------------------------------------------------------------------------------------------------------------------------------------------------------------------------------------------------------------------------------------------------------------------------------------------------------------------------

>KF861710 Human Peninsular Malaysia

ATGAAGAACTTCATTCTCTTGGCCGTCTCCTCCATCCTGCTGGTGGACTTGCTCCCAACACACTTCGAACATAATGTAGATCTCTCCAGGGCCATAAATGTAAATGGAGTAAGCCTCAATAATGTAGACACCAGTTCACTTGGCGCAGCACAGGTAAGACAAAGTGCTAGCCGAGGCAGAGGACTTGGTGAGAAACCAAAAGAAGGAGCTGATAAAGAAAAGAAAAAAGAAAAAGAAAAAGAAGAAGAACCAAAGAAGCCAAATGAAAATAAGCTGAAACAACCGGAAGGAAATAGGGAAGCTCCAGGACAACCGGAAGGAAATAGGGAAGCTCCAGGACAACCGGAAGGAAATAGGGAAGCTCCAGGACAACCGGAAGGAAATAGGGAAGCTCCAGGACAACCGGAAGGAAATAGGGAAGCTCCAGGACAACCGGAAGGAAATAGGGAAGCTCCAGGACAACCGGAAGGAAATAGGGAAGCTCCAGGACAACCGGAAGGAAATAGGGAAGCTCCAGGACAACCGGAAGGAAATAGGGAAGCTCCAGGACAACCGGAAGGAAATAGGGAAGCTCCAGGACAACCGGAAGGAGATAGGGAAGCTCCAGCACAACCGGAAGGAAATAGGGAAGCTCCAGGACAACCGGAAGGAAATAGGGAAGTTCCAGCACAACCGGAAGGAAATAGGGAAGCTCCAGCACAACCACAAGGAAATGGGGGGGCAGGTCAAGCACAACCACAAAAAAACGAAGGAGGAAACGCAGGAGCACGGAAAGGACAGGGACAAAACAATCAGGGTGCGAATGCCCCAAATGAAAAAGTTGTAAATGATTACCTACAGAAAATTAGATCTAGCGTTACCACCGAGTGGACTCCATGCAGTGTAACCTGTGGAAATGGTGTAAGAATTAAAAGAAAAGCTCATGCAGATAAGAAAAAGGCAGAGGACCTTACTATGGATGACCTTGAAGTGGAAGCTTGTGTAATGGATAAGTGTGCTGGCATATTTAACGTTGTGAGTAATTCATTAGGGTTAGTCATATTGTTAGTCCTAGCATTATTCAATTAA---------------------------------------------------------------------------------------------------------------------------------------------------------------------------------------------------------------------------------------------------------------------------------------------------------------------------------------------------------------------------------------------------------------------------------

>KF861709 Human Peninsular Malaysia

TTGAAGAACTTCATTCTCTTGGCCGTCTCCTCCATCCTGCTGGTGGACTTGCTCCCCACACACTTCGAACATAATGTAGATCTCTCCAGGGCCATAAATGTAAATGGAGTAAGCTTCAATAATGTAGACACCAGTTCACTTGGCGCAGCACAGGTAAGACAAAGTGCTAGCCGAGGCAGAGGACTTGGTGAGAAGCCAAAAGAAGGAGATGATAAAGAAAAGAAAAAAGAAAAAGAAAAAGGAGAAGAACCAAAGAAGCTAAATGAAAATAATCCGAAAAAACCGAATGAAGAAGGTGATGGAGCTAAGCTGAAACAACCGAATGAAGAAGGTGATGGAGCTAAGCTGAAACAACCGAATGAAGAAGGTGATGGAGCTAAGCTGAAACAACCGAATGCAGAAGGTGGAGCTAAGCTGAAACAACCGAATGCAGAAGGTGGAGCAAATGCAGGACAACCGAATGCAGAAGGTGGAGCAAATGCAGGACAACCGAATGCAGAAGGTGGAGCAAATGCAGGACAACCGAATGCAGGTGGAGCAAATGCAGGACAACCGAATGCAGAAGGTGGAGCAAATGCAGGACAACCGAATGCAGAAGGTGGAGCAAATGCAGGACAACCGAATGCAGAAGGTGGAGCAAATGCAGGACAACCGAATGCAGAAGGTGGAGCAAATGCACGACAACCTAATGCAGAAGGTGGAGCAAATGCAGGACAACCGAATGCAGAAGGTGGAGCAAATGCAGGACAACCGAATGCAGAAGGTGGAGCAAATGCAGGACGACCGAATGCAGAAGGTGGAGCAAATGCACGACAACCTAATGCAGAAGGTGGAGCAAACGCAGGACAACCGAATGCAGAAGGTGGAGCAAATGCAGGACAACCGAATGCAGAAGGTGGAGCAAATGCACGACAGCCACAGGCAGAAGGTGGTGGAGCAAATGCACGACAAGGAGGAAATGAGGGGAATAAACAAGCAGGAAAAGGACAGGGACAAAACAATCAGGGTGCGAATGCCCCAAATGAAAAAGTTGTAAATGATTACCTACAGAAAATTAGATCTAGCGTTACCATCGAGTGGACTCCATGCAGTGTAACCTGTGGAAATGGTGTAAGAATTAGAAGAAGAGCTCATGCAGATAAGAAAAAGGCAGAGGACCTTACTATGGATGACCTTGAAGTGGAAGCTTGTGTAATGGATAAGTGTGCTGGCATATTTAACGTTGTGAGTAATTCATTAGGGTTAGTAATATTGTTAGTCCTAGCATTATTCAATTAA---------------------------------------------------------------------------------------------------------------------------------------------------------------------------------------------------------------

>KF861708 Human Peninsular Malaysia

ATGAAGAACTTCATTCTCTTGGCCGTCTCCTCCATCCTGCTGGTGGACTTGCTCCCCACACACTTCGAACATAATGTAGATCTCTCCAGGGCCATAAATGTAAATGGAGTAAGCTTCAATAATGTAGACACCAGTTCACTTGGCGCAGCACAGGTAAGACAAAGTGCTAGCCGAGGCAGAGGACTTGGTGAGAAGCCAAAAGAAGGAGATGATAAAGAAAAGAAAAAAGAAAAAGAAAAAGAAGAAGAACCAAAGAAGCTAAATGAAAATAATCCGAAAAAACCGAATGAAGAAGGTGATGGAGCTAAGCTGGAACAACCGAATGAAGAAGGTGATGGAGCTAAGCTGAAACAACCGAATGAAGAAGGTGATGGAGCTAAGCTGAAACAACCGAATGCAGAAGGTGGAGCTAAGCTGAAACAACCGAATGCAGAAGGTGGAGCAAATGCAGGACAACCGAATGCAGAAGGTGGAGCAAATGCAGGACAACCGAATGCAGAAGGTGGAGCAAATGCAGGACAACCGAATGCAGGTGGAGCAAATGCAGGACAACCGAATGCAGAAGGTGGAGCAAATGCAGGACAACCGAATGCAGAAGGTGGAGCAAATGCAGGACAACCGAATGCAGAAGGTGGAGCAAATGCAGGACAACCGAATGCAGAAGGTGGAGCAAATGCACGACAACCTAATGCAGAAGGTGGAGCAAATGCAGGACAACCGAATGCAGAAGGTGGAGCAAATGCAGGACAACCGAATGCAGAAGGTGGAGCAAATGCAGGACAACCGAATGCAGAAGGTGGAGCAAATGCACGACAACCTAATGCAGAAGGTGGAGCAAACGCAGGACAACCGAATGCAGAAGGTGGAGCAAATGCAGGACAACCGAATGCAGAAGGTGGAGCAAATGCACGACAGCCACAGGCAGAAGGTGGTGGAGCAAATGCACGACAAGGAGGAAATGAGGGGAATAAACAAGCAGGAAAAGGACAGGGACAAAACAATCAGGGTGCGAATGCCCCAAATGAAAAAGTTGTAAATGATTACCTACAGAAAATTAGATCTAGCGTTACCATCGAGTGGACTCCATGCAGTGTAACCTGTGGAAATGGTGTAAGAATTAGAAGAAGAGCTCATGCAGATAAGAAAAAGGCAGAGGACCTTACTATGGGTGACCTTGAAGTGGAAGCTTGTGTAATGGATAAGTGTGCTGGCGTATTTAACGTTGTGAGTAATTCATTAGGGTTAGTAATATTGTTAGTCCTAGCATTATTCAATTAA---------------------------------------------------------------------------------------------------------------------------------------------------------------------------------------------------------------

>KF861707 Human Peninsular Malaysia

ATGAAGAACTTCATTCTCTTGGCCGTCTCCTCCATCCTGCTGGTGGACTTGCTCCCCACACACTTCGAACATAATGTAGATCTCTCCAGGGCCATAAATGTAAATGGAGTAAGCTTCAATAATGTAGACACCAGTTCACTTGGCGCAGCACAGGTAAGACAAAGTGCTAGCCGAGGCAGAGGACTTGGTGAGAAGCCAAAAGAAGGAGATGATAAAGAAAAGAAAAAAGAAAAAGAAAAAGAAGAAGAACCAAAGAAGCTAAATGAAAATAATCCGAAAAAACCGAATGAAGAAGGTGATGGAGCTAAGCTGGAACAACCGAATGAAGAAGGTGATGGAGCTAAGCTGAAACAACCGAATGAAGAAGGTGATGGAGCTAAGCTGAAACAACCGAATGCAGAAGGTGGAGCTAAGCTGAAACAACCGAATGCAGAAGGTGGAGCAAATGCAGGACAACCGAATGCAGAAGGTGGAGCAAATGCAGGACAACCGAATGCAGAAGGTGGAGCAAATGCAGGACAACCGAATGCAGGTGGAGCAAATGCAGGACAACCGAATGCAGAAGGTGGAGCAAATGCAGGACAACCGAATGCAGAAGGTGGAGCAAATGCAGGACAACCGAATGCAGAAGGTGGAGCAAATGCAGGACAACCGAATGCAGAAGGTGGAGCAAATGCACGACAACCTAATGCAGAAGGTGGAGCAAATGCAGGACAACCGAATGCAGAAGGTGGAGCAAATGCAGGACAACCGAATGCAGAAGGTGGAGCAAATGCAGGACAACCGAATGCAGAAGGTGGAGCAAATGCACGACAACCTAATGCAGAAGGTGGAGCAAACGCAGGACAACCGAATGCAGAAGGTGGAGCAAATGCAGGACAACCGAATGCAGAAGGTGGAGCAAATGCACGACAGCCACAGGCAGAAGGTGGTGGAGCAAATGCACGACAAGGAGGAAATGAGGGGAATAAACAAGCAGGAAAAGGACAGGGACAAAACAATCAGGGTGCGAATGCCCCAAATGAAAAAGTTGTAAATGATTACCTACAGAAAATTAGATCTAGCGTTACCATCGAGTGGACTCCATGCAGTGTAACCTGTGGAAATGGTGTAAGAATTAGAAGAAGAGCTCATGCAGATAAGAAAAAGGCAGAGGACCTTACTATGGGTGACCTTGAAGTGGAAGCTTGTGTAATGGATAAGTGTGCTGGCGTATTTAACGTTGTGAGTAATTCATTAGGGTTAGTAATATTGTTAGTCCTAGCATTATTCAATTAA---------------------------------------------------------------------------------------------------------------------------------------------------------------------------------------------------------------

>KF861706 Human Peninsular Malaysia

ATGAAGAACTTCATTCTCTTGGCCGTCTCCTCCATCCTGCTGGTGGACTTGCTCCCAACACACTTCGAACATAATGTAGATCTCTCCAGGGCCATAAATGTAAATGGAGTAAGCTTCAATAATGTAGACACCAGTTCACTTGGCGCAGCACAGGTAAGACAAAGTGCTAGCCGAGGCAGAGGACTTGGTGAGAAACCAAAAGAAGGAGCTGATAAAGAAAAGAAAAAAGAAAAAGAAAAAGAAGAAGAACCAAAGAAGCCAAATGAAAATAAGCTGAAACAACCGGAAGGAAATAGGGAAGCTCCAGGACAACCGGAAGGAAATAGGGAAGCTCCAGGACAACCGGAAGGAAATAGGGAAGCTCCAGGACAACCGGAAGGAAATAGGGAAGCTCCAGGACAACCGGAAGGAAATAGGGAAGCTCCAGGACAACCGGAAGGAAATAGGGAAGCTCCAGGACAACCGGAAGGAAATAGGGAAGCTCCAGGACAACCGGAAGGAAATAGGGAAGCTCCAGCACAACCGGAAGGAAATAGGGAAGCTCCAGGACAACCGGAAGGAAATAGGGAAGCTCCAGCACAACCGGAAGGAAATAGGGAAGCTCCAGCACAACCACAAGGAAATGGGGGGGCAGGTCAAGCACAACCACAAAAAAACGAAGGAGGAAACGCAGGAGCACGGAAAGGACAGGGACAAAACAATCAGGGTGCGAATGCCCCAAATGAAAAAGTTGTAAATGATTACCTACAGAAAATTAGATCTAGCGTTACCACCGAGTGGACTCCATGCAGTGTAACCTGTGGAAATGGTGTAAGAATTAAAAGAAAAGCTCATGCAGATAAGAAAAAGGCAGAGGACCTTACTATGGATGACCTTGAAGTGGAAGCTTGTGTAATGGATAAGTGTGCTGGCATATTTAACGTTGTGAGTAATTCATTAGGGTTAGTCATATTGTTAGTCCTAGCATTATTCAATTAA---------------------------------------------------------------------------------------------------------------------------------------------------------------------------------------------------------------------------------------------------------------------------------------------------------------------------------------------------------------------------------------------------------------------------------------------------------------------------------------------------------------------------

>KF861705 Human Peninsular Malaysia

ATGAAGAACTTCATTCTCTTGGCCGTCTCCTCCATCCTGCTGGTGGACTTGCTCCCAACACACTTCGAACATAATGTAGATCTCTCCAGGGCCATAAATGTAAATGGAGTAAGCTTCAATAATGTAGACACCAGTTCACTTGGCGCAGCACAGGTAAGACAAAGTGCTAGCCGAGGCAGAGGACTTGGTGAGAAACCAAAAGAAGGAGCTGATAAAGAAAAGAAAAAAGAAAAAGAAAAAGAAGAAGAACCAAAGAAGCCAAATGAAAATAAGCTGAAACAACCGGAAGGAAATAGGGAAGCTCCAGGACAACCGGAAGGAAATAGGGAAGCTCCAGGACAACCGGAAGGAAATAGGGAAGCTCCAGGACAACCGGAAGGAAATAGGGAAGCTCCAGGACAACCGGAAGGAAATAGGGAAGCTCCAGGACAACCGGAAGGAAATAGGGAAGCTCCAGGACAACCGGAAGGAAATAGGGAAGCTCCAGGACAACCGGAAGGAAATAGGGAAGCTCCAGGACAACCGGAAGGAAATAGGGAAGCTCCAGGACAACCGGAAGGAAATAGGGAAGCTCCAGCACAACCGGAAGGAAATAGGGAAGCTCCAGGACAACCGGAAGGAAATAGGGAAGCTCCAGCACAACCGGAAGGAAATAGGGAAGCTCCAGCACAACCACAAGGAAATGGGGGGGCAGGTCAAGCACAACCACAAAAAAACGAAGGAGGAAACGCAGGAGCACGGAAAGGACAGGGACAAAACAATCAGGGTGCGAATGCCCCAAATGAAAAAGTTGTAAATGATTACCTACAGAAAATTAGATCTAGCGTTACCACCGAGTGGACTCCATGCAGTGTAACCTGTGGAAATGGTGTAAGAATTAAAAGAAAAGCTCATGCAGATAAGAAAAAGGCAGAGGACCTTACTATGGATGACCTTGAAGTGGAAGCTTGTGTAATGGATAAGTGTGCTGGCATATTTAACGTTGTGAGTAATTCATTAGGGTTAGTCATATTGTTAGTCCTAGCATTATTCAATTAA---------------------------------------------------------------------------------------------------------------------------------------------------------------------------------------------------------------------------------------------------------------------------------------------------------------------------------------------------------------------------------------------------------------------------------------------------------------

>KF861704 Human Peninsular Malaysia

ATGAAGAACTTCATTCTCTTGGCCGTCTCCTCCATCCTGCTGGTGGACTTGCTCCCCACACACTTCGAACATAATGTAGATCTCTCCAGGGCCATAAATGTAAATGGAGTAAGCTTCAATAATGTAGACACCAGTTCACTTGGCGCAGCACAGGTAAGACAAAGTGCTAGCCGAGGCAGAGGACTTGGTGAGAAACCAAAAGAAGGAGCTGATAAAGAAAAGAAAAAAGAAAAAGAAAAAGAAGAAGAACCAAAGAAGCCAAATGAAAATAAGCTGAAACAACCGGAAGGAAATAGGGAAGCTCCAGGACAACCGGAAGGAAATAGGGAAGCTCCAGGACAACCGGAAGGAAATAGGGAAGCTCCAGGACAACCGGAAGGAAATAGGGAAGCTCCAGGACAACCGGAAGGAAATAGGGAAGCTCCAGGACAACCGGAAGGAAATAGGGAAGCTCCAGGACAACCGGAAGGAAATAGGGAAGCTCCAGGACAACCGGAAGGAAATAGGGAAGCTCCAGGACAACCGGAAGGAAATAGGGAAGCTCCAGCACAACCGGAAGGAAATAGGGAAGCTCCAGGACAACCGGAAGGAAATAGGGAAGCTCCAGCACAACCACAAGGAAATGGGGGGGCAGGTCAAGCACAACCGGAAGAAAATAGGGAAGCTCCAGCACAACCACAAGGAAATGGGGGGGCAGGTCAAGCACAACCACAAAAAAACGAAGGAGGAAACGCAGGAGCACGGAAAGGACAGGGACAAAACAATCAGGGTGCGAATGTCCCAAATGAAAAAGTTGTGAATGATTACCTACACAAAATTAGATCTAGCGTTACCACCGAGTGGACTCCATGCAGTGTAACCTGTGGAAATGGTGTAAGAATTAGAAGAAAAGGTCATGCAGGTAATAAAAAGGCAGAGGACCTTACTATGGATGACCTTGAGGTGGAAGCTTGTGTAATGGATAAGTGCGCTGGCATATTTAACGTTGTGAGTAATTCATTAGGCTTAGTCATATTGTTAGTCCCAGCATTATTCAATTAA------------------------------------------------------------------------------------------------------------------------------------------------------------------------------------------------------------------------------------------------------------------------------------------------------------------------------------------------------------------------------------------------------------------------------------------------------------

>KF861703 Human Peninsular Malaysia

ATGAAGAACTTCATTCTCTTGGCCGTCTCCTCCATCCTGCTGGTGGACTTGCTCCCCACACACTTCGAACATAATGTAGATCTCTCCAGGGCCATAAATGTAAATGGAGTAAGCTTCAATAATGTAGACACCAGTTCACTTGGCGCAGCACAGGTAAGACAAAGTGCTAGCCGAGGCAGAGGACTTGGTGAGAAGCCAAAAGAAGGAGCTGATAAAGAAAAGAAAAAAGAAAAAGAAAAAGAAAAAGAAGAAGAACCAAAGAAGCCTAATGAAAATAAGCTGAAACAACCGGAACAACCAGCAGCAGGAGCAGGGGGCGAACAACCAGCAGCAGGAGCAGGAGGCGAACAACCAGCAGCAGGAGCAGGAGGCGAACAACCAGCAGCAGGAGCAGGAGGCGAACAACCAGCAGCAGGAGCAGGAGGCGAACAACCAGCAGCAGGAGCAAGAGGCGAACAACCAGCAGCAGGAGCAGGAGGCGAACAACCAGCAGCAGGAGCAGGAGGCGAACAACCAGCAGCAGGAGCAGGAGGCGAACAACCAGCAGCAGGAGCAAGAGGCGAACAACCAGCAGCAGGAGCAGGAGGCGAACAACCAGCAGCAGGAGCAGGAGGCGAACAACCAGCACCAGCACCAAGGAGGGAACAACCAGCAGCAGGAGCAGGGGGCGAACAACCAGCACCAGCACCAAGGAGGGAACAACCAGCACCAGGAGCAGGTGCGGGAGATGGAGCACGAGGAGGAAACGCAGGGGCAGGTAAAGGACAGGGACAAAACAATCAGGGTGCGAATGTCCCAAATGAAAAAGTTGTGAATGATTACCTACACAAAACTAGATCTAGCGTTACCACCGAGTGGACTCCATGCAGTGTAACCTGTGGAAATGGTGTAAGAATTAGAAGAAAAGGTCATGCAGGTAATAAAAAGGCAGAGGACCTTACTATGGATGACCTTGAGGTGGAAGCTTGTGTAATGGATAAGTGTGCTGGCATATTTAACGTTGTGAGTAATTCATTAGGCTTAGTCATATTGTTAGTCCTAGCATTATTCAATTAA---------------------------------------------------------------------------------------------------------------------------------------------------------------------------------------------------------------------------------------------------------------------------------------------------------------------------------------------------------------------------------------------------------------------------------------------

>KF861702 Human Peninsular Malaysia

ATGAAGAACTTCATTCTCTTGGCCGTCTCCTCCATCCTGTTGGTGGACTTGCTCCCCACACACTTCGAACATAATGTAGATCTCTCCAGGGCCATAAATGTAAATGGAGTAAGCTTCAATAATGTAGACACCAGTTCACTTGGCGCAGCACAGGTAAGACAAAGTGCTAGCCGAGGCAGAGGACTTGGTGAGAAGCCAAAAGAAGGAGCTGATAAAGAAAAGAAAAAAGAAAAAGAAAAAGAAAAAGAAGAAGAACCAAAGAAGCCAAATGAAAATAAGCTGATACAACCGGAACAACCAGCAGCAGGAGCAGGGGGCGAACAACCAGCAGCAGGAGCAGGGGGCGAACAACCAGCAGCAGGAGCAGGAGGCGAACAACCAGCAGCAGGAGCAGGAGGCGAACAACCAGCAGCAGGAGCAGGAGGCGAACAACCAGCAGCAGGAGCAAGAGGCGAACAACCAGCAGCAGGAGCAGGAGGCGAACAACCAGCAGCAGGAGCAGGAGGCGAACAACCAGCAGCAGGAGCAGGAGGCGAACAACCAGCAGCAGGAGCAGGAGGCGAACAACCAGCAGCAGGAGCAGGAGGCGAACAACCAGCAGCAGGAGCAAGAGGCGAACAACCAGCAGCAGGAGCAGGAGGCGAACAACCAGCAGCAGGAGCAGGAGGCGAACAACCAGCACCAGCACCAAGGAGGGAACAACCAGCAGCAGGAGCAGGGGGCGAACAACCAGCACCAGCACCAAGGAGGGAACAACCAGCACCAGGAGCAGGTGCGGGAGATGGAGCACGAGGAGGAAACGCAGGGGCAGGTAAAGGACAGGGACAAAACAATCAGGGTGCGAATGTCCCAAATGAAAAAGTTGTGAATGATTACCTACACAAAATTAGATCTAGCGTTACCACCGAGTGGACTCCATGCAGTGTAACCTGTGGAAATGGTGTAAGAATTAGAAGAAAAGGTCATGCAGGTAATAAAAAGGCAGAGGACCTTACTATGGATGACCTTGAGGTGGAAGCTTGTGTAATGGATAAGTGCGCTGGCATATTTAACGTTGTGAGTAATTCACTAGGCTTAGTCATATTGTTAGTCCTAGCATTATTCAATTAA---------------------------------------------------------------------------------------------------------------------------------------------------------------------------------------------------------------------------------------------------------------------------------------------------------------------------------------------------------------------------------------

>KF861701 Human Peninsular Malaysia

ATGAAGAACTTCATTCTCTTGGCCGTCTCCTCCATCCTGCTGGTGGACTTGCTCCCCACACACTTCGAACATAATGTAGATCTCTCCAGGGCCATAAATGTAAATGGAGTAAGCTTCAATAATGTAGACACCAGTTCACTTGGCGCAGCACAGGTAAGACAAAGTGCTAGCCGAGGCAGAGGACTTGGTGAGAAGCCAAAAGAAGGAGCTGATAAAGAAAAGAAAAAAGGAAAAGAAAAAGAAAAAGAAGAAGAACCAAAGAAGCCAAATGAAAATAAGCTGAAACAACCGGAACAACCAGCAGCAGGAGCAGGGGGCGAACAACCAGCAGCAGGAGCAGGAGGCGAACAACCAGCAGCAGGAGCAGGAGGCGAACAACCAGCAGCAGGAGCAGGAGGCGAACAACCAGCAGCAGGAGCAGGAGGCGAACAACCAGCAGCAGGAGCAAGAGGCGAACAACCAGCAGCAGGAGCAGGAGGCGAACAACCAGCAGCAGGAGCAGGAGGCGAACAACCAGCAGCAGGAGCAGGAGGCGAACAACCAGCAGCAGGAGCAAGAGGCGAACAACCAGCAGCAGGAGCAGGAGGCGAACAACCAGCAGCAGGAGCAGGAGGCGAACAACCAGCACCAGCACCAAGGAGGGAACAACCAGCAGCAGGAGCAGGGGGCGAACAACCAGCACCAGCACCAAGGAGGGAACAACCAGCACCAGGAGCAGGTGCGGGAGATGGAGCACGAGGAGGAAACGCAGGGGCAGGTAAAGGACAGGGACAAAACAATCAGGGTGCGAATGTCCCAAATGAAAAAGTTGTGAATGATTACCTACACAAAATTAGATCTAGCGTTACCACCGAGTGGACTCCATGCAGTGTAACCTGTGGAAATGGTGTAAGAATTAGAAGAAAAGGTCATGCAGGTAATAAAAAGGCAGAGGACCTTACTATGGATGACCTTGAGGTGGAAGCTTGTGTAATGGATAAGTGCGCTGGCATATTTAACGTTGTGAGTAATTCATTAGGCTTAGTCATATTGTTAGTCCTAGCATTATTCAATTAA---------------------------------------------------------------------------------------------------------------------------------------------------------------------------------------------------------------------------------------------------------------------------------------------------------------------------------------------------------------------------------------------------------------------------------------------

>KF861700 Human Peninsular Malaysia

ATGAAGAACTTCATTCTCTTGGCCGTCTCCTCCATCCTGCTGGTGGACTTGCTCCCCACACACTTCGAACATAATGTAGATCTCTCCAGGGCCATAAATGTAAATGGAGTAAGCTTCAATAATGTAGACACCAGTTCACTTGGCGCAGCACAGGTAAGACAAAGTGCTAGCCGAGGCAGAGGACTTGGTGAGAAGCCAAAAGAAGGAGCTGATAAAGAAAAGAAAAAAGAAAAAGAAAAAGAAAAAGAAGAAGAACCAAAGAAGCCAAATGAAAATAAGCTGAAACAACCGGAACAACCAGCAGCAGGAGCAGGGGGCGAACAACCAGCAGCAGGAGCAGGAGGCGAACAACCAGCAGCAGGAGCAGGAGGCGAACAACCAGCAGCAGGAGCAGGAGGCGAACAACCAGCAGCAGGAGCAGGAGGCGAACAACCAGCAGCAGGAGCAAGAGGCGAACAACCAGCAGCAGGAGCAGGAGGCGAACAACCAGCAGCAGGAGCAGGAGGCGAACAACCAGCAGCAGGAGCAGGAGGCGAACAACCAGCAGCAGGAGCAAGAGGCGAACAACCAGCAGCAGGAGCAGGAGGCGAACAACCAGCAGCAGGAGCAGGAGGCGAACAACCAGCACCAGCACCAAGGAGGGAACAACCAGCAGCAGGAGCAGGGGGCGAACAACCAGCACCAGCACCAAGGAGGGAACAACCAGCACCAGGAGCAGGTGCGGGAGATGGAGCACGAGGAGGAAACGCAGGGGCAGGTAAAGGACAGGGACAAAACAATCAGGGTGCGAATGTCCCAAATGAAAAAGTTGTGAATGATTACCTACACAAAATTAGATCTAGCGTTACCACCGAGTGGACTCCATGCAGTGTAACCTGTGGAAATGGTGTAAGAATTAGAAGAAAAGGTCATGCAGGTAATAAAAAGGCAGAGGACCTTACTATGGATGACCTTGAGGTGGAAGCTTGTGTAATGGATAAGTGCGCTGGCATATTTAACGTTGTGAGTAATTCATTAGGCTTAGTCATATTGTTAGTCCTAGCATTATTCAATTAA---------------------------------------------------------------------------------------------------------------------------------------------------------------------------------------------------------------------------------------------------------------------------------------------------------------------------------------------------------------------------------------------------------------------------------------------

>KF861699 Human Peninsular Malaysia

ATGAAGAACTTCATTCTCTTGGCCGTCTCCTCCATCCTGCTGGTGGACTTGCTCCCAACACACTTCGAACATAATGTAGATCTCTCCAGGGCCATAAATGTAAATGGAGTAAGCTTCAATAATGTAGACACCAGTTCACTTGGCGCAGCACAGGTAAGACAAAGTGCTAGCCGAGGCAGAGGACTTGGTGAGAAACCAAAAGAAGGAGCTGATAAAGAAAAGAAAAAAGAAAAAGAAAAAGAAGAAGAACCAAAGAAGCCAAATGAAAATAAGCTGAAACAACCGGAAGGAAATAGGGAAGCTCCAGGACAACCGGAAGGAAATAGGGAAGCTCCAGGACAACCGGAAGGAAATAGGGAAGCTCCAGGACAACCGGAAGGAAATAGGGAAGCTCCAGGACAACCGGAAGGAAATAGGGAAGCTCCAGGACAACCGGAAGGAAATAGGGAAGCTCCAGGACAACCGGAAGGAAATAGGGAAGCTCCAGGACAACCGGAAGGAAATAGGGAAGCTCCAGGACAACCGGAAGGAAATAGGGAAGCTCCAGGACAACCGGAAGGAAATAGGGAAGCTCCAGGACAACCGGAAGGAAATAGGGAAGCTCCAGCACAACCGGAAGGAAATAGGGAAGCTCCAGCACAACCACAAGGAAATGGGGGGGCAGGTCAAGCACAACCACAAAAAAACGAAGGAGGAAACGCAGGAGCACGGAAAGGACAGGGACAAAACAATCAGGGTGCGAATGCCCCAAATGAAAAAGTTGTAAATGATTACCTACAGAAAATTAGATCTAGCGTTACCACCGAGTGGACTCCATGCAGTGTAACCTGTGGAAATGGTGTAAGAATTAAAAGAAAAGCTCATGCAGATAAGAAAAAGGCAGAGGACCTTACTATGGATGACCTTGAAGTGGAAGCTTGTGTAATGGATAAGTGTGCTGGCATATTTAACGTTGTGAGTAATTCATTAGGGTTAGTCATATTGTTAGTCCTAGCATTATTCAATTAA---------------------------------------------------------------------------------------------------------------------------------------------------------------------------------------------------------------------------------------------------------------------------------------------------------------------------------------------------------------------------------------------------------------------------------------------------------------------------------------------

>KF861698 Human Peninsular Malaysia

ATGAAGAACTTCATTCTCTTGGCCGTCTCCTCCATCCTGCTGGTGGACTTGCTCCCCACACACTTCGAACATAATGTAGATCTCTCCAGGGCCATAAATGTAAATGGAGTAAGCTTCAATAATGTAGACACCAGTTCACTTGGCGCAGCACAGGTAAGACAAAGTGCTAGCCGAGGCAGAGGACTTGGTGAGAAACCAAAAGAAGGAGCTGATAAAGAAAAGAAAAAAGAAAAAGAAAAAGAAGAAGAACCAAAGAAGCCAAATGAAAATAAGCTGAAACAACCGGAAGGAAATAGGGAAGCTCCAGGACAACCGGAAGGAAATAGGGAAGCTCCAGGACAACCGGAAGGAAATAGGGAAGCTCCAGGACAACCGGAAGGAAATAGGGAAGCTCCAGGACAACCGGAAGGAAATAGGGAAGCTCCAGGACAACCGGAAGGAAATAGGGAAGCTCCAGGACAACCGGAAGGAAATAGGGAAGCTCCAGGACAACCGGAAGGAAATAGGGAAGCTCCAGCACAACCGGAAGGAAATAGGGAAGCTCCAGGACAACCGGAAGGAAATAGGGAAGCTCCAGCACAACCACAAGGAAATGGGGGGGCAGGTCAAGCACAACCGGAAGGAAATAGGGAAGCTCCAGCACAACCACAAGGAAATGGGGGGGCAGGTCAAGCACAACCACAAAAAAACGAAGGAGGAAACGCAGGAGCACGGAAAGGACAGGGACAAAACAATCAGGGTGCGAATGTCCCAAATGAAAAAGTTGTGAATGATTACCTACACAAAATTAGATCTAGCGTTACCACCGAGTGGACTCCATGCAGTGTAACCTGTGGAAATGGTGTAAGAATTAGAAGAAAAGGTCATGCAGGTAATAAAAAGGCAGAGGACCTTACTATGGATGACCTTGAGGTGGAAGCTTGTGTAATGGATAAGTGCGCTGGCATATTTAACGTTGTGAGTAATTCATTAGGCTTAGTCATATTGTTAGTCCTAGCATTATTCAATTAA------------------------------------------------------------------------------------------------------------------------------------------------------------------------------------------------------------------------------------------------------------------------------------------------------------------------------------------------------------------------------------------------------------------------------------------------------------------------------------------

>KF861697 Human Peninsular Malaysia

ATGAAGAACTTCATTCTCTTGGCCGTCTCCTCCATCCTGCTGGTGGACTTGCTCCCCACACACTTCGAACATAATGTAGATCTCTCCAGGGCCATAAATGTAAATGGAGTAAGCTTCAATAATGTAGACACCAGTTCACTTGGCGCAGCACAGGTGAGACAAAGTGCTAGCCGAGGCAGAGGACTTGGTGAGAAGCCAAAAGAAGGAGCTGATAAAGAAAAGAAAAAAGAAAAAGGAAAAGAAAAAGAAGAAGAACCAAAGAAGCCAAATGAAAATAAGCTGAAACAACCGAATGAAGGACAACCACAAGCACAGGGTGATGGAGCAAATGCAGGACAACCACAAGCACAAGGAGATGGAGCAAATGCAGGACAACCACAAGCACAGGGTGATGGAGCAAATGCAGGGCAACCACAAGCACAGGGTGATGGAGCAAATGCAGGACAACCACAAGCACAAGGAGATGGAGCAAATGCAGGACAACCACAAGCACAGGGTGATGGAGCAAATGCAGGGCAACCACAAGCACAGGGTGATGGAGCAAATGCAGGACAACCACAAGCACAAGGAGATGGAGCAAATGCAGGACAACCACAAGCACAAGGAGATGGAGCAAATGCAGGACAACCACAAGCACAGGGTGATGGAGCAAATGCAGGACAACCACAAGCACAGGGTGATAGGGCGAATGCAGGACAACCACAAGCACAAGGAGATGGGGCAAATGTACCACGACAAGGAAGAAACGGGGGAGGTGCACCAGCAGGAGGAAATGAGGGGAATAAACAAGCAGGAAAAGGACAGGGACAAAACAATCAGGGTGCGAATGCCCCAAATGAAAAAGTTGTGAATGATTACCTACACAAAATTAGATCTAGCGTTACCACCGAGTGGACTCCATGCAGTGTAACCTGTGGAAATGGTGTAAGAATTAGAAGAAAAGCTCATGCAGGTAATAAAAAGGCAGAGGACCTTACTATGGATGACCTTGAGGTGGAAGCTTGTGTAATGGATAAGTGCGCTGGCATATTTAACGTTGTGAGTAATTCATTAGGCTTAGTCATATTGTTAGTCCCAGCATTATTCAATTAA---------------------------------------------------------------------------------------------------------------------------------------------------------------------------------------------------------------------------------------------------------------------------------------------------------------------------------------------------------------------------------------------------------

>KF861696 Human Peninsular Malaysia

ATGAAGAACTTCATTCTCTTGGCCGTCTCCTCCATCCTGCTGGTGGACTTGCTCCCCACACACTTCGAACATAATGTAGATCTCTCCAGGGCCATAAATGTAAATGGAGTAAGCTTCAATAATGTAGACACCAGTTCACTTGGCGCAGCACAGGTGAGACAAAGTGCTAGCCGAGGCAGAGGACTTGGTGAGAAGCCAAAAGAAGGAGCTGATAAAGAAAAGAAAAAAGAAAAAGGAAAAGAAAAAGAAGAAGAACCAAAGAAGCCAAATGAAAATAAGCTGAAACAACCGAATGAAGGACAACCACAAGCACAGGGTGATGGAGCAAATGCAGGACAACCACAAGCACAAGGAGATGGAGCAAATGCAGGACAACCACAAGCACAGGGTGATGGAGCAAATGCAGGGCAACCACAAGCACAGGGTGATGGAGCAAATGCAGGACAACCACAAGCACAAGGAGATGGAGCAAATGCAGGACAACCACAAGCACAGGGTGATGGAGCAAATGCAGGGCAACCACAAGCACAGGGTGATGGAGCAAATGCAGGACAACCACAAGCACAAGGAGATGGAGCAAATGCAGGACAACCACAAGCACAAGGAGATGGAGCAAATGCAGGACAACCACAAGCACAGGGTGATGGAGCAAATGCAGGACAACCACAAGCACAGGGTGATAGGGCGAATGCAGGACAACCACAAGCACAAGGAGATGGGGCAAATGTACCACGACAAGGAAGAAACGGGGGAGGTGCACCAGCAGGAGGAAATGAGGGGAATAAACAAGCAGGAAAAGGACAGGGACAAAACAATCAGGGTGCGAATGCCCCAAATGAAAAAGTTGTGAATGATTACCTACACAAAATTAGATCTAGCGTTACCACCGAGTGGACTCCATGCAGTGTAACCTGTGGAAATGGTGTAAGAATTAGAAGAAAAGCTCATGCAGGTAATAAAAAGGCAGAGGACCTTACTATGGATGACCTTGAGGTGGAAGCTTGTGTAATGGATAAGTGCGCTGGCATATTTAACGTTGTGAGTAATTCATTAGGCTTAGTCATATTGTTAGTCCTAGCATTATTCAATTAA---------------------------------------------------------------------------------------------------------------------------------------------------------------------------------------------------------------------------------------------------------------------------------------------------------------------------------------------------------------------------------------------------------

>KF861695 Human Peninsular Malaysia

ATGAAGAACTTCATTCTCTTGGCCGTCTCCTCCATCCTGCTGGTGGACTTGCTCCCCACACACTTCGAACATAATGTAGATCTCTCCAGGGCCATAAATGTAAATGGAGTAAGCTTCAATAATGTAGACACCAGTTCACTTGGCGCAGCACAGGTGAGACAAAGTGCTAGCCGAGGCAGAGGACTTGGTGAGAAGCCAAAAGAAGGAGCTGATAAAGAAAAGAAAAAAGAAAAAGGAAAAGAAAAAGAAGAACCAAAGAAGCCAAATGAAAATAAGCTGAAACAACCGAATGAAGGACAACCACAAGCACAGGGTGATGGAGCAAATGCAGGACAACCACAAGCACAAGGAGATGGAGCAAATGCAGGACAACCACAAGCACAGGGTGATGGAGCAAATGCAGGGCAACCACAAGCACAGGGTGATGGAGCAAATGCAGGACAACCACAAGCACAAGGAGATGGAGCAAATGCGGGACAACCACGAGCACAGGGTGATGGAGCAAATGCAGGGCAACCACAAGCACAGGGTGATGGAGCAAATGCAGGACAACCACAAGCACAAGGAGATGGAGCAAATGCAGGACAACCACAAGCACAAGGAGATGGAGCAAATGCAGGACAACCACAAGCACAGGGTGATGGAGCAAATGCAGGACAACCACAAGCACAGGGTGATAGGGCGAATGCAGGACAACCACAAGCACAAGGAGATGGGGCAAATGTACCACGACAAGGAAGAAACGGGGGAGGTGCACCAGCAGGAGGAAATGAGGGGAATAAACAAGCAGGAAAAGGACAGGGACAAAACAATCAGGGTGCGAATGCCCCAAATGAAAAAGTTGTGAATGATTACCTACACAAAATTAGATCTAGCGTTACCACCGAGTGGACTCCATGCAGTGTAACCTGTGGAAATGGTGTAAGAATTAGAAGAAAAGCTCATGCAGGTAATAAAAAGGCAGAGGACCTTACTATGGATGACCTTGAGGTGGAAGCTTGTGTAATGGATAAGTGCGCTGGCATATTTAACGTTGTGAGTAATTCATTAGGCTTAGTCATATTGTTAGTCCTAGCATTATTCAATTAA------------------------------------------------------------------------------------------------------------------------------------------------------------------------------------------------------------------------------------------------------------------------------------------------------------------------------------------------------------------------------------------------------------

>EU708437 Human Peninsular Malaysia

ATGAAGAACTTCATTCTCTTGGCCGTCTCCTCCATCCTGCTGGTGGACTTGCTCCCCACACACTTCGAACATAATGTAGATCTCTCCAGGGCCATAAATCTAAATGGAGTAAGCTTCAATAATGTAGACACCAGTTCACTTGGCGCAGCACAGGTAAGACAAAGTGCTAGCCGAGGCAGAGGACTTGGTGAGAAACCAAAAGAAGGAGCTGATAAAGAAAAGAAAAAAGAAAAAGATAAAGAAGAAGAACCAAAGAAGCCAAATGAAAATAAGCTGAAACAACCGAATGCAGGACAACCACAAGCACAGGGTGATGGAGCAAATGCAGGACAACCACAAGCACAAGGAGATGGAGCAAATGCAGGACAACCACAAGCACAGGGTGATGGAGCAAATGCAGGACAACCACAAGCACAAGGAGATGGAGCAAATGCAGGACAACCACAAGCACAAGGAGATGGAGCAAATGCAGGACAACCACAAGCACAAGGAGATGGAGCAAATGCAGGACAACCACAAGCACAGGGTGATGGAGCAAATGCAGGACAACCACAAGCACAGGGTGATGGAGCAAATGCAGGACAACCACAAGCACAGGGTGATGGAGCAAATGCAGGACAACCACAAGCACAGGGTGATAGGGCGAATGCAGGACAACCACAAGCACAAGGAGATGGGGCAAATGTACCACGACAAGGAAGAAACGGGGGAGGTGCACCAGCAGGAGGAAATGAGGGGAATAAACAAGCAGGAAAAGGACAGGGACAAAACAATCAGGGTGCGAATGCCCCAAATGAAAAAGTTGTAAATGATTACCTACAGAAAATTAGATCTAGCGTTACCACCGAGTGGACTCCATGCAGTGTAACCTGTGGAAATGGTGTAAGAATTAGAAGAAGAGCTCATGCAGATAAGAAAAAGGCAGAGGACCTTACTATGGATGACCTTGAGGTGGAAGCTTGTGTAATGGATAAGTGCGCTGGCATATTTAACGTTGTGAGTAATTCATTAGGGTTAGTCATATTGTTAGTCCTAGCATTATTCAATTAA---------------------------------------------------------------------------------------------------------------------------------------------------------------------------------------------------------------------------------------------------------------------------------------------------------------------------------------------------------------------------------------------------------------------------------------------------

>EU687470 Human Peninsular Malaysia

ATGAAGAACTTCATTCTCTTGGCCGTCTCCTCCATCCTGCTGGTGGACTTGCTCCCCACACACTTCGAACATAATGTAGATCTCTCCAGGGCCATAAATGTAAATGGAGTAAGCTTCAATAATGTAGACACCAGTTCACTTGGCGCAGCACGGGTAAGACAAAGTGCTAGCCGAGGCAGAGGACTTGGTGAGAAGCCAAAAGAAGGAGCTGATAAAGAAAAGAAAAAAGAAAAAGAAAAAGAAAAAGAAGAAGAACCAAAGAAGCCAAATGAAAATAAGCTGAAACAACCGGAACAACCAGCAGCAGGAGCAGGGGGCGAACAACCAGCAGCAGGAGCAGGAGGCGAACAACCAGCAGCAGGAGCAGGAGGCGAACAACCAGCAGCAGGAGCAGGAGGCGAACAACCAGCAGCAGGAGCAAGAGGCGAACAACCAGCAGCAGGAGCAGGAGGCGAACAACCAGCAGCAGGAGCAGGAGGCGAACAACCAGCAGCAGGAGCAGGAGGCGAACAACCAGCAGCAGGAGCAAGAGGCGAACAACCAGCAGCAGGAGCAGGAGGCGAACAACCAGCAGCAGGAGCAAGAGGCGAACAACCAGCAGCAGGAGCAGGAGGCGAACAACCAGCAGCAGGAGCAGGAGGCGAACAACCAGCAGCAGGAGCAAGAGGCGAACAACCAGCACCAGCACCAAGGAGGGAACAACCAGCACCAGGAGCAGGTGCGGGAGATGGAGCACGAGGAGGAAACGCAGGGGCAGGTAAAGGACAGGGACAAAACAATCAGGGTGCGAATGTCCCAAATGAAAAAGTTGTGAATGATTACCTACACAAAATTAGATCTAGCGTTACCACCGAGTGGACTCCATGCAGTGTAACCTGTGGAAATGGTGTAAGAATTAGAAGAAAAGGTCATGCAGGTAATAAAAAGGCAGAGGACCTTACTATGGATGACCTTGAGGTGGAAGCTTGTGTAATGGATAAGTGCGCTGGCATATTTAACGTTGTGAGTAATTCATTAGGCTTAGTCATATTGTTAGTCCTAGCATTATTCAATTAA---------------------------------------------------------------------------------------------------------------------------------------------------------------------------------------------------------------------------------------------------------------------------------------------------------------------------------------------------------------------------------------------------------------------------------------------

>EU687469 Human Peninsular Malaysia

ATGAAGAACTTCATTCTCTTGGCCGTCTCCTCCATCCTGCTGGTGGACTTGCTCCCCACACACTTCGAACATAATGTAGATCTCTCCAGGGCCATAAATGTAAATGGAGTAAGCTTCAATAATGTAGACACCAGTTCACTTGGCGCAGCACAGGTGAGACAAAGTGCTAGCCGAGGCAGAGGACTTGGTGAGAAGCCAAAAGAAGGAGCTGATAAAGAAAAGAAAAAAGAAAAAGGAAAAGAAAAAGAAGAAGAACCAAAGAAGCCAAATGAAAATAAGCTGAAACAACCGAATGAAGGACAACCACAAGCACAGGGTGATGGAGCAAATGCAGGGCAACCACAAGCACAGGGTGATGGAGCAAATGCAGGACAACCACAAGCACAAGGAGATGGAGCAAATGCAGGACAACCACAAGCACAGGGTGATGGAGCAAATGCAGGACAACCACAAGCACAGGGTGATGGAGCAAATGCAGGACAACCACAAGCACAAGGAGATGGAGCAAATGCAGGACAACCACAAGCACAGGGTGATGGAGCAAATGCAGGGCAACCACAAGCACAGGGTGATGGAGCAAATGCAGGACAACCACAAGCACAAGGAGATGGAGCAAATGCAGGACAACCACAAGCACAAGGAGATGGAGCAAATGCAGGACAACCACAAGCACAGGGTGATGGAGCAAATGCAGGACAACCACAAGCACAGGGTGATAGGGCGAATGCAGGACAACCACAAGCACAAGGAGATGGGGCAAATGTACCACGACAAGGAAGAAACGGGGGAGGTGCACCAGCAGGAGGAAATGAGGGGAATAAACAAGCAGGAAAAGGACAGGGACAAAACAATCAGGGTGCGAATGCCCCAAATGAAAAAGTTGTGAATGATTACCTACACAAAATTAGATCTAGCGTTACCACCGAGTGGACTCCATGCAGTGTAACCTGTGGAAATGGTGTAAGAATTAGAAGAAAAGCTCATGCAGGTAATAAAAAGGCAGAGGACCTTACTATGGATGACCTTGAGTTGGAAGCTTGTGTAATGGATAAGTGCGCTGGCATATTTAACGTTGTGAGTAATTCATTAGGCTTAGTCATATTGTTAGTCCTAGCATTATTCAATTAA---------------------------------------------------------------------------------------------------------------------------------------------------------------------------------------------------------------------------------------------------------------------------------------------------------------------------------------------------------------------

>EU687468 Human Peninsular Malaysia

ATGAAGAACTTCATTCTCTTGGCCGTCTCCTCCATCCTGCTGGTGGACTTGCTCCCCACATACTTCGAACATAATGTAGATCTCTCCAGGGCCATAAATGTAAATGGAGTAAGCTTCAATAATGTAGACACCAGTTCACTTGGCGCAGCACAGGTAAGACAAAGTGCTAGCCGAGGCAGAGGACTTGGTGAGAAGCCAAAAGAAGGAGCTGATAAAGAAAAGAAAAAAGAAAAAGAAAAAGAAAAAGAAGAAGAACCAAAGAAGCCAAATGAAAATAAGCTGAAACAACCAGGGGGCGAACAAGCAGGACCAGGGGGCGAACAAGCAGGACCAGGAGGCGAACAAGCAGGACCAGGAGGCGAACAAGCAGGACCAAGACCAGGGGGCGAACAAGCAGGACCAGGAGGCGAACAAGCAGGACCAAGACCAGGGGGCGAACAAGCAGGACCAGGAGGCGAACAAGCAGGACCAAGACCAGGGGGCGAACAAGCAGGACCAGGAGGCGAACAAGCAGGACCAAGACCAGGGGGCGAACAAGCAGGACCAGGAGGCGAACAAGCAGGACCAAGACCAGGGGGCGAACAAGCAGGACCAGGAGGCGAACAAGCAGGACCAAGACCAGGGGGCGAACAAGCAGGACCAGGGGGCGAACAACCAGCACCAAGACCAGGGGGAGAACAACCAGCACCAGCACCAAGGAGGGAACAACCAGCACCAGGACCAGGGGGCGAACAACCAGCACCAGGAGCAGGTGCGGGAGATGGAGCACGAGGAGGAAACGCAGGGGCAGGTAAAGGACAGGGACAAAACAATCAGGGTGCGAATGTCCCAAATGAAAAAGTTGTGAATGATTACCTACACAAAATTAGATCTAGCGTTACCACCGAGTGGACTCCATGCAGTGTAACCTGTGGAAATGGTGTAAGAATTAGAAGAAGACAGAATGCTGGTAATAAAAAGGCAGAGGACCTTACTATGGATGACCTTGAGGTGGAAACTTGTGTAATGGATAAGTGCGCTGGCATATTTAACGTTGTGAGTAATTCATTAGGGTTAGTCATATTGTTAGTCCTAGCATTATTCAATTAA------------------------------------------------------------------------------------------------------------------------------------------------------------------------------------------------------------------------------------------------------------------------------------------------------------------------------------------------------------------------------------------------------------

>EU687467 Human Peninsular Malaysia

ATGAAGAACTTCATTCTCTTGGCCGTCTCCTCCATCCTGCTGGTGGACTTGCTCCCCACACACTTCGAACATAATGTAGATCTCTCCAGGGCCATAAATGTAAATGGAGTAAGCTTCAATAATGTAGACACCAGTTCACTTGGCGCAGCACAGGTGAGACAAAGTGCTAGCCGAGGCAGAGGACTTGGTGAGAAGCCAAAAGAAGGAGCTGATAAAGAAAAGAAAAAAGAAAAAGAAAAAGAAGAAGAACCAAAGAAGCCAAATGAAAATAAGCTGAAACAACCTAATGCAGAAGGTGATGGAGCAAATGCACGACAACCTAATGCAGAAGGTGATGGAGCAAATGCACGACAACCGAATGCAGAAGGTGATGGAGCAAATGCACGACAACCTAATGCAGAAGGTGATGGAGCAAATGCACGACAACCGAATGCAGAAGGTGATGGAGCAAATGCACGACAACCTAATGCAGAAGGTGATGGAGCAAATGCACGACAACCTAATGCAGAAGGTGATGGAGCAAATGCACGACAACCTAATGCAGAAGGTGATGGAGCAAATGCACGACAACCGAATGCAGAAGGTGATGGAGCAAATGCACGACAACCTAATGCAGAAGGTGATGGAGCAAATGCACGACAACCGAATGCAGAAGGTGGTGGAGCAAATGCACGACAGCCACAGGCAGAAGGTGATGGAGCAAATGCACGACAACCACAAGCACAAGGAGATGGAGGAAATGCACGACAAGGAGGAAACGGGGGAGGTGCACCAGCAGGAGGAAATGAGGGGAATAAACAAGCAGGAAAAGGACAGGGACAAAACAATCAGGGTGCGAATGCCCCAAATGAAAAAGTTGTAAATGATTACCTACAGAAAATTAGATCTAGCGTTACCACCGAGTGGACTCCATGCAGTGTAACCTGTGGAAATGGTGTAAGAATTAGAAGAAGAGCTCATGCAGATAAGAAAAAGGCAGAGGACCTTACTATGGATGACCTTGAAGTGGAAGCTTGTGTAATGGATAAGTGTGCTGGCATATTTAACGTTGTGAGTAATTCATTAGGGTTCGTCATATTGTTAGTCCTAGCATTATTCAATTAA---------------------------------------------------------------------------------------------------------------------------------------------------------------------------------------------------------------------------------------------------------------------------------------------------------------------------------------------------------------------------------------------

>AY327572 Human Sarawak

ATGAGGAACTTCATTCTCTTGGCCGTCTCCTCCATCCTGCTGGTGGACTTGCTCCCCACACACTTCGAACATAATGTAGATCTCTCCAGGGCCATAAATGTAAATGGAGTAAGCTTCAATAATGTAGACACCAGTTCACTTGGCGCAGCACAGGTAGGACAAAGTGCTAGCCGAGGCAGAGGACTTGGTGAAAAGCCAAAAGAAGGAGCTGATAAAGAAAAAAAAAAAGAAAAAGAAGAACCAAAGAAGCCAAATGAAAATAAGCTGAAACAACCACAGGTAGAAGGTGATGGAGCAAATGCACGACAACCACAGGCAGAAGGTGATGGAGCAAATGCACGACAGCCACAGGCAGAAGGCGATGGAGCAAATGCACGACAACCACAGGCAGAAGGTGATGGAGCAAATGCACGACAGCCACAGGCAGAAGGTGATGGAGCAAATGCACGACAACCACAGGCAGAAGGTGATGGAGCAAATGCACGACAACCACAGGCAGAAGGTGATGGAGCAAATGCACGACAACCACAGGCAGAAGGTGATGGAGCAAATGCACGACAACCACAGGCAGAAGGTGATGGAGCAAATGCACGACAGCCACAGGCAGAAGGTGGTGGAGCAAATGCACGACAGCCACAGGCAGAAGGTGGTGGAGCAAATGCACGACAGCCACAGGCAGAAGGTGATGGAGCAAATGCACGACAACCACAAGCACAAGGAGATGGAGGAAATGCACGACAAGGAGGAAACGGGGGAGGTGCACCAGCAGGAGGAAATGAGGGGAATAAACAAGCAGGAAAAGGACAGGGACAAAACAATCAGGGTGCGAATGCCCCAAATGAAAAAGTTGTAAATGATTACCTACAGAAAATTAGATCTAGCGTTACCACCGAGTGGACTCCATGCAGTGTAACCTGTGGAAATGGTGTAAGAATTAGAAGAAGAGCTCATGCAGATAAGAAAAAGGCAGAGGACCTTACTATGGATGACCTTGAAGTGGAAGCTTGTGTAATGGATAAGTGTGCTGGCATATTTAACGTTGTGAGTAATTCATTAGGGTTAGTCATATTGTTAGTCCTAGCATTATTCAAT---------------------------------------------------------------------------------------------------------------------------------------------------------------------------------------------------------------------------------------------------------------------------------------------------------------------------------------------------------------------------------------------------------

>AY327570 Human Sarawak

ATGAAGAACTTCATTCTCTTGGCCGTCTCCTCCATCCTGCTGGTGGACTTGCTCCCCACACACTTCGAACATAATGTAGATCTCTCCAGGGCCATAAATGTAAATGGAGTAAGCTTCAATAATGTAGACACCAGTTCACTTGGCGCAGCACAGGTAAGACAAAGTGCTAGCCGAGGCAGAGGACTTGGTGAGAAGCCAAAAGAAGGAGATGATAAAGAAAAGAAAAAAGAAAAAGAAAAAGAAGAAGAACCAAAGAACCTAAATGAAAATAAGCCGAAACAACCGAATGCAGAAGGTGATGGAGCTAAGCCGAAACAACCGAATGCAGAAGGTGATGGAGCTAAGCTGAAACAACCGAATGCAGAAGGTGATGGAGCTAAGCTGAAACAACCGAATGCAGAAGGTGATGGAGGAAATGCACGACAACCGAATGCAGAAGGTGATGGAGGAAATGCACGACAACCGAATGCAGAAGGTGATGGAGGAAATGCACGACAACCGAATGCAGAAGGTGATGGAGGAAATGCACGACAACCGAATGCAGAAGGTGATGGAGGAAATGCACGACAACCGAATGCAGAAGGTGATGGAGGAAATGCACGACAACCTAATGCAGAAGGTGATGGAGGAAATGCACGACAACCGAATGCAGAAGGTGATGGAGGAAATGCACGACAACCGAATGCAGAAGGTGATGGAGGAAATGCACGACAACCTAATGCAGAAGGTGATGGAGGAAATGCACGACAACCTAATGCAGAAGGTGATGGAGGAAATGCACGACAACCTAATGCAGAAGGTGATGGAGGAAATGCACGACAACCGAATGCAGAAGGTGATGGAGCAAATGCACGACAGCCACAGGCAGAAGGTGGTGGAGGAAATGCACGACAAGGAGGAAATGAGGGGAATAAACAAGCAGGAAAAGGACAGGGACAAAACAATCAGGGTGCGAATGCCCCAAATGAAAAAGTTGTAAATGATTACCTACAGAAAATTAGATCTAGCGTTACCACCGAGTGGACTCCATGCAGTGTAACCTGTGGAAATGGTGTAAGAATTAGAAGAAGAGCTCATGCAGATAAGAAAAAGGCAGAGGACCTTACTATGGATGACCTTGAAGTGGAAGCTTGTGTAATGGATAAGTGTGCTGGCATATTTAACGTTGTGAGTAATTCATTAGGGTTAGTCATATTGTTAGTCCTAGCATTATTCAAT---------------------------------------------------------------------------------------------------------------------------------------------------------------------------------------------------------------------------------------------------------------------------

>AY327568 Human Sarawak

ATGAAGAACTTCATTCTCTTGGCCGTCTCCTCCATCCTGCTGGTGGACTTGCTCCCCACACACTTCGAACATAATGTAGATCTCTCCAGGGCCATAAATATAAATGGAGTAAGCTTCAATAATGTAGACACCAGTTCACTTGGCGCAGCACAGGTAAGACAAAGTGCTAGCCGAGGCAGAGGACTTGGTGAAAAGCCAAAAGAAGGAGCTGATAAAGAAAAAAAAAAAGAAAAAGAAGAACCAAAGAAGCCAAATGAAAATAAGCTGAAACAACCGAATGCAGAAGGTGGAGCAAATGCAGGACAACCGAATGCAGAAGGTGGAGCAAATGCAGGACAACCGAATGCAGAAGGTGGAGCAAATGCAGGACAACCTAATGCAGAAGGTGGAGCAAATGCAGGACAACCGAATGCAGAAGGTGGAGCAAATGCAGGACAACCTAATGCAGAAGGTGGAGCAAATGCAGGACAACCGAATGCAGAAGGTGGAGCAAATGCAGGACAACCGAATGCAGAAGGTGGAGCAAATGCACGACAACCGAATGCAGAAGGTGGAGCAAATGCAGGACAACCGAATGCAGAAGGTGGAGCAAATGCAGGACAACCGAATGCAGAAGGTGGAGCAAATGCACGACAACCGAATGCAGAAGGTGGAGCAAATGCAGGACAACCGAATGCAGAAGGTGGAGCAAATGCAGGACAACCGAATGCAGAAGGTGGAGCAAATGCACGACAGCCACAGGCAGAAGGTGGTGGAGCAAATGCACGACAAGGAGGAAATGAGGGGAATAAACAAGCAGGAAAAGGACAGGGACAAAACAATCAGGGTGCGAATGCCCCAAATGAAAAAGTTGTAAATGATTACCTACAGAAAATTAGATCTAGCGTTACCACCGAGTGGACTCCATGCAGTGTAACCTGTGGAAATGGTGTAAGAATTAGAAGAAGAGCTCATGCAGATAAGAAAAAGGCAGAGGACCTTACTATGGATGACCTTGAAGTGGAAGCTTGTGTAATGGATAAGTGCGCTGGCATATTTAACGTTGTGAGTAATTCATTAGGCTTAGTCATATTGTTAGTCCTAGCATTATTCAAT------------------------------------------------------------------------------------------------------------------------------------------------------------------------------------------------------------------------------------------------------------------------------------------------------------------------------------------------------------------------------------------------------

>AY327566 Human Sarawak

ATGAAGAACTTCATTCTCTTGGCCGTCTCCTCCATCCTGCTGGTGGACTTGCTCCCCACACACTTCGAACATAATGTAGATCTCTCCAGGGCCATAAATGTAAATGGAGTAAGCTTCAATAATGTAGACACCAGTTCACTTGGCGCAGCACAGGTAAGACAAAGTGCTAGCCGAGGCAGAGGACTTGGTGAGAAGCCAAAAGAAGGAGATGATAAAGAAAAGAAAAAAGAAAAAGAAAAAGAAGAAGAACCAAAGAACCTAAATGAAAATAAGCCGAAACAACCGAATGCAGAAGGTGATGGAGCTAAGCCGAAACAACCGAATGCAGAAGGTGATGGAGCTAAGCTGAAACAACCGAATGCAGAAGGTGATGGAGCTAAGCTGAAACAACCGAATGCAGAAGGTGATGGAGGAAATGCACGACAACCGAATGCAGAAGGTGATGGAGGAAATGCACGACAACCGAATGCAGAAGGTGATGGAGGAAATGCACGACAACCGAATGCAGAAGGTGATGGAGGAAATGCACGACAACCGAATGCAGAAGGTGATGGAGGAAATGCACGACAACCGAATGCAGAAGGTGATGGAGGAAATGCACGACAACCTAATGCAGAAGGTGATGGAGGAAATGCACGACAACCGAATGCAGAAGGTGATGGAGGAAATGCACGACAACCGAATGCAGAAGGTGATGGAGGAAATGCACGACAACCTAATGCAGAAGGTGATGGAGGAAATGCACGACAACCTAATGCAGAAGGTGATGGAGGAAATGCACGACAACCTAATGCAGAAGGTGATGGAGGAAATGCACGACAACCGAATGCAGAAGGTGATGGAGCAAATGCACGACAGCCACAGGCAGAAGGTGGTGGAGGAAATGCACGACAAGGAGGAAATGAGGGGAATAAACAAGCAGGAAAAGGACAGGGACAAAACAATCAGGGTGCGAATGCCCCAAATGAAAAAGTTGTAAATGATTACCTACAGAAAATTAGATCTAGCGTTACCACCGAGTGGACTCCATGCAGTGTAACCTGTGGAAATGGTGTAAGAATTAGAAGAAGAGCTCATGCAGATAAGAAAAAGGCAGAGGACCTTACTATGGATGACCTTGAAGTGGAAGCTTGTGTAATGGATAAGTGTGCTGGCATATTTAACGTTGTGAGTAATTCATTAGGGTTAGTCATATTGTTAGTCCTAGCATTATTCAAT---------------------------------------------------------------------------------------------------------------------------------------------------------------------------------------------------------------------------------------------------------------------------

>AY327564 Human Sarawak

ATGAGGAACTTCATTCTCTTGGCCGTCTCCTCCATCCTGCTGGTGGACTTGCTCCCCACACACTTCGAACATAATGTAGATCTCTCCAGGGCCATAAATGTAAATGGAGTAAGCTTCAATAATGTAGACACCAGTTCACTTGGCGCAGCACAGGTAGGACAAAGTGCTAGCCGAGGCAGAGGACTTGGTGAAAAGCCAAAAGAAGGAGCTGATAAAGAAAAAAAAAAAGAAAAAGAAGAACCAAAGAAGCCAAATGAAAATAAGCTGAAACAACCACAGGCAGAAGGTGATGGAGCAAATGCACGACAACCACAGGCAGAAGGTGATGGAGCAAATGCACGACAGCCACAGGCAGAAGGCGATGGAGCAAATGCACGACAACCACAGGCAGAAGGTGATGGAGCAAATGCACGACAGCCACAGGCAGAAGGTGATGGAGCAAATGCACGACAACCACAGGCAGAAGGTGATGGAGCAAATGCACGACAACCACAGGCAGAAGGTGATGGAGCAAATGCACGACAACCACAGGCAGAAGGTGATGGAGCAAATGCACGACAACCACAGGCAGAAGGTGATGGAGCAAATGCACGACAGCCACAGGCAGAAGGTGGTGGAGCAAATGCACGACAGCCACAGGCAGAAGGTGGTGGAGCAAATGCACGACAGCCACAGGCAGAAGGTGATGGAGCAAATGCACGACAACCACAAGCACAAGGAGATGGAGGAAATGCACGACAAGGAGGAAACGGGGGAGGTGCACCAGCAGGAGGAAATGAGGGGAATAAACAAGCAGGAAAAGGACAGGGACAAAACAATCAGGGTGCGAATGCCCCAAATGAAAAAGTTGTAAATGATTACCTACAGAAAATTAGATCTAGCGTTACCACCGAGTGGACTCCATGCAGTGTAACCTGTGGAAATGGTGTAAGAATTAGAAGAAGAGCTCATGCAGATAAGAAAAAGGCAGAGGACCTTACTATGGATGACCTTGAAGTGGAAGCTTGTGTAATGGATAAGTGTGCTGGCATATTTAACGTTGTGAGTAATTCATTAGGGTTAGTCATATTGTTAGTCCTAGCATTATTCAAT---------------------------------------------------------------------------------------------------------------------------------------------------------------------------------------------------------------------------------------------------------------------------------------------------------------------------------------------------------------------------------------------------------

>AY327562 Human Sarawak

ATGAAGAACTTCATTCTCTTGGCCGTCTCCTCCATCCTGCTGGTGGACTTGTTCCCCACACACTTCGAACATAATGTAGATCTCTCCAGGGCCATAAATGTAAATGGAGTAAGCTTCAATAATGTAGACACCAGTTCACTTGGCGCAGCACAGGTAAGACAAAGTGCTAGCCGAGGCAGAGGACTTGGTGAGAAGCCAAAAGAAGGAGCTGATAAAGAAAAGAAAAAAGAAAAAGAAAAAGAAGAACCAAAGAAGCCAAATGAAAATAAGCTGAAACAACCGGAAGGAAATCAAGATGGGCGAGCACAACCGGAAGGAAATCAGGATGGTCGAGCGCAACCGGAAGGAAATCAGGATGGTCGAGCGCAGCCGGAAGGAAATCAAGATGGGCGAGCACAACCGGAAGGAAATCAGGATGGTCGAGCGCAACCGGAAGGAAATCAGGATGGTCGAGCGCAGCCGGAAGGAAATCAGGATGGTCGAGCGCAGCCGGAAGGAAATCAGGATGGGCGAGCGCAACCGGAAGGAAATCAGGATGGACGAGCACAACCGGAAGGAAATAGGGAAGCTCCAGCACAACCACAAGGAAATGGGGGGGCAGGTCAAGCACAACCGGAAGGAAATAGGGAAGCTCCAGCACAACCACAAGGAAATGGGGGGGCAGGTCAAGCACAACCGGAAGGAAATAGGGAAGCTCCAGCACAACCACAAGGAAATGGGGGGGCAGGTCAAGCACAACCACAAAAAAACGAAGGAGGAAACGCAGGAGCACGGAAAGGACAGGGACAAAACAATCAGGGTGCGAATGCCCCAAATGAAAAAGTTGTAAATGATTACCTACAGAAAATTAGATCTAGCGTTACCACCGAGTGGACTCCATGCAGTGTAACCTGTGGAAATGGTGTAAGAATTAGAAGAAGAGCTCATGCAGATAAGAAAAAGGCAGAGGACCTTACTATGGATGACCTTGAAGTGGAAGCTTGTGTAATGGATAAGTGCGCTGGCATATTTAACGTTGTGAGTAATTCATTAGGGTTAGTCATATTGTTAGTCCTAGCATTATTCAAT---------------------------------------------------------------------------------------------------------------------------------------------------------------------------------------------------------------------------------------------------------------------------------------------------------------------------------------------------------------------------------------------------------------------------------

>AY327560 Human Sarawak

ATGAAGAACTTCATTCTCTTGGCCGTCTCCTCCATCCTGCTGGTGGACTTGTTCCCCACACACTTCGAACATAATGTAGATCTCTCCAGGGCCATAAATGTAAATGGAGTAAGCTTCAATAATGTAGACACCAGTTCACTTGGCGCAGCACAGGTGAGACAAAGTGCTAGCCGAGGCAGAGGACTTGGTGAGAAGCCAAAAGAAGGAGCTGATAAAGAAAAGAAAAAAGAAAAAGAAGAAAAACCAAAGAAGCCAAATGAAAATAAGCTGAAACAACCAGGGGGCGAACAACCAGCAGCAGGGGGCGAACAACCAGCAGCAGGGGGCGAACAACCAGCAGCAGGGGGTGAACAACCAGCAGCAGGGGGTGAACAACCAGCAGCAGGGGGTGAACAACCAGCAGCAGGGGGTGAACAACCAGCAGCAGGGGGCGAACGACCAGCAGCAGGGGGCGAACAACCAGCAGCAGGGGGTGAACAACCAGCAGCAGGGGGCGAACGACCAGCAGCAGGGGGTGAACAACCAGCAGCAGGGGGCGAACGACCAGCAGCAGGGGGCGAACAACCAGCAGCAGGGGGCGAACAACCAGCAGCAGGGGGTGAACAACCAGCACCAGCACCAAGGAGGGAACAACCAGCAGCAGGGGGCGAACAACCAGCACCAGCACCAAGGAGGGAACAACCAGCAGCAGGAGCAGGTGCGGGAGATGGAGCACGAGGAGGAAACGCAGGGGCAGGTAAAGGACAGGGACAAAACAATCAGGGTGCGAATGTCCCAAATGAAAAAGTTGTGAATGATTACCTACACAAAATTAGATCTAGCGTTACCACCGAGTGGACTCCATGCAGTGTAACCTGTGGAAATGGTGTAAGAATTAGAAGAAGACAGAATGCTGGTAATAAAAAGGCAGAGGACCTTACTATGGATGACCTTGAGGTGGAAGCTTGTGTAATGGATAAGTGCGCTGGCATATTTAACGTTGTGAGTAATTCATTAGGGTTAGTCATATTGTTAGTCCTAGCATTATTCAAT---------------------------------------------------------------------------------------------------------------------------------------------------------------------------------------------------------------------------------------------------------------------------------------------------------------------------------------------------------------------------------------------------------------------------------------------------------------------

>AY327558 Human Sarawak

ATGAGGAACTTCATTCTCTTGGCCGTCTCCTCCATCCTGCTGGTGGACTTGCTCCCCACACACTTCGAACATAATGTAGATCTCTCCAGGGCCATAAATGTAAATGGAGTAAGCTTCAATAATGTAGACACCAGTTCACTTGGCGCAGCACAGGTAAGACAAAGTGCTAGCCGAGGCAGAGGACTTGGTGAGAAGCGAAAAGAAGGAGCTGATAAAGAAAAGAAAAAAGAAAAAGAAGAAGAACCAAAGAAGCCAAATGAAAATAAGCTGAAACAACCGAATGAAGGACAACCACAAGCACAGGGTGATGGAGCAAATGCAGGACAACCACAAGCACAAGGAGATGGAGCAAATGCAGGACAACCACAAGCACAGGGTGATGGAGCAAATGCAGGACAACCACAAGCACAAGGAGATGGAGCAAATGCAGGACAACCACAAGCACAAGGAGATGGAGCAAATGCAGGACAACCACAAGCACAGGGTGATGGAGCAAATGCAGGACAACCACAAGCACAAGGAGATGGAGCAAATGCAGGACAACCACAAGCACAAGGAGATGGAGCAAATGCAGGACAACCACAAGCACAGGGTGATGGAGCAAATGCAGGACAACCACAAGCACAGGGTGATGGAGCAAATGCAGGACAACCACAAGCACAGGGTGATAGGGCGAATGCAGGACAACCACAAGCACAAGGAGATGGGGCAAATGTACCACGACAAGGAAGAAACGGGGGAGGTGCACCAGCAGGAGGAAATGAGGGGAATAAACAAGCAGGAAAAGGACAGGGACAAAACAATCAGGGTGCGAATGCCCCAAATGAAAAAGTTGTGAATGATTACCTACACAAAATTAGATCTAGCGTTACCACCGAGTGGACTCCATGCAGTGTAACCTGTGGAAATGGTGTAAGAATTAGAAGAAAAGCTCATGCAGGTAATAAAAAGGCAGAGGACCTTACTATGGATGACCTTGAGGTGGAAGCTTGTGTAATGGATAAGTGCGCTGGCATATTTAACGTTGTGAGTAATTCATTAGGGTTAGTCATATTGTTAGTCCTAGCATTATTCAAT------------------------------------------------------------------------------------------------------------------------------------------------------------------------------------------------------------------------------------------------------------------------------------------------------------------------------------------------------------------------------------------------------------------------

>GU002533 Human Sarawak

ATGAAGAACTTCATTCTCTTGGCCGTCTCCTCCATCCTGCTGGTGGACTTGCTCCCCACACACTTCGAACATAATGTAGATCTCTCCAGGGCCATAAATGTAAATGGAGTAAGCTTCAATAATGTAGACACCAGTTCACTTGGCGCAGCACAGGTAAGACAAAGTGCTAGCCGAGGCAGAGGACTTGGTGAGAAGCCAAAAGAAGGAGATGATAAAGAAAAGAAAAAAGAAAAAGAAAAAGAAGAAGAACCAAAGAACCTAAATGAAAATAAGCCGAAACAACCGAATGCAGAAGGTGATGGAGCTAAGCCGAAACAACCGAATGCAGAAGGTGATGGAGCTAAGCTGAAACAACCGAATGCAGAAGGTGATGGAGCTAAGCTGAAACAACCGAATGCAGAAGGTGATGGAGGAAATGCACGACAACCGAATGCAGAAGGTGATGGAGGAAATGCACGACAACCGAATGCAGAAGGTGATGGAGGAAATGCACGACAACCGAATGCAGAAGGTGATGGAGGAAATGCACGACAACCGAATGCAGAAGGTGATGGAGGAAATGCACGACAACCGAATGCAGAAGGTGATGGAGGAAATGCACGACAACCTAATGCAGAAGGTGATGGAGGAAATGCACGACAACCGAATGCAGAAGGTGATGGAGGAAATGCACGACAACCGAATGCAGAAGGTGATGGAGGAAATGCACGACAACCTAATGCAGAAGGTGATGGAGGAAATGCACGACAACCTAATGCAGAAGGTGATGGAGGAAATGCACGACAACCTAATGCAGAAGGTGATGGAGGAAATGCACGACAACCGAATGCAGAAGGTGATGGAGCAAATGCACGACAGCCACAGGCAGAAGGTGGTGGAGGAAATGCACGACAAGGAGGAAATGAGGGGAATAAACAAGCAGGAAAAGGACAGGGACAAAACAATCAGGGTGCGAATGCCCCAAATGAAAAAGTTGTAAATGATTACCTACAGAAAATTAGATCTAGCGTTACCACCGAGTGGACTCCATGCAGTGTAACCTGTGGAAATGGTGTAAGAATTAGAAGAAGAGCTCATGCAGATAAGAAAAAGGCAGAGGACCTTACTATGGATGACCTTGAAGTGGAAGCTTGTGTAATGGATAAGTGTGCTGGCATATTTAACGTTGTGAGTAATTCATTAGGGTTAGTCATATTGTTAGTCCTAGCATTATTCAATTAA------------------------------------------------------------------------------------------------------------------------------------------------------------------------------------------------------------------------------------------------------------------------

>GU002527 Human Sarawak

ATGAAGAACTTCATTCTCTTGGCCGTCTCCTCCATCCTGCTGGTGGACTTGCTCCCCACACACTTCGAACATAATGTAGATCTCTCCAGGGCCATAAATGTAAATGGAGTAAGCTTCAATAATGTAGACACCAGTTCACTTGGCGCAGCACAGGTAAGACAAAGTGCTAGCCGAGGCAGAGGACTTGGTGAGAAGCCAAAAGAAGGAGATGATAAAGAAAAGAAAAAAGAAAAAGAAAAAGAAGAAGAACCAAAGAACCTAAATGAAAATAAGCCGAAACAACCGAATGCAGAAGGTGATGGAGCTAAGCCGAAACAACCGAATGCAGAAGGTGATGGAGCTAAGCTGAAACAACCGAATGCAGAAGGTGATGGAGCTAAGCTGAAACAACCGAATGCAGAAGGTGATGGAGGAAATGCACGACAACCGAATGCAGAAGGTGATGGAGGAAATGCACGACAACCGAATGCAGAAGGTGATGGAGGAAATGCACGACAACCGAATGCAGAAGGTGATGGAGGAAATGCACGACAACCGAATGCAGAAGGTGATGGAGGAAATGCACGACAACCGAATGCAGAAGGTGATGGAGGAAATGCACGACAACCTAATGCAGAAGGTGATGGAGGAAATGCACGACAACCGAATGCAGAAGGTGATGGAGGAAATGCACGACAACCGAATGCAGAAGGTGATGGAGGAAATGCACGACAACCTAATGCAGAAGGTGATGGAGGAAATGCACGACAACCTAATGCAGAAGGTGATGGAGGAAATGCACGACAACCTAATGCAGAAGGTGATGGAGGAAATGCACGACAACCGAATGCAGAAGGTGATGGAGCAAATGCACGACAGCCACAGGCAGAAGGTGGTGGAGGAAATGCACGACAAGGAGGAAATGAGGGGAATAAACAAGCAGGAAAAGGACAGGGACAAAACAATCAGGGTGCGAATGCCCCAAATGAAAAAGTTGTAAATGATTACCTACAGAAAATTAGATCTAGCGTTACCACCGAGTGGACTCCATGCAGTGTAACCTGTGGAAATGGTGTAAGAATTAGAAGAAGAGCTCATGCAGATAAGAAAAAGGCAGAGGACCTTACTATGGATGACCTTGAAGTGGAAGCTTGTGTAATGGATAAGTGTGCTGGCATATTTAACGTTGTGAGTAATTCATTAGGGTTAGTCATATTGTTAGTCCTAGCATTATTCAATTAA------------------------------------------------------------------------------------------------------------------------------------------------------------------------------------------------------------------------------------------------------------------------

>GU002526 Human Sarawak

ATGAAGAACTTCATTCTCTTGGCCGTCTCCTCCATCCTGCTGGTGGACTTGCTCCCCACACACTTCGAACATAATGTAGATCTCTCCAGGGCCATAAATGTAAATGGAGTAAGCTTCAATAATGTAGACACCAGTTCACTTGGCGCAGCACAGGTAAGACAAAGTGCTAGCCGAGGCAGAGGACTTGGTGAGAAGCCAAAAGAAGGAGATGATAAAGAAAAGAAAAAAGAAAAAGAAAAAGAAGAAGAACCAAAGAACCTAAATGAAAATAAGCCGAAACAACCGAATGCAGAAGGTGATGGAGCTAAGCCGAAACAACCGAATGCAGAAGGTGATGGAGCTAAGCTGAAACAACCGAATGCAGAAGGTGATGGAGCTAAGCTGAAACAACCGAATGCAGAAGGTGATGGAGGAAATGCACGACAACCGAATGCAGAAGGTGATGGAGGAAATGCACGACAACCGAATGCAGAAGGTGATGGAGGAAATGCACGACAACCGAATGCAGAAGGTGATGGAGGAAATGCACGACAACCGAATGCAGAAGGTGATGGAGGAAATGCACGACAACCGAATGCAGAAGGTGATGGAGGAAATGCACGACAACCTAATGCAGAAGGTGATGGAGGAAATGCACGACAACCGAATGCAGAAGGTGATGGAGGAAATGCACGACAACCGAATGCAGAAGGTGATGGAGGAAATGCACGACAACCTAATGCAGAAGGTGATGGAGGAAATGCACGACAACCTAATGCAGAAGGTGATGGAGGAAATGCACGACAACCTAATGCAGAAGGTGATGGAGGAAATGCACGACAACCGAATGCAGAAGGTGATGGAGCAAATGCACGACAGCCACAGGCAGAAGGTGGTGGAGGAAATGCACGACAAGGAGGAAATGAGGGGAATAAACAAGCAGGAAAAGGACAGGGACAAAACAATCAGGGTGCGAATGCCCCAAATGAAAAAGTTGTAAATGATTACCTACAGAAAATTAGATCTAGCGTTACCACCGAGTGGACTCCATGCAGTGTAACCTGTGGAAATGGTGTAAGAATTAGAAGAAGAGCTCATGCAGATAAGAAAAAGGCAGAGGACCTTACTATGGATGACCTTGAAGTGGAAGCTTGTGTAATGGATAAGTGTGCTGGCATATTTAACGTTGTGAGTAATTCATTAGGGTTAGTCATATTGTTAGTCCTAGCATTATTCAATTAA------------------------------------------------------------------------------------------------------------------------------------------------------------------------------------------------------------------------------------------------------------------------

>GU002525 Human Sarawak

ATGAAGAACTTCATTCTCTTGGCCGTCTCCTCCATCCTGCTGGTGGACTTGCTCCCCACACACTTCGAACATAATGTAGATCTCTCCAGGGCCATAAATGTAAATGGAGTAAGCTTCAATAATGTAGACACCAGTTCACTTGGCGCAGCACAGGTAAGACAAAGTGCTAGCCGAGGCAGAGGACTTGGTGAGAAGCCAAAAGAAGGAGATGATAAAGAAAAGAAAAAAGAAAAAGAAAAAGAAGAAGAACCAAAGAACCTAAATGAAAATAAGCCGAAACAACCGAATGCAGAAGGTGATGGAGCTAAGCCGAAACAACCGAATGCAGAAGGTGATGGAGCTAAGCTGAAACAACCGAATGCAGAAGGTGATGGAGCTAAGCTGAAACAACCGAATGCAGAAGGTGATGGAGGAAATGCACGACAACCGAATGCAGAAGGTGATGGAGGAAATGCACGACAACCGAATGCAGAAGGTGATGGAGGAAATGCACGACAACCGAATGCAGAAGGTGATGGAGGAAATGCACGACAACCGAATGCAGAAGGTGATGGAGGAAATGCACGACAACCGAATGCAGAAGGTGATGGAGGAAATGCACGACAACCTAATGCAGAAGGTGATGGAGGAAATGCACGACAACCGAATGCAGAAGGTGATGGAGGAAATGCACGACAACCGAATGCAGAAGGTGATGGAGGAAATGCACGACAACCTAATGCAGAAGGTGATGGAGGAAATGCACGACAACCTAATGCAGAAGGTGATGGAGGAAATGCACGACAACCTAATGCAGAAGGTGATGGAGGAAATGCACGACAACCGAATGCAGAAGGTGATGGAGCAAATGCACGACAGCCACAGGCAGAAGGTGGTGGAGGAAATGCACGACAAGGAGGAAATGAGGGGAATAAACAAGCAGGAAAAGGACAGGGACAAAACAATCAGGGTGCGAATGCCCCAAATGAAAAAGTTGTAAATGATTACCTACAGAAAATTAGATCTAGCGTTACCACCGAGTGGACTCCATGCAGTGTAACCTGTGGAAATGGTGTAAGAATTAGAAGAAGAGCTCATGCAGATAAGAAAAAGGCAGAGGACCTTACTATGGATGACCTTGAAGTGGAAGCTTGTGTAATGGATAAGTGTGCTGGCATATTTAACGTTGTGAGTAATTCATTAGGGTTAGTCATATTGTTAGTCCTAGCATTATTCAATTAA------------------------------------------------------------------------------------------------------------------------------------------------------------------------------------------------------------------------------------------------------------------------

>GU002524 Human Sarawak

ATGAAGAACTTCATTCTCTTGGCCGTCTCCTCCATCCTGCTGGTGGACTTGCTCCCCACACACTTCGAACATAATGTAGATCTCTCCAGGGCCATAAATGTAAATGGAGTAAGCTTCAATAATGTAGACACCAGTTCACTTGGCGCAGCACAGGTAAGACAAAGTGCTAGCCGAGGCAGAGGACTTGGTGAGAAGCCAAAAGAAGGAGATGATAAAGAAAAGAAAAAAGAAAAAGAAAAAGAAGAAGAACCAAAGAACCTAAATGAAAATAAGCCGAAACAACCGAATGCAGAAGGTGATGGAGCTAAGCCGAAACAACCGAATGCAGAAGGTGATGGAGCTAAGCTGAAACAACCGAATGCAGAAGGTGATGGAGCTAAGCTGAAACAACCGAATGCAGAAGGTGATGGAGGAAATGCACGACAACCGAATGCAGAAGGTGATGGAGGAAATGCACGACAACCGAATGCAGAAGGTGATGGAGGAAATGCACGACAACCGAATGCAGAAGGTGATGGAGGAAATGCACGACAACCGAATGCAGAAGGTGATGGAGGAAATGCACGACAACCGAATGCAGAAGGTGATGGAGGAAATGCACGACAACCTAATGCAGAAGGTGATGGAGGAAATGCACGACAACCGAATGCAGAAGGTGATGGAGGAAATGCACGACAACCGAATGCAGAAGGTGATGGAGGAAATGCACGACAACCTAATGCAGAAGGTGATGGAGGAAATGCACGACAACCTAATGCAGAAGGTGATGGAGGAAATGCACGACAACCTAATGCAGAAGGTGATGGAGGAAATGCACGACAACCGAATGCAGAAGGTGATGGAGCAAATGCACGACAGCCACAGGCAGAAGGTGGTGGAGGAAATGCACGACAAGGAGGAAATGAGGGGAATAAACAAGCAGGAAAAGGACAGGGACAAAACAATCAGGGTGCGAATGCCCCAAATGAAAAAGTTGTAAATGATTACCTACAGAAAATTAGATCTAGCGTTACCACCGAGTGGACTCCATGCAGTGTAACCTGTGGAAATGGTGTAAGAATTAGAAGAAGAGCTCATGCAGATAAGAAAAAGGCAGAGGACCTTACTATGGATGACCTTGAAGTGGAAGCTTGTGTAATGGATAAGTGTGCTGGCATATTTAACGTTGTGAGTAATTCATTAGGGTTAGTCATATTGTTAGTCCTAGCATTATTCAATTAA------------------------------------------------------------------------------------------------------------------------------------------------------------------------------------------------------------------------------------------------------------------------

>GU002491 Human Sarawak

ATGAAGAACTTCATTCTCTTGGCCGTCTCCTCCATCCTGCTGGTGGACTTGCTCCCCACACACTTCGAACATAATGTAGATCTCTCCAGGGCCATAAATGTAAATGGAGTAAGCTTCAATAATGTAGACACCAGTTCACTTGGCGCAGCACAGGTAAGACAAAGTGCTAGCCGAGGCAGAGGACTTGGTGAGAAGCCAAAAGAAGGAGCTGATAAAGAAAAGAAAAAAGAAAAAGAAAAAGAAAAAGAAGAAGAACCAAAGAAGCCAAATGAAAATAAGCTGAAACAACCGGAACAACCAGCAGCAGGAGCAGGGGGCGAACAACCAGCAGCAGGAGCAGGAGGCGAACAACCAGCAGCAGGAGCAGGAGGCGAACAACCAGCAGCAGGAGCAGGAGGCGAACAACCAGCAGCAGGAGCAGGAGGCGAACAACCAGCAGCAGGAGCAGGAGGCGAACAACCAGCAGCAGGAGCAAGAGGCGAACAACCAGCAGCAGGAGCAGGAGGCGAACAACCAGCAGCAGGAGCAGGAGGCGAACAACCAGCAGCAGGAGCAAGAGGCGAACAACCAGCAGCAGGAGCAGGAGGCGAACAACCAGCAGCAGGAGCAGGAGGCGAACAACCAGCACCAGCACCAAGGAGGGAACAACCAGCAGCAGGAGCAGGGGGCGAACAACCAGCACCAGCACCAAGGAGGGAACAACCAGCACCAGGAGCAGGTGCGGGAGATGGAGCACGAGGAGGAAACGCAGGGGCAGGTAAAGGACAGGGACAAAACAATCAGGGTGCGAATGTCCCAAATGAAAAAGTTGTGAATGATTACCTACACAAAATTAGATCTAGCGTTACCACCGAGTGGACTCCATGCAGTGTAACCTGTGGAAATGGTGTAAGAATTAGAAGAAAAGGTCATGCAGGTAATAAAAAGGCAGAGGACCTTACTATGGATGACCTTGAGGTGGAAGCTTGTGTAATGGATAAGTGCGCTGGCATATTTAACGTTGTGAGTAATTCATTAGGCTTAGTCATATTGTTAGTCCTAGCATTATTCAATTAA---------------------------------------------------------------------------------------------------------------------------------------------------------------------------------------------------------------------------------------------------------------------------------------------------------------------------------------------------------------------------------------------------------------------------------------------

>GU002490 Human Sarawak

ATGAAGAACTTCATTCTCTTGGCCGTCTCCTCCATCCTGCTGGTGGACTTGCTCCCCACACACTTCGAACATAATGTAGATCTCTCCAGGGCCATAAATGTAAATGGAGTAAGCTTCAATAATGTAGACACCAGTTCACTTGGCGCAGCACAGGTAAGACAAAGTGCTAGCCGAGGCAGAGGACTTGGTGAGAAGCCAAAAGAAGGAGATGATAAAGAAAAGAAAAAAGAAAAAGAAAAAGAAGAAGAACCAAAGAACCTAAATGAAAATAAGCCGAAACAACCGAATGCAGAAGGTGATGGAGCTAAGCCGAAACAACCGAATGCAGAAGGTGATGGAGCTAAGCTGAAACAACCGAATGCAGAAGGTGATGGAGCTAAGCTGAAACAACCGAATGCAGAAGGTGATGGAGGAAATGCACGACAACCGAATGCAGAAGGTGATGGAGGAAATGCACGACAACCGAATGCAGAAGGTGATGGAGGAAATGCACGACAACCGAATGCAGAAGGTGATGGAGGAAATGCACGACAACCGAATGCAGAAGGTGATGGAGGAAATGCACGACAACCGAATGCAGAAGGTGATGGAGGAAATGCACGACAACCTAATGCAGAAGGTGATGGAGGAAATGCACGACAACCGAATGCAGAAGGTGATGGAGGAAATGCACGACAACCGAATGCAGAAGGTGATGGAGGAAATGCACGACAACCTAATGCAGAAGGTGATGGAGGAAATGCACGACAACCTAATGCAGAAGGTGATGGAGGAAATGCACGACAACCTAATGCAGAAGGTGATGGAGGAAATGCACGACAACCGAATGCAGAAGGTGATGGAGCAAATGCACGACAGCCACAGGCAGAAGGTGGTGGAGGAAATGCACGACAAGGAGGAAATGAGGGGAATAAACAAGCAGGAAAAGGACAGGGACAAAACAATCAGGGTGCGAATGCCCCAAATGAAAAAGTTGTAAATGATTACCTACAGAAAATTAGATCTAGCGTTACCACCGAGTGGACTCCATGCAGTGTAACCTGTGGAAATGGTGTAAGAATTAGAAGAAGAGCTCATGCAGATAAGAAAAAGGCAGAGGACCTTACTATGGATGACCTTGAAGTGGAAGCTTGTGTAATGGATAAGTGTGCTGGCATATTTAACGTTGTGAGTAATTCATTAGGGTTAGTCATATTGTTAGTCCTAGCATTATTCAATTAA------------------------------------------------------------------------------------------------------------------------------------------------------------------------------------------------------------------------------------------------------------------------

>GU002489 Human Sarawak

ATGAAGAACTTCATTCTCTTGGCCGTCTCCTCCATCCTGCTGGTGGACTTGCTCCCCACACACTTCGAACATAATGTAGATCTCTCCAGGGCCATAAATGTAAATGGAGTAAGCTTCAATAATGTAGACACCAGTTCACTTGGCGCAGCACAGGTAAGACAAAGTGCTAGCCGAGGCAGAGGACTTGGTGAGAAGCCAAAAGAAGGAGATGATAAAGAAAAGAAAAAAGAAAAAGAAAAAGAAGAAGAACCAAAGAACCTAAATGAAAATAAGCCGAAACAACCGAATGCAGAAGGTGATGGAGCTAAGCTGAAACAACCGAATGCAGAAGGTGATGGAGCTAAGCTGAAACAACCGAATGCAGAAGGTGATGGAGGAAATGCACGACAACCGAATGCAGAAGGTGATGGAGGAAATGCACGACAACCGAATGCAGAAGGTGATGGAGGAAATGCACGACAACCGAATGCAGAAGGTGATGGAGGAAATGCACGACAACCGAATGCAGAAGGTGATGGAGGAAATGCACGACAACCGAATGCAGAAGGTGATGGAGGAAATGCACGACAACCTAATGCAGAAGGTGATGGAGGAAATGCACGACAACCGAATGCAGAAGGTGATGGAGGAAATGCACGACAACCGAATGCAGAAGGTGATGGAGGAAATGCACGACAACCTAATGCAGAAGGTGATGGAGGAAATGCACGACAACCTAATGCAGAAGGTGATGGAGGAAATGCACGACAACCTAATGCAGAAGGTGATGGAGGAAATGCACGACAACCGAATGCAGAAGGTGATGGAGCAAATGCACGACAGCCACAGGCAGAAGGTGGTGGAGGAAATGCACGACAAGGAGGAAATGAGGGGAATAAACAAGCAGGAAAAGGACAGGGACAAAACAATCAGGGTGCGAATGCCCCAAATGAAAAAGTTGTAAATGATTACCTACAGAAAATTAGATCTAGCGTTACCACCGAGTGGACTCCATGCAGTGTAACCTGTGGAAATGGTGTAAGAATTAGAAGAAGAGCTCATGCAGATAAGAAAAAGGCAGAGGACCTTACTATGGATGACCTTGAAGTGGAAGCTTGTGTAATGGATAAGTGTGCTGGCATATTTAACGTTGTGAGTAATTCATTAGGGTTAGTCATATTGTTAGTCCTAGCATTATTCAATTAA------------------------------------------------------------------------------------------------------------------------------------------------------------------------------------------------------------------------------------------------------------------------------------------------------------

>GU002488 Human Sarawak

ATGAAGAACTTCATTCTCTTGGCCGTCTCCTCCATCCTGCTGGTGGACTTGCTCCCCACACACTTCGAACATAATGTAGATCTCTCCAGGGCCATAAATGTAAATGGAGTAAGCTTCAATAATGTAGACACCAGTTCACTTGGCGCAGCACAGGTAAGACAAAGTGCTAGCCGAGGCAGAGGACTTGGTGAGAAGCCAAAAGAAGGAGATGATAAAGAAAAGAAAAAAGAAAAAGAAAAAGAAGAAGAACCAAAGAACCTAAATGAAAATAAGCCGAAACAACCGAATGCAGAAGGTGATGGAGCTAAGCCGAAACAACCGAATGCAGAAGGTGATGGAGCTAAGCTGAAACAACCGAATGCAGAAGGTGATGGAGCTAAGCTGAAACAACCGAATGCAGAAGGTGATGGAGGAAATGCACGACAACCGAATGCAGAAGGTGATGGAGGAAATGCACGACAACCGAATGCAGAAGGTGATGGAGGAAATGCACGACAACCGAATGCAGAAGGTGATGGAGGAAATGCACGACAACCGAATGCAGAAGGTGATGGAGGAAATGCACGACAACCGAATGCAGAAGGTGATGGAGGAAATGCACGACAACCTAATGCAGAAGGTGATGGAGGAAATGCACGACAACCGAATGCAGAAGGTGATGGAGGAAATGCACGACAACCGAATGCAGAAGGTGATGGAGGAAATGCACGACAACCTAATGCAGAAGGTGATGGAGGAAATGCACGACAACCTAATGCAGAAGGTGATGGAGGAAATGCACGACAACCTAATGCAGAAGGTGATGGAGCAAATGCACGACAACCGAATGCAGAAGGTGATGGAGCAAATGCACGACAGCCACAGGCAGAAGGTGGTGGAGGAAATGCACGACAAGGAGGAAATGAGGGGAATAAACAAGCAGGAAAAGGACAGGGACAAAACAATCAGGGTGCGAATGCCCCAAATGAAAAAGTTGTAAATGATTACCTACAGAAAATTAGATCTAGCGTTACCACCGAGTGGACTCCATGCAGTGTAACCTGTGGAAATGGTGTAAGAATTAGAAGAAGAGCTCATGCAGATAAGAAAAAGGCAGAGGACCTTACTATGGATGACCTTGAAGTGGAAGCTTGTGTAATGGATAAGTGTGCTGGCATATTTAACGTTGTGAGTAATTCATTAGGGTTAGTCATATTGTTAGTCCTAGCATTATTCAATTAA------------------------------------------------------------------------------------------------------------------------------------------------------------------------------------------------------------------------------------------------------------------------

>GU002487 Human Sarawak

ATGAAGAACTTCATTCTCTTGGCCGTCTCCTCCATCCTGCTGGTGGACTTGCTCCCCACACACTTCGAACATAATGTAGATCTCTCCAGGGCCATAAATGTAAATGGAGTAAGCTTCAATAATGTAGACACCAGTTCACTTGGCGCAGCACAGGTAAGACAAAGTGCTAGCCGAGGCAGAGGACTTGGTGAGAAGCCAAAAGAAGGAGATGATAAAGAAAAGAAAAAAGAAAAAGAAAAAGAAGAAGAACCAAAGAACCTAAATGAAAATAAGCCGAAACAACCGAATGCAGAAGGTGATGGAGCTAAGCCGAAACAACCGAATGCAGAAGGTGATGGAGCTAAGCTGAAACAACCGAATGCAGAAGGTGATGGAGCTAAGCTGAAACAACCGAATGCAGAAGGTGATGGAGGAAATGCACGACAACCGAATGCAGAAGGTGATGGAGGAAATGCACGACAACCGAATGCAGAAGGTGATGGAGGAAATGCACGACAACCGAATGCAGAAGGTGATGGAGGAAATGCACGACAACCGAATGCAGAAGGTGATGGAGGAAATGCACGACAACCTAATGCAGAAGGTGATGGAGGAAATGCACGACAACCTAATGCAGAAGGTGATGGAGGAAATGCACGACAACCGAATGCAGAAGGTGATGGAGGAAATGCACGACAACCGAATGCAGAAGGTGATGGAGGAAATGCACGACAACCTAATGCAGAAGGTGATGGAGGAAATGCACGACAACCTAATGCAGAAGGTGATGGAGGAAATGCACGACAACCTAATGCAGAAGGTGATGGAGGAAATGCACGACAACCGAATGCAGAAGGTGATGGAGCAAATGCACGACAGCCACAGGCAGAAGGTGGTGGAGGAAATGCACGACAAGGAGGAAATGAGGGGAATAAACAAGCAGGAAAAGGACAGGGACAAAACAATCAGGGTGCGAATGCCCCAAATGAAAAAGTTGTAAATGATTACCTACAGAAAATTAGATCTAGCGTTACCACCGAGTGGACTCCATGCAGTGTAACCTGTGGAAATGGTGTAAGAATTAGAAGAAGAGCTCATGCAGATAAGAAAAAGGCAGAGGACCTTACTATGGATGACCTTGAAGTGGAAGCTTGTGTAATGGATAAGTGTGCTGGCATATTTAACGTTGTGAGTAATTCATTAGGGTTAGTCATATTGTTAGTCCTAGCATTATTCAATTAA------------------------------------------------------------------------------------------------------------------------------------------------------------------------------------------------------------------------------------------------------------------------

>GU002486 Human Sarawak

ATGAGGAACTTCATTCTCTTGGCCGTCTCCTCCATCCTGCTGGTGGACTTGCTCCCCACACACTTCGAACATAATGTAGATCTCTCCAGGGCCATAAATGTAAATGGAGTAAGCTTCAATAATGTAGACACCAGTTCACTTGGCGCAGCACAGGTAAGACAAAGTGCTAGCCGAGGCAGAGGACTTGGTGAGAAGCGAAAAGAAGGAGCTGATAAAGAAAAGAAAAAAGAAAAAGAAGAAGAACCAAAGAAGCCAAATGAAAATAAGCTGAAACAACCGAATCCAGACCAACCACAAGCACAGGGTGATGGAGCAAATGCAGGACAACCACAAGCACAAGGAGATGGAGCAAATGCAGGACAACCACAAGCACAAGGAGATGGAGCAAATGCAGGACAACCACAAGCACAGGGTGATGGAGCAAATGCAGGACAACCACAAGCACAAGGAGATGGAGCAAATGCAGGACAACCACAAGCACAAGGAGATGGAGCAAATGCAGGACAACCACAAGCACAAGGAGATGGAGCAAATGCAGGACAACCACAAGCACAGGGTGATGGAGCAAATGCAGGACAACCACAAGCACAAGGAGATGGAGCAAATGCAGGACAACCACAAGCACAGGGTGATGGAGCAAATGCAGGACAACCACAAGCACAGGGTGATAGGGCGAATGCAGGACAACCACAAGCACAAGGAGATGGGGCAAATGTACCACGACAAGGAAGAAACGGGGGAGGTGCACCAGCAGGAGGAAATGAGGGGAATAAACAAGCAGGAAAAGGACAGGGACAAAACAATCAGGGTGCGAATGCCCCAAATGAAAAAGTTGTGAATGATTACCTACACAAAATTAGATCTAGCGTTACCACCGAGTGGACTCCATGCAGTGTAACCTGTGGAAATGGTGTAAGAATTAGAAGAAGACAGAATGCTGGTAATAAAAAGGCAGAGGACCTTACTATGGATGACCTTGAGGTGGAAGCTTGTGTAATGGATAAGTGCGCTGGCATATTTAACGTTGTGAGTAATTCATTAGGGTTAGTCATATTGTTAGTCCTAGCATTATTCAATTAA---------------------------------------------------------------------------------------------------------------------------------------------------------------------------------------------------------------------------------------------------------------------------------------------------------------------------------------------------------------------------------------------------------------------

>GU002485 Human Sarawak

ATGAAGAACTTCATTCTCTTGGCCGTCTCCTCCATCCTGCTGGTGGACTTGTTCCCCACACACTTCGAACATAATGTAGATCTCTCCAGGGCCATAAATGTAAATGGAGTAAGCTTCAATAATGTAGACACCAGTTCACTTGGCGCAGCACAGGTAAGACAAAGTGCTAGCCGAGGCAGAGGACTTGGTGAGAAGCCAAAAGAAGGAGCTGATAAAGAAAAGAAAAAAGAAAAAGAAGAACCAAAGAAGCCAAATGAAAATAAGCTGAAACAACCACAAGCAGAAGGTGATGGAGCAAATGCACGACAACCACAGGCAGAAGGTGATGGAGCAAATGCACGACAACCACAAGCAGAAGGTGATGGAGCAAATGCACGACAACCACAGGCAGAAGGTGATGGAGCAAATGCACGACAACCACAGGCAGAAGGTGATGGAGCAAATGCACGACAACCACAAGCAGAAGGTGATGGAGCAAATGCACGACAACCACAGGCAGAAGGTGATGGAGCAAATGCACGACAACCACAGGCAGAAGGTGATGGAGCAAATGCACGACAACCACAAGCAGAAGGTGATGGAGCAAATGCACGACAGCCACAGGCAGAAGGTGATGGAGCAAATGCACGACAGCCACAGGCAGAAGGTGGTGGAGCAAATGCACGACAGCCACAGGCAGAAGGTGATGGAGCAAATGCACGACAACCACAAGCACAAGGAGATGGAGGAAATGCACGACAAGGAGGAAACGGGGGAGGTGCACCAGCAGGAGGAAATGAGGGGAATAAACAAGCAGGAAAAGGACAGGGACAAAACAATCAGGGTGCGAATGCCCCAAATGAAAAAGTTGTAAATGATTACCTACAGAAAATTAGATCTAGCGTTACCACCGAGTGGACTCCATGCAGTGTAACCTGTGGAAATGGTGTAAGAATTAGAAGAAGAGCTCATGCAGATAAGAAAAAGGCAGAGGACCTTACTATGGATGACCTTGAAGTGGAAGCTTGTGTAATGGATAAGTGTGCTGGCATATTTAACGTTGTGAGTAATTCATTAGGGTTAGTCATATTGTTAGTCCTAGCATTATTCAATTAA------------------------------------------------------------------------------------------------------------------------------------------------------------------------------------------------------------------------------------------------------------------------------------------------------------------------------------------------------------------------------------------------------

>GU002484 Human Sarawak

ATGAAGAACTTCATTCTCTTGGCCGTCTCCTCCATCCTGCTGGTGGACTTGCTCCCCACACACTTCGAACATAATGTAGATCTCTCCAGGGCCATAAATATAAATGGAGTAAGCTTCAATAATGTAGACACCAGTTCACTTGGCGCAGCACAGGTAAGACAAAGTGCTAGCCGAGGCAGAGGACTTGGTGAAAAGCCAAAAGAAGGAGCTGATAAAGAAAAGAAAAAAGAAAAAGAAAAAGAAGAAGAACCAAAGAAGCCAAATGAAAATAAGCTGAAACAACCTAATGCAGAAGGTGATGGAGCAAATGCACGACAACCGAATGCAGAAGGTGATGGAGCAAATGCACGACAACCTAATGCAGAAGGTGATGGAGCAAATGCACGACAACCGAATGCAGAAGGTGATGGAGCAAATGCACGACAACCTAATGCAGAAGGTGATGGAGCAAATGCACGACAACCTAATGCAGAAGGTGATGGAGCAAATGCACGACAACCTAATGCAGAAGGTGATGGAGCAAATGCACGACAACCGAATGCAGAAGGTGATGGAGCAAATGCACGACAACCGAATGCAGAAGGTGATGGAGCAAATGCACGACAACCTAATGCAGAAGGTGATGGAGCAAATGCACGACAACCGAATGCAGAAGGTGGTGGAGCAAATGCACGACAACCGAATGCAGAAGGTGATGGAGCAAATGCACGACAACCACAAGCACAAGGAGATGGAGGAAATGCACGACAAGGAGGAAACGGGGGAGGTGCACCAGCAGGAGGAAATGAGGGGAATAAACAAGCAGGAAAAGGACAGGGACAAAACAATCAGGGTGCGAATGCCCCAAATGAAAAAGTTGTAAATGATTACCTACAGAAAATTAGATCTAGCGTTACCACCGAGTGGACTCCATGCAGTGTAACCTGTGGAAATGGTGTAAGAATTAGAAGAAGAGCTCATGCAGATAAGAAAAAGGCAGAGGACCTTACTATGGATGACCTTGAAGTGGAAGCTTGTGTAATGGATAAGTGTGCTGGCATATTTAACGTTGTGAGTAATTCATTAGGGTTAGTCATATTGTTAGTCCTAGCATTATTCAATTAA---------------------------------------------------------------------------------------------------------------------------------------------------------------------------------------------------------------------------------------------------------------------------------------------------------------------------------------------------------------------------------------------

>GU002483 Human Sarawak

ATGAAGAACTTCATTCTCTTGGCCGTCTCCTCCATCCTGCTGGTGGACTTGTTCCCCACACACTTCGAACATAATGTAGATCTCTCCAGGGCCATAAATGTAAATGGAGTAAGCTTCAATAATGTAGACACCAGTTCACTTGGCGCAGCACAGGTAAGACAAAGTGCTAGCCGAGGCAGAGGACTTGGTGAGAAGCCAAAAGAAGGAGCTGATAAAGAAAAGAAAAAAGAAAAAGAAAAAGAAAAAGAAGAAGAACCAAAGAAGCCAAATGAAAATAAGCTGAAACAACCGGAAGGAAATCAAGATGGGCGAGCACAACCGGAAGGAAATCAGGATGGTCGAGCGCAACCGGAAGGAAATCAGGATGGTCGAGCGCAACCGGAAGGAAATCAAGATGGGCGAGCACAACCGGAAGGAAATCAGGATGGTCGAGCGCAACCGGAAGGAAATCAAGATGGGCGAGCACAACCGGAAGGAAATCAGGATGGTCGAGCGCAACCGGAAGGAAATCAGGATGGGCGAGCGCAACCGGAAGGAAATCAGGATGGACGAGCACAACCGGAAGGAAATAGGGAAGCTCCAGCACAACCACAAGGAAATGGGGGGGCAGGTCAAGCACAACCGGAAGGAAATAGGGAAGCTCCAGCACAACCACAAGGAAATGGGGGGGCAGGTCAAGCACAACCGGAAGGAAATAGGGAAGCTCCAGCACAACCACAAGGAAATGGGGGGGCAGGTCAAGCACAACCACAAAAAAACGAAGGAGGAAACGCAGGAGCACGGAAAGGACAGGGACAAAACAATCAGGGTGCGAATGCCCCAAATGAAAAAGTTGTGAATGATTACCTACACAAAATTAGATCTAGCGTTACCACCGAGTGGACTCCATGCAGTGTAACCTGTGGAAATGGTGTAAGAATTAGAAGAAAAGCTCATGCAGATAAGAAAAAGGCAGAGGACCTTACTATGGATGACCTTGAAGTGGAAGCTTGTGTAATGGATAAGTGCGCTGGCATATTTAACGTTGTGAGTAATTCATTAGGGTTAGTCATATTGTTAGTCCTAGCATTATTCAATTAA---------------------------------------------------------------------------------------------------------------------------------------------------------------------------------------------------------------------------------------------------------------------------------------------------------------------------------------------------------------------------------------------------------------------

>GU002482 Human Sarawak

ATGAGGAACTTCATTCTCTTGGCCGTCTCCTCCATCCTGCTGGTGGACTTGCTCCCCACACACTTCGAACATAATGTAGATCTCTCCAGGGCCATAAATGTAAATGGAGTAAGCTTCAATAATGTAGACACCAGTTCACTTGGCGCAGCACAGGTAAGACAAAGTGCTAGCCGAGGCAGAGGACTTGGTGAGAAGCGAAAAGAAGGAGCTGATAAAGAAAAGAAAAAAGAAAAAGAAGAAGAACCAAAGAAGCCAAATGAAAATAAGCTGAAACAACCGGAACAAGCAGCACCAGGAGCAGGGGGAGAACAACCAGCACCAGGAGCAGGGGGAGAACGACCAGCACCAGGAGCAGGGGGAGAACAACCAGCACCAGGAGCAGGGGGAGAACAACCAGCACCAGGAGCAGGGGGAGAACAACCAGCACCAGGAGCAGGGGGAGAACAACCAGCACCAGGAGCAGGGGGAGAACGACCAGCACCAGGAGCAGGGGGAGAACAACCAGCACCAGGAGCAGGGGGAGAACGACCAGCACCAGGAGCAGGGGGAGAACAACCAGCACCAGGAGCAGGGGGAGAACGACCAGCACCAGGAGCAGGGGGAGAACAACCAGCACCAGGAGCAGGGGGAGAACAACCAGCACCAGGAGCAGGGGGAGAACAACCAGCACCAGCACCAAGGAGGGAACAACCAGCACCAGCACCAGGTGCGGGAGATGGAGCACGAGGAGGAAACGCAGGGGCAGGTAAAGGACAGGGACAAAACAATCAGGGTGCGAATGTCCCAAATGAAAAAGTTGTGAATGATTACCTACACAAAATTAGATCTAGCGTTACCACCGAGTGGACTCCATGCAGTGTAACCTGTGGAAATGGTGTAAGAATTAGAAGAAAAGCTCATGCAGATAAGAAAAAGGCAGAGGACCTTACTATGGATGACCTTGAAGTGGAAGCTTGTGTAATGGATAAGTGCGCTGGCATATTTAACGTTGTGAGTAATTCATTAGGGTTAGTCATATTGTTAGTCCTAGCATTATTCAATTAA---------------------------------------------------------------------------------------------------------------------------------------------------------------------------------------------------------------------------------------------------------------------------------------------------------------------------------------------------------------------------------------------------------------------------------------------------------

>GU002480 Human Sarawak

ATGAGGAACTTCATTCTCTTGGCCGTCTCCTCCATCCTGCTGGTGGACTTGCTCCCCACACACTTCGAACATAATGTAGATCTCTCCAGGGCCATAAATGTAAATGGAGTAAGCTTCAATAATGTAGACACCAGTTCACTTGGCGCAGCACAGGTAAGACAAAGTGCTAGCCGAGGCAGAGGACTTGGTGAGAAGCGAAAAGAAGGAGCTGATAAAGAAAAGAAAAAAGAAAAAGAAGAAGAACCAAAGAAGCCAAATGAAAATAAGCTGAAACAACCGAATCCAGGACAACCACAAGCACAAGGAGATGGAGCAAATGCAGGACAACCACAAGCACAAGGAGATGGAGCAAATGCAGGACAACCACAAGCACAGGGTGATGGAGCAAATGCAGGACAACCACAAGCACAAGGAGATGGAGCAAATGCAGGACAACCACAAGCACAGGGTGATGGAGCAAATGCAGGACAACCACAAGCACAAGGAGATGGAGCAAATGCAGGACAACCACAAGCACAGGGTGATGGAGCAAATGCAGGACAACCACAAGCACAAGGAGATGGAGCAAATGCAGGACAACCACAAGCACAGGGTGATGGAGCAAATGCAGGACAACCACAAGCACAGGGTGATGGAGCAAATGCAGGACAACCACAAGCACAGGGTGATGGAGCAAATGCAGGACAACCACAAGCACAGGGTGATAGGGCGAATGCAGGACAACCACAAGCACAAGGAGATGGGGCAAATGTACCACGACAAGGAAGAAACGGGGGAGGTGCACCAGCAGGAGGAAATGAGGGGAATAAACAAGCAGGAAAAGGACAGGGACAAAACAATCAGGGTGCGAATGCCCCAAATGAAAAAGTTGTGAATGATTACCTACACAAAATTAGATCTAGCGTTACCACCGAGTGGACTCCATGCAGTGTAACCTGTGGAAATGGTGTAAGAATTAGAAGAAAAGCTCATGCAGGTAATAAAAAGGCAGAGGACCTTACTATGGATGACCTTGAGGTGGAAGCTTGTGTAATGGATAAGTGCGCTGGCATATTTAACGTTGTGAGTAATTCATTAGGCTTAGTCATATTGTTAGTCCTAGCATTATTCAATTAA---------------------------------------------------------------------------------------------------------------------------------------------------------------------------------------------------------------------------------------------------------------------------------------------------------------------------------------------------------------------------------

>GU002479 Human Sarawak

ATGAGGAACTTCATTCTCTTGGCCGTCTCCTCCATCCTGCTGGTGGACTTGCTCCCCACACACTTCGAACATAATGTAGATCTCTCCAGGGCCATAAATGTAAATGGAGTAAGCTTCAATAATGTAGACACCAGTTCACTTGGCGCAGCACAGGTAAGACAAAGTGCTAGCCGAGGCAGAGGACTTGGTGAGAAGCGAAAAGAAGGAGCTGATAAAGAAAAGAAAAAAGAAAAAGAAGAAGAACCAAAGAAGCCAAATGAAAATAAGCTGAAACAACCGAATCCAGACCAACCACAAGCACAGGGTGATGGAGCAAATGCAGGACAACCACAAGCACAAGGAGATGGAGCAAATGCAGGACAACCACAAGCACAGGGTGATGGAGCAAATGCAGGACAACCACAAGCACAGGGTGATGGAGCAAATGCAGGACAACCACAAGCACAAGGAGATGGAGCAAATGCAGGACAACCACAAGCACAGGGTGATGGAGCAAATGCAGGACAACCACAAGCACAAGGAGATGGAGCAAATGCAGGACAACCACAAGCACAAGGAGATGGAGCAAATGCAGGACAACCACAAGCACAGGGTGATGGAGCAAATGCAGGACAACCACAAGCACAGGGTGATGGAGCAAATGCAGGACAACCACAAGCACAGGGTGATAGGGCGAATGCAGGACAACCACAAGCACAAGGAGATGGGGCAAATGTACCACGACAAGGAAGAAACGGGGGAGGTGCACCAGCAGGAGGAAATGAGGGGAATAAACAAGCAGGAAAAGGACAGGGACAAAACAATCAGGGTGCGAATGCCCCAAATGAAAAAGTTGTGAATGATTACCTACACAAAATTAGATCTAGCGTTACCACCGAGTGGACTCCATGCAGTGTAACCTGTGGAAATGGTGTAAGAATTAGAAGAAAAGCTCATGCAGGTAATAAAAAGGCAGAGGACCTTACTATGGATGACCTTGAGGTGGAAGCTTGTGTAATGGATAAGTGCGCTGGCATATTTAACGTTGTGAGTAATTCATTAGGGTTAGTCATATTGTTAGTCCTAGCATTATTCAATTAA---------------------------------------------------------------------------------------------------------------------------------------------------------------------------------------------------------------------------------------------------------------------------------------------------------------------------------------------------------------------------------------------------------------------

>GU002478 Human Sarawak

ATGAAGAACTTCATTCTCTTGGCCGTCTCCTCCATCCTGCTGGTGGACTTGTTCCCCACACACTTCGAACATAATGTAGATCTCTCCAGGGCCATAAATGTAAATGGAGTAAGCTTCAATAATGTAGACACCAGTTCACTTGGCGCAGCACAGGTGAGACAAAGTGCTAGCCGAGGCAGAGGACTTGGTGAGAAGCCAAAAGAAGGAGCTGATAAAGAAAAGAAAAAAGAAAAAGAAGAAAAACCAAAGAAGCCAAATGAAAATAAGCTGAAACAACCAGGGGGCGAACAACCAGCAGCAGGGGGCGAACAACCAGCAGCAGGGGGCGAACAACCAGCAGCAGGGGGTGAACAACCAGCAGCAGGGGGCGAACGACCAGCAGCAGGGGGCGAACAACCAGCAGCAGGAGGTGAACAACCAGCAGCAGGGGGCGAACAACCAGCAGCAGGGGGCGAACAACCAGCAGCAGGGGGCGAACGACCAGCAGCAGGGGGCGAACAACCAGCAGCAGGGGGTGAACAACCAGCAGCAGGGGGCGAACGACCAGCAGCAGGGGGCGAACAACCAGCAGCAGGGGGTGAACAACCAGCACCAGCACCAAGGAGGGAACAACCAGCAGCAGGGGGCGAACAACCAGCACCAGCACCAAGGAGGGAACAACCAGCACCAGGAGCAGGTGCGGGAGATGGAGCACGAGGAGGAAACCCAGGGGCAGGTAAAGGACAGGGACAAAACAATCAGGGTGCGAATGTCCCAAATGAAAAAGTTGTGAATGATTACCTACACAAAATTAGATCTAGCGTTACCACCGAGTGGACTCCATGCAGTGTAACCTGTGGAAATGGTGTAAGAATTAGAAGAAGACAGAATGCTGGTAATAAAAAGGCAGAGGACCTTACTATGGATGACCTTGAGGTGGAAGCTTGTGTAATGGATAAGTGCGCTGGCATATTTAACGTTGTGAGTAATTCATTAGGGTTAGTCATATTGTTAGTCCTAGCATTATTCAATTAA---------------------------------------------------------------------------------------------------------------------------------------------------------------------------------------------------------------------------------------------------------------------------------------------------------------------------------------------------------------------------------------------------------------------------------------------------------------------------------------

>GU002477 Human Sarawak

ATGAAGAACTTCATTCTCTTGGCCGTCTCCTCCATCCTGCTGGTGGACTTGTTCCCCACACACTTCGAACATAATGTAGATCTCTCCAGGGCCATAAATGTAAATGGAGTAAGCTTCAATAATGTAGACACCAGTTCACTTGGCGCAGCACAGGTGAGACAAAGTGCTAGCCGAGGCAGAGGACTTGGTGAGAAGCCAAAAGAAGGAGCTGATAAAGAAAAGAAAAAAGAAAAAGAAGAAAAACCAAAGAAGCCAAATGAAAATAAGCTGAAACAACCAGGGGGCGAACAACCAGCAGCAGGGGGCGAACAACCAGCAGCAGGGGGTGAACAACCAGCAGCAGGGGGTGAACAACCAGCAGCAGGGGGTGAACAACCAGCAGCAGGGGGCGAACGACCAGCAGCAGGGGGCGAACAACCAGCAGCAGGGGGTGAACAACCAGCAGCAGGGGGCGAACGACCAGCAGCAGGGGGTGAACAACCAGCAGCAGGGGGCGAACGACCAGCAGCAGGGGGCGAACAACCAGCAGCAGGGGGCGAACAACCAGCAGCAGGGGGTGAACAACCAGCACCAGCACCAAGGAGGGAACAACCAGCAGCAGGGGGCGAACAACCAGCACCAGCACCAAGGAGGGAACAACCAGCACCAGGAGCAGGTGCGGGAGATGGAGCACGAGGAGGAAACGCAGGGGCAGGTAAAGGACAGGGACAAAACAATCAGGGTGCGAATGTCCCAAATGAAAAAGTTGTGAATGATTACCTACACAAAATTAGATCTAGCGTTACCACCGAGTGGACTCCATGCAGTGTAACCTGTGGAAATGGTGTAAGAATTAGAAGAAGACAGAATGCTGGTAATAAAAAGGCAGAGGACCTTACTATGGATGACCTTGAGGTGGAAGCTTGTGTAATGGATAAGTGCGCTGGCATATTTAACGTTGTGAGTAATTCATTAGGGTTAGTCATATTGTTAGTCCTAGCATTATTCAATTAA------------------------------------------------------------------------------------------------------------------------------------------------------------------------------------------------------------------------------------------------------------------------------------------------------------------------------------------------------------------------------------------------------------------------------------------------------------------------------------------------------------

>GU002476 Human Sarawak

ATGAGGAACTTCATTCTCTTGGCCGTCTCCTCCATCCTGCTGGTGGACTTGCTCCCCACACACTTCGAACATAATGTAGATCTCTCCAGGGCCATAAATGTAAATGGAGTAAGCTTCAATAATGTAGACACCAGTTCACTTGGCGCAGCACAGGTGAGACAAAGTGCTAGCCGAGGCAGAGGACTTGGTGAGAAGCCAAAAGAAGGAGCTGATAAAGAAAAGAAAAAAGAAAAAGAAGAAAAACCAAAGAAGCCAAATGAAAATAAGCTGAAACAACCAGGGGGCGAACAACCAGCAGCAGGGGGCGAACAACCAGCAGCAGGGGGCGAACAACCAGCAGCAGGGGGTGAACAACCAGCAGCAGGGGGCGAACGACCAGCAGCAGGGGGCGAACAACCAGCAGCAGGGGGTGAACAACCAGCAGCAGGGGGCGAACAACCAGCAGCAGGGGGCGAACAACCAGCAGCAGGGGGCGAACGACCAGCAGCAGGGGGCGAACAACCAGCAGCAGGGGGTGAACAACCAGCAGCAGGGGGTGAACAACCAGCAGCAGGGGGCGAACGACCAGCAGCAGGGGGCGAACAACCAGCACCAGCACCAAGGAGGGAACAACCAGCAGCAGGGGGCGAACAACCAGCACCAGCACCAAGGAGGGAACAACCAGCACCAGGAGCAGGTGCGGGAGATGGAGCACGAGGAGGAAACGCAGGGGCAGGTAAAGGACAGGGACAAAACAATCAGGGTGCGAATGTCCCAAATGAAAAAGTTGTGAATGATTACCTACACAAAATTAGATCTAGCGTTACCACCGAGTGGACTCCATGCAGTGTAACCTGTGGAAATGGTGTAAGAATTAGAAGAAGACAGAATGCTGGTAATAAAAAGGCAGAGGACCTTACTATGGATGACCTTGAGGTGGAAGCTTGTGTAATGGATAAGTGCGCTGGCATATTTAACGTTGTGAGTAATTCATTAGGGTTAGTCATATTGTTAGTCCTAGCATTATTCAATTAA---------------------------------------------------------------------------------------------------------------------------------------------------------------------------------------------------------------------------------------------------------------------------------------------------------------------------------------------------------------------------------------------------------------------------------------------------------------------------------------

>GU002475 Human Sarawak

ATGAGGAACTTCATTCTCTTGGCCGTCTCCTCCATCCTGCTGGTGGACTTGCTCCCCACACACTTCGAACATAATGTAGATCTCTCCAGGGCCATAAATGTAAATGGAGTAAGCTTCAATAATGTAGACACCAGTTCACTTGGCGCAGCACAGGTAAGACAAAGTGCTAGTCGAGGCAGAGGACTTGGTGAGAAGCGAAAAGAAGGAGCTGATAAAGAAAAGAAAAAAGAAAAAGAAGAAGAACCAAAGAAGCCAAATGAAAATAAGCTGAAACAACCGGAACAAGCAGCACCAGGAGCAGGGGGAGAACAACCAGCACCAGGAGCAGGGGGAGAACGACCAGCACCAGGAGCAGGGGGAGAACAACCAGCACCAGGAGCAGGGGGAGAACAACCAGCACCAGGAGCAGGGGGAGAACAACCAGCACCAGGAGCAGGGGGAGAACAACCAGCACCAGGAGCAGGGGGAGAACGACCAGCACCAGGAGCAGGGGGAGAACAACCAGCACCAGGAGCAGGGGGAGAACAACCAGCACCAGGAGCAGGGGGAGAACAACCAGCACCAGGAGCAGGGGGAGAACGACCAGCACCAGGAGCAGGGGGAGAACAACCAGCACCAGGAGCAGGGGGAGAACGACCAGCACCAGGAGCAGGGGGAGAACAACCAGCACCAGCACCAAGGAGGGAACAACCAGCACCAGGACCAGGTGCGGGAGATGGAGCACGAGGAGGAAACGCAGGGGCAGGTAAAGGACAGGGACAAAACAATCAGGGTGCGAATGTCCCAAATGAAAAAGTTGTGAATGATTACCTACACAAAATTAGATCTAGCGTTACCACCGAGTGGACTCCATGCAGTGTAACCTGTGGAAATGGTGTAAGAATTAGAAGAAGACAGAATGCTGGTAATAAAAAGGCAGAGGACCTTACTATGGATGACCTTGAGGTGGAAGCTTGTGTAATGGATAAGTGCGCTGGCATATTTAACGTTGTGAGTAATTCATTAGGGTTAGTCATATTGTTAGTCCTAGCATTATTCAATTAA---------------------------------------------------------------------------------------------------------------------------------------------------------------------------------------------------------------------------------------------------------------------------------------------------------------------------------------------------------------------------------------------------------------------------------------------------------

>GU002474 Human Sarawak

ATGAGGAACTTCATTCTCTTGGCCGTCTCCTCCATCCTGCTGGTGGACTTGCTCCCCACACACTTCGAACATAATGTAGATCTCTCCAGGGCCATAAATGTAAATGGAGTAAGCTTCAATAATGTAGACACCAGTTCACTTGGCGCAGCACAGGTAAGACAAAGTGCTAGTCGAGGCAGAGGACTTGGTGAGAAGCGAAAAGAAGGAGCTGATAAAGAAAAGAAAAAAGAAAAAGAAGAAGAACCAAAGAAGCCAAATGAAAATAAGCTGAAACAACCGGAACAAGCAGCACCAGGAGCAGGGGGAGAACAACCAGCACCAGGAGCAGGGGGAGAACGACCAGCACCAGGAGCAGGGGGAGAACAACCAGCACCAGGAGCAGGGGGAGAACAACCAGCACCAGGAGCAGGGGGAGAACAACCAGCACCAGGAGCAGGGGGAGAACAACCAGCACCAGGAGCAGGGGGAGAACGACCAGCACCAGGAGCAGGGGGAGAACAACCAGCACCAGGAGCAGGGGGAGAACAACCAGCACCAGGAGCAGGGGGAGAACAACCAGCACCAGGAGCAGGGGGAGAACGACCAGCACCAGGAGCAGGGGGAGAACAACCAGCACCAGGAGCAGGGGGAGAACGACCAGCACCAGGAGCAGGGGGAGAACAACCAGCACCAGCACCAAGGAGGGAACAACCAGCACCAGGACCAGGTGCGGGAGATGGAGCACGAGGAGGAAACGCAGGGGCAGGTAAAGGACAGGGACAAAACAATCAGGGTGCGAATGTCCCAAATGAAAAAGTTGTGAATGATTACCTACACAAAATTAGATCTAGCGTTACCACCGAGTGGACTCCATGCAGTGTAACCTGTGGAAATGGTGTAAGAATTAGAAGAAGACAGAATGCTGGTAATAAAAAGGCAGAGGACCTTACTATGGATGACCTTGAGGTGGAAGCTTGTGTAATGGATAAGTGCGCTGGCATATTTAACGTTGTGAGTAATTCATTAGGGTTAGTCATATTGTTAGTCCTAGCATTATTCAATTAA---------------------------------------------------------------------------------------------------------------------------------------------------------------------------------------------------------------------------------------------------------------------------------------------------------------------------------------------------------------------------------------------------------------------------------------------------------

>GU002473 Human Sarawak

ATGAGGAACTTCATTCTCTTGGCCGTCTCCTCCATCCTGCTGGTGGACTTGCTCCCCACACACTTCGAACATAATGTAGATCTCTCCAGGGCCATAAATGTAAATGGAGTAAGCTTCAATAATGTAGACACCAGTTCACTTGGCGCAGCACAGGTAAGACAAAGTGCTAGTCGAGGCAGAGGACTTGGTGAGAAGCGAAAAGAAGGAGCTGATAAAGAAAAGAAAAAAGAAAAAGAAGAAGAACCAAAGAAGCCAAATGAAAATAAGCTGAAACAACCGGAACAAGCAGCACCAGGAGCAGGGGGAGAACAACCAGCACCAGGAGCAGGGGGAGAACGACCAGCACCAGGAGCAGGGGGAGAACAACCAGCACCAGGAGCAGGGGGAGAACAACCAGCACCAGGAGCAGGGGGAGAACAACCAGCACCAGGAGCAGGGGGAGAACAACCAGCACCAGGAGCAGGGGGAGAACGACCAGCACCAGGAGCAGGGGGAGAACAACCAGCACCAGGAGCAGGGGGAGAACAACCAGCACCAGGAGCAGGGGGAGAACAACCAGCACCAGGAGCAGGGGGAGAACGACCAGCACCAGGAGCAGGGGGAGAACAACCAGCACCAGGAGCAGGGGGAGAACGACCAGCACCAGGAGCAGGGGGAGAACAACCAGCACCAGCACCAAGGAGGGAACAACCAGCACCAGGACCAGGTGCGGGAGATGGAGCACGAGGAGGAAACGCAGGGGCAGGTAAAGGACAGGGACAAAACAATCAGGGTGCGAATGTCCCAAATGAAAAAGTTGTGAATGATTACCTACACAAAATTAGATCTAGCGTTACCACCGAGTGGACTCCATGCAGTGTAACCTGTGGAAATGGTGTAAGAATTAGAAGAAGACAGAATGCTGGTAATAAAAAGGCAGAGGACCTTACTATGGATGACCTTGAGGTGGAAGCTTGTGTAATGGATAAGTGCGCTGGCATATTTAACGTTGTGAGTAATTCATTAGGGTTAGTCATATTGTTAGTCCTAGCATTATTCAATTAA---------------------------------------------------------------------------------------------------------------------------------------------------------------------------------------------------------------------------------------------------------------------------------------------------------------------------------------------------------------------------------------------------------------------------------------------------------

>GU002472 Human Sarawak

ATGAGGAACTTCATTCTCTTGGCCGTCTCCTCCATCCTGCTGGTGGACTTGCTCCCCACACACTTCGAACATAATGTAGATCTCTCCAGGGCCATAAATGTAAATGGAGTAAGCTTCAATAATGTAGACACCAGTTCACTTGGCGCAGCACAGGTAAGACAAAGTGCTAGTCGAGGCAGAGGACTTGGTGAGAAGCGAAAAGAAGGAGCTGATAAAGAAAAGAAAAAAGAAAAAGAAGAAGAACCAAAGAAGCCAAATGAAAATAAGCTGAAACAACCGGAACAAGCAGCACCAGGAGCAGGGGGAGAACAACCAGCACCAGGAGCAGGGGGAGAACGACCAGCACCAGGAGCAGGGGGAGAACAACCAGCACCAGGAGCAGGGGGAGAACAACCAGCACCAGGAGCAGGGGGAGAACAACCAGCACCAGGAGCAGGGGGAGAACAACCAGCACCAGGAGCAGGGGGAGAACGACCAGCACCAGGAGCAGGGGGAGAACAACCAGCACCAGGAGCAGGGGGAGAACAACCAGCACCAGGAGCAGGGGGAGAACAACCAGCACCAGGAGCAGGGGGAGAACGACCAGCACCAGGAGCAGGGGGAGAACAACCAGCACCAGGAGCAGGGGGAGAACGACCAGCACCAGGAGCAGGGGGAGAACAACCAGCACCAGCACCAAGGAGGGAACAACCAGCACCAGGACCAGGTGCGGGAGATGGAGCACGAGGAGGAAACGCAGGGGCAGGTAAAGGACAGGGACAAAACAATCAGGGTGCGAATGTCCCAAATGAAAAAGTTGTGAATGATTACCTACACAAAATTAGATCTAGCGTTACCACCGAGTGGACTCCATGCAGTGTAACCTGTGGAAATGGTGTAAGAATTAGAAGAAGACAGAATGCTGGTAATAAAAAGGCAGAGGACCTTACTATGGATGACCTTGAGGTGGAAGCTTGTGTAATGGATAAGTGCGCTGGCATATTTAACGTTGTGAGTAATTCATTAGGGTTAGTCATATTGTTAGTCCTAGCATTATTCAATTAA---------------------------------------------------------------------------------------------------------------------------------------------------------------------------------------------------------------------------------------------------------------------------------------------------------------------------------------------------------------------------------------------------------------------------------------------------------

>GU002471 Human Sarawak

ATGAGGAACTTCATTCTCTTGGCCGTCTCCTCCATCCTGCTGGTGGACTTGCTCCCCACACACTTCGAACATAATGTAGATCTCTCCAGGGCCATAAATGTAAATGGAGTAAGCTTCAATAATGTAGACACCAGTTCACTTGGCGCAGCACAGGTAAGACAAAGTGCTAGTCGAGGCAGAGGACTTGGTGAGAAGCGAAAAGAAGGAGCTGATAAAGAAAAGAAAAAAGAAAAAGAAGAAGAACCAAAGAAGCCAAATGAAAATAAGCTGAAACAACCGGAACAAGCAGCACCAGGAGCAGGGGGAGAACAACCAGCACCAGGAGCAGGGGGAGAACGACCAGCACCAGGAGCAGGGGGAGAACAACCAGCACCAGGAGCAGGGGGAGAACAACCAGCACCAGGAGCAGGGGGAGAACAACCAGCACCAGGAGCAGGGGGAGAACAACCAGCACCAGGAGCAGGGGGAGAACGACCAGCACCAGGAGCAGGGGGAGAACAACCAGCACCAGGAGCAGGGGGAGAACAACCAGCACCAGGAGCAGGGGGAGAACAACCAGCACCAGGAGCAGGGGGAGAACGACCAGCACCAGGAGCAGGGGGAGAACAACCAGCACCAGGAGCAGGGGGAGAACGACCAGCACCAGGAGCAGGGGGAGAACAACCAGCACCAGCACCAAGGAGGGAACAACCAGCACCAGGACCAGGTGCGGGAGATGGAGCACGAGGAGGAAACGCAGGGGCAGGTAAAGGACAGGGACAAAACAATCAGGGTGCGAATGTCCCAAATGAAAAAGTTGTGAATGATTACCTACACAAAATTAGATCTAGCGTTACCACCGAGTGGACTCCATGCAGTGTAACCTGTGGAAATGGTGTAAGAATTAGAAGAAGACAGAATGCTGGTAATAAAAAGGCAGAGGACCTTACTATGGATGACCTTGAGGTGGAAGCTTGTGTAATGGATAAGTGCGCTGGCATATTTAACGTTGTGAGTAATTCATTAGGGTTAGTCATATTGTTAGTCCTAGCATTATTCAATTAA---------------------------------------------------------------------------------------------------------------------------------------------------------------------------------------------------------------------------------------------------------------------------------------------------------------------------------------------------------------------------------------------------------------------------------------------------------

>JQ219896 Human Singapore

ATGAAGAACTTCATTCTCTTGGCCGTCTCCTCCATCCTGCTGGTGGACTTGCTCCCCACACACTTCGAACATAATGTAGATCTCTCCAGGGCCATAAATGTAAATGGAGTAAGCTTCAATAATGTAGACACCAGTTCACTTGGCGCAGCACAGGTGAGACAAAGTGCTAGCCGAGGCAGAGGACTTGGTGAGAAGCCAAAAGAAGGAGCTGATAAAGAAAAGAAAAAAGAAAAAGAAGAAGAACCAAAGAAGCCAAATGAAAATAAGCTGAAACAACCGGATGCAGTACCAGGGGGCGAAGAACCAGCACCAGGAAGGGAACAGCCAGCACCAGGAAGGGAGGAACCAGCACCAGGAAGGGAACAGCCAGCACCAGGAAGGGAAGAACCAGCACCAGGAAGGGAACAGCCAGCACCAGGAAGGGAAGAACCAGCGCCAGGAAGGGAACAGCCAGCACCAGGAAGGGAACAGCCAGCACCAGGAAGGGAGGAACCAGCACCAGGAAGGGAACAACCAGCACCAGGAAGGGAGGAACCAGCACCAGGAAGGGAACAACCAGCACCAGGAAGGGAACAGCCAGCACCAGGAAGGGAACAGCCAGCACCGGGGGGTGAACAACCAGCACCAGGAAGGGAACAGCCAGCACCGGGTGGTGAACAACCAGCACCAGCACCAGGAGCAGGTGCGGGAGATGGAGCACGAGGAGGAAACGCAGGGGCAGGGAAAGGACAGGGACAAAACAATCAGGGTGCAAATGTCCCAAATGAAAAAGTTGTGAATGATTACCTACACAAAATTAGATCTAGCGTTACCACCGAGTGGACTCCATGCAGTGTAACCTGTGGAAATGGTGTAAGAATTAGAAGAAAAGGTCATGCAGGTAATAAAAAGGCAGAGGACCTTACTATGGATGACCTTGAGGTGGAAGCTTGTGTAATGGATAAGTGCGCTGGCATATTTAACGTTGTGAGTAATTCATTAGGCTTAGTCATATTGTTAGTCCTAGCATTATTCAATTAA---------------------------------------------------------------------------------------------------------------------------------------------------------------------------------------------------------------------------------------------------------------------------------------------------------------------------------------------------------------------------------------------------------------------------------------------------------------------------------

>JQ219895 Human Singapore

ATGAAGAACTTCATTCTCTTGGCCGTCTCCTCCATCCTGCTGGTGGACTTGCTCCCCACACACTTCGAACATAATGTAGATCTCTCCAGGGCCATAAATGTAAATGGAGTAAGCTTCAATAATGTAGACACCAGTTCACTTGGCGCAGCACAGGTGAGACAAAGTGCTAGCCGAGGCAGAGGACTTGGTGAGAAGCCAAAAGAAGGAGCTGATAAAGAAAAGAAAAAAGAAAAAGGAAAAGAAAAAGAAGAAGAACCAAAGAAGCCAAATGAAAATAAGCTGAAACAACCGAATGAAGGACAACCACAAGCACAGGGTGATGGAGCAAATGCAGGACAACCACAAGCACAAGGAGATGGAGCAAATGCAGGACAACCACAAGCACAGGGTGATGGAGCAAATGCAGGACAACCACAAGCACAGGGTGATGGAGCAAATGCAGGACAACCACAAGCACAAGGAGATGGAGCAAATGCAGGACAACCACAAGCACAGGGTGATGGAGCAAATGCAGGGCAACCACAAGCACAGGGTGATGGAGCAAATGCAGGACAACCACAAGCACAAGGAGATGGAGCAAATGCAGGACAACCACAAGCACAAGGAGATGGAGCAAATGCAGGACAACCACAAGCACAGGGTGATGGAGCAAATGCAGGACAACCACAAGCACAGGGTGATAGGGCGAATGCAGGACAACCACAAGCACAAGGAGATGGGGCAAATGTACCACGACAAGGAAGAAACGGGGGAGGTGCACCAGCAGGAGGAAATGAGGGGAATAAACAAGCAGGAAAAGGACAGGGACAAAACAATCAGGGTGCGAATGCCCCAAATGAAAAAGTTGTGAATGATTACCTACACAAAATTAGATCTAGCGTTACCACCGAGTGGACTCCATGCAGTGTAACCTGTGGAAATGGTGTAAGAATTAGAAGAAAAGCTCATGCAGGTAATAAAAAGGCAGAGGACCTTACTATGGATGACCTTGAGGTGGAAGCTTGTGTAATGGATAAGTGCGCTGGCATATTTAACGTTGTGAGTAATTCATTAGGCTTAGTCATATTGTTAGTCCTAGCATTATTCAATTAA---------------------------------------------------------------------------------------------------------------------------------------------------------------------------------------------------------------------------------------------------------------------------------------------------------------------------------------------------------------------------------------------------------

>JQ219894 Human Singapore

ATGAAGAACTTCATTCTCTTGGCCGTCTCCTCCATCCTGCTGGTGGACTTGCTCCCCACACACTTCGAACATAATGTAGATCTCTCCAGGGCCATAAATGTAAATGGAGTAAGCTTCAATAATGTAGACACCAGTTCACTTGGCGCAGCACAGGTGAGACAAAGTGCTAGCCGAGGCAGAGGACTTGGTGAGAAGCCAAAAGAAGGAGCTGATAAAGAAAAGAAAAAAGAAAAAGAAAAAGAAAAAGAAGAACCAAAGAAGCCAAATGAAAATAAGCTGAAACAACCGGAACAACCAGCAGCAGGAGCAGGGGGCGAACAACCAGCAGCAGGAGCAGGAGGCGAACAACCAGCAGCAGGAGCAGGAGGCGAACAACCAGCAGCAGGAGCAGGAGGCGAACAACCAGCGGCAGGAGCAGGGGGCGAACAACCAGCAGCAGGAGCAGGAGGCGAACAACCAGCAGCAGGAGCAGGAGGCGAACAACCAGCAGCAGGAGCAGGAGGCGAACAACCAGCAGCAGGAGCAGGAGGCGAACAACCAGCAGCAGGAGCAGGGGGCGAACAACCAGCAGCAGGAGCAGGAGGCGAACAACCAGCACCAGCACCAAGGAGGGAACAACCAGCAGCAGGAGCAGGGGGCGAACAACCAGCACCAGCACCAAGGAGGGAACAACCAGCACCAGGAGCAGGTGCGGGAGATGGAGCACGAGGAGGAAACGCAGGGGCAGGTAAAGGACAGGGACAAAACAATCAGGGTGCGAATGTCCCAAATGAAAAAGTTGTGAATGATTACCTACACAAAATTAGATCTAGCGTTACCACCGAGTGGACTCCATGCAGTGTAACCTGTGGAAATGGTGTAAGAATTAGAAGAAGACAGAATGCTGGTAATAAAAAGGCAGAGGACCTTACTATGGATGACCTTGAGGTGGAAGCTTGTGTAATGGATAAGTGCGCTGGCATATTTAACGTTGTGAGTAATTCATTAGGCTTAGTCATATTGTTAGTCTTAGCATTATTCAATTAA---------------------------------------------------------------------------------------------------------------------------------------------------------------------------------------------------------------------------------------------------------------------------------------------------------------------------------------------------------------------------------------------------------------------------------------------------------------------------

>JQ219893 Human Singapore

ATGAAGAACTTCATTCTCTTGGCCGTCTCCTCCATCCTGCTGGTGGACTTGCTCCCCACACACTTCGAACATAATGTAGATCTCTCCAGGGCCATAAATGTAAATGGAGTAAGCTTCAATAATGTAGACACCAGTTCACTTGGCGCAGCACAGGTGAGACAAAGTGCTAGCCGAGGCAGAGGACTTGGTGAGAAGCCAAAAGAAGGAGCTGATAAAGAAAAGAAAAAAGAAAAAGAAAAAGAAAAAGAAGAACCAAAGAAGCCAAATGAAAATAAGCTGAAACAACCGGAACAACCAGCAGCAGGAGCAGGGGGCGAACAACCAGCAGCAGGAGCAGGAGGCGAACAACCAGCAGCAGGAGCAGGAGGCGAACAACCAGCAGCAGGAGCAGGAGGCGAACAACCAGCGGCAGGAGCAGGGGGCGAACAACCAGCAGCAGGAGCAGGAGGCGAACAACCAGCAGCAGGAGCAGGAGGCGAACAACCAGCAGCAGGAGCAGGAGGCGAACAACCAGCAGCAGGAGCAGGAGGCGAACAACCAGCAGCAGGAGCAGGGGGCGAACAACCAGCAGCAGGAGCAGGAGGCGAACAACCAGCACCAGCACCAAGGAGGGAACAACCAGCAGCAGGAGCAGGGGGCGAACAACCAGCACCAGCACCAAGGAGGGAACAACCAGCACCAGGAGCAGGTGCGGGAGATGGAGCACGAGGAGGAAACGCAGGGGCAGGTAAAGGACAGGGACAAAACAATCAGGGTGCGAATGTCCCAAATGAAAAAGTTGTGAATGATTACCTACACAAAATTAGATCTAGCGTTACCACCGAGTGGACTCCATGCAGTGTAACCTGTGGAAATGGTGTAAGAATTAGAAGAAGACAGAATGCTGGTAATAAAAAGGCAGAGGACCTTACTATGGATGACCTTGAGGTGGAAGCTTGTGTAATGGATAAGTGCGCTGGCATATTTAACGTTGTGAGTAATTCATTAGGCTTAGTCATATTGTTAGTCTTAGCATTATTCAATTAA---------------------------------------------------------------------------------------------------------------------------------------------------------------------------------------------------------------------------------------------------------------------------------------------------------------------------------------------------------------------------------------------------------------------------------------------------------------------------

>JQ219892 Human Singapore

ATGAAGAACTTCATTCTCTTGGCCGTCTCCTCCATCCTGCTGGTGGACTTGCTCCCCACACACTTCGAACATAATGTAGATCTCACCAGGGCCATAAATGTAAATGGAGTAAGCTTCAATAATGTAGACACCAGTTCACTTGGCGCAGCACAGGTAAGACAAAGTGCTAGCCGAGGCAGAGGACTTGGTGAGAAGCCAAAAGAAGGAGATGATAAAGAAAAGAAAAAAGAAAAAGAAAAAGAAGAAGAACCAAAGAAGCTAAATGAAAATAATCCGAAAAAACCGAATGAAGAAGGTGATGGAGCTAAGCTGAAACAACCGAATGAAGAAGGTGATGGAGCTAAGCTGAAACAACCGAATGCAGAAGGTGGAGCTAAGCTGAAACAACCGAATGCAGAAGGTGGAGCAAATGCAGGACAACCGAATGCAGAAGGTGGAGCAAATGCAGGACAACCGAATGCAGAAGGTGGAGCAAATGCAGGACAACCGAATGCAGGTGGAGCAAATGCAGGACAACCGAATGCAGAAGGTGGAGCAAATGCAGGACAACCGAATGCAGAAGGTGGAGCAAATGCAGGACAACCGAATGCAGAAGGTGGAGCAAATGCACGACAACCGAATGCAGAAGGTGGAGCAAATGCACGACAACCTAATGCAGAAGGTGGAGCAAATGCAGGACAACCGAATGCAGAAGGTGGAGCAAATGCAGGACAACCGAATGCAGAAGGTGGAGCAAATGCAGGACAACCGAATGCAGAAGGTGGAGCAAATGCACGACAACCTAATGCAGAAGGTGGAGCAAACGCAGGACAACCGAATGCAGAAGGTGGAGCAAATGCAGGACAACCGAATGCAGAAGGTGGAGCAAATGCACGACAGCCACAGGCAGAAGGTGGTGGAGCAAATGCACGACAGCCACAGGCAGAAGGTGGTGGAGCAAATGCACGACAAGGAGGAAATGAGGGGAATAAACAAGCAGGAAAAGGACAGGGACAAAACAATCAGGGTGCGAATGCCCCAAATGAAAAAGTTGTAAATGATTACCTACAGAAAATTAGATCTAGCGTTACCATCGAGTGGACTCCATGCAGTGTAACCTGTGGAAATGGTGTAAGAATTAGAAGAAGAGCTCATGCAGATAAGAAAAAGGCAGAGGACCTTACTATGGATGACCTTGAAGTGGAAGCTTGTGTAATGGATAAGTGTGCTGGCATATTTAACGTTGTGAGTAATTCATTAGGGTTAGTAATATTGTTAGTCCTAGCATTATTCAATTAA---------------------------------------------------------------------------------------------------------------------------------------------------------------------------------------------------------------

>JQ219891 Human Singapore

ATGAAGAACTTCATTCTCTTGGCCGTCTCCTCCATCCTGCTGGTGGACTTGCTCCCCACACACTTCGAACATAATGTAGATCTCTCCAGGGCCATAAATGTAAATGGAGTAAGCTTCAATAATGTAGACACCAGTTCACTTGGCGCAGCACAGGTAAGACAAAGTGCTAGCCGAGGCAGAGGACTTGGTGAGAAGCCAAAAGAAGGAGATGATAAAGAAAAGAAAAAAGAAAAAGAAAAAGAAGAAGAACCAAAGAAGCTAAATGAAAATAATCCGAAAAAACCGAATGAAGAAGGTGATGGAGCTAAGCTGAAACAACCGAATGAAGAAGGTGATGGAGCTAAGCTGAAACAACCGAATGCAGAAGGTGGAGCTAAGCTGAAACAACCGAATGCAGAAGGTGGAGCAAATGCAGGACAACCGAATGCAGAAGGTGGAGCAAATGCAGGACAACCGAATGCAGAAGGTGGAGCAAATGCAGGACAACCGAATGCAGGTGGAGCAAATGCAGGACAACCGAATGCAGAAGGTGGAGCAAATGCAGGACAACCGAATGCAGAAGGTGGAGCAAATGCAGGACAACCGAATGCAGAAGGTGGAGCAAATGCACGACAACCGAATGCAGAAGGTGGAGCAAATGCACGACAACCTAATGCAGAGGGTGGAGCAAATGCAGGACAACCGAATGCAGAAGGTGGAGCAAATGCAGGACAACCGAATGCAGAAGGTGGAGCAAATGCAGGACAACCGAATGCAGAAGGTGGAGCAAATGCACGACAACCTAATGCAGAAGGTGGAGCAAACGCAGGACAACCGAATGCAGAAGGTGGAGCAAATGCAGGACAACCGAATGCAGAAGGTGGAGCAAATGCAGGACAACCGAATGCAGAAGGTGGAGCAAATGCAGGACAACCGAATGCAGAAGGTGGAGCAAATGCACGACAACCTAATGCAGAAGGTGGAGCAAACGCAGGACAACCGAATGCAGAAGGTGGAGCAAATGCAGGACAACCGAATGCAGAAGGTGGAGCAAATGCACGACAGCCACAGGCAGAAGGTGGTGGAGCAAATGCACGACAGCCACAGGCAGAAGGTGGTGGAGCAAATGCACGACAAGGAGGAAATGAGGGGAATAAACAAGCAGGAAAAGGACAGGGACAAAACAATCAGGGTGCGAATGCCCCAAATGAAAAAGTTGTAAATGATTACCTACAGAAAATTAGATCTAGCGTTACCATCGAGTGGACTCCATGCAGTGTAACCTGTGGAAATGGTGTAAGAATTAGAAGAAGAGCTCATGCAGATAAGAAAAAGGCAGAGGACCTTACTATGGATGACCTTGAAGTGGAAGCTTGTGTAATGGATAAGTGTGCTGGCATATTTAACGTTGTGAGTAATTCATTAGGGTTAGTAATATTGTTAGTCCTAGCATTATTCAATTAA------------------------------------------

>JQ219890 Human Singapore

ATGAAGAACTTCATTCTCTTGGCCGTCTCCTCCATCCTGCTGGTGGACTTGCTCCCCACACACTTCGAACATAATGTAGATCTCTCCAGGGCCATAAATGTAAATGGAGTAAGCTTCAATAATGTGGACACCAGTTCACTTGGCGCAGCACAGGTAAGACAAAGTGCTAGCCGAGGCAGAGGACTTGGTGAGAAGCCAAAAGAAGGAGATGATAAAGAAAAGAAAAAAGAAAAAGAAAAAGAAGAAGAACCAAAGAAGCTAAATGAAAATAATCCGAAAAAACCGAATGAAGAAGGTGATGGAGCTAAGCTGAAACAACCGAATGAAGAAGGTGATGGAGCTAAGCTGAAACAACCGAATGCAGAAGGTGGAGCTAAGCTGAAACAACCGAATGCAGAAGGTGGAGCAAATGCAGGACAACCGAATGCAGAAGGTGGAGCAAATGCAGGACAACCGAATGCAGAAGGTGGAGCAAATGCAGGACAACCGAATGCAGGTGGGGCAAATGCAGGACAACCGAATGCAGAAGGTGGAGCAAATGCAGGACAACCGAATGCAGAAGGTGGAGCAAATGCAGGACAACCGAATGCAGAAGGTGGAGCAAATGCACGACAACCGAATGCAGAAGGTGGAGCAAATGCACGACAACCTAATGCAGAAGGTGGAGCAAATGCAGGACAACCGAATGCAGAAGGTGGAGCAAATGCAGGACAACCGAATGCAGAAGGTGGAGCAAATGCAGGACAACCGAATGCAGAAGGTGGAGCAAATGCACGACAACCTAATGCAGAAGGTGGAGCAAACGCAGGACAACCGAATGCAGAAGGTGGAGCAAATGCAGGACAACCGAATGCAGAAGGTGGAGCAAATGCACGACAGCCACAGGCAGAAGGTGGTGGAGCAAATGCACGACAGCCACAGGCAGAAGGTGGTGGAGCAAATGCACGACAAGGAGGAAATGAGGGGAATAAACAAGCAGGAAAAGGACAGGGACAGAACAATCAGGGTGCGAATGCCCCAAATGAAAAAGTTGTAAATGATTACCTACAGAAAATTAGATCTAGCGTTACCATCGAGTGGACTCCATGCAGTGTAACCTGTGGAAATGGTGTAAGAATTAGAAGAAGAGCTCATGCAGATAAGAAAAGGGCAGAGGACCTTACTATGGATGACCTTGAGGTGGAAGCTTGTGTAATGGATAAGTGTGCTGGCATATTTAACGTTGTGAGTAATTCATTAGGGTTAGTAATATTGTTAGTCCTAGCATTATTCAATTAA---------------------------------------------------------------------------------------------------------------------------------------------------------------------------------------------------------------

>JQ219889 Human Singapore

ATGAGGAACTTCATTCTCTTGGCCGTCTCCTCCATCCTGCTGGTGGACTTGCTCCCCACACACTTCGAACATAATGTAGATCTCTCCAGGGCCATAAATGTAAATGGAGTAAGCTTCAATAATGTAGACACCAGTTCACTTGGCGCAGCACAGGTAAGACAAAGTGCTAGCCGAGGCAGAGGACTTGGTGAGAAGCCAAAAGAAGGAGATGATAAAGAAAAGAAAAAAGAAAAAGAAAAAGAAGAAGAACCAAAGAAGCTAAATGAAAATAATCCGAAAAAACCGAATGAAGAAGGTGATGGAGCTAAGCTGAAACAACCGAATGAAGAAGGTGATGGAGCTAAGCTGAAACAACCGAATGCAGAAGGTGGAGCTAAGCTGAAACAACCGAATGCAGAAGGTGGAGCAAATGCAGGACAACCGAATGCAGAAGGTGGAGCAAATGCAGGACAACCGAATGCAGAAGGTGGAGCAAATGCAGGACAACCGAATGCAGGTGGAGCAAATGCAGGACAACCGAATGCAGAAGGTGGAGCAAATGCAGGACAACCGAATGCAGAAGGTGGAGCAAATGCAGGACAACCGAATGCAGAAGGTGGAGCAAATGCACGACAACCGAATGCAGAAGGTGGAGCAAATGCACGACAACCTAATGCAGAAGGTGGAGCAAATGCAGGACAACCGAATGCAGAAGGTGGAGCAAATGCAGGACAACCGAATGCAGAAGGTGGAGCAAATGCAGGACAACCGAATGCAGAAGGTGGAGCAAATGCACGACAACCTAATGCAGAAGGTGGAGCAAACGCAGGACAACCGAATGCAGAAGGTGGAGCAAATGCAGGACAACCGAATGCAGAAGGTGGAGCAAATGCACGACAGCCACAGGCAGAAGGTGGTGGAGCAAATGCACGACAGCCACAGGCAGAAGGTGGTGGAGCAAATGCACGACAAGGAGGAAATGAGGGGAATAAACAAGCAGGAAAAGGACAGGGACAAAACAATCAGGGTGCGAATGCCCCAAATGAAAAAGTTGTAAATGATTACCTACAGAAAATTAGATCTAGCGTTACCATCGAGTGGACTCCATGCAGTGTAACCTGTGGAAATGGTGTAAGAATTAGAAGAAGAGCTCATGCAGATAAGAAAAAGGCAGAGGACCTTACTATGGATGACCTTGAAGTGGAAGCTTGTGTAATGGATAAGTGTGCTGGCATATTTAACGTTGTGAGTAATACATTAGGGTTAGTAATATTGTTAGTCCTAGCATTATTCAATTAA---------------------------------------------------------------------------------------------------------------------------------------------------------------------------------------------------------------

>JQ219888 Human Singapore

ATGAAGAACTTCATTCTCTTGGCCGTCTCCTCCATCCTGCTGGTGGACTTGCTCCCCACACACTTCGAACATAATGTAGATCTCTCCAGGGCCATAAATGTAAATGGAGTAAGCTTCAATAATGTAGACACCAGTTCACTTGGCGCAGCACAGGTAAGACAAAGTGCTAGCCGAGGCAGAGGACTTGGTGAGAAGCCAAAAGAAGGAGATGATAAAGAAAAGAAAAAAGAAAAAGAAAAAGAAGAAGAACCAAAGAAGCTAAATGAAAATAATCCGAAAAAACCGAATGAAGAAGGTGATGGAGCTAAGCTGAAACAACCGAATGAAGAAGGTGATGGAGCTAAGCTGAAACAACCGAATGCAGAAGGTGGAGCTAAGCTGAAACAACCGAATGCAGAAGGTGGAGCAAATGCAGGACAACCGAATGCAGAAGGTGGAGCAAATGCAGGACAACCGAATGCAGAAGGTGGAGCAAATGCAGGACAACCGAATGCAGGTGGAGCAAATGCAGGACAACCGAATGCAGAAGGTGGAGCAAATGCAGGACAACCGAATGCAGAAGGTGGAGCAAATGCAGGACAACCGAATGCAGAAGGTGGAGCAAATGCACGACAACCGAATGCAGAAGGTGGAGCAAATGCACGACAACCTAATGCAGGAGGTGGAGCAAATGCAGGACAACCGAATGCAGAAGGTGGAGCAAATGCAGGACAACCGAATGCAGAAGGTGGAGCAAATGCAGGACAACCGAATGCAGAAGGTGGAGCAAATGCACGACAGCCTAATGCAGAAGGTGGAGCAAACGCAGGACAACCGAATGCAGAAGGTGGAGCAAATGCAGGACAACCGAATGCAGAAGGTGGAGCAAATGCACGACAGCCACAGGCAGAAGGTGGTGGAGCAAATGCACGACAGCCACAGGCAGAAGGTGGTGGAGCAAATGCACGACAAGGAGGAAATGAGGGGAATAAACAAGCAGGAAAAGGACAGGGACAAAACAATCAGGGTGCGAATGCCCCAAATGAAAAAGTTGTAAATGATTACCTACAGAAAATTAGATCTAGCGTTACCATCGAGTGGACTCCATGCAGTGTAACCTGTGGAAATGGTGTAAGAATTAGAAGAAGAGCTCATGCAGATAAGAAAAAGGCAGAGGACCTTACTGTGGATGACCTTGAAGTGGAAGCTTGTGTAATGGATAAGTGTGCTGGCATATTTAACGTTGTGAGTAATTCATTAGGGTTAGTAATATTGTTAGTCCTAGCATTATTCAATTAA---------------------------------------------------------------------------------------------------------------------------------------------------------------------------------------------------------------

>JQ219887 Human Singapore

ATGAAGAACTTCATTCTCTTGGCCGTCTCCTCCATCCTGCTGGTGGACTTGCTCCCCACACACTTCGAACATAATGTAGATCTCTCCAGGGCCATAAATGTAAATGGAGTAAGCTTCAATAATGTAGACACCAGTTCACTTGGCGCAGCACAGGTAAGACAAAGTGCTAGCCGAGGCAGAGGACTTGGTGAGAAGCCAAAAGAAGGAGATGATAAAGAAAAGAAAAAAGAAAAAGAAAAAGAAGAAGAACCAAAGAAGCTAAATGAAAATAATCCGAAAAAACCGAATGAAGAAGGTGATGGAGCTAAGCTGAAACAACCGAATGAAGAAGGTGATGGAGCTAAGCTGAAACAACCGAATGCAGAAGGTGGAGCTAAGCTGAAACAACCGAATGCAGAAGGTGGAGCAAATGCAGGACAACCGAATGCAGAAGGTGGAGCAAATGCAGGACAACCGAATGCAGAAGGTGGAGCAAATGCAGGACAACCGAATGCAGGTGGAGCAAATGCAGGACAACCGAATGCAGAAGGTGGAGCAAATGCAGGACAACCGAATGCAGAAGGTGGAGCAAATGCAGGACAACCGAATGCAGAAGGTGGAGCAAATGCACGACAACCGAATGCAGAAGGTGGAGCAAATGCACGACAACCTAATGCAGAAGGTGGAGCAAATGCAGGACAACCGAATGCAGAAGGTGGAGCAAATGCAGGACAACCGAATGCAGAAGGTGGAGCAAATGCAGGACAACCGAATGCAGAAGGTGGAGCAAATGCACGACAACCTAATGCAGAAGGTGGAGCAAACGCAGGACAACCGAATGCAGAAGGTGGAGCAAATGCAGGACAACCGAATGCAGAAGGTGGAGCAAATGCACGACAGCCACAGGCAGAAGGTGGTGGAGCAAATGCACGACAGCCACAGGCAGAAGGTGGTGGAGCAAATGCACGACAAGGAGGAAATGAGGGGAATAAACAAGCAGGAAAAGGACAGGGACAAAACAATCAGGGTGCGAATGCCCCAAATGAAAAAGTTGTAAATGATTACCTACAGAAAATTAGATCTAGCGTTACCATCGAGTGGACTCCATGCAGTGTAACCTGTGGAAATGGTGTAAGAATTAGAAGAAGAGCTCATGCAGATAAGAAAAAGGCAGAGGACCTTACTATGGATGACCTTGAAGTGGAAGCTTGTGTAATGGATAGGTGTGCTGGCATATTTAACGTTGTGAGTAATTCATTAGGGTTAGTAATATTGTTGGTCCTAGCATTATTCAATTAA---------------------------------------------------------------------------------------------------------------------------------------------------------------------------------------------------------------

>JQ219886 Human Singapore

ATGAAGAACTTCATTCTCTTGGCCGTCTCCTCCATCCTGCTGGTGGACTTGCTCCCCACACACTTCGAACATAATGTAGATCTCTCCAGGGCCATAAATGTAAATGGAGTAAGCCTCAATAATGTAGACACCAGTTCACTTGGCGCAGCACAGGTAAGACAAAGTGCTAGCCGAGGCAGAGGACTTGGTGAGAAGCCAAAAGAAGGAGATGATAAAGAAAAGAAAAAAGAAAAAGAAAAAGAAGAAGAACCAAAGAAGCTAAATGAAAATAATCCGAAAAAACCGAATGAAGAAGGTGATGGAGCTAAGCTGAAACAACCGAATGCAGAAGGTGGAGCTAAGCTGAAACAACCGAATGCAGAAGGTGGAGCAAATGCAGGACAACCGAATGCAGAAGGTGGAGCAAATGCAGGACAACCGAATGCAGAAGGTGGAGCAAATGCAGGACAACCGAATGCAGGTGGAGCAAATGCAGGACAACCGAATGCAGAAGGTGGAGCAAATGCAGGACAACCGAATGCAGAAGGTGGAGCAAATGTAGGACAACCGAATGCAGAAGGTGGAGCAAATGCACGACAACCGAATGCAGAAGGTGGAGCAAATGCACGACAACCTAATGCAGAAGGTGGAGCAAATGCAGGACAACCGAATGCAGAAGGTGGAGCAAATGCAGGACAACCGAATGCAGAAGGTGGAGCAAATGCAGGACAACCGAATGCAGAAGGTGGAGCAAATGCACGACAACCTAATGCAGAAGGTGGAGCAAACGCAGGACAACCGAATGCAGAAGGTGGAGCAAATGCAGGACAACCGAATGCAGAAGGTGGAGCAAATGCACGACAGCCACAGGCAGAAGGTGGTGGAGCAAATGCACGACAGCCACAGGCAGAAGGTGGTGGAGCAAATGCACGACAAGGAGGAAATGAGGGGAATAAACGAGCAGGAAAAGGACAGGGACAAAACAATCAGGGTGCGAATGCCCCAAATGAAAAAGTTGTAAATGATTACCTACAGAAAATTAGATCTAGCGTTACCATCGAGTGGACTCCATGCAGTGTAACCTGTGGAAATGGTGTAAGAATTAGAAGAAGAGCTCATGCAGATAAGAAAAAGGCAGAGGACCTTACTATGGATGACCTTGAAGTGGAAGCTTGTGTAATGGATAAGTGTGCTGGCATATTTAACGTTGTGAGTAATTCATTAGGGTTAGTAATATTGTTAGTCCTAGCATTATTCAATTAA---------------------------------------------------------------------------------------------------------------------------------------------------------------------------------------------------------------------------------------------------

>JQ219885 Human Singapore

ATGAAGAACTTCATTCTCTTGGCCGTCTCCTCCATCCTGCTGGTGGACTTGCTCCCCACACACTTCGAACATAATGTAGATCTCTCCAGGGCCATAAATGTAAATGGAGTAAGCTTCAATAATGTAGACACCAGTTCACTTGGCGCAGCACAGGTAAGACAAAGTGCTAGCCGAGGCAGAGGACTTGGTGAGAAGCCAAAAGAAGGAGATGATAAAGAAAAGAAAAAAGAAAAAGAAAAAGAAGAAGAACCAAAGAAGCTAAATGAAAATAATCCGAAAAAACCGAATGAAGAAGGTGATGGAGCTAAGCTGAAACAACCGAATGAAGAAGGTGATGGAGCTAAGCTGAAACAACCGAATGCAGAAGGTGGAGCAAATGCAGGACAACCGAATGCAGAAGGTGGAGCAAATGCAGGACAACCGAATGCAGAAGGTGGAGCAAATGCACGACAACCTAATGCAGAAGGTGGAGCAAACGCAGGACAACCGAATGCAGAAGGTGGAGCAAATGCAGGACAACCGAATGCAGAAGGTGGAGCAAATGCACGACAGCCACAGGCAGAAGGTGGTGGAGCAAATGCACGACAGCCACAGGCAGAAGGTGGTGGAGCAAATGCACGACAAGGAGGAAATGAGGGGAATAAACAAGCAGGAAAAGGACAGGGACAAAACAATCAGGGTGCGAATGCCCCAAATGAAAAAGTTGTAAATGATTACCTACAGAAAATTAGATCTAGCGTTACCATCGAGTGGACTCCATGCAGTGTAACCTGTGGAAATGGTGTAAGAATTAGAAGAAGAGCTCATGCAGATAAGAAAAAGGCAGAGGACCTTACTATGGATGACCTTGAAGTGGAAGCTTGTGTAATGGATAAGTGTGCTGGCATATTTAACGTTGTGAGTAATTCATTAGGGTTAGTAATATTGTTAGTCCTAGCATTATTCAATTAA------------------------------------------------------------------------------------------------------------------------------------------------------------------------------------------------------------------------------------------------------------------------------------------------------------------------------------------------------------------------------------------------------------------------------------------------------------------------------------------------------------------------------------------------------

>JQ219884 Human Singapore

ATGAAGAACTTCATTCTCTTGGCCGTCTCCTCCATCCTGCTGGTGGACTTGCTCCCCACACACTTCGAACATAATGTAGATCTCTCCAGGGCCATAAATGTAAATGGAGTAAGCTTCAATAATGTAGACACCAGTTCACTTGGCGCAGCACAGGTAAGACAAAGTGCTAGCCGAGGCAGAGGACTTGGTGAGAAGCCAAAAGAAGGAGATGATAAAGAAAAGAAAAAAGAAAAAGAAAAAGAAGAAGAACCAAAGAAGCTAAATGAAAATAATCCGAAAAAACCGAATGAAGAAGGTGATGGAGCTAAGCTGAAACAACCGAATGAAGAAGGTGATGGAGCTAAGCTGAAACAACCGAATGCAGAAGGTGGAGCTAAGCTGAAACAACCGAATGCAGAAGGTGGAGCAAATGCAGGACAACCGAATGCAGAAGGTGGAGCAAATGCAGGACAACCGAATGCAGAAGGTGGAGCAAATGCAGGACAACCGAATGCAGGTGGAGCAAATGCAGGACAACCGAATGCAGAAGGTGGAGCAAATGCAGGACAACCGAATGCAGAAGGTGGAGCAAATGCAGGACAACCGAATGCAGAAGGTGGAGCAAATGCACGACAACCGAATGCAGAAGGTGGAGCAAATGCACGACAACCTAATGCAGAAGGTGGAGCAAATGCAGGACAACCGAATGCAGAAGGTGGAGCAAATGCAGGACAACCGAATGCAGAAGGTGGAGCAAATGCAGGACAACCGAATGCAGAAGGTGGAGCAAATGCACGACAACCTAATGCAGAAGGTGGAGCAAACGCAGGACAACCGAATGCAGAAGGTGGAGCAAATGCAGGACAACCGAATGCAGAAGGTGGAGCAAATGCACGACAGCCACAGGCAGAAGGTGGTGGAGCAAATGCACGACAGCCACAGGCAGAAGGTGGTGGAGCAAATGCACGACAAGGAGGAAATGAGGGGAATAAACAAGCAGGAAAAGGACAGGGACAAAACAATCAGGGTGCGAATGCCCCAAATGAAAAAGTTGTAAATGACTACCTACAGAAAATTAGATCTAGCGTTACCATCGAGTGGACTCCATGCAGTGTAACTTGTGGAAATGGTGTAAGAATTAGAAGAAGAGCTCATGCAGATAAGAAAAAGGCAGAGGACCTTACTATGGATGACCTTGAAGTGGAAGCTTGTGTAATGGATAAGTGTGCTGGCATATTTAACGTTGTGAGTAATTCATTAGGGTTAGTAATATTGTTAGTCCTAGCATTATTCAATTAA---------------------------------------------------------------------------------------------------------------------------------------------------------------------------------------------------------------

>JQ219883 Human Singapore

ATGAGGAACTTCATTCTCTTGGCCGTCTCCTCCATCCTGCTGGTGGACTTGCTCCCCACACACTTCGAACATAATGTAGATCTCTCCAGGGCCATAAATGTAAATGGAGTAAGCTTCAATAATGTAGACACCAGTTCACTTGGCGCAGCACAGGTAAGACAAAGTGCTAGCCGAGGCAGAGGACTTGGTGAGAAGCCAAAAGAAGGAGATGATAAAGAAAAGAAAAAAGAAAAAGAAAAAGAAGAAGAACCAAAGAAGCTAAATGAAAATAATCCGAAAAAACCGAATGAAGAAGGTGATGGAGCTAAGCTGAAACAACCGAATGAAGAAGGTGATGGAGCTAAGCTGAAACAACCGAATGCAGAAGGTGGAGCTAAGCTGAAACAACCGAATGCGGAAGGTGGAGCAAATGCAGGACAACCGAATGCAGAAGGTGGAGCAAATGCAGGACAACCGAATGCAGAAGGTGGAGCAAATGCAGGACAACCGAATGCAGGTGGAGCAAATGCAGGACAACCGAATGCAGAAGGTGGAGCAAATGCAGGACAACCGAATGCAGAAGGTGGAGCAAATGCAGGACAACCGAATGCAGAAGGTGGAGCAAATGCACGACAACCGAATGCAGAAGGTGGAGCAAATGCACGACAACCTAATGCAGAAGGTGGAGCAAATGCAGGACAACCGAATGCAGAAGGTGGAGCAAATGCAGGACAACCGAATGCAGAAGGTGGAGCAAATGCAGGACAACCGAATGCAGAAGGTGGAGCAAATGCACGACAACCTAATGCAGAAGGTGGAGCAAACGCAGGACAACCGAATGCAGAAGGTGGAGCAAATGCAGGACAACCGAATGCAGAAGGTGGAGCAAATGCACGACAGCCACAGGCAGAAGGTGGTGGAGCAAATGCACGACAGCCACAGGCAGAAGGTGGTGGAGCAAATGCACGACAAGGAGGAAATGAGGGGAATAAACAAGCAGGAAAAGGACAGGGACAAAACAATCAGGGTGCGAATGCCCCAAATGAAAAAGTTGTAAATGATTACCTACAGAAAATTAGATCTAGCGTTACCATCGAGTGGACTCCATGCAGTGTAACCTGTGGAAATGGTGTAAGAATTAGAAGAAGAGCTCATGCAGATAAGAAAAAGGCAGAGGACCTTACTATGGATGACCTTGAAGTGGAAGCTTGTGTAATGGATAAGTGTGCTGGCATATTTAACGTTGTGAGTAATTCATTAGGGTTAGTAATATTGTTAGTCCTAGCATTATTCAATTAA---------------------------------------------------------------------------------------------------------------------------------------------------------------------------------------------------------------

>GU002523 P. inui_outgroup

ATGAAGAACTTCATTCTTTTGGCCGTTTCGTCCATCCTGTTGGTGGAATTGTTCCCCACACACTGCGGGCATAATGTAGATCTCTCCAGGGCCATAAATTTAAGTGGAGTAAGCTTCAATAATGTAGACGCCAGTTCACTTGGCGCAGCACACGTAAGACAAAGTGTTAGCCGAAGTAGAGGACTTGGTGAATACCCAGGAGACGAGGAAGGAGCTGTTAAGGAAAAAAAGAAGGGAAGAAGACGAAAAGGAGGAAACCCACCTGAAGAAAACCTGAAGCAACCAGGAGGAGAGGCAGGAGCAGCAGGGCAAGGAGGAGAGGCAGGAGCAGCCGGCCAAGGAGGAGAGGCAGGAGCAGCCGGCCAAGGAGGAGAGGCAGGAGCAGCAGGCCAAGGAGGAGAGGCTGGAGCAGCAGGCCAAGCAGGAGCAGCAGGACAAGGAGGAGAGGCAGGAGCAGCAGGGCAAGGAGGAGAGGCAGGAGCAGCAGGGCAAGGAGGAGAGGCAGGAGCAGCCGGCCAAGGAGGAGAGGCAGGAGCAGCAGGGCAAGGAGGAGAGGCAGGAGCAGCCGGCCAAGGAGGAGAGGCAGGAGCAGCAGGCCAAGGAGGAGAGGCTGGAGCAGCAGGCCAAGCAGGAGCAGCAGGACAAGCAGGAGCAGGAGGAGGAGCAGCAGGACAAGGAGGAGCAGCAGGACAAGGAGGAGCAGCAGGACAAGCAGGAGCAGCAGGACAAGCAGGACAAGCAGGACAAGCAGGAGCAGCAGGAGCAGCAGGAGCAGCAGGACAAGGAGGAGCAGCAGGACAAGGAGGAGCAGCAGGACAAGGAGGAGCAGCTGGACAAGGAGGAGCAGCAGGAAAAGGAGGAGGAGCAGGCCAACCAGGAGCAGCAGGACAAGGAGGAGCAGCAAGAGCAGGAGCAGGGGGAAATTCGGGAGGTGAAAATATCCCAGATGCAAAGGTTGTGAAAGGTTACCTAGACAAAGTGAGACCTACCCTTACCACCGAATGGAGTGCATGCAGTGTAACCTGTGGAACGGGTGTAAGAGTTAGAAGAAAAGTTAGTGCATCAAACAAGAAACCAGAGGAACTTACTACGGATGACGTTGAGACAGAAATTTGTAAAATGGATAAGTGCGCTAGCATATTTAACGTTGTGAGTAACTCATTAGGGGTAGTCATATTGTTAGTCATAGCATTATTCAATTAA------------------------------------------------------------------------------------------------------------------------------------------------------------------------------------------------------------------------------------------------------------------------------------------

>GU002522 P. coatneyi_outgroup

ATGAAGAACTTCATTCTCTTGGCCGTTTCTTCCATCCTGTTGGTGGACTTGTTCCCCACGCACTTCGGACATAATGTAGATCTCTCCAGGGCCATAAATTTAAATGGAGTAAGCTTCAATAATGTAGACACCAGTTTACTTGGCGCAGCACAGGTAAGACAAAGTGCTAGCCGAGGCAGAGGACTTGGTGAGAAACCAAAAAAAAAGGCGGAAAAAAAAGAAGAAGAACCAAAAAAGCCAAATGAAAATAAGCTGAAGCAACCAGTAGATGGAGCACGAGATGGGCCAGCACCAGCAGCAGATGGAGCAAGAGATGGACCAGCACCAGCAGCAGATGGAGCACGAGATGGACCAGCACCAGCAGCAGATGGAGCAAGAGATGGACCAGCACCAGCAGCAGATGGAGCAAGAGATGGACCAGCACCAGCAGCAGATGGAGCAAGAGATGGACCAGCACCAGCAGCAGATGGAGCACGAGATGGACCAGCACCAGCAGCAGATGGAGCAAGAGATGGGCCAGCACCACCAGCCGATGGAGCAAGAGATGGGCCAGCACCACCAGCAGCAGATGGAGCACGAGATGGACCAGCACCACCAGCAGCAGATGGAGCACGAGATGGGCCAGCACCACCAGCAGGACAAGGAGGAGGAAATGCAGCAGGCCAAGCACAAGGAGGAGGAAATGCCGGAAACAAAAAAGCAGGAGACGCAGCTGGAAACGCAGGAGCAGCAAAAGGACAGGGACAAAATAATGAAGGTGCGAATGTCCCAAATGAGAAAGTTGTGAATGATTACCTACAGAAAATTAGATCTACCGTTACCACCGAATGGACTCCATGCAGTGTAACCTGTGGAAATGGTGTAAGACTTAGAAGAAAAGCTCATGCAGAAAAGAAAAAACCAGAGGACCTTACCATGGATGACCTTGACGTGGAAGTTTGTGCAATGGATAAGTGCGCTGGCATATTTAACTTTGTGAGTAATTCATTAGGGCTAGTCATATTGTTAGTCCTAGCATTATTCAATTAA------------------------------------------------------------------------------------------------------------------------------------------------------------------------------------------------------------------------------------------------------------------------------------------------------------------------------------------------------------------------------------------------------------------------------------------------------------------------
